# Blockage of PPARγ T166 phosphorylation enhances the inducibility of beige adipocytes and improves metabolic dysfunctions

**DOI:** 10.1038/s41418-022-01077-x

**Published:** 2022-11-03

**Authors:** Nanfei Yang, Yuxin Wang, Qiang Tian, Qiuping Wang, Yan Lu, Luchen Sun, Sijie Wang, Yuncheng Bei, Jianguo Ji, Hu Zhou, Wei Yang, Pengju Yao, Wenyuan Zhu, Lingyun Sun, Zhifeng Huang, Xiaokun Li, Pingping Shen

**Affiliations:** 1 State Key Laboratory of Pharmaceutical Biotechnology, Department of Rheumatology and Immunology, The Affiliated Nanjing Drum Tower Hospital, The Affiliated Hospital of Nanjing University Medical School, School of Life Sciences, Nanjing University, Nanjing, 210023 China; 2 School of Pharmaceutical Science, Wenzhou Medical University, Wenzhou, Zhejiang 325035 China; 3 State Key Laboratory of Protein and Plant Gene Research, School of Life Sciences, Peking University, Beijing, 100871 China; 4 Department of Analytical Chemistry and CAS Key Laboratory of Receptor Research, Shanghai Institute of Materia Medica, Chinese Academy of Sciences, 555 Zuchongzhi Road, Shanghai, 201203 China; 5 Department of Medicine, David Geffen School of Medicine, University of California Los Angeles, Los Angeles, CA 90095 USA; 6 Department of Rheumatology and Immunology, the Affiliated Drum Tower Hospital of Nanjing University Medical School, 321 Zhongshan Road, Nanjing, 210008 China; 7 Oujiang Laboratory (Zhejiang Lab for Regenerative Medicine, Vision and Brain Health) & School of Pharmaceutical Science, Wenzhou Medical University, Wenzhou, Zhejiang 325035 China; 8 Shenzhen Research Institute of Nanjing University, Shenzhen, 518000 China

**Keywords:** Cell biology, Molecular biology

## Abstract

Beige adipocytes in mammalian white adipose tissue (WAT) can reinforce fat catabolism and energy expenditure. Promoting beige adipocyte biogenesis is a tantalizing tactic for combating obesity and its associated metabolic disorders. Here, we report that a previously unidentified phosphorylation pattern (Thr166) in the DNA-binding domain of PPARγ regulates the inducibility of beige adipocytes. This unique posttranslational modification (PTM) pattern influences allosteric communication between PPARγ and DNA or coactivators, which impedes the PPARγ-mediated transactivation of beige cell-related gene expression in WAT. The genetic mutation mimicking T166 phosphorylation (p-T166) hinders the inducibility of beige adipocytes. In contrast, genetic or chemical intervention in this PTM pattern favors beige cell formation. Moreover, inhibition of p-T166 attenuates metabolic dysfunction in obese mice. Our results uncover a mechanism involved in beige cell fate determination. Moreover, our discoveries provide a promising strategy for guiding the development of novel PPARγ agonists for the treatment of obesity and related metabolic disorders.

## Introduction

White adipose tissue (WAT) plays a central role in whole-body metabolic homeostasis [1]. WAT incorporates excess nutrients into lipid droplets, buffering metabolic stress and avoiding lipid toxicity to peripheral metabolic organs [2]. However, during obesity, adipocyte hypertrophy in WAT disturbs the normal energy balance, turning adipose tissue into inflamed tissue and further leading to ectopic fat deposition, which together result in insulin resistance and multitudinous complications [3–5]. Thus, restoration of the balance of energy consumption and expenditure in WAT is critical for attenuating WAT metabolic dysfunctions [6].

Beige adipocytes (or beige cells) are a type of inducible brown-like fat cell in WAT. Beige adipocyte biogenesis represents an elaborate process of adaptive metabolic remodeling in WAT [7–9]. The generated beige cells possess increased lipid catabolism and mitochondrial respiration, which accelerate the energy expenditure of WAT [10, 11]. Moreover, recent studies have demonstrated the crucial roles of beige cells beyond energy regulation. They act as endocrine cells that secrete bioactive factors to improve insulin resistance and inflammation. Due to this promising role, inducing beige adipocyte generation is considered a tantalizing tactic for combating metabolic disorders [12].

PPARγ is indispensable for adipocyte differentiation. Fat-specific ablation of PPARγ causes lipoatrophy and severe metabolic disturbance [13, 14]. In overnutrition state, PPARγ promotes healthy WAT expansion and maintains metabolic homeostasis [15]. However, in pathologic obesity state, the function of PPARγ is dysregulated by several PTMs [16–19], which change its transactivation behavior and further aggravating obesity related metabolic disorders. Pharmacological treatment with thiazolidinediones (TZDs), classical PPARγ full agonists, regulates these PTMs, strongly reverses the unhealthy WAT remodeling, and improves metabolic disorders. Notably, TZDs dramatically induce the beige adipocyte biogenesis in WAT [20]. TZD activates PPARγ, enhance its binding to PRDM16 or PGC1α (pro-browning cofactors), and increases beige cell gene expression [18, 21]. Meanwhile, TZDs reinforce the cooperation of KLF11 and PPARγ, and sustain the histone modifications in beige-selective genes [22]. However, TZDs treatment cause several severe side effects, such as weight gain, osteoporosis, fluid retention and heart failure [23, 24]. Till date, few selective PPARγ modulators (SPPARMs) were proven to exhibit pro-browning effect in WAT. One major reason is the mechanisms that molecular fine-tuning of PPARγ and recognition of cell-specific genes remain elusive.

Here, we identified a previously unknown phosphorylation pattern in the DNA-binding domain (DBD) of PPARγ (p-T166), which was proven to be a molecular regulator in beige cell development. Phosphorylation of this threonine site by PKCα impeded the inducibility of beige adipocyte and lipid catabolism. p-T166 alters the molecular behavior of PPARγ, which disrupts its binding to transcriptional cofactors and regulates the expression of beige cell-related lipid catabolism genes. More importantly, the level of p-T166 is positively correlated with the obese state, and specific blockage of p-T166 can prevent obesity related metabolic dysfunctions.

## Results

### Identification of T166 phosphorylation pattern in PPARγ

Previously, we found that CDDO, a ligand binding to PPARγ ligand binding domain (LBD) (Fig. S1a) [25, 26], markedly increased the beige cell marker expressions and enhanced binding of pro-browning cofactor PRDM16 to PPARγ (Fig. S1a–d). The analogue, CDDO-Me (C28 carboxyl of CDDO was replaced with carboxymethyl, also the ligand of PPARγ [25]) could not effectively induce beige cell gene expression (Fig. S1b, c). The effect of CDDO on beige cell differentiation was not due to simply driving overall adipogenesis because when the mRNA levels of *UCP1* and *Cidea* were normalized to the classical adipogenic marker gene *aP2* (also known as *Fabp4*), the induction of these genes was still significant (Fig. S1e). In parallel, compound SO1989, a CDDO analogue we have previously reported that could promote mitochondrial biogenesis and fatty acid oxidation in macrophages [27], was used in the experiment. As Fig. S1a–c shown, SO1989 cannot promote beige cell differentiation. These intriguing discoveries led us to further investigate the conformational perturbations of PPARγ upon CDDO binding.

**Fig. 1:**
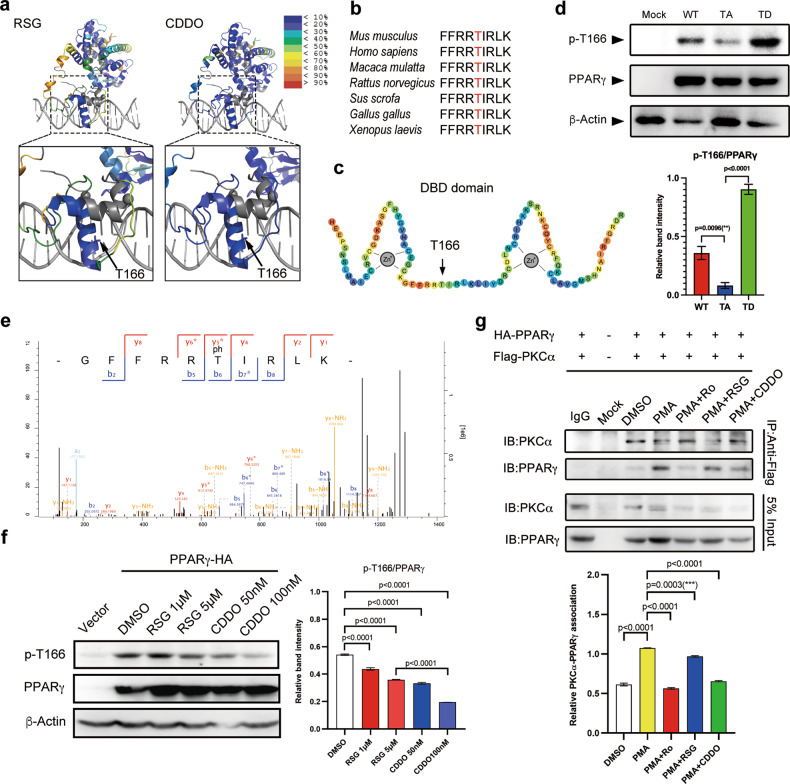
PKCα-mediated PPARγ phosphorylation at T166 can be inhibited by CDDO. **a** Differential HDX mass spectrometry data for PPARγ (PDB:3e00) with rosiglitazone (RSG) and CDDO. The ribbon diagram is colored according to HDX stabilization/destabilization. Percentages of deuterium difference are color-coded according to the color gradient key. **b** Sequence alignment of the PKCα catalytic consensus site in PPARγ, which is conserved in mammals. **c** Schematic representation of the PPARγ DBD revealing that T166 is located between two zinc finger structures. **d** Detection of PPARγ phosphorylation at T166 in HEK293T cells with overexpressing WT, TA mutant, or TD mutant PPARγ, or without overexpression (Mock). Experiments were repeated three times. **e** Annotation of MS/MS spectra of the peptides of PPARγ phosphorylated at T166. **f** Treatment of HEK293T cells overexpressing WT PPARγ with RSG or CDDO followed by the detection of p-T166. Experiments were repeated three times. **g** Co-immunoprecipitation of WT PPARγ and exogenous PKCα from HEK293T cells treated with PMA (100 nM) and Ro (2 μM), RSG (1 μM), or CDDO (100 nM). Experiments were repeated three times. Data were analyzed by one-way ANOVA followed by Tukey’s test (**d**, **f**, **g**). Data are presented as mean ± SEM. *P < 0.05. **P < 0.01, ***P < 0.001.

Next, we performed differential hydrogen deuterium exchange (HDX) analysis and found that the CDDO−PPARγ complex exhibited a profile distinct from that of the RSG positive control (Fig. S2a, Supplementary dataset 1–2). CDDO binding to the LBD caused a large magnitude change in the DBD (Fig. S2a, Fig. 1a), particularly at residues 148−155 and 161−169 (Fig. S2b), which are located in the first zinc finger. Notably, CDDO-Me did not induce a similar effect in these regions (Fig. S2a, Supplementary dataset 3), suggesting that the C28 carboxyl in the CDDO molecule was critical for CDDO-dependent conformational fine-tuning in the first zinc finger, and the conformational alteration in the DBD might be linked to PPARγ function in beige cell fate determination.

Subsequently, we noticed that the conserved FFRRTIRLK motif at residues 161-169 contained a potential phosphorylation site at Thr166 (corresponding to the Thr136 site in PPARγ_1_) (Fig. 1b, c). To verify that p-T166 can occur, we generated a mouse monoclonal antibody against p-T166 and used it to detect the phosphorylation signal. In 293 T cells, overexpressed TD (T166D, aspartic acid mutation) exhibited a stronger luminescence level than WT and TA (T166A, alanine mutation) (Fig. 1d), while TA dramatically decreased the signal, suggesting that the T166 phosphorylation occurred in cells. Furthermore, to determine the upstream kinase, the phosphorylation site scanning server (http://scansite4.mit.edu) was used and two highly likely candidates: PKCα and Aurora B (AuroB) were predicted. Overnight incubation of PPARγ-overexpressing 293 T cells with Ro-31-8220 (PKCα inhibitor) led to significant attenuation of p-T166 (Fig. S3a). In addition, PMA (a PKC activator) significantly stimulated the activation of PKCα and increased p-T166 level (Fig. S3b). To verify the direct interplay between PKCα and PPARγ, we performed an in vitro kinase assay coupled with HPLC‒MS/MS, which showed that PKCα exclusively phosphorylated PPARγ at the T166 site (Fig. S3c, Fig. 1e). Moreover, molecular modeling revealed that the catalytic center of PKCα contained a binding pocket for the FFRRTIRLK motif located in the DBD helix (Fig. S3d). In cells, the immediate interaction between PKCα and PPARγ could be disrupted by PKC inhibition (Fig. S3e), confirming the kinase−substrate relationship.

Considering the robust impact of CDDO on the structural conformation of the PPARγ DBD, we next evaluated the efficiency and specificity of this ligand for p-T166 intervention. We treated 293 T cells overexpressing WT-PPARγ with ligands and evaluated the p-T166 level. The results showed that a CDDO concentration of 100 nM was sufficient to attenuate p-T166 levels (Fig. 1f). By detecting T638 phosphorylation of PKCα (a marker for PKCα activation), we found that CDDO did not inhibit PKCα activation (Fig. S3f). Co-immunoprecipitation (Co-IP) assays showed that the interaction of PKCα and PPARγ could be interrupted by CDDO (Fig. 1g). These results suggest that the inhibitory effect of CDDO on p-T166 might not rely on the influence of PKCα activation but instead was a result of the inhibition of PPARγ binding to PKCα. Finally, we found that CDDO did not inhibit Ser112 or Ser273 phosphorylation (two previously identified phosphorylation sites) (Fig. S3g, h). These results suggest that CDDO interrupts the PKCα-PPARγ axis and inhibits p-T166.

Taken together, these data provide evidence that PKCα-mediated PPARγ phosphorylation at T166 is a novel PTM pattern.

### T166 phosphorylation pattern in primary adipocytes can be inhibited by CDDO

**Fig. 2:**
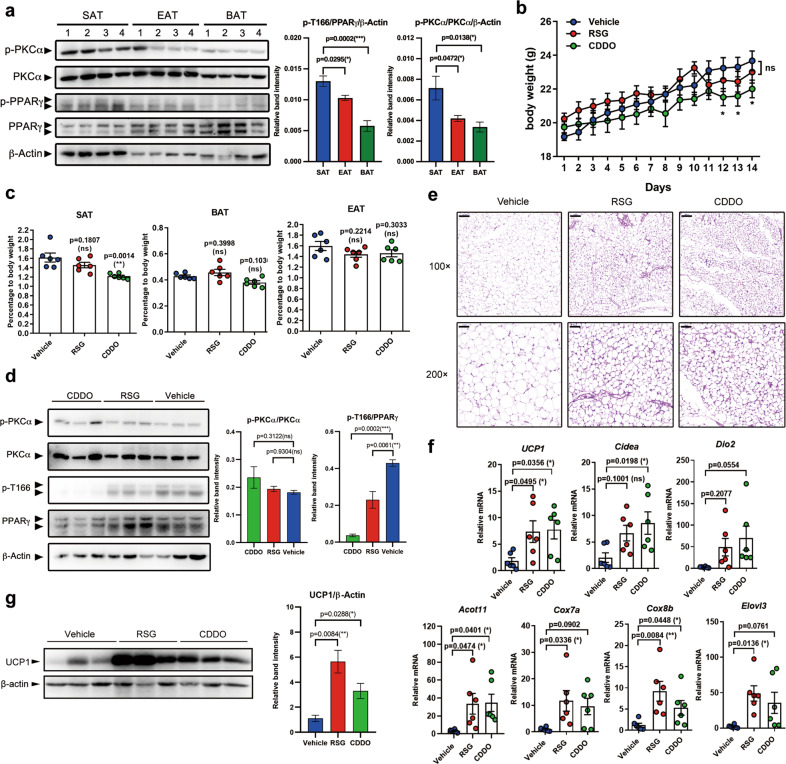
CDDO inhibits p-T166 and promotes the biogenesis of beige adipocytes. **a** Isolated subcutaneous adipose tissue (SAT), epididymal adipose tissue (EAT), and brown adipose tissue (BATs) were homogenized. The whole tissue protein was analyzed by western blotting to evaluate the levels of p-PKCα and PPARγ p-T166 (*n* = 4). **b** Body weight curve of 6-week-old C57/B6J mice treated with vehicle, RSG (5 mg/kg), or CDDO (3 mg/kg) for 14 days. **c** The content of adipose fat pads (percentage of body weight) from mice in the three groups. **d** The pure SAT adipocytes were isolated by using collagenase digestion method. The levels of PPARγ p-T166, PPARγ, p-PKCα, and PKCα in adipocytes were detected by western blotting. Each lane contains total protein from two mice. **e** H&E staining of SAT. 100× magnification, scale bar, 100 μm; 200× magnification, scale bar, 50 μm. **f** Relative mRNA levels of browning-related genes in SAT. Gene expression is normalized to the 36B4 endogenous control. **g** Detection of the level of UCP1 in SAT. Each lane contains total protein from two mice. β-Actin was the endogenous control. Biologically independent samples: (**b**–**g**), there were 6 mice in each group (*n* = 6). Data are expressed as the mean ± S.E.M. Data were analyzed by two-way ANOVA followed by Bonferroni’s test (**b**) or one-way ANOVA followed by Tukey’s test (**a**, **c**, **d, f, g**). *P < 0.05. **P < 0.01, ***P < 0.001.

We next evaluated the p-T166 level and PKCα activation in adipose tissue. Subcutaneous adipose tissue (SAT) showed significant higher levels of p-T166 and p-PKCα than brown adipose tissue (BAT) and epididymal adipose tissue (EAT) (Fig. 2a), suggesting that p-T166 could occur in SAT under basal physiological conditions. Next, CDDO was used to induce brown remodeling in vivo (rosiglitazone was the positive control). After 14 days treatment, CDDO reduced mice body weight and SAT mass (Fig. 2b, c). By evaluating the histological features of SAT, we found that both CDDO significantly induced multilocular UCP1^+^ adipocyte biogenesis (Fig. 2e, Fig. S4a). At both mRNA and protein level, CDDO significantly induced beige cell marker expression (Fig. 2g–f). By quantification of the p-T166 level in SAT isolated adipocytes, we found that CDDO exhibited a strong effect on p-T166 inhibition (Fig. 2d). The in vitro study on SVF-differentiated adipocytes also verified that CDDO can reduce the p-T166 level in differentiated primary adipocytes (Fig. S4b). Meanwhile, the mitochondrial membrane potential was greatly increased by CDDO (Fig. S4c). Next, we collected human adipose samples through liposuction and performed SVF cell differentiation in vitro. After 10 days of differentiation treated with CDDO, we found that CDDO could significantly inhibit p-T166 and enhance UCP1 expression in human adipocytes (Fig. S4d–h). All these data indicate that p-T166 in primary adipocytes can be inhibited by CDDO, and this PTM might be involved in WAT browning.

### T166 phosphorylation restrains the inducibility of beige adipocytes

To further study the role of p-T166 in beige cell induction, we generated two global PPARγ T166 mutant mouse strains, T166A (TA) and T166D (TD), to mimic the persistent in vivo dephosphorylated or phosphorylated status, respectively (Fig. S5a). cDNA sequencing of the *pparg* gene locus verified the successful homologous recombination of the WT allele with the TA or TD mutant (Fig. S5b). To prove the mutations at protein level, we further used the western blotting to analyze the p-T166 level in SAT and SVF cell differentiated adipocytes. As shown in Fig. S5c, d, p-T166 antibody discriminated the mutations, which also confirmed that the 166 threonine was successfully mutated to alanine or aspartic acid. Next, we found that on a standard chow diet, the body weight of homozygous TA mice at 8 weeks was lower than that of their WT) and TD counterparts (Fig. S5e). Dual-energy X-ray absorptiometry (DEXA) scanning showed that TA mice had a reduction in total fat percentage (Fig. S5f–g). Further quantitative analysis of adipose content indicated that TA mice had a slightly lower SAT content (Fig. S5h). To analyze the morphological characteristics of adipocytes in adipose tissues, TissueGnostics imaging system was used. By measuring the lipid droplet size (Fig. S5i), we found that lipid deposition was slightly increased in both SAT and BAT from TD mice, while it was reduced in samples from TA mice (Fig. S5j–k). No significant differences in beige cell marker expression in SAT among the three genotypes (Fig. S5l–m).

**Fig. 3:**
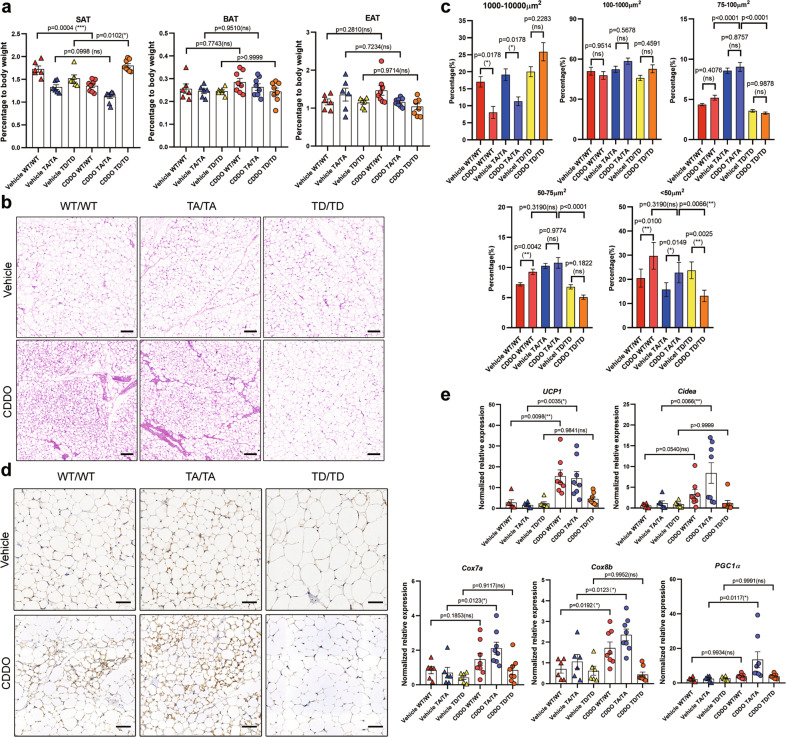
T166 A/D mutation regulates the CDDO mediated beigeing program in SAT. **a** 8-week-old WT/WT, TA/TA and TD/TD mice treated with vehicle or CDDO (3 mg/kg) for 14 days. Three kinds of adipose fat pads (percentage of body weight) from WT/WT, TA/TA and TD/TD mice. (Vehicle WT/WT group *n* = 6; Vehicle TA/TA group *n* = 6; Vehicle TD/TD group *n* = 6; CDDO WT/WT group *n* = 8; CDDO TA/TA group *n* = 8; CDDO TD/TD group *n* = 8). **b** Hematoxylin and eosin (H&E) staining of SAT. 100× magnification, scale bar, 100 μm. (*n* = 5) (**c**) Quantitation of the size and percentage of lipid droplets in SAT by TissueFAXS Cytometry analysis. (*n* = 5) (**d**) UCP1 immunohistochemical staining of SAT. 200× magnification, scale bar, 50 μm. (*n* = 5) (**e**) Relative mRNA levels of browning-related genes in isolated adipocytes from SAT. (Vehicle WT/WT group *n* = 6; Vehicle TA/TA group *n* = 6; Vehicle TD/TD group *n* = 6; CDDO WT/WT group *n* = 8; CDDO TA/TA group *n* = 8; CDDO TD/TD group *n* = 8). Data are expressed as the mean ± S.E.M. Data were analyzed by one-way ANOVA followed by Tukey’s test (**a**, **c**, **e**). *P < 0.05, **P < 0.01, ***P < 0.001.

We next test the inducibility of beige adipocytes in T166 mutant mice. Upon 14 days of CDDO treatment, TD blocked the CDDO-induced reduction in SAT content in WT controls (Fig. 3a). Strikingly, in contrast to WT and TA mice that generated a large number of multilocular UCP1^+^ adipocytes in the SAT, TD mice had dramatically impaired beige cell recruitment (Fig. 3b–d). The damage to WAT browning in TD mice was also reflected by the quantity of beige cell markers (Fig. 3e, Fig. S7a). Furthermore, we analyzed the morphological transformation of adipocytes. In WT and TA SAT depots, the percentage of large lipid droplets (1000-10000 μm^2^) was dramatically reduced in the CDDO-treated group. However, TD promoted the formation of large lipid droplets upon drug administration (Fig. 3c). On the other hand, TA promoted the formation of small lipid droplets (<100 μm^2^). These intriguing results suggested that p-T166 inhibits CDDO-induced “white-to-beige” conversion in SATs. To further confirm that p-T166 mediated PPARγ activation-induced WAT browning, we further treated mice with RSG to establish a classical WAT browning model. Three genotypes of mice were administered RSG for 7 days. The histological analysis revealed that in contrast to WT and TA mice, which generated a large number of multilocular adipocytes containing small lipid droplets in the SAT, TD mice resisted the RSG-elicited morphological remodeling (Fig. S6a, b). The quantification of the mRNA levels of beige cell markers showed that TD impaired RSG-induced browning gene expression (Fig. S6c). Taken together, these findings indicate that p-T166 impedes PPARγ ligand-induced beige cell recruitment in vivo.

### T166 phosphorylation modulates the transcriptional action of PPARγ

**Fig. 4:**
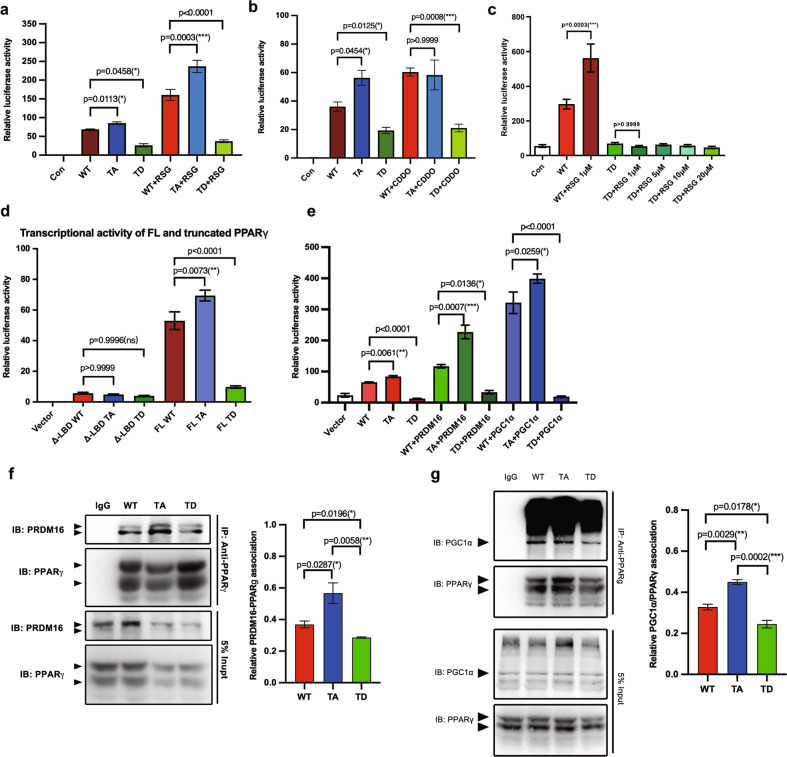
p-T166 in the DBD modulates the transcriptional action of PPARγ. **a**–**e** Luciferase reporter assay evaluating the transcriptional activity of (**a**), WT, TA, and TD following treatment with vehicle or RSG; (**b**) WT, TA, and TD following treatment with vehicle or CDDO; (**c**) WT PPARγ under a gradient concentration of RSG; (**d**) Full-length or LBD-truncated PPARγ; (**e**) PRDM16 or PGC1α co-expressed with WT or mutant PPARγ. In (**a**–**e**), *n* = 3. **f** Co-immunoprecipitation of endogenous PPARγ with PRDM16 in PPARγ homozygous WT, TA, and TD SVF differentiated primary adipocytes. Experiments were repeated three times. **g** Co-IP analysis of the interaction between endogenous PPARγ and PGC1α in the three genotypes SVF differentiated primary adipocytes. Experiments were repeated three times. Data are expressed as the mean ± S.E.M. Data were analyzed by one-way ANOVA followed by Tukey’s test (**a**–**e**, **f**, **g**). *P < 0.05. **P < 0.01, ***P < 0.001.

To examine mechanism of p-T166 in beige cell induction, we evaluated the effects of this PTM on PPARγ transcriptional action. In 293 T cells, luciferase reporter assays revealed that TD sharply attenuated the transcriptional activity of PPARγ at PPRE (PPAR response element) (Fig. 4a). Upon treatment with PPARγ agonists, TD completely blocked RSG- or CDDO-induced PPARγ activation (Fig. 4a–c). Because the ligand-induced transcriptional activation of PPARγ relies on allosteric communication between the ligand-dependent domain (LBD) and DBD, we speculated that p-T166 disrupted this effect. We overexpressed truncated PPARγ (Δ-LBD, LBD deletion) and evaluated its transcriptional activity. The three types of Δ-LBD PPARγ had a similar basal PPRE binding activity; however, following readdition of the LBD to the truncated proteins (the full-length protein), TD destroyed the LBD-activated DBD binding at PPRE, abolishing its transcriptional activity (Fig. 4d). This result suggested that p-T166 disturbed communication between the LBD and DBD. Subsequently, we added pro-beigeing cofactors to the transcriptional assay system. The co-factor-elicited enhancement of transactivation in the WT and TA groups was completely abrogated by TD (Fig. 4e), revealing that p-T166 disables co-factor-determined recognition and transactivation.

Previous studies demonstrated that the loss of function in PPARγ transcriptional activity causes lipoatrophy [28, 29]. However, in our model, TD mice generated similar volumes of BAT and WAT as those found in WT mice. Additionally, after 10 days of differentiation in vitro, the expression levels of adipogenesis genes (*PPARγ* and *adiponectin*) in TD were slightly lower than those in WT (Fig. S7c), TD adipocytes still had similar lipid storage ability as their WT counterparts (Fig. S7b). These data indicated that p-T166 probably induced an unknown compensatory mechanism to maintain normal adipogenesis. In contrast, TD dramatically impeded CDDO-induced beige cell-specific marker expression (Fig. S7d, e), while little impacting white adipocyte-related genes (Fig. S7f). These results indicate that the beige cell program was sensitive to p-T166. Next, Co-IP showed that in primary cultured differentiated adipocytes, TA reinforced the binding of PRDM16 and PGC1α to PPARγ. In contrast, TD inhibited the interplay between PPARγ and PGC1α (Fig. 4f–g). These data suggested that p-T166 could regulate the assembly of the pro-browning transcriptional complex. Finally, structural modeling highlighted that following phosphorylation of T166, there was a steric clash between T166 and F162 or F195, which led to fine-tuning of the DBD conformation (Fig. S8).

Taken together, these data show that p-T166 in the DBD regulates the transcriptional action of PPARγ in determining the beige cell phenotype.

### T166 phosphorylation reprograms lipid metabolism in adipocytes

**Fig. 5:**
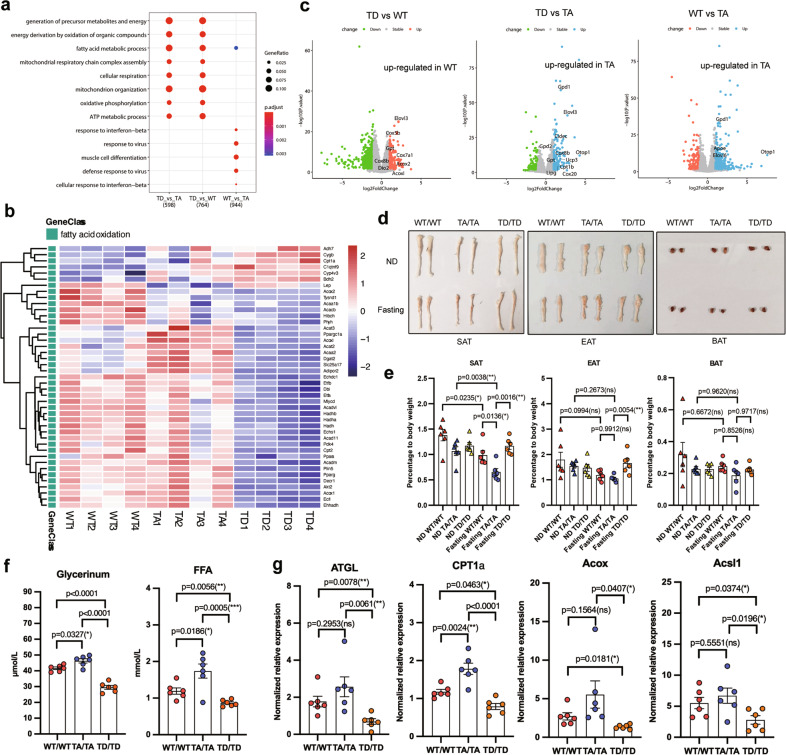
T166 phosphorylation controls fat degradation and lipid catabolism. **a** GO analysis of differentially expressed genes among the three genotypes (*n* = 4). **b** Expression of fatty acid oxidation genes by RNA-Seq (*n* = 4). *P* < 0.05 according to an unpaired two-sided Student’s *t*-test and LogFC >0.5. **c** Volcano plot showing beige cell markers. (**d**) The gross appearance of adipose tissue from homozygous WT/WT, TA/TA and TD/TD mice following 3 h 4°C acute cold challenge pretreated with overnight fasting (*n* = 6). **e** The content of adipose fat pads (percentage of body weight) from WT/WT, TA/TA and TD/TD mice (*n* = 6). **f** Serum free fatty acid (FFA) and glycerin levels (*n* = 6). **g** Q-PCR analysis of lipid catabolism genes in isolated adipocytes from SATs (*n* = 6). Data are expressed as the mean ± S.E.M. Data were analyzed using one-way ANOVA followed by Tukey’s test (**e**–**g**). *P < 0.05. **P < 0.01, ***P < 0.001.

PPARγ activation can simultaneously increase the gene expressions of lipid catabolism and anabolism during beige adipocyte development [30–32]. To explore the effect of p-T166 on lipid metabolism, we performed unbiased RNA-Seq in SVF-differentiated SAT adipocytes. The results showed that homozygous WT, TA, and TD adipocytes had distinct molecular signatures (Fig. S9b). Gene Ontology enrichment (GO) indicated that p-T166 controlled fatty acid metabolism and energy derivative production (Fig. 5a). Gene set enrichment analysis (GSEA) indicated that p-T166 regulated several significant browning-related pathways, including oxidative phosphorylation, fatty acid metabolism and adrenergic signaling (Fig. S9f). With respect to the lipid biosynthetic process, each genotype enriched a unique profile of gene sets despite the outcomes of lipid storage being similar (Fig. S9a, Fig. S7b). In contrast, TD impeded most lipid catabolic gene expressions, especially those related to the fatty acid catabolic process (Fig. S9e and S9g). Some genes involved in fatty acid oxidation were highly enriched in TA cells, such as *Acat3*, *Ppargc1a* and *Acox* (Fig. 5b). Compared with WT and TD, TA enhanced the expression of some beige cell-specific genes (*Otop1*, *Elovl3*, *Cox8b*, *Cidec*, etc.) (Fig. 5c), indicating that in vitro differentiated TA adipocytes autonomously skewed to a beige cell phenotype. All of these data indicate that p-T166 specifically inhibits the fatty acid catabolism pathway. Next, we applied the differentially expressed genes in ChIP-X enrichment analysis (ChEA; https://maayanlab.cloud/chea3/). This method enriched PPARγ-transcribed genes in T166 mutant adipocytes based on the ChIP-seq databases (Table S2). We found that TA PPARγ specifically transcribed fatty acid oxidation genes, such as *ACAA2* (the enzyme catalyzes mitochondrial beta-oxidation), *CPT2* (deliver fatty acids into mitochondria) and *Acsl1* (convert fatty acids to acyl-CoAs in the β-oxidation process) (Fig. S9c). To confirm the bioinformatics analysis, we subjected cultured adipocytes to ChIP‒qPCR analysis. As expected, TA preferred to bind the promoters/enhancers of lipid catabolism-related genes enriched by ChEA, while TD inhibited this effect (Fig. S9d). These results showed that p-T166 regulates PPARγ transcriptional activity for fatty acid catabolism genes.

As lipid catabolism is closely associated with mitochondrial activity, we clustered the mitochondrial assembly and oxidative phosphorylation pathways. TD adipocytes showed inhibition of both processes (Fig. S10a, b). Subsequent fluorescence microscopy and bioenergetics measurements confirmed that TD adipocytes exhibited a markedly decreased mitochondrial membrane potential (Fig. S10c) and oxygen consumption rate (OCR) (Fig. S10d), indicating that p-T166 inhibits mitochondrial activity.

After recognizing the role of p-T166 in regulating lipid metabolism, we next sought to explore its related physiological function. Homozygous WT, TA and TD mice were fasted overnight and then subjected to a 6-h acute cold challenge. Intriguingly, this condition quickly induced lipid breakdown in TA-SATs, directly causing a severe reduction in adipose mass (Fig. 5d, e). Correspondingly, after the cold challenge, the serum concentrations of free fatty acids (FFAs) and glycerin in TA mice were higher than those of their WT and TD counterparts (Fig. 5f), suggesting that TA rapidly promoted lipid catabolism. Moreover, lipid catabolism genes were highly expressed in TA-SATs (Fig. 5g), while TD inhibited this effect. In parallel, we used isoprenaline (ISO) to activate β-adrenergic receptor (β-AR) signaling, a key pathway in cold-induced beige cell formation and lipid mobilization. In cultured adipocytes, TA cells showed enhanced beige cells and related lipid catabolism genes relative to their WT or homozygous TD counterparts in response to 6 h of ISO treatment. In contrast, TD inhibited the expression of these genes (Fig. S10e). All of these phenomena indicate that p-T166 controls lipid catabolism in adipocytes.

### T166 phosphorylation correlates with metabolic dysfunctions

To study the correlation between metabolic disturbance and p-T166, we challenged WT mice with a high-fat diet (HFD) and analyzed the serum metabolic markers, in parallel with evaluating the p-T166 level in SAT. We found that the 4-month HFD treatment induced marked metabolic dysfunction in mice. The serum content of adiponectin, an insulin-sensitizing and anti-inflammatory adipokine, was decreased (Fig. S11a), while the concentrations of serum triacylglycerol, glucose and cholesterol were significantly increased (Fig. S11b). Moreover, proinflammatory cytokines (TNFα and IL-6) were also dramatically induced (Fig. S11c). Next, we found that the level of p-T166 in SAT was highly increased upon the 4-month HFD treatment (Fig. S11d). Subsequently, we readapted 4-month HFD-fed mice to 8 weeks of normal diet feeding (HFD converted to chow diets, HCC model), which improved metabolic disorders (hyperlipidemia, hypercholesterolemia, and insulin resistance) (Fig. S11e, S11g, h). Moreover, HCC attenuated p-T166 intensity in SAT (Fig. S11f). We also assessed other phosphorylation patterns in SAT, and we found that HCC treatment had little impact on S112 or S273 phosphorylation (Fig. S11i). These results indicate that the p-T166 level is correlated with metabolic dysfunction.

### Genetical blockage of T166 phosphorylation enhances energy expenditure and improves metabolic dysfunctions

**Fig. 6:**
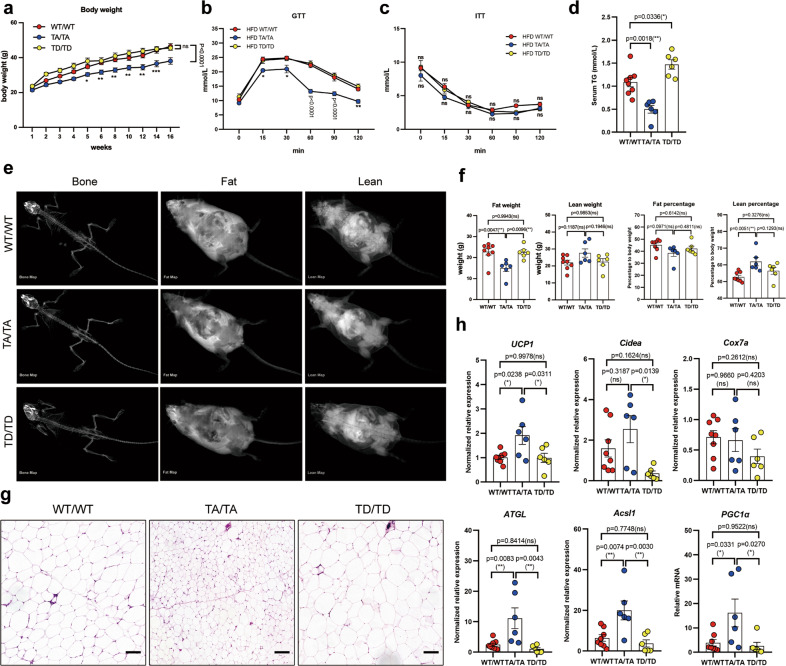
Blockade of T166 phosphorylation ameliorates HFD-induced obesity and related metabolic dysfunction. **a** Body weight curve of WT/WT, TA/TA and TD/TD mice treated with an HFD for 16 weeks. **b**, **c** Glucose tolerance test (GTT) (**b**) and insulin tolerance test (ITT) (**c**) in WT, TA and TD mice fed an HFD for 12 weeks. **d** The total triglyceride content in the serum was measured using a commercial kit. **e**, **f** Dual-energy X-ray absorptiometry (DEXA) scans showing (**e**) lean mass/percentage and fat mass/percentage (**f**) of mice following an HFD. **g** H&E staining of SAT in HFD-fed mice. 100× magnification; scale bar, 100 μm. **h** Relative mRNA levels of browning-related genes in isolated adipocytes from SATs. Gene expression is normalized to the 36B4 endogenous control. Biologically independent samples: WT/WT group *n* = 8; TA/TA group *n* = 6; TD/TD group *n* = 6. In (**g**), 100× magnification; scale bar, 100 μm. Data are expressed as the mean ± S.E.M. Data were analyzed by two-way ANOVA followed by Bonferroni’s test (**a**–**c**) or one-way ANOVA followed by Tukey’s test (**d**, **f**, **h**). *P < 0.05. **P < 0.01, ***P < 0.001.

We then included TA and TD mice in the analysis. Homozygous WT, TA and TD mice were simultaneously fed a HFD, and the metabolic index levels were evaluated. Surprisingly, TA mice showed resistance to diet-induced obesity, improved insulin sensitivity, and lower fasting lipidemia than their WT and TD counterparts (Fig. 6a-d). DEXA scanning indicated that HFD-treated TA mice had a decreased fat mass/percentage and a higher lean percentage (Fig. 6e-f). Furthermore, TA mice had decreased BAT and SAT contents (Fig. S12f, Fig. 6g). Subsequent mRNA quantitation in SAT revealed that TA had higher expressions of beige biomarker genes (Fig. 6h). The metabolic cage assay was used to analyze energy homeostasis. TA mice consumed a much higher volume of O_2_ and produced a greater volume of CO_2_ than WT and TD mice (Fig. S12a, b). Correspondingly, the heat production of TA mice was higher than that of their WT and TD counterparts (Fig. S12c). The respiratory exchange ratio (RER) in TD mice was lower than that in mice of the other genotypes (Fig. S12d). Analysis of the EAT uncovered an intriguing phenomenon where the EAT from TA mice was larger than that from WT mice (Fig. S12f) but ameliorated inflammatory cell infiltration, suggesting that TA might promote the healthy expansion of EAT (Fig. S12g). The evaluation of peripheral metabolic organs, the liver and skeletal muscle, indicated that TA mice exhibited greatly alleviated steatosis in these tissues (Fig. S12h). Taken together, these data confirm that p-T166 regulates adipose remodeling; more importantly, inhibition of p-T166 can improve obesity related metabolic disorders.

## Discussion

The regulatory diversity and complexity of PPARγ governing the adipocyte development is largely unknown. The PTMs located in the PPARγ-LBD control beige cell differentiation [18, 33] by altering LBD conformation and impeding the interaction of PPARγ with pro-browning cofactors. In contrast, DBD has been defined as the “performer” of the commands given by the LBD [34]. With respect to the nuclear receptor family (NR), no evidence has successfully highlighted the significance of the DBD in determining cell-specific programs. Here, for the first time, we identified p-T166 in DBD directly determines the action of PPARγ in beige cell adipocyte differentiation. Moreover, p-T166 interrupts the interplay of LBD with cofactors in adipocytes, supporting the notion found in vitamin D receptor (VDR) that specific conformational changes in the DBD can alter the coactivator interaction surfaces in the LBD [35, 36]. As NR members are highly homologous, the DBD and its PTM patterns may have a broad effect on diverse cell types.

Promoting beige cell biogenesis is can improve obesity-related metabolic dysfunction [37, 38]. Here, p-T166 restrained beige adipocyte inducibility and inhibited lipid catabolism activity in WAT. Blockage of p-T166 restored the beigeing program impaired by HFD, combating obesity related metabolic dysfunctions. From a therapeutic standpoint, pharmacological intervention to inhibit p-T166 could be a convenient approach treating obesity. Moreover, this strategy might avoid the typical side effects caused by TZDs [39]. Actually, specific modulation of PTMs to control unique subsets of PPARγ target genes has been reported. Bruce etc. demonstrated that targeting Ser273 phosphorylation using SPPARMs specifically elicited insulin-sensitizing gene expression in adipocytes [40, 41]. In our study, p-T166 acted in a relatively specific manner to reduce the expression levels of genes involved in fatty acid oxidation, respiratory chain assembly and oxidative phosphorylation in adipocytes. Thus, the modulation of p-T166 by CDDO is a promising therapeutic regimen for treating obesity-related metabolic disorders.

Allostery is an inherent regulatory process that functions to finely adjust the transcriptional behavior of nuclear receptors (NRs) [42, 43]. For PPARγ, the binding of distinct ligands to its LBD alters the DBD recognition specificity on DNA, which elicits overlapping, but discrete, patterns of target gene expression [44, 45]. A single point mutation in PPARγ-LBD is sufficient to hinder the communication of transcriptional signals from LBD to DBD and change the DNA binding affinity [34]. This evidence indicates that communication between the LBD and DBD via allosteric interactions controls PPARγ function. Here, TD disturbed intradomain LBD-DBD communication, and p-T166 altered the interaction of PPARγ with pro-browning cofactors, which docked on LBD [46], suggesting that p-T166 was an allosteric signal to control transcriptional complex assembly. Although DBD conformation in tailoring cofactor binding and transcriptional activity in particular NRs have been demonstrated [47], a role for PTMs in DBD to determine cell-specific program has not been reported. In addition, CDDO sharply decreased H/D exchange in the peptides of the DBD neighboring the T166 site and the loop between helix 9 and helix 10 in the LBD, which has been shown to participate in the formation of the LBD-DBD interface by crystallography [34]. This effect enhanced LBD-DBD, further making the conformational shrinking of DBD and impeding the PKCα catalytic action on PPARγ. This long distal ligand-induced stability of DBD might restrict the binding activity of PPARγ and allow it to bind only limited subsets of genes (most likely beige cell-specific genes).

Protein‒protein interactions (PPIs) are commonly exploited in drug development. A number of small molecules targeting PPIs have been shown to effectively treat cancer, immunological disease and neurodegenerative disease [48, 49]. Here, CDDO disturbed the interplay between PPARγ and PKCα without affecting PKCα activity. In addition, CDDO induced allosteric communication between the LBD and DBD. These actions of CDDO inhibited PPARγ p-T166 and thus activated beige cell biogenesis. Furthermore, unlike PKCα inhibitor in adipocytes [50, 51], CDDO did not impede normal adipogenesis, which suggested that targeting the PPI of PKCα-PPARγ was a safe tactic for promoting WAT browning.

## Materials and methods

### Generation of PPARγ-T166A (TA) and PPARγ-T166D (TD) mice and animal study

The generation approach is outlined in Fig. S5a. Mice with the PPARg mutation were generated using CRISPR-Cas9 technique, as described previously [52]. Cas9 mRNA was ordered from Sigma-aldrich. For sgRNA preparation, T7 promoter sequence was appended to the sgRNA template by PCR amplification. Run the PCR products on a 2%(wt/vol) agarose gel in TAE buffer to evaluate the expected size. Conducting the gel purification of T7-sgRNA PCR product by using PCR purification kit (QIAQuick). The sgRNA was transcribed in vitro by using T7-sgRNA PCR product as the template with the MEGAshortscript™ T7 (Thermo Fisher AM1354) kit according to the kit protocol. Dilute the sgRNA to 500 ng/µl in RNase-free water and check its quality on a 2% (wt/vol) agarose gel in TAE buffer. Single-cell zygotes from superovulated 3- to 4-week-old C57BL/6 J females mice mated to C57BL/6 J males were injected with Cas9 mRNA, sgRNA and ssDNA, and cultured overnight. Transfer the two-cell embryos into oviducts of pseudopregnant Swiss Webster surrogate females. The offspring pups were genotyped for the TA or TD mutations by identified by PCR and DNA sequencing. The genotyping forward primer: CAAGACATGGTCTCCCTCTG. The genotyping reverse primer: AGTTGTCCTAGTGCCTACTG. The DNA sequencing primer: CTGGCCTACACGAAGAAGCTC. F1 offspring carrying the mutant allele were crossed with C57BL/6 J mice from Gempharmtech Co., Ltd. F2 heterozygotes were crossed with heterozygotes to generate homozygotes and WT littermates, the latter of which were used as controls.

Mice used in this study were kept on a 12-h light–dark cycle and fed standard chow. To generate the diet-induced obesity (DIO) model, 3−4-week-old mice were fed a high-fat diet (HFD) (D12492, 60% fat, 20% carbohydrate, 20% protein, total 5.24 kcal/g; Research Diets Inc., New Brunswick, NJ) for 16 weeks. For chemical intervention, 6−8-week-old mice were treated with 5 mg/kg/day rosiglitazone (RSG) or 3 mg/kg/day CDDO (Bardoxolone). Mice were weighed daily until sacrifice under anesthesia using diethylether. Animal welfare and experimental procedures were carried out in strict compliance with the ‘Guide for the Care and Use of Laboratory Animals’ (Ministry of Science and Technology of China) and the related ethical regulations of Nanjing University. Animal sample sizes were determined based on previous studies and literatures of the field using similar experimental paradigms.

### Human samples

We obtain subcutaneous adipose samples from 3 female individuals (BMI = 21.1, 25.5 and 27.0) undergoing the liposuction surgery at Nanjing drum tower hospital, the affiliated hospital of Nanjing university medical school. None of the individuals had any previous history of cardiovascular disease, diabetes, systemic illness, chronic inflammatory disease. Patients did not follow any special diet before the surgery. SVF cells were isolated according to the standard purification protocol.

### Chemicals and antibodies

The following chemicals were used in the present study: CDDO (APExBIO, A3220), rosiglitazone (MedChemExpress, HY-17386), isoprenaline hydrochloride (MedChemExpress, HY-B0468), CDDO-Im (MedChemExpress, HY-15725), CDDO-Me (Bardoxolone methyl, PubChem CID: 400769), and SO1989 [27] (kindly provided by Prof. Sun Hongbin, China pharmaceutical University).

The following commercial antibodies were used in the present study: rabbit anti-PPARγ (#81B8, Cell Signaling Technology), mouse anti-PPARγ (sc-7273, Santa Cruz), anti-UCP1 (ab209483, Abcam), anti-PRDM16 (ab106410, Abcam), anti-HA (A01244, Genscript), anti-Flag (20543-1-AP, Proteintech), anti-p-PKCα (ab32502, Abcam), anti-PKCα (#2056, Cell Signaling Technology), anti-PPARγ p-Ser273 (bs-4888R, Bioss), anti-PPARγ p-Ser112 (ab195925, Abcam), anti-p-ERK1/2 (ab223500, Abcam), anti-ERK1/2 (ab17942, Abcam), anti-CDK5R1 (bs-11611R, Bioss), anti-CDK5 (bs-0559R, Bioss), anti-p-histone H1 (05-1324, Millipore), anti-β-actin (A00702, Genscript), and anti-GAPDH (#3683, Cell Signaling Technology).

For detection of p-T166 by western blotting, mouse monoclonal antibody against p-T166 was generated by immunizing mice with a KLH (keyhole limpet hemocyanin)-conjugated phosphopeptide corresponding to residues surrounding T166 of PPARγ (KLH-KGFFRRpTIRLKLIYDRC). The cell culture supernatant of the hybridoma clones were screened using ELISA and dot blotting.

### Primary adipocyte differentiation and culture

Stromal vascular fractions (SVFs) were isolated from the inguinal WAT (iWAT) and interscapular BAT. Subsequently, adipose deposits were homogenized and digested by shaking for 90 min at 37 °C in Hanks buffer (61.5 mM NaCl, 2.5 mM KCl, 0.65 mM CaCl_2_, 2.5 mM glucose, 50 mM HEPES, 50 U/mL, 50 μg/mL Pen/Strep) containing 5% (w/v) BSA and 0.5 mg/mL collagenase type I (Worthington). Digested cells were filtered through a 100-μm strainer and centrifuged for 10 min at 1000 ×g. The SVF cells were rinsed twice with PBS and seeded in DMEM/F12 supplemented with 10% FBS. After culture for 48 h, non-adherent cells were removed by washing 3 times with PBS. Adipogenic differentiation was induced when the cells reached confluence. The differentiation culture medium used was DMEM/F12 supplemented with 10% FBS, 850 nM insulin, 1 nM triiodothyronine (T3), 0.5 mM 3-isobutyl-1-methylxanthine (IBMX), 5 μM dexamethasone (Dex), and 125 nM indomethacin. Four days after incubation, the differentiation medium was replaced with maintenance DMEM/F12 medium containing 10% FBS, 850 nM insulin, and 1 nM T3. The culture medium was changed every two days. On day 8, mature adipocytes were harvested and subjected to the following assays.

### TissueGnostics imaging analysis

Image acquisition: The TissueFAXS Quantitative Imaging System (TissueGnostics, Vienna, Austria) was used to acquire images from the slides. The entire slide was scanned at low magnification using the 2.5× objective, and then the 20× objective was used to acquire high magnification images and exclude the blank area outside the tissue.

Quantitative analysis of tissue section images: Image processing and analysis was performed using StrataQuest 6.0.182 (TissueGnostics, Vienna, Austra). Image processing included reconstruction of whole images and segmentation of all the structures on the slide. After automatic color separation, the hematoxylin and eosin channels were independent of each other. All lipid droplets were identified by dense reticular formation with nuclear location but a low-stained background. In the StrataQuest software, all lipid droplets were projected onto a two-dimensional scatter plot and gating tools were used to select the boundaries of objects of a certain size. At the same time, lipid droplets were highlighted for assessment and reconfirmation.

### Lipid and mitochondrial staining

Differentiated adipocytes were stained with 100 nM MitoTracker Red (ThermoFisher, M7512) in serum-free DMEM/F12 for 15 min at 37 °C in accordance with the manufacturer’s instructions. After rinsing 5 times with PBS, adipocytes were fixed with 4% paraformaldehyde for 15 min at 37 °C and washed again twice with PBS. The lipid droplets were labeled with 2 μM BODIPY 493/503 (ThermoFisher, D3922) for 15 min. Cells were rinsed 5 times with PBS and the nuclei were stained with DAPI. Confocal microscopy was used to analyze the lipid droplets and the membrane potential of the mitochondria.

### RNA-sequencing and data analysis

For RNA-Seq, total RNA was extracted from differentiated adipocytes in vitro using TRIzol reagent (Invitrogen). Next generation sequencing library preparations were conducted according to the manufacturer’s protocol. The libraries were sequenced on a Novaseq 6000 (Illumina) and the reads were aligned to the GRCm38.97 genome (ENSEMBL) using Hisat2 (v2.0.1). Analysis of differentially expressed genes was conducted using the DEseq2 (v1.28.1) Bioconductor package. Gene Ontology (GO) and Kyoto Encyclopedia of Genes and Genomes (KEGG) enrichment analyses were performed using the ClusterProfiler (v3.16.0) R package. The module eigengene expression, adjacency matrix heatmap, module-trait relationships, and other related parameters were calculated and visualized according to package instructions.

### Hydrogen deuterium exchange mass spectrometry (HDX-MS)

Differentiation HDX experiments were conducted as previously reported [36, 53]. Briefly, 20 μM full-length mouse PPARγ2 (apoprotein) was mixed with a 1:10 molar excess of ligands and incubated for 2 h at 5 °C. After complex formation, the apo- or liganded protein was mixed with reaction buffer (20 mM Na_3_PO_4_, 500 mM NaCl, pH 7.6) containing D_2_O and incubated at 5 °C for 0, 10, 30, 60, 300, 900, 1200, and 3600 s. The reaction system was quenched with buffer containing 4 M guanidine hydrochloride, 100 mM citric acid, and 0.2 M TCEP, pH 2.5 at 0.5 °C. Immediately, proteins were passed through a pepsin column (2.1 mm × 30 mm, ThermoFisher), and the digested peptides were desalted on an Acclaim PepMap300 C18 column (1.0 mm × 15 mm, (ThermoFisher). Peptides were separated on an ACQUITY UPLC Peptide CSH C18 column (1 mm × 50 mm, Waters). Digestion and separation were conducted at 0.5°C. Mass spectrometry data were acquired using a high-resolution mass spectrometer (Orbitrap Fusion, ThermoFisher) over a mass range of 300−1500 *m/z* for 15 min. Three replicates were obtained for each HDX experiment at each time point, and three non-deuterated FIP200 protein samples were used for data analysis of tandem MS/MS experiments. The BioPharm Finder (version 2.0, ThermoFisher) was used to identify the peptides, and the sorted peptides (scoreå 80) were analyzed using the HD-Examiner software (Sierra Analytics). The MS/MS data were analyzed by BioPharm Finder (version 2.0) to identify the peptides. The peptides (Scoreå 80) were further analyzed by HD-Examiner, and each peptide (both deuterated and non-deuterated) was manually confirmed with respect to the correct retention time, *m/z* range, presence of overlapping peptide envelopes, and charge state. The separate evaluation of deuterium uptake variability from the three replicates defined a significance threshold of ±5% difference in HDX for a single time point. Comparisons between the apoprotein and apoprotein−ligand complex were plotted using HD-Examiner.

### Oxygen consumption assays

Cells were differentiated and treated as previously described [54]. The oxygen consumption rate (OCR) was obtained using the XF24 Extracellular Flux Analyzer (Seahorse Biosciences). In this experiment, the OCR was calculated from five independent measurements. Baseline OCR was measured every 7 min and following the sequential injection of oligomycin (1 μM), carbonyl cyanide-4-(trifluoromethoxy) phenylhydrazone (FCCP) (1 μM), and antimycin A (2 μM).

### Histological and immunohistochemical analysis

Tissues were fixed in 10% formaldehyde for at least 24 h and embedded in paraffin. Sections (5-μm) were cut and stained with hematoxylin and eosin (H&E) or specifically labeled for immunohistochemistry (IHC). Images were visualized and photographed using a Nikon A1R confocal laser scanning microscope. Cell size was analyzed using the TissueGnostics imaging system or the Image J software.

### Quantitative real-time PCR

Total RNA was isolated from adipose tissue or adipocytes using TRIzol reagent (ThermoFisher), and cDNA was synthesized using a 5× All-In-One RT MasterMix (abm, G486) kit in accordance with the manufacturer’s instructions. RT-qPCR was performed on a StepOnePlus (Applied Biosystems) using AceQ qPCR SYBR Green Master Mix (Vazyme, Q111-02). The expression of the measured genes was normalized to the 36B4 mRNA expression level. The sequences of the primers used for qPCR are shown in Table S1.

### Molecular docking

PPARγ (PDB:3e00) and PKCα (PDB:3iw4) were docked using the ClusPro and Zdock servers. The top overlapping predictions between the two servers were further modeled by Pymol. As shown in Fig S3d, the PPARγ DBD is colored purple, the LBD is colored green, and PKCα is colored red. T166 is marked in blue and the predominant catalytic elements in PKCα are marked in yellow.

### In vitro kinase assay

Human PPARγ was purchased from Cayman Chemical (Item No. 61700) and active human PKCα was purchased from Millipore (14−484). An in vitro kinase assay was conducted in the 1× kinase buffer (Cell Signaling Technology, 9820 S) supplemented with 1 mM CaCl_2_, PKC lipid activator (Millipore, 20−133), and 10 mM ATP (Cell Signaling Technology, 9804 S). In each reaction tube, 200 ng PPARγ was incubated with 10, 50, or 100 ng PKCα at 30°C for 2 h. At the same time, one tube without PKCα was maintained on ice as a negative control. Histone H1 (Millipore, 14−155) was used as a positive control. The reaction products were subjected to western blotting and MS/MS phosphorylation pattern identification.

### Glucose tolerance (GTT) and insulin tolerance (ITT) tests

For the GTT, mice were fasted for 12 h, following which glucose (2 g/kg) was intraperitoneally injected. For the ITT, mice were fasted for 12 h and then injected intraperitoneally with insulin (0.6 U/kg). Blood was collected from the tail vein at the indicated times (0, 15, 30, 60, 90, and 120 min) after injection of either glucose (GTT) or insulin (ITT). Blood glucose levels were measured using a glucometer.

### PPARγ gene−luciferase reporter assay

Genes encoding WT, TA, and TD mouse PPARγ2 were cloned separately into the pcDNA3.1(-) vector. pIRES-hPPARγ/PPRE-Luc and pRL-control plasmids were purchased from Addgene. HEK293T cells were transfected with PPARγ2, PPRE-Luc, and pRL at a molar ratio of 50:50:1. After a 6-h transfection period, the culture medium was replaced with fresh DMEM containing 10% FBS. Small molecules were added to the culture wells and incubated for 24 h. Cells were collected and lysed in luciferase lysis buffer, and the luciferase activity of the cells was measured using the dual-luciferase reporter assay system (Promega, E1910).

### Phosphorylation pattern identification

The products of the in vitro kinase assay were digested with Lys-C (Promega, V167A). The resulting peptides were dissolved in 0.2% formic acid (FA) and separated using an online Nano-LC system (Microtech Scientific) equipped with a C18 reversed-phase column. A linear gradient of 5−30% acetonitrile in 0.2% FA was used for column elution. The MS data were acquired using an LTQ-Orbitrap mass spectrometer (ThermoFisher). Full scan spectra (*m/z* 300–1600) were acquired with a resolution of 60,000 at 400 *m/z* after the accumulation of 1,000,000 ions. The five most-intense ions per scan were selected for collision-induced dissociation fragmentation in the linear ion trap after the accumulation of 3000 ions. The maximal filling times were set at 500 ms for full scans and 150 ms for MS/MS scans. The dynamic exclusion list was restricted to 500 entries, with a maximum retention period of 60 s and a relative mass window of 10 ppm. Raw files were analyzed using the MaxQuant software. Peak list files were searched against the UniProt protein sequence database.

The search parameters were set as follows: enzyme, trypsin; up to two missed cleavages; carbamidomethyl cysteine as a fixed modification; and methionine oxidation and protein N-terminal acetylation as variable modifications. The MS tolerance was 6 ppm, while the MS/MS tolerance was 0.5 Da. The required false discovery rate was set to 1% at the peptide and protein levels, and the minimum acquired peptide length was seven amino acids. At least one unique or razor peptide per protein group was required for protein identification.

### Co-immunoprecipitation (Co-IP)

Mouse PKCα, PRDM16, and PGC1α genes were cloned into the pcDNA3.1(-) vector. HEK293T cells were co-transfected with these genes and PPARγ2. After 24 h, the cells were lysed in Western blotting and IP lysis buffer (Cell Signaling Technology, 9803 S). Total protein (500 μg − 1 mg) was pre-cleared with 1 μg rabbit IgG (Cell Signaling Technology, #3900) and 20 μL Protein A + G agarose (Abcam, ab193262) for 3 h at 4 °C. Total protein was isolated by centrifugation and 0.5 μg anti-Flag antibody was added. Following an overnight incubation at 4 °C, 30 μL Protein A + G agarose was used to purify the immune complex, following which the agarose was washed 7 times in wash buffer (150 mM NaCl, 1 mM EDTA, 20 mM Tris, 1% Triton X-100, pH 8.0). Protein was eluted in 1× SDS-PAGE loading buffer at 100 °C for 10 min. For Co-IP assays in primary adipocytes, mature adipocytes differentiated in vitro were collected and lysed, and 2 μg mouse anti-PPARγ antibody (Santa Cruz, sc-7273) was used for IP.

### ChIP assay

Adipose tissue was ground into small pieces, cross-linked in 0.5% paraformaldehyde (PFA) for 5 min at room temperature, and quenched with 125 mM glycine for 10 min. The tissue was subsequently washed three times with PBS prior to homogenization. A Dounce tissue homogenizer was used to isolate the nucleus, and the remaining tissue was homogenized in NPB buffer (25 mM HEPES, 1.5 mM MgCl_2_, 10 mM KCl, 1% NP-40, 1 mM DTT, 1 mM PMSF, protease inhibitor cocktail, pH 7.8). Homogenates were filtered through a 100-μm strainer. Nuclei were resuspended in sonication buffer (50 mM HEPES, 140 mM NaCl, 1 mM EDTA, 1% Triton X-100, 0.1% Na-deoxycholate, 0.5% SDS, 0.5 mM PMSF, protease inhibitor cocktail, pH 7.9), incubated on ice for 20 min, and sonicated to generate 200−500-bp fragments. After sonication, 10 μg sheared chromatin was diluted in ChIP dilution buffer (16.7 mM Tris, 1.2 mM EDTA, 167 mM NaCl, 1.1% Triton X-100, and 0.01% SDS, pH 8.0) and incubated overnight at 4 °C with a rabbit anti-PPARγ monoclonal antibody (Cell Signaling Technology, #81B8). A 1% chromatin solution was used as the input control. The following day, ChIP-grade Protein G magnetic beads (Cell Signaling Technology, #9006) were added to the immunoprecipitates and incubated for 2 h. The beads were washed twice each with low-salt buffer (150 mM NaCl, 20 mM Tris-HCl, 2 mM EDTA, 1% Triton-X, 0.1% SDS, pH 8.0), high-salt buffer (500 mM NaCl, 20 mM Tris-HCl, 2 mM EDTA, 1% Triton-X; 0.1% SDS, pH 8.0), LiCl buffer (0.25 M LiCl, 10 mM Tris-HCl, 1 mM EDTA, 1% deoxycholate, pH 8.0), and TE buffer (10 mM Tris, 1 mM EDTA, pH 8.0), and the proteins were eluted in ChIP elution buffer (50 mM Tris, 1 mM EDTA, 1% SDS, 50 mM NaHCO_3_, pH 8.0). The eluate was de-crosslinked, digested with proteinase K, and subjected to DNA purification. RT-qPCR was subsequently performed using the primers shown in Table S1.

### Statistical analysis

Statistical analysis was performed using the GraphPad Prism 8.0 software. Error bars indicate the standard error of the mean (S.E.M.) unless otherwise indicated. The unpaired Student’s *t*-test was used for two-group comparisons, and the one-way ANOVA followed by Tukey’s test were used for multiple-group comparisons. The two-way ANOVA followed by Bonferroni’s test was used for the GTT, ITT, seahorse test, and body weight and rectal temperature measurement. ^*^P < 0.05, ^**^P < 0.01, ^***^P < 0.001.

### Reporting summary

Further information on experimental design is available in the Nature Research Reporting Summary linked to this paper.

## Supplementary information

Supplemental Figures and Legends

Supplemental dataset 1

Supplemental dataset 2

Supplemental dataset 3

Table S1. Primers in this study

Table S2

Uncropped Western blotting data

Reporting Summary checklist

## Data Availability

RNA-seq data have been deposited in GEO under accession number GSE158615. All source data presented in this study are available from the corresponding author upon request.

## References

[1] Kusminski CM, Bickel PE, Scherer PE (2016). Targeting adipose tissue in the treatment of obesity-associated diabetes. Nat Rev Drug Disco.

[2] Rosen ED, Spiegelman BM (2014). What we talk about when we talk about fat. Cell.

[3] Cildir G, Akıncılar SC, Tergaonkar V (2013). Chronic adipose tissue inflammation: all immune cells on the stage. Trends Mol Med.

[4] Kotas ME, Medzhitov R (2015). Homeostasis, inflammation, and disease susceptibility. Cell.

[5] Nathan C, Ding A (2010). Nonresolving inflammation. Cell.

[6] Harms M, Seale P (2013). Brown and beige fat: development, function and therapeutic potential. Nat Med.

[7] Kim K-H, Kim YH, Son JE, Lee JH, Kim S, Choe MS (2017). Intermittent fasting promotes adipose thermogenesis and metabolic homeostasis via VEGF-mediated alternative activation of macrophage. Cell Res.

[8] Wang W, Seale P (2016). Control of brown and beige fat development. Nat Rev Mol Cell Biol.

[9] Bostrom P, Wu J, Jedrychowski MP, Korde A, Ye L, Lo JC (2012). A PGC1-alpha-dependent myokine that drives brown-fat-like development of white fat and thermogenesis. Nature.

[10] Wu J, Bostrom P, Sparks LM, Ye L, Choi JH, Giang AH (2012). Beige adipocytes are a distinct type of thermogenic fat cell in mouse and human. Cell.

[11] Symonds ME, Pope M, Budge H (2015). The ontogeny of brown adipose tissue. Annu Rev Nutr.

[12] Bartelt A, Heeren J (2014). Adipose tissue browning and metabolic health. Nat Rev Endocrinol.

[13] He W, Barak Y, Hevener A, Olson P, Liao D, Le J (2003). Adipose-specific peroxisome proliferator-activated receptor gamma knockout causes insulin resistance in fat and liver but not in muscle. Proc Natl Acad Sci USA.

[14] Wang F, Mullican SE, DiSpirito JR, Peed LC, Lazar MA (2013). Lipoatrophy and severe metabolic disturbance in mice with fat-specific deletion of PPARgamma. Proc Natl Acad Sci USA.

[15] Jonker JW, Suh JM, Atkins AR, Ahmadian M, Li P, Whyte J (2012). A PPARgamma-FGF1 axis is required for adaptive adipose remodelling and metabolic homeostasis. Nature.

[16] Hu E, Kim JB, Sarraf P, Spiegelman BM (1996). Inhibition of adipogenesis through MAP kinase-mediated phosphorylation of PPARgamma. Science.

[17] Choi JH, Banks AS, Estall JL, Kajimura S, Boström P, Laznik D (2010). Anti-diabetic drugs inhibit obesity-linked phosphorylation of PPARγ by Cdk5. Nature.

[18] Qiang L, Wang L, Kon N, Zhao W, Lee S, Zhang Y (2012). Brown remodeling of white adipose tissue by SirT1-dependent deacetylation of Ppargamma. Cell.

[19] Katafuchi T, Holland WL, Kollipara RK, Kittler R, Mangelsdorf DJ, Kliewer SA (2018). PPARgamma-K107 SUMOylation regulates insulin sensitivity but not adiposity in mice. Proc Natl Acad Sci USA.

[20] Inagaki T, Sakai J, Kajimura S (2016). Transcriptional and epigenetic control of brown and beige adipose cell fate and function. Nat Rev Mol Cell Biol.

[21] Ohno H, Shinoda K, Spiegelman BM, Kajimura S (2012). PPARgamma agonists induce a white-to-brown fat conversion through stabilization of PRDM16 protein. Cell Metab.

[22] Loft A, Forss I, Siersbæk MS, Schmidt SF, Larsen AS, Madsen JG (2015). Browning of human adipocytes requires KLF11 and reprogramming of PPARγ superenhancers. Genes Dev.

[23] Ahmadian M, Suh JM, Hah N, Liddle C, Atkins AR, Downes M (2013). PPARgamma signaling and metabolism: the good, the bad and the future. Nat Med.

[24] Gross B, Pawlak M, Lefebvre P, Staels B (2017). PPARs in obesity-induced T2DM, dyslipidaemia and NAFLD. Nat Rev Endocrinol.

[25] Wang Y, Porter WW, Suh N, Honda T, Gribble GW, Leesnitzer LM (2000). A synthetic triterpenoid, 2-cyano-3,12-dioxooleana-1,9-dien-28-oic acid (CDDO), is a ligand for the peroxisome proliferator-activated receptor gamma. Mol Endocrinol.

[26] Lapillonne H, Konopleva M, Tsao T, Gold D, McQueen T, Sutherland RL (2003). Activation of peroxisome proliferator-activated receptor gamma by a novel synthetic triterpenoid 2-cyano-3,12-dioxooleana-1,9-dien-28-oic acid induces growth arrest and apoptosis in breast cancer cells. Cancer Res.

[27] Yang N, Tang Q, Qin W, Li Z, Wang D, Zhang W (2019). Treatment of obesity-related inflammation with a novel synthetic pentacyclic oleanane triterpenoids via modulation of macrophage polarization. EBioMedicine.

[28] Broekema MF, Savage DB, Monajemi H, Kalkhoven E (2019). Gene-gene and gene-environment interactions in lipodystrophy: lessons learned from natural PPARγ mutants. Biochimica et Biophysica Acta (BBA) - Mol Cell Biol Lipids.

[29] Hernandez-Quiles M, Broekema MF, Kalkhoven E (2021). PPARgamma in metabolism, immunity, and cancer: unified and diverse mechanisms of action. Front Endocrinol (Lausanne).

[30] Santos GM, Neves Fde A, Amato AA (2015). Thermogenesis in white adipose tissue: an unfinished story about PPARgamma. Biochim Biophys Acta.

[31] Yu XX, Lewin DA, Forrest W, Adams SH (2002). Cold elicits the simultaneous induction of fatty acid synthesis and β-oxidation in murine brown adipose tissue: prediction from differential gene expression and confirmation in vivo. FASEB J.

[32] Lodhi Irfan J, Yin L, Jensen-Urstad Anne PL, Funai K, Coleman T, Baird John H (2012). Inhibiting adipose tissue lipogenesis reprograms thermogenesis and PPARγ activation to decrease diet-induced obesity. Cell Metab.

[33] Wang H, Liu L, Lin JZ, Aprahamian TR, Farmer SR (2016). Browning of white adipose tissue with roscovitine induces a distinct population of UCP1(+) adipocytes. Cell Metab.

[34] Chandra V, Huang P, Hamuro Y, Raghuram S, Wang Y, Burris TP (2008). Structure of the intact PPAR-gamma-RXR- nuclear receptor complex on DNA. Nature.

[35] Zhang J, Chalmers MJ, Stayrook KR, Burris LL, Wang Y, Busby SA (2011). DNA binding alters coactivator interaction surfaces of the intact VDR-RXR complex. Nat Struct Mol Biol.

[36] Zheng J, Chang MR, Stites RE, Wang Y, Bruning JB, Pascal BD (2017). HDX reveals the conformational dynamics of DNA sequence specific VDR co-activator interactions. Nat Commun.

[37] Kajimura S, Spiegelman BM, Seale P (2015). Brown and beige fat: physiological roles beyond heat generation. Cell Metab.

[38] Chen Y, Ikeda K, Yoneshiro T, Scaramozza A, Tajima K, Wang Q (2019). Thermal stress induces glycolytic beige fat formation via a myogenic state. Nature.

[39] Marciano DP, Chang MR, Corzo CA, Goswami D, Lam VQ, Pascal BD (2014). The therapeutic potential of nuclear receptor modulators for treatment of metabolic disorders: PPARgamma, RORs, and Rev-erbs. Cell Metab.

[40] Choi JH, Banks AS, Estall JL, Kajimura S, Bostrom P, Laznik D (2010). Anti-diabetic drugs inhibit obesity-linked phosphorylation of PPARgamma by Cdk5. Nature.

[41] Choi JH, Banks AS, Kamenecka TM, Busby SA, Chalmers MJ, Kumar N (2011). Antidiabetic actions of a non-agonist PPARgamma ligand blocking Cdk5-mediated phosphorylation. Nature.

[42] Fernandez EJ (2018). Allosteric pathways in nuclear receptors - Potential targets for drug design. Pharm Ther.

[43] Meijer FA, Leijten-van de Gevel IA, de Vries R, Brunsveld L (2019). Allosteric small molecule modulators of nuclear receptors. Mol Cell Endocrinol.

[44] Camp HS, Li O, Wise SC, Hong YH, Frankowski CL, Shen X (2000). Differential activation of peroxisome proliferator-activated receptor-gamma by troglitazone and rosiglitazone. Diabetes.

[45] Sears DD, Hsiao A, Ofrecio JM, Chapman J, He W, Olefsky JM (2007). Selective modulation of promoter recruitment and transcriptional activity of PPARgamma. Biochem Biophys Res Commun.

[46] Lodhi IJ, Dean JM, He A, Park H, Tan M, Feng C (2017). PexRAP inhibits PRDM16-mediated thermogenic gene expression. Cell Rep.

[47] Meijsing SH, Pufall MA, So AY, Bates DL, Chen L, Yamamoto KR (2009). DNA binding site sequence directs glucocorticoid receptor structure and activity. Science.

[48] Zhang M, Xing CY, Liu J (2013). Study of the efficacy of mizoribine in lupus nephritis in Chinese patients. Rheumatol Int.

[49] Kawasaki Y (2009). Mizoribine: a new approach in the treatment of renal disease. Clin developmental Immunol.

[50] McGowan K, DeVente J, Carey JO, Ways DK, Pekala PH (1996). Protein kinase C isoform expression during the differentiation of 3T3-L1 preadipocytes: loss of protein kinase C-alpha isoform correlates with loss of phorbol 12-myristate 13-acetate activation of nuclear factor kappaB and acquisition of the adipocyte phenotype. J Cell Physiol.

[51] Fleming I, MacKenzie SJ, Vernon RG, Anderson NG, Houslay MD, Kilgour E (1998). Protein kinase C isoforms play differential roles in the regulation of adipocyte differentiation. Biochemical J.

[52] Yang H, Wang H, Jaenisch R (2014). Generating genetically modified mice using CRISPR/Cas-mediated genome engineering. Nat Protoc.

[53] Brooun A, Gajiwala KS, Deng YL, Liu W, Bolanos B, Bingham P (2016). Polycomb repressive complex 2 structure with inhibitor reveals a mechanism of activation and drug resistance. Nat Commun.

[54] Su S, Guntur AR, Nguyen DC, Fakory SS, Doucette CC, Leech C (2018). A renewable source of human beige adipocytes for development of therapies to treat metabolic syndrome. Cell Rep.

