# Supplementary material for: Blockage of PPARγ T166 phosphorylation enhances the inducibility of beige adipocytes and improves metabolic dysfunctions
Source: Cell Death Differ. 2022 Nov 3;30(3):766–78. doi: 10.1038/s41418-022-01077-x (PMC9984430; doi:10.1038/s41418-022-01077-x)
Supplement: Supplementary file 1 — Supplemental Figures and Legends [file 41418_2022_1077_MOESM1_ESM.pdf]

Supplemental Figures and legends

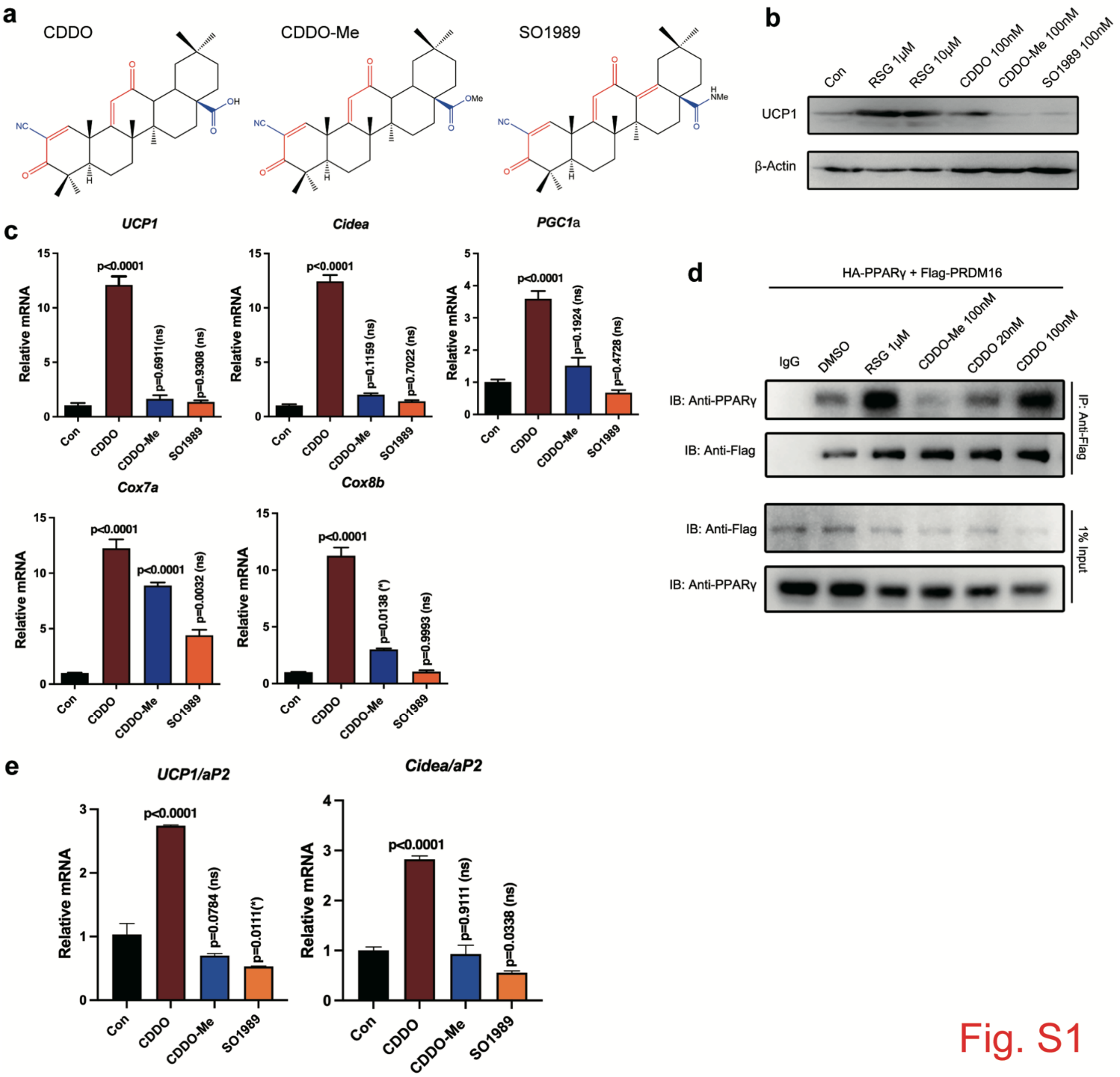

Fig. S1

Fig S1. CDDO induce beige cell differentiation *in vitro*.

(**a**) The chemical structure of CDDO, CDDO-Me and SO1989.

(**b**) SVF cells were differentiation in vitro for 5 days. Western blotting of UCP1 in SAT SVF-derived adipocytes following treatment with compounds during differentiation (RSG 1 μM or 10 μM, CDDO 100 nM, CDDO-Me 100 nM, SO1989 100 nM).

(**c**) Quantitation of the mRNA levels of browning-linked genes in SAT-derived SVF differentiated adipocytes (n = 3) (RSG 1 μM, CDDO 100nM, CDDO-Me 100nM, SO1989 100 nM).

(**d**) Co-immunoprecipitation of overexpressed PPARγ (HA tag) with PRDM16 (Flag tag) in 293T cells.

(**e**) UCP1 and Cidea mRNA levels were normalized to aP2 (adipogenesis marker gene) level.

Data are expressed as the mean ± S.E.M. Data were analyzed by one-way ANOVA followed by Tukey's test (**c** and **e**). $^{*}P < 0.05$. $^{**}P < 0.01$, $^{***}P < 0.001$.

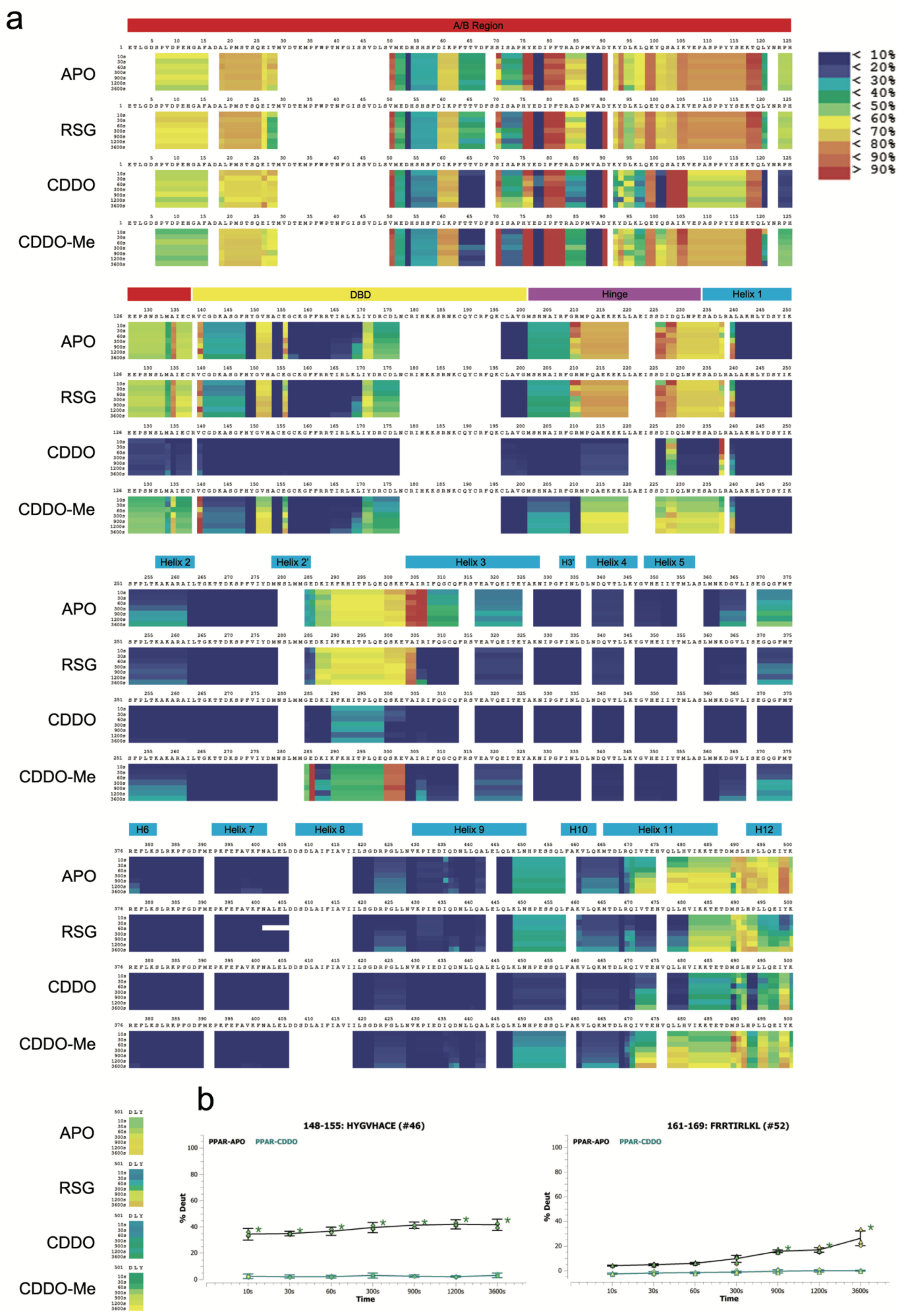

Fig S2. The dynamic features of PPARγ binding with small molecules.

(a) Heatmap of H/D-exchange for full-length PPARγ. Ligand-free state (APO protein); with rosiglitazone (RSG); with CDDO; with CDDO-Me. Each peptide fragment is presented as one horizontal colored bar. The percentage deuteration levels of the peptides at seven time points (10, 30, 60, 300, 900, 1200, and 3600 s) are shown according to the color gradient key.

(b) Deuterium incorporation kinetic curves of peptides 148-155 and 161-176.

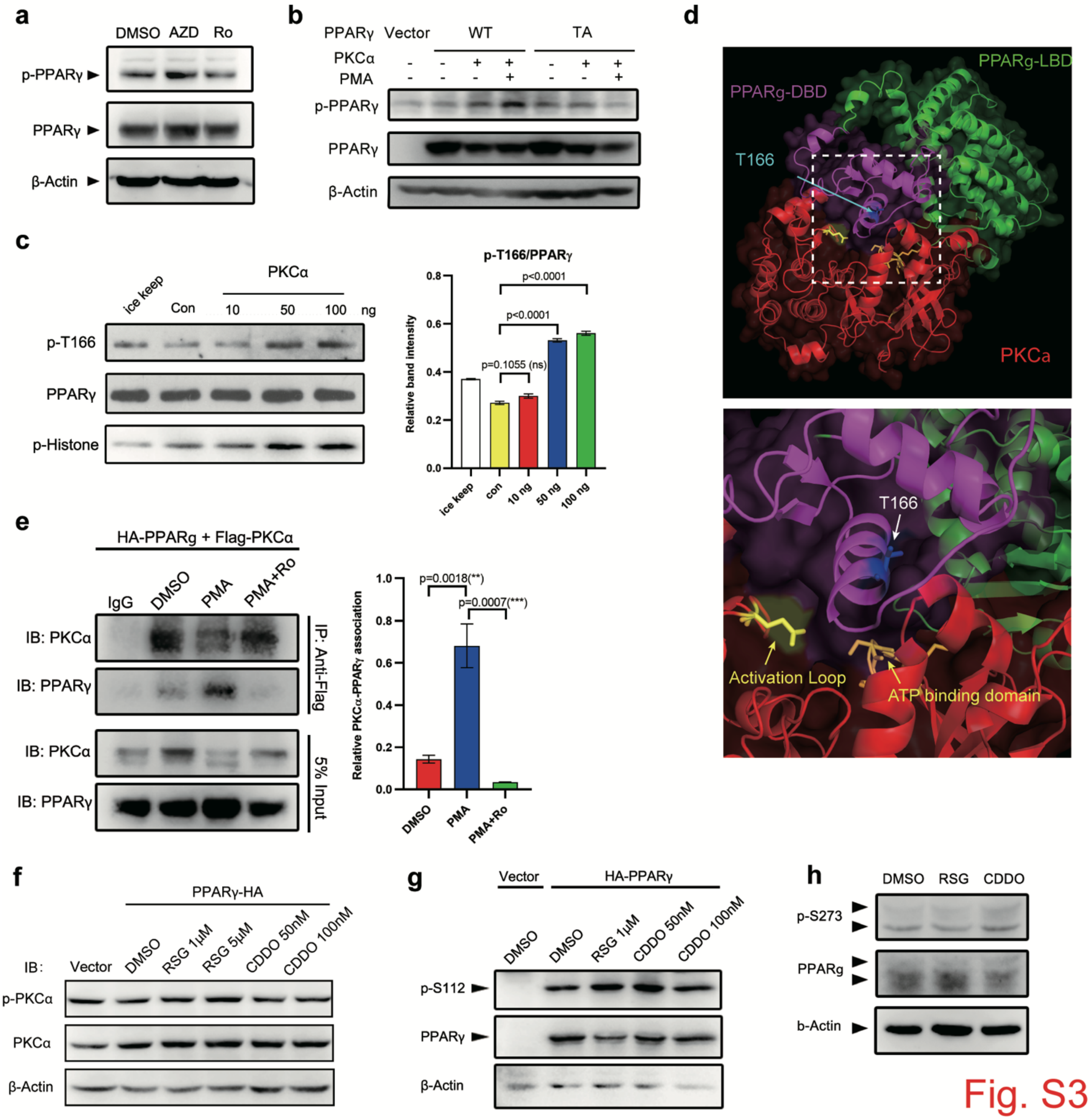

Fig. S3

Fig S3. PKCα phosphorylates PPARγ at T166.

(**a**) PPARγ-overexpressing HEK293T cells were treated with 100 nM AZD1152 (AZD) or 100 nM Ro31-8220 (Ro) overnight and PPARγ phosphorylation at T166 was analyzed by western blotting.

(**b**) Detection of PPARγ phosphorylation at T166 in MEF cells co-expressing PKCα and either WT or TA mutant PPARγ.

(**c**) *In vitro* kinase assay conducted using a constitutively active form of PKCα (caPKCα) and PPARγ1 purified from *E. coli*. Experiments were repeated three times.

(**d**) Predicted three-dimensional interplay between PKCα and PPARγ. The PPARγ DBD (purple) docked into the catalytic pocket of PKCα (red). The docking model was generated with the ZDOCK server (http://zdock.umassmed.edu/) using PDB codes 3e00 (PPARγ) and 3iw4 (PKCα).

(**e**) Co-immunoprecipitation of PPARγ and PKCα from HEK293T cells upon treatment with PMA (100 nM) and Ro (100 nM). Experiments were repeated three times.

(**f**) PKCα-overexpressing HEK293T cells were treated with RSG or CDDO for 24 h and the levels of p-PKCα (T638 phosphorylation) and PKCα were evaluated by western blotting.

(**g**) PPARγ-overexpressing HEK293T cells were treated with RSG or CDDO for 24 h and the level of Ser112 phosphorylation was detected by western blotting.

(**h**) Primary adipocytes were treated with RSG (1 μM) and CDDO (100 nM) and the level of PPARγ phosphorylation at Ser273 was evaluated by western blotting.

Data are expressed as the mean ± S.E.M. Data were analyzed by one-way ANOVA followed by Tukey's test (**c, e**). $^{*}P < 0.05$. $^{**}P < 0.01$, $^{***}P < 0.001$.

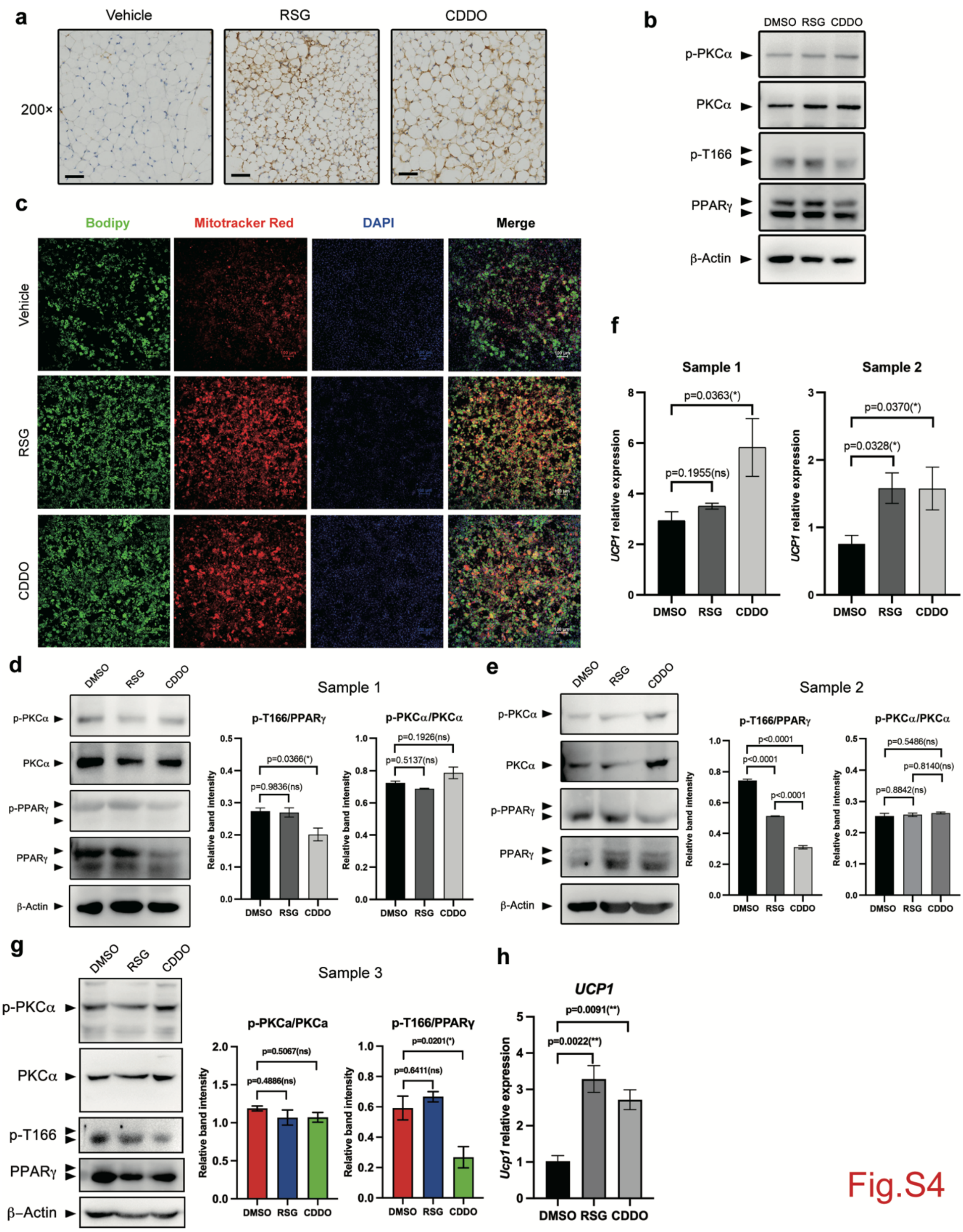

Fig S4. CDDO induced the beige phenotype in both mouse and human adipocytes.

(a) UCP1 immunohistochemical staining of SAT (n=6). Related to Figure 2.

(b) Differentiated mature adipocytes were treated with RSG (100 nM) and CDDO (100 nM) and the levels of p-T166 and PKCα activation were measured by western blotting.

(c) Confocal microscopy analysis of the mitochondrial potential (MitoTracker red) and lipid droplets (Biodipy) in SAT-derived SVF-differentiated adipocytes. 100× magnification; scale bar, 100 μm.

(d)-(f) Two human female subcutaneous adipose samples from liposuction. The SVF cells were isolated and differentiated into mature adipocytes. The small molecule compounds (100nM RSG or 100nM CDDO) were kept incubated during the differentiation process. The mature adipocytes were collected and applied to western blotting and Q-PCR analysis. p-T166 level and PKCα activation in sample 1 **(d)**. p-T166 level and PKCα activation in sample 2 **(e)**. UCP1 mRNA expression levels **(f)**. Experiments were repeated three times.

(g)-(h) One additional BMI=27.0 human female subcutaneous adipose samples from liposuction. The SVF cells were isolated and differentiated into mature adipocytes. The small molecule compounds (100nM RSG or 100nM CDDO) were kept incubated during the differentiation process. The mature adipocytes were collected and applied to western blotting and Q-PCR analysis. p-T166 level and PKCα activation in this case **(g)**. UCP1 mRNA expression levels **(h).** Experiments were repeated three times.

Data are expressed as the mean ± S.E.M. Data were analyzed by one-way ANOVA followed by Tukey's test. $^{*}P < 0.05$. $^{**}P < 0.01$, $^{***}P < 0.001$.

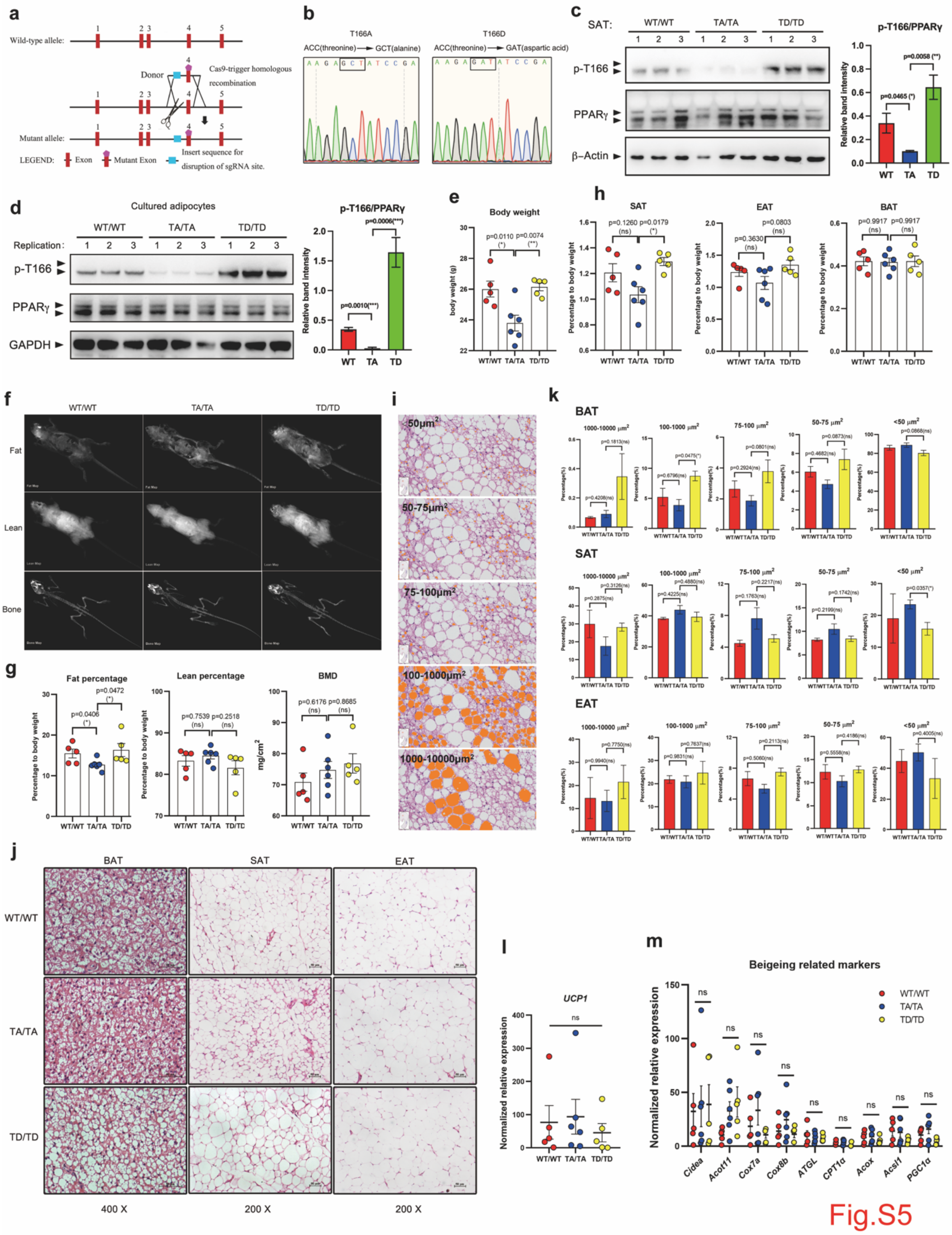

Fig S5. The metabolic phenotype of T166A/D mice.

(**a**) The generation strategy of T166D mice using CRISPR/Cas9 technology.

(**b**) DNA sequencing confirmed the replacement of the PPARγ WT allele by the T166 mutant allele. The mutated sites are highlighted.

(**c**) SAT from WT, TA and TD mice are isolated and the whole tissues protein samples are analyzed by Western blotting. Every lane contains one mouse sample.

(**d**) SVF cells from WT, TA and TD SAT are differentiated into mature adipocytes and the p-T166 level were detected by p-T166 antibody. Experiments were repeated three times.

(**e**) Body weight of homozygous WT, TA, and TD mice at 8 weeks of age.

(**f**)- (**g**) Dual-energy X-ray absorptiometry (DEXA) scans (**f**) showing lean percentage, fat percentage, and bone mineral density (BMD) (**g**) of WT and T166 mutant mice at 8 weeks old.

(**h**) Fat pad percentage quantitation by manual weighing.

(**i**) The principle of the quantitation of the size of lipid droplets by TissueFAXS Cytometry.

(**j**) H&E staining of EAT, SAT, and BAT from WT, TA, and TD mice. 400× magnification, scale bar, 50 μm; 200× magnification, scale bar, 50 μm.

(**k**) Statistical data in (**j**) by TissueFAXS Cytometry. The vertical axis represents the percentage of lipid droplets of an indicated size among the total lipid droplets.

(**l**)-(**m**) Beige cell biomarker gene expression in SATs from WT, TA, and TD mice under standard condition.

Biologically independent samples (**e-m**): WT/WT group n = 5; TA/TA group n = 6; TD/TD group = 5. Data are expressed as the mean ± S.E.M. Data were analyzed by one-way ANOVA followed by Tukey's test. $^{*}P < 0.05$. $^{**}P < 0.01$, $^{***}P < 0.001$.

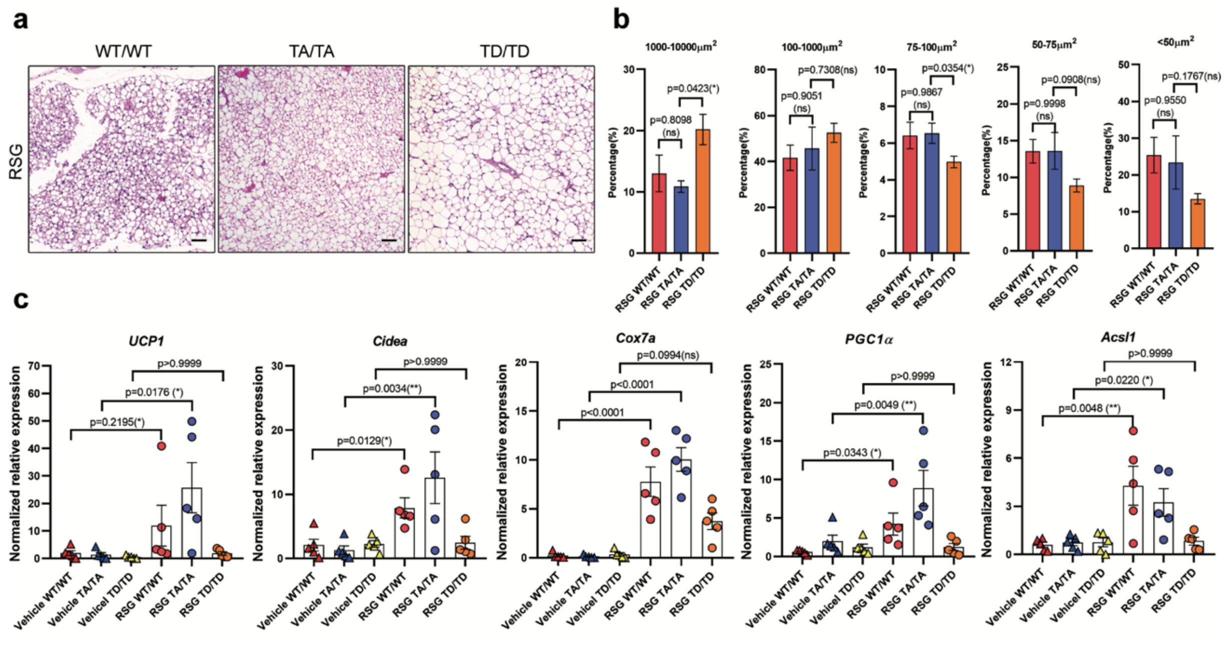

Fig. S6

Fig S6. T166 A/D mutation regulates the RSG mediated beigeing program in SAT.

(a) WT/WT, TA/TA and TD/TD mice were treated with vehicle or RSG (5 mg/kg) for 7 days. Hematoxylin and eosin (H&E) staining of SAT. 100× magnification, scale bar, 100 μm. (RSG WT/WT group n=5; RSG TA/TA group n=5; RSG TD/TD group n=5).

(b) Quantitation of the size and percentage of lipid droplets in SAT by TissueFAXS Cytometry analysis (RSG WT/WT group n=5; RSG TA/TA group n=5; RSG TD/TD group n=5).

(c) Relative mRNA levels of browning-related genes in isolated adipocytes from SAT. (Vehicle WT/WT group n = 5; Vehicle TA/TA group n=5; Vehicle TD/TD group n = 5; RSG WT/WT group n = 5; RSG TA/TA group n=5; RSG TD/TD group n = 5).

Data are expressed as the mean ± S.E.M. Data were analyzed by one-way ANOVA followed by Tukey's test (**b-c**). $^{*}P < 0.05$, $^{**}P < 0.01$, $^{***}P < 0.001$.

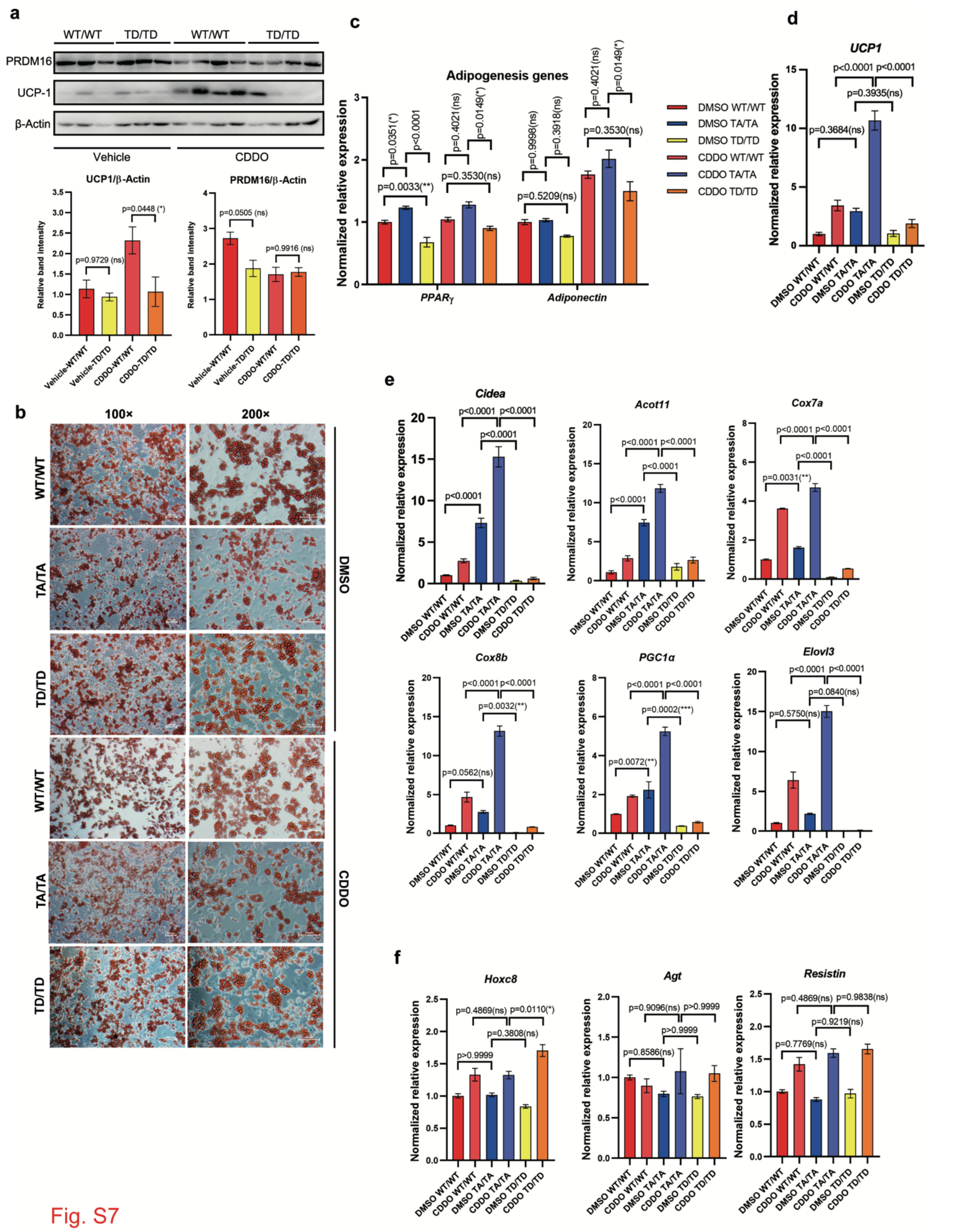

Fig S7. T166D mutation impedes CDDO-induced beige cell differentiation *in vitro*.

(**a**) Western blotting of the levels of UCP1 and PRDM16 in SAT. Each lane contains whole protein samples from two mice. β-Actin is the endogenous control. Related to Figure 3.

(**b**) Oil O red staining of SAT-derived SVF-differentiated adipocytes. 100× magnification, scale bar, 100 μm; 200× magnification, scale bar, 100 μm.

(**c**) Adipogenesis-related gene expression was evaluated by qPCR. Gene expression is normalized to the 36B4 endogenous control.

(**d**)-(**e**) Beige cell biomarkers were analyzed by qPCR. Gene expression is normalized to the 36B4 endogenous control.

(**f**) The biomarkers of white adipocytes were analyzed by qPCR. Gene expression is normalized to the 36B4 endogenous control.

Biologically independent samples: there were 3 independent biological samples in each experiment (n = 3). Data are expressed as the mean ± S.E.M. Data were analyzed by one-way ANOVA followed by Tukey's test. $^{*}P < 0.05$. $^{**}P < 0.01$, $^{***}P < 0.001$.

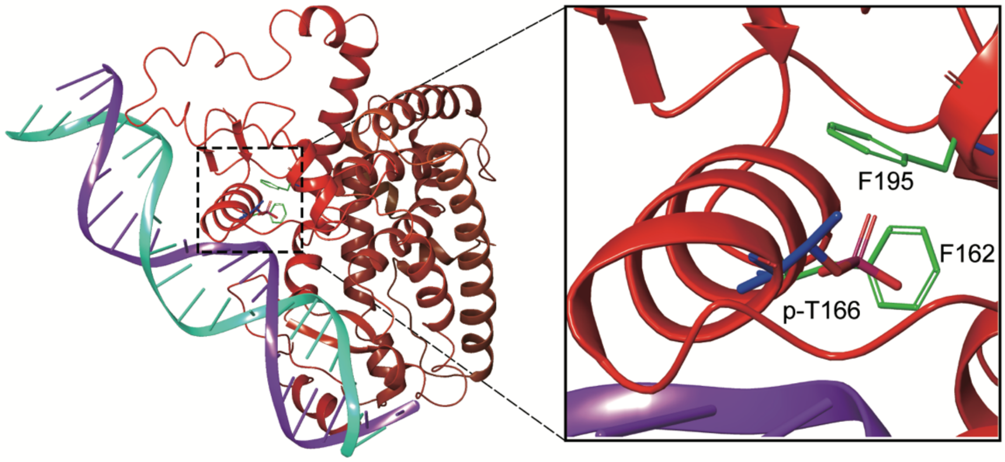

Fig. S8

Fig S8. T166 phosphorylation causes steric clash between T166 and F162 or F195 in DNA-binding domain.

Structural mimicking of T166 phosphorylation in the DBD using crystal data (PDB:3e00).

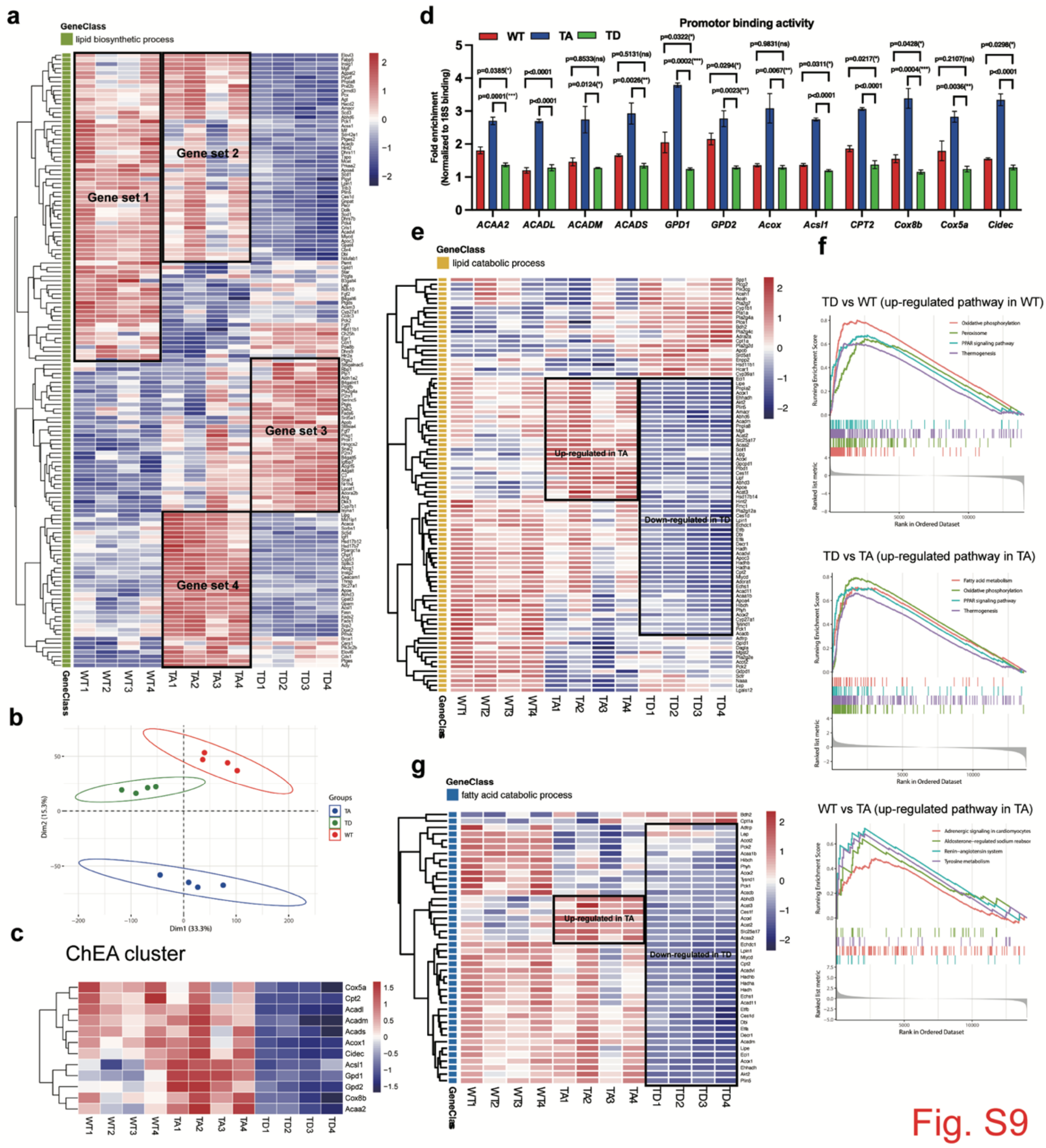

Fig S9. T166A/D mutation reprogram the lipid catabolism in adipocytes.

(**a**) Heat map of differentially expressed genes related to the lipid biosynthetic process (n = 4).

(**b**) Principal component (PC) analysis of differentially expressed genes in the transcriptome (n = 4).

(**c**) ChEA cluster of the p-T166 mediated PPARγ targeted downstream fatty acid oxidation genes (n = 4).

(**d**) ChIP-qPCR analysis of PPARγ binding to the promoters of fatty acid oxidation genes in (**c**) (n=3). Data are expressed as the mean ± S.E.M. Data were analyzed by one-way ANOVA followed by Tukey's test. $^{*}P < 0.05$. $^{**}P < 0.01$, $^{***}P < 0.001$.

(**e**) Heat map of differentially expressed genes related to lipid catabolic genes (n = 4).

(**f**) GSEA analysis of differentially expressed genes.

(**g**) Heat map of differentially expressed genes related fatty acid catabolic process (n = 4).

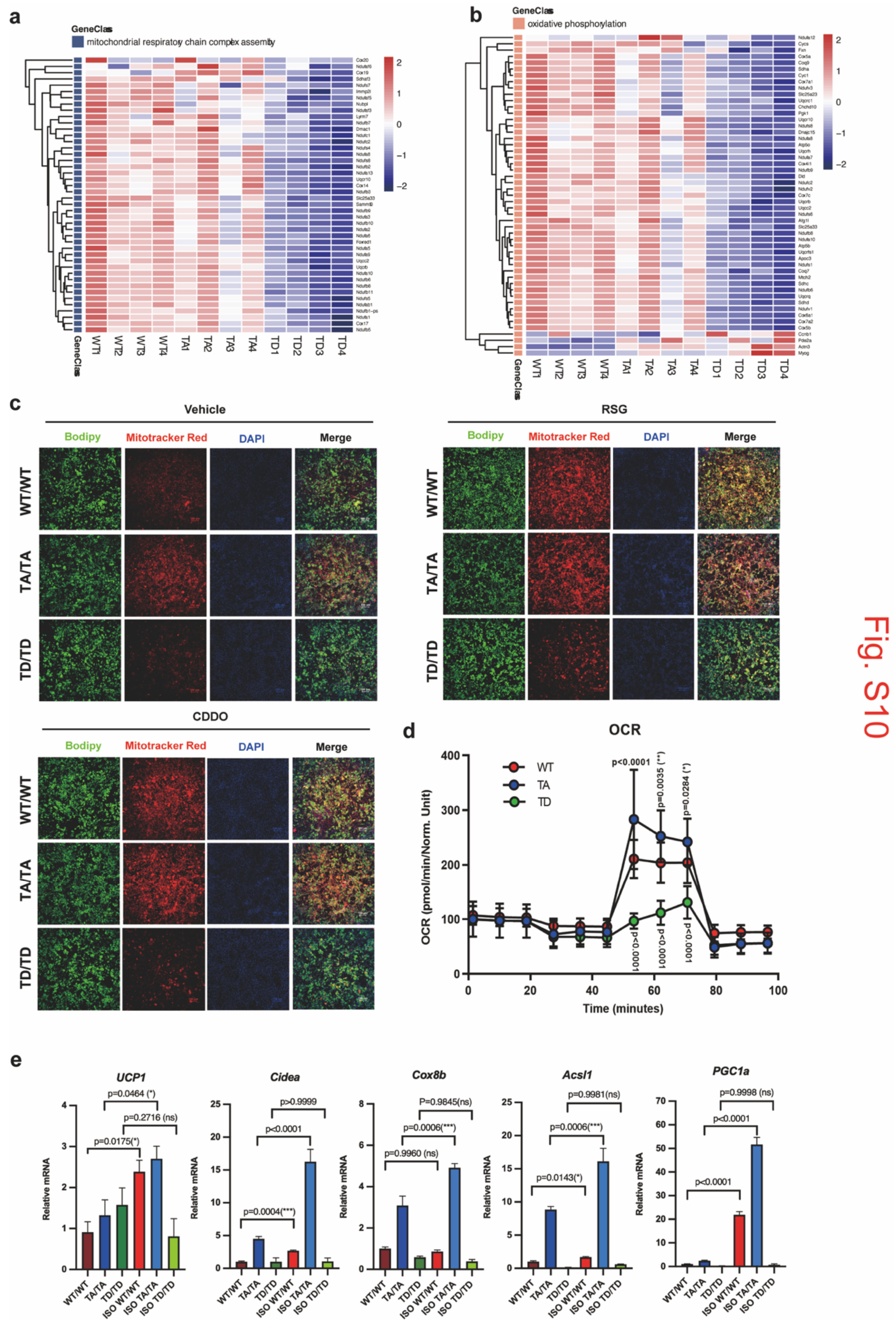

Fig. S10

Fig S10. T166 phosphorylation impedes mitochondrial activity in adipocytes.

(**a**)-(**b**) Heat map of differentially expressed genes related to mitochondrial respiratory chain complex assembly (**a**) or oxidative phosphorylation (**b**) in WT/WT, TA/TA, and TD/TD adipocytes based on RNA-Seq (n=4).

(**c**) Confocal microscopy analysis of the mitochondrial potential (MitoTracker red) and lipid droplets (Biodipy) in SAT-derived SVF-differentiated adipocytes following treatment with 100nM RSG and 100nM CDDO. 100× magnification; scale bar, 100 μm.

(**d**) Oxygen consumption rate (OCR) of cultured SAT-derived adipocyte mitochondria (n = 7).

Data were analyzed using two-way ANOVA followed by Bonferroni's test (**d**) $^{*}P < 0.05$. $^{**}P < 0.01$, $^{***}P < 0.001$.

(**e**) SVF differentiated adipocytes were treated with 2μM isoprenaline (ISO) for 12h followed by Q-PCR analysis of browning genes (n=3). Gene expression is normalized to the 36B4 endogenous control. Data are expressed as the mean ± S.E.M. Data were analyzed by one-way ANOVA followed by Tukey's test. $^{*}P < 0.05$. $^{**}P < 0.01$, $^{***}P < 0.001$.

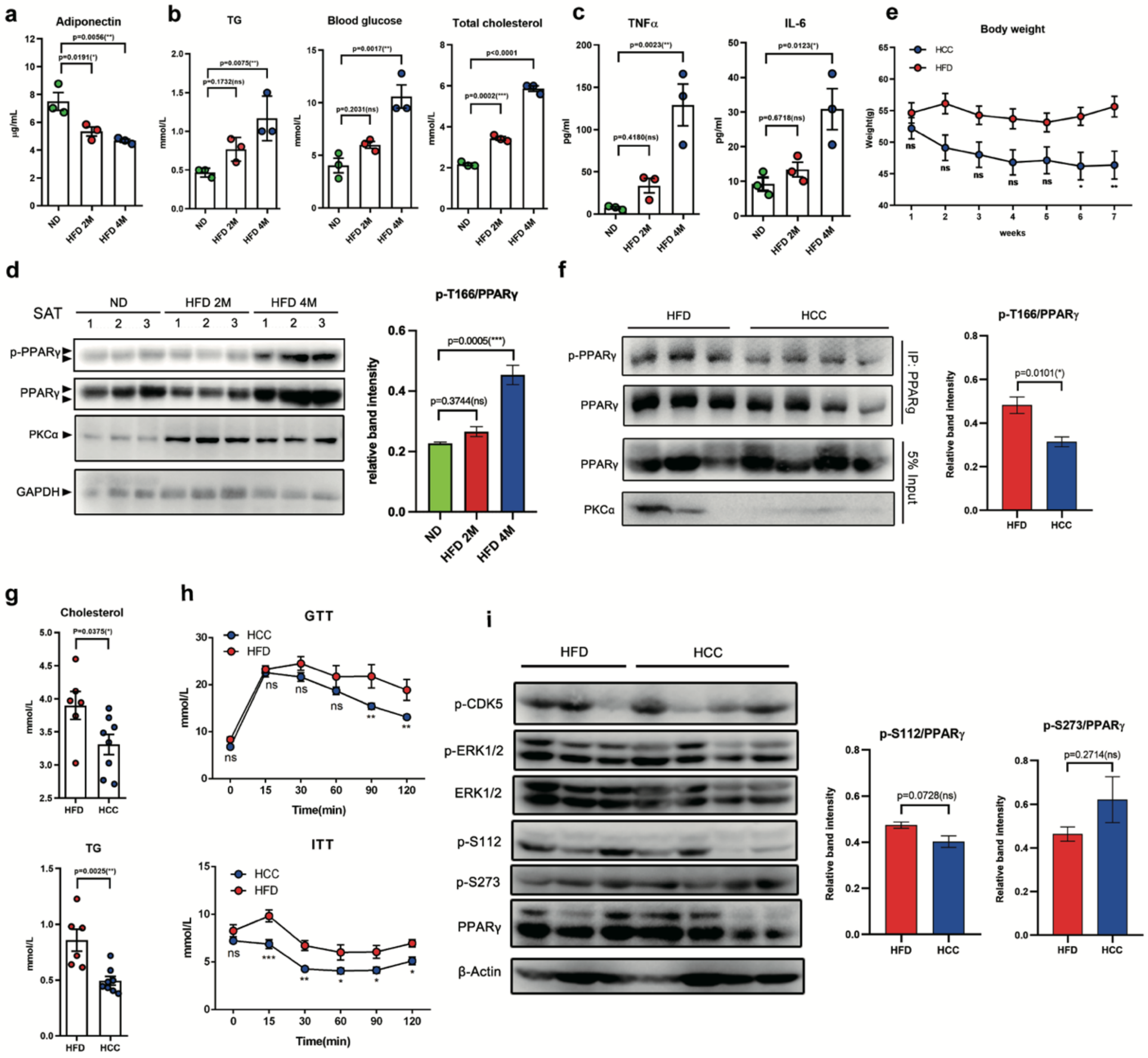

Fig S11. The level of PPARγ phosphorylation at T166 is correlated with the metabolic state.

(**a**) C57/B6J mice were fed an HFD for 2 (2M) or 4 months (4M). Quantitation of adiponectin in the serum by ELISA.

(**b**) Evaluation of lipid metabolites using commercial kits.

(**c**) Evaluation of serum inflammatory cytokines by ELISA.

(**d**) Western blotting analysis of the T166 phosphorylation level in SAT using an anti-p-T166 antibody. Every lane contains a protein sample from one mouse.

(**e**) Obese mice after a 4-month HFD were readapted to 8 weeks of chow feeding (HFD-to-chow converted, HCC). The body weight curves of HFD-treated and HCC-treated mice.

(**f**) Immunoprecipitation (IP) followed by western blotting analysis of PPARγ phosphorylation at T166. Every lane contains protein samples from two mice.

(**g**) Serum triglyceride (TG) and total cholesterol were analyzed by commercial kits.

(**h**) Glucose tolerance test (GTT) and insulin tolerance test (ITT) in HFD- and HCC-treated mice.

(**i**) Western blotting analysis of S112 and S273 phosphorylation of PPARγ. Every lane contains protein samples from two mice. Biologically independent samples: in **a**–**e**, n = 3 in each group; in **f**–**i**, HFD group n = 6, HCC group n = 8. Data are expressed as the mean ± S.E.M. Data were analyzed by two-way ANOVA followed by Bonferroni's test (**e**, **h**) or one-way ANOVA followed by Tukey's test (**a**–**c**, **d**). or Student's t-test (**f**, **g**, **i**) $^{*}P < 0.05$. $^{**}P < 0.01$, $^{***}P < 0.001$.

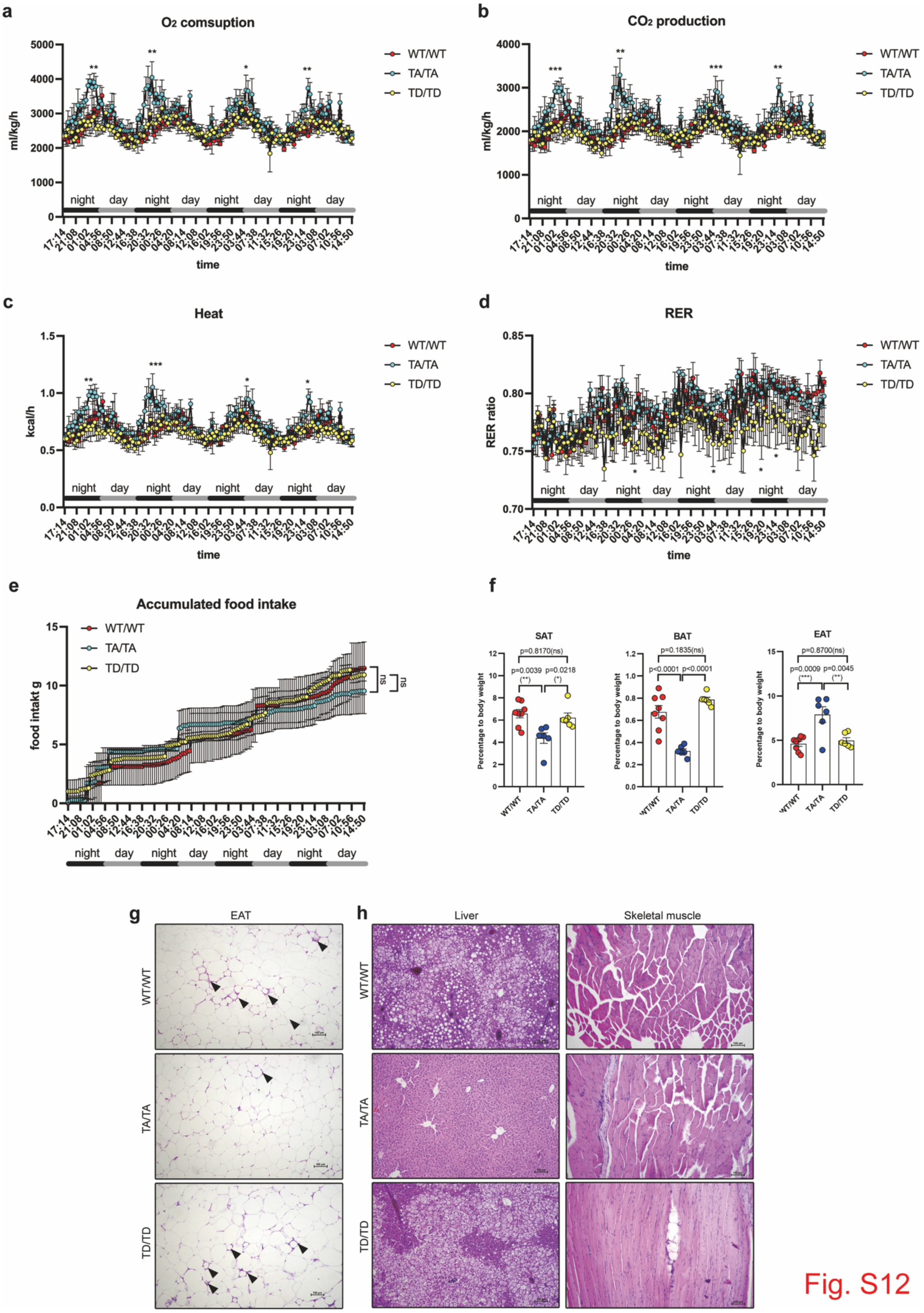

Fig S12. T166A enhanced the energy expenditure in HFD mice, and inhibited the inflammation cell infiltration in EAT, and tissue steatosis in liver and skeletal muscle. Related to Figure 6.

(a-e) Calorimetric analyses of HFD mice (WT、TA and TD) at ambient temperature (n=4). (a) Oxygen consumption; (b) Carbon dioxide production; (c) heat production; (d) RER ratio; (e) food intake on 4 constitutive days. Data are expressed as the mean ± S.E.M. Data were analyzed by two-way ANOVA followed by Bonferroni's test.

(**f**) Fat pad percentage quantitation by manual weighting.

(**g**) H&E staining of EAT. 100× magnification; scale bar, 100 μm.

(**h**) H&E staining of liver, and skeletal muscle. 100× magnification; scale bar, 100 μm. Biologically independent samples: WT/WT group n = 8; TA/TA group n = 6; TD/TD group n = 6. 100× magnification; scale bar, 100 μm. Data are expressed as the mean ± S.E.M. Data were analyzed by one-way ANOVA followed by Tukey's test . $^{*}P < 0.05$. $^{**}P < 0.01$, $^{***}P < 0.001$.
