# Supplementary material for: Blockage of PPARγ T166 phosphorylation enhances the inducibility of beige adipocytes and improves metabolic dysfunctions
Source: Cell Death Differ. 2022 Nov 3;30(3):766–78. doi: 10.1038/s41418-022-01077-x (PMC9984430; doi:10.1038/s41418-022-01077-x)

4-15: GDSPVDPEHGAF (#1)

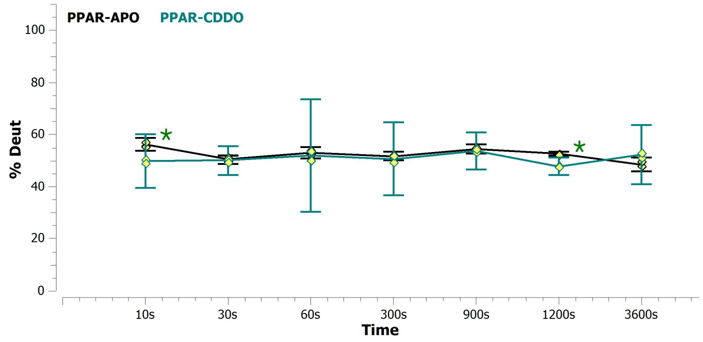

16-25: ADALPMSTSQ (#2)

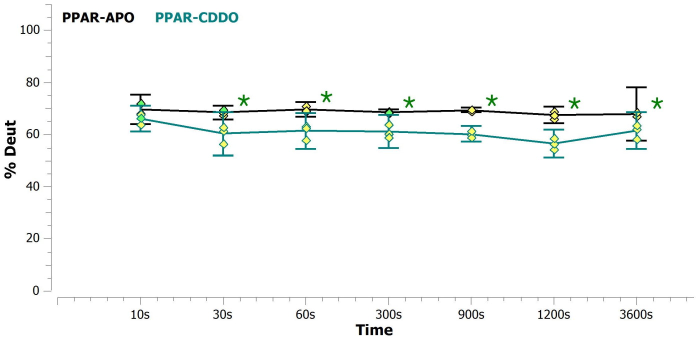

16-26: ADALPMSTSQE (#3)

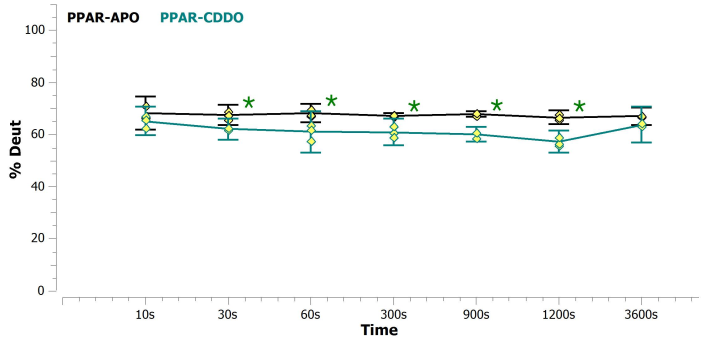

16-28: ADALPMSTSQEIT (#4)

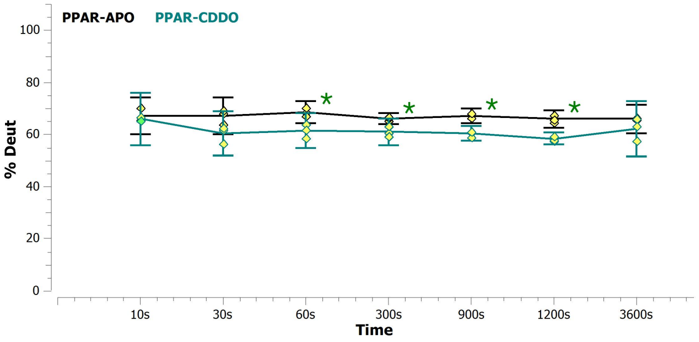

17-26: DALPMSTSQE (#5)

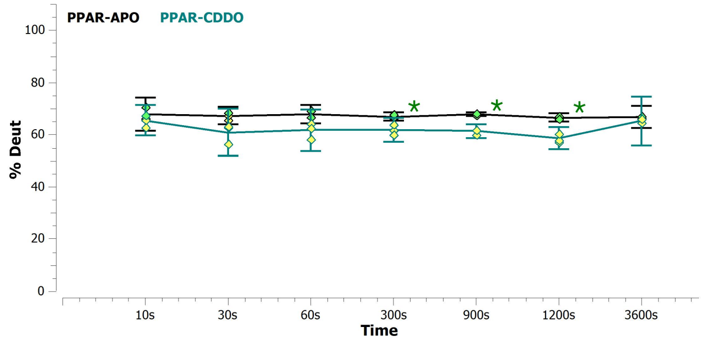

48-58: LSVMEDHSHSF (#6)

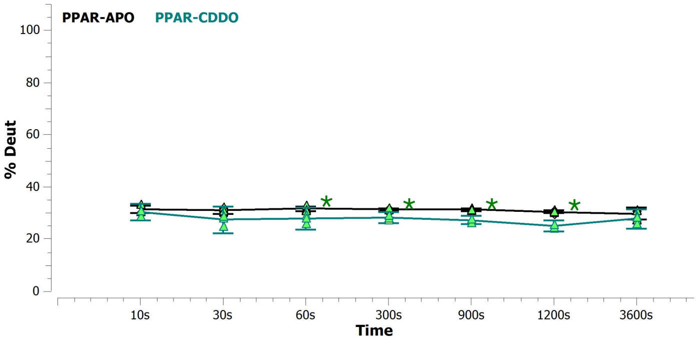

48-62: LSVMEDHSHSFDIKP (#7)

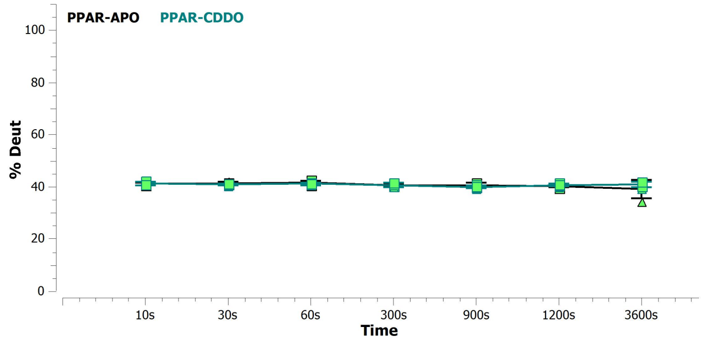

49-58: SVMEDHSHSF (#8)

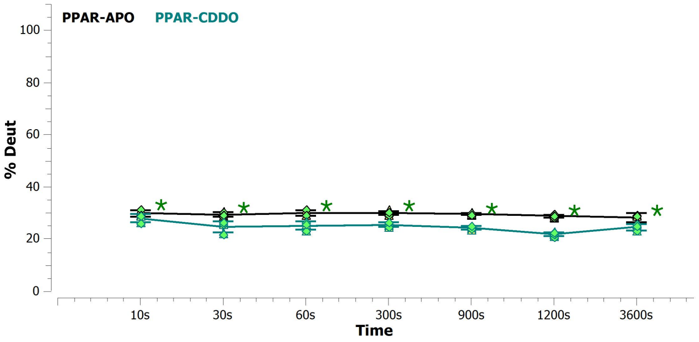

49-62: SVMEDHSHSFDIKP (#9)

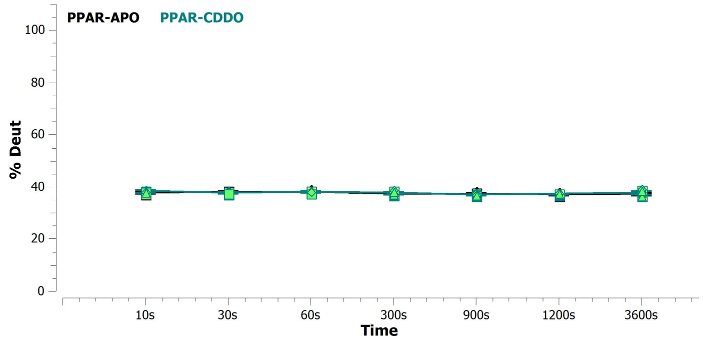

49-67: SVMEDHSHSFDIKPFTTVD (#10)

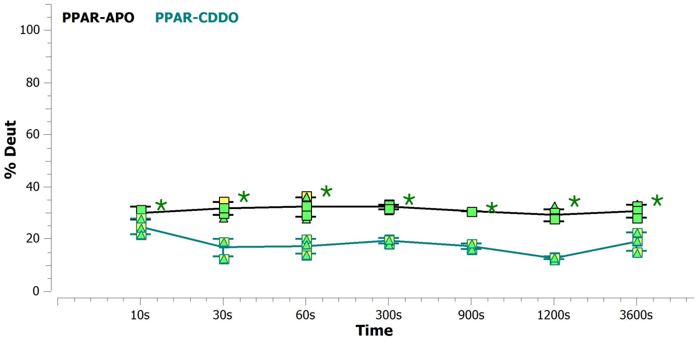

51-58: MEDHSHSF (#11)

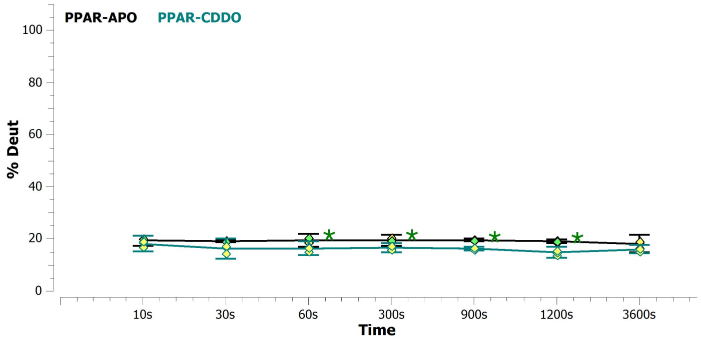

52-58: EDHSHSF (#12)

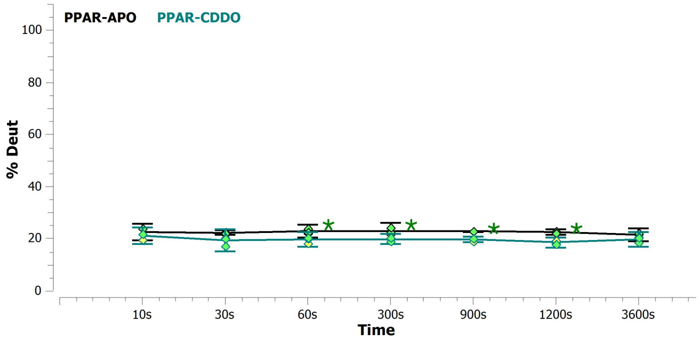

52-62: EDHSHSFDIKP (#13)

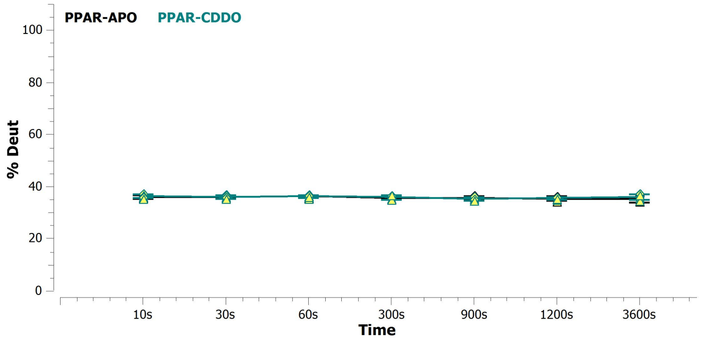

52-67: EDHSHSFDIKPFTTVD (#14)

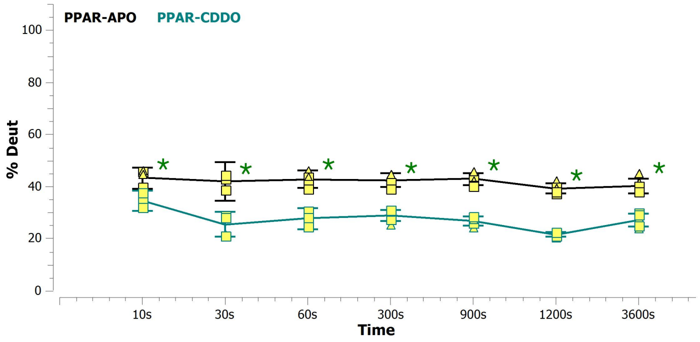

68-76: FSSISAPHY (#15)

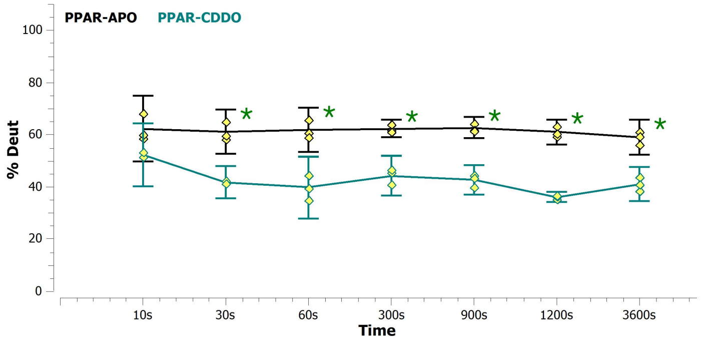

68-86: FSSISAPHYEDIPFTRADP (#16)

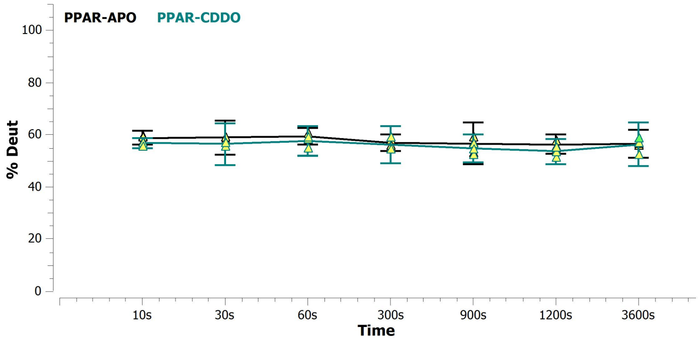

69-76: SSISAPHY (#17)

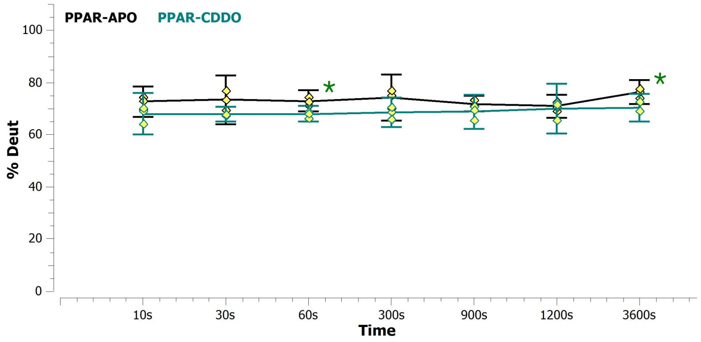

69-82: SSISAPHYEDIPFT (#18)

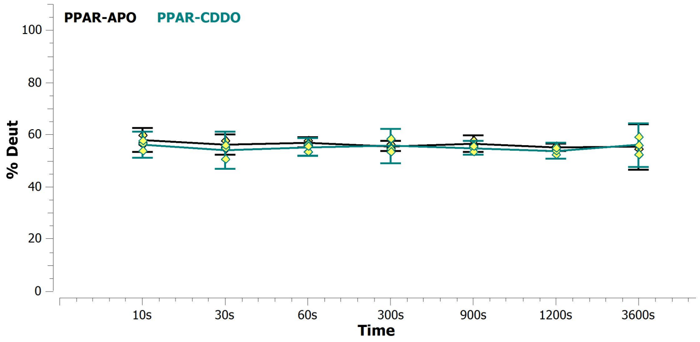

69-86: SSISAPHYEDIPFTRADP (#19)

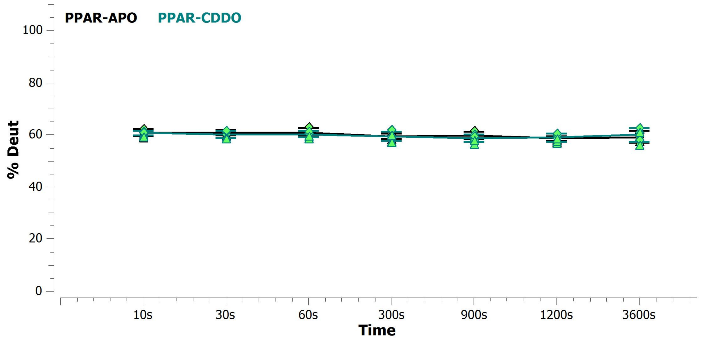

69-87: SSISAPHYEDIPFTRADPM (#20)

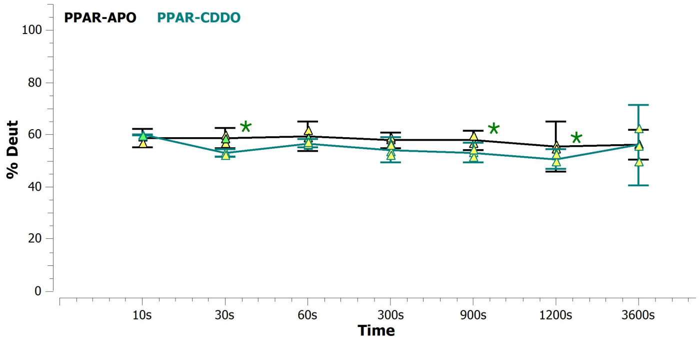

69-89: SSISAPHYEDIPFTRADPMVA (#21)

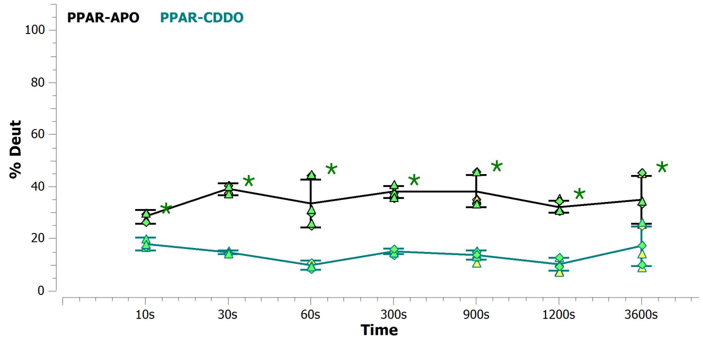

73-90: APHYEDIPFTRADPMVAD (#22)

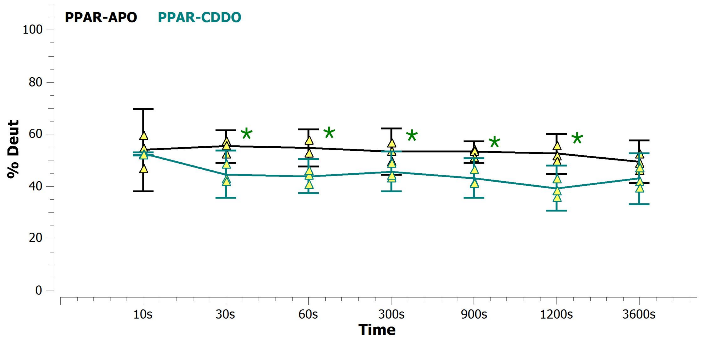

77-90: EDIPFTRADPMVAD (#23)

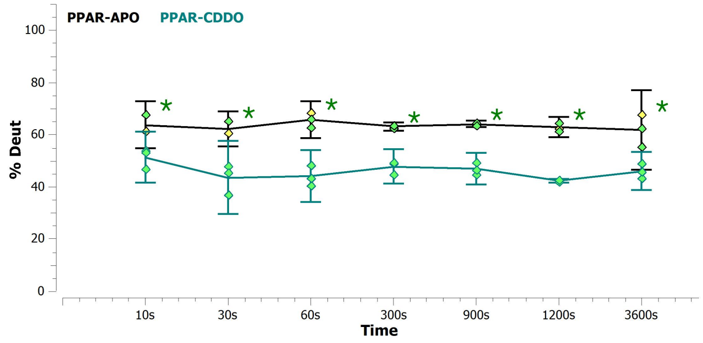

90-95: DYKYDL (#24)

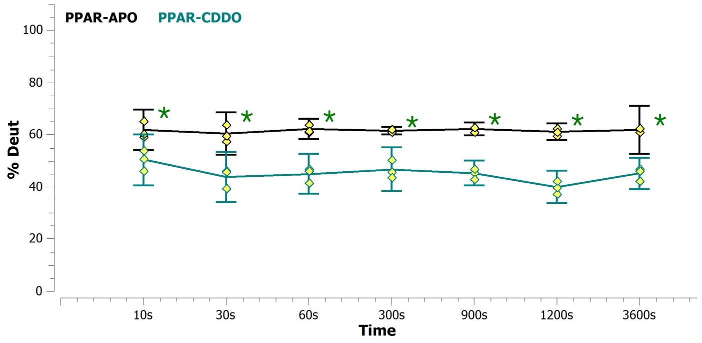

90-97: DYKYDLKL (#25)

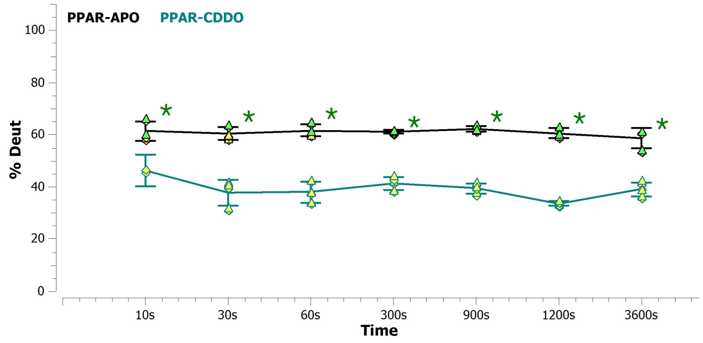

90-99: DYKYDLKLQE (#26)

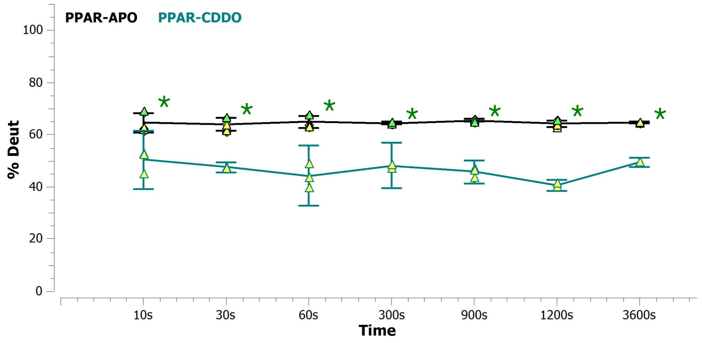

91-97: YKYDLKL (#27)

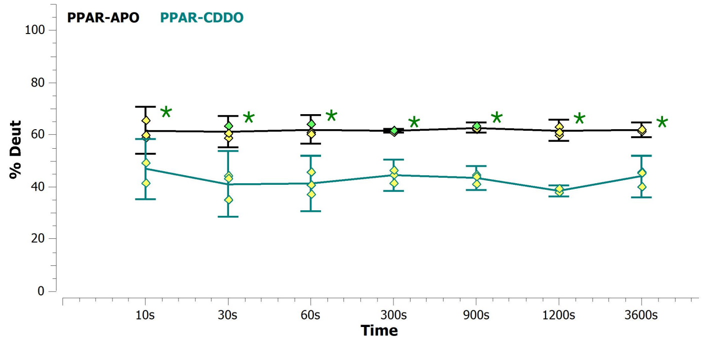

91-99: YKYDLKLQE (#28)

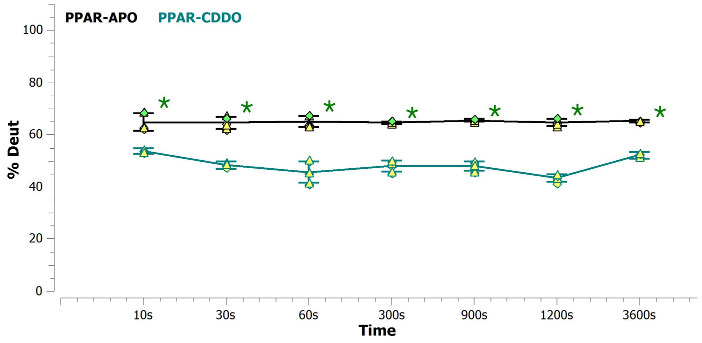

92-99: KYDLKLQE (#29)

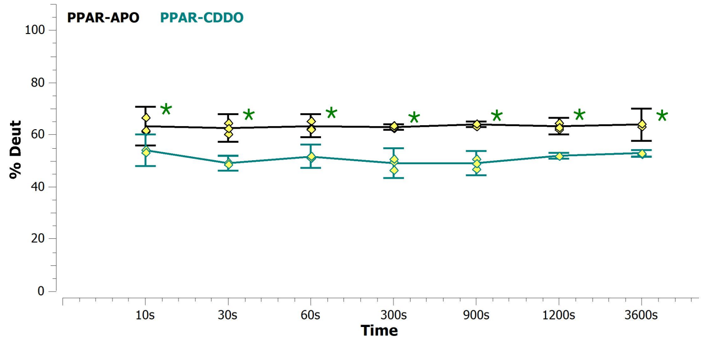

96-103: KLQEYQSA (#30)

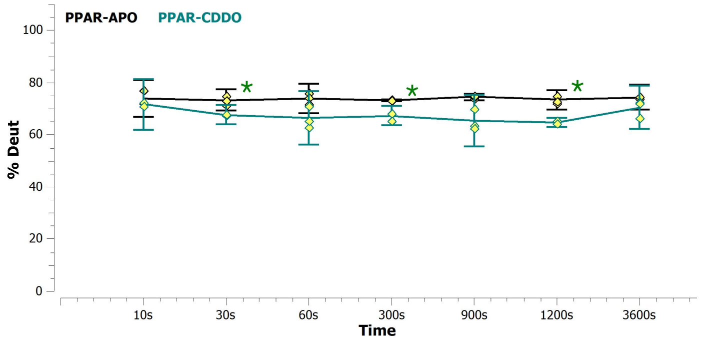

98-103: QEYQSA (#31)

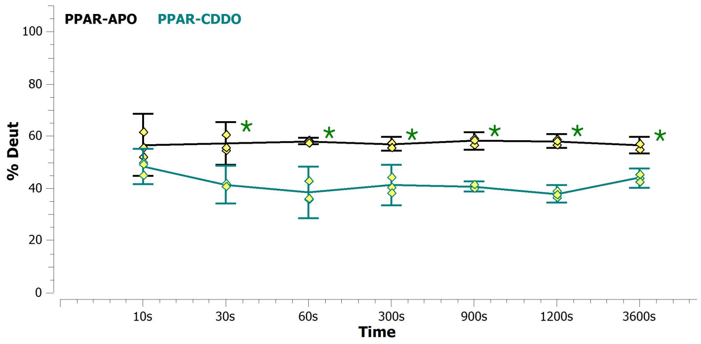

100-119: YQSAIKVEPASPPYYSEKTQ (#32)

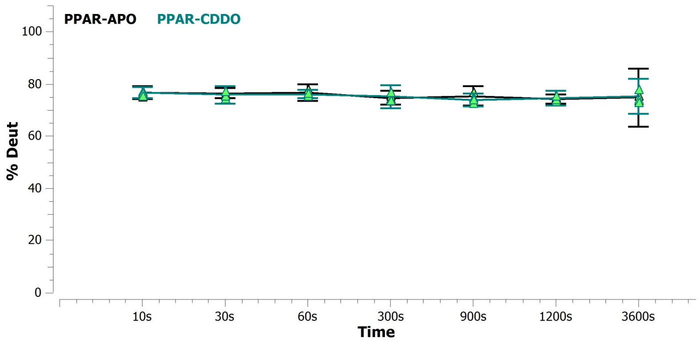

104-116: IKVEPASPPYYSE (#33)

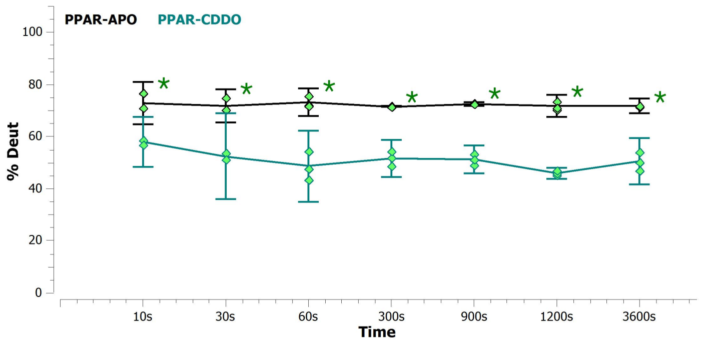

104-119: IKVEPASPPYYSEKTQ (#34)

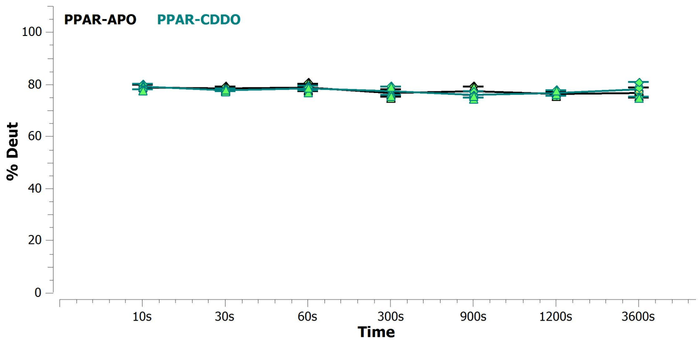

104-120: IKVEPASPPYYSEKTQL (#35)

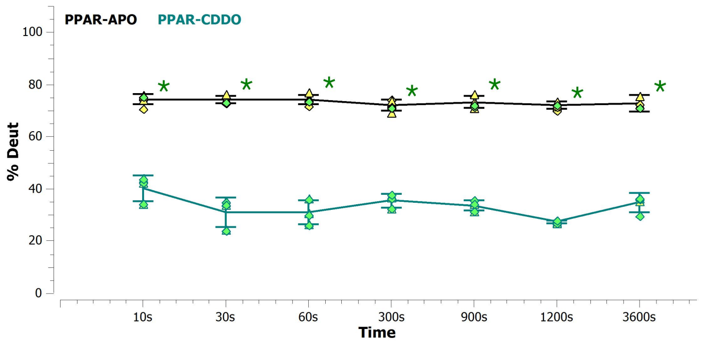

121-132: YNRPHEEPSNSL (#36)

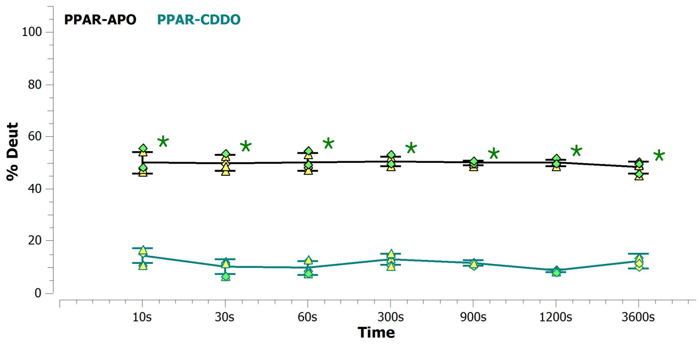

121-133: YNRPHEEPSNSLM (#37)

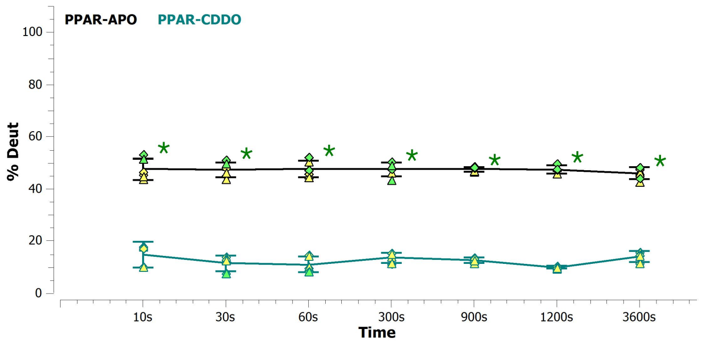

121-134: YNRPHEEPSNSLMA (#38)

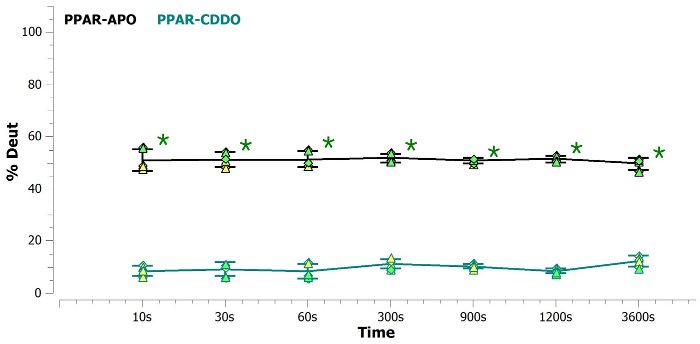

133-137: MAIEC (#39)

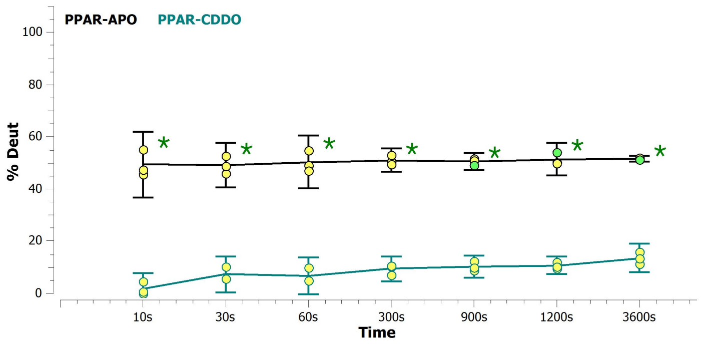

137-147: CRVCGDKASGF (#40)

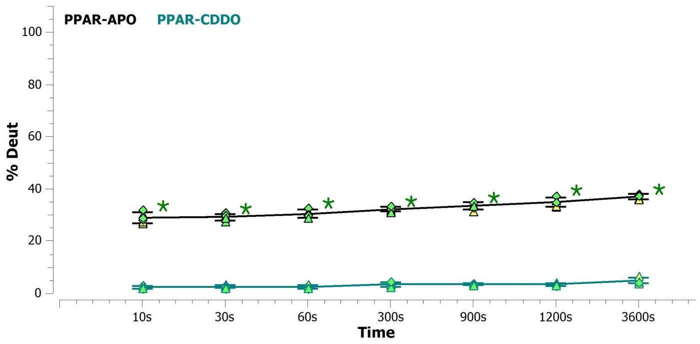

137-152: CRVCGDKASGFHYGVH (#41)

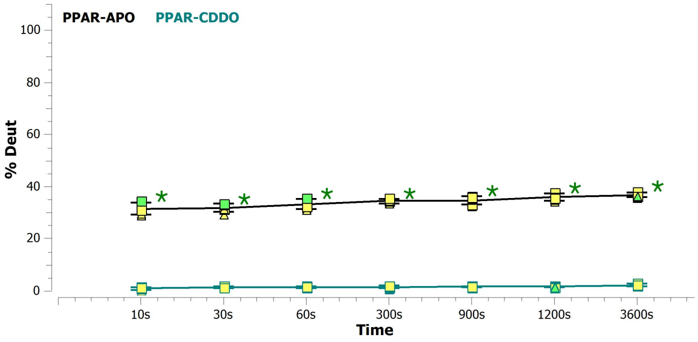

137-155: CRVCGDKASGFHYGVHACE (#42)

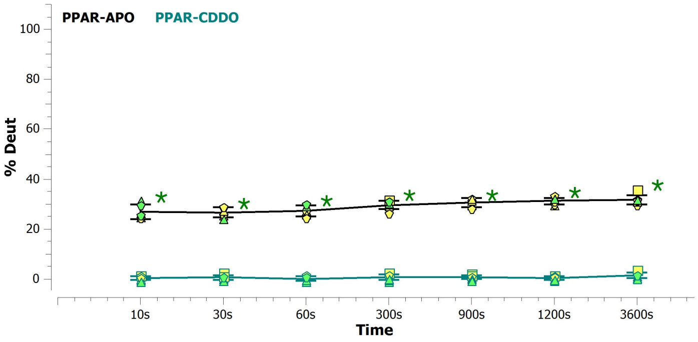

137-159: CRVCGDKASGFHYGVHACEGCKG (#43)

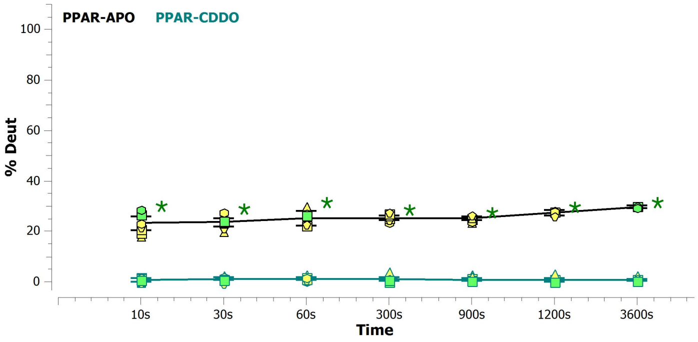

138-155: RVCGDKASGFHYGVHACE (#44)

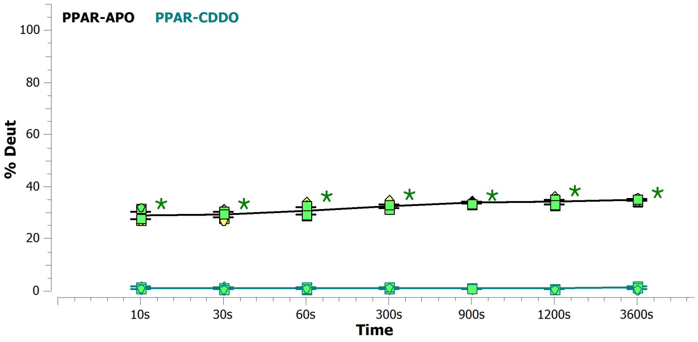

138-160: RVCGDKASGFHYGVHACEGCKGF (#45)

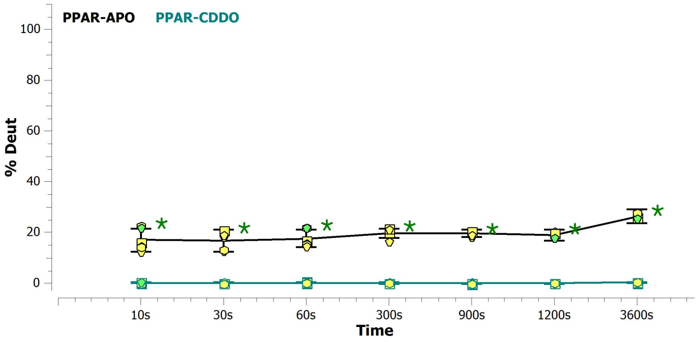

148-155: HYGVHACE (#46)

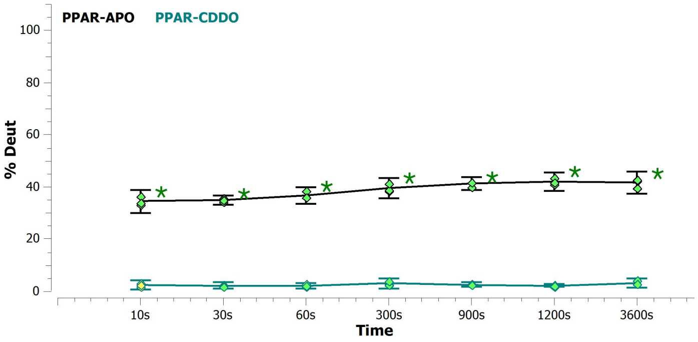

148-159: HYGVHACEGCKG (#47)

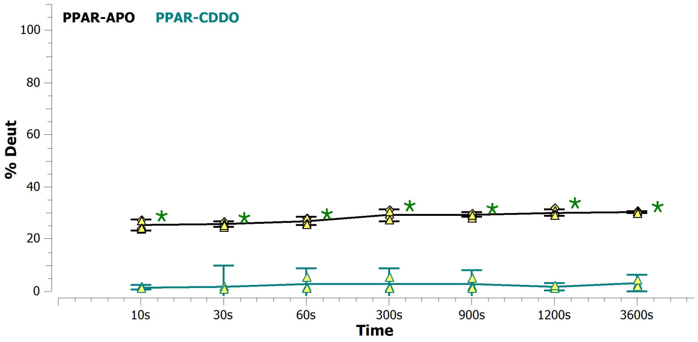

153-161: ACEGCKGFF (#48)

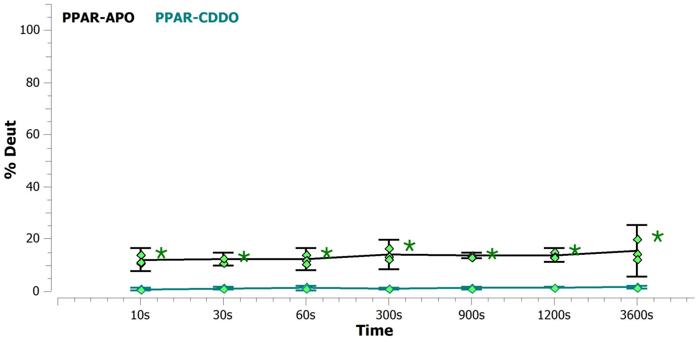

156-169: GCKGFFRRTIRLKL (#49)

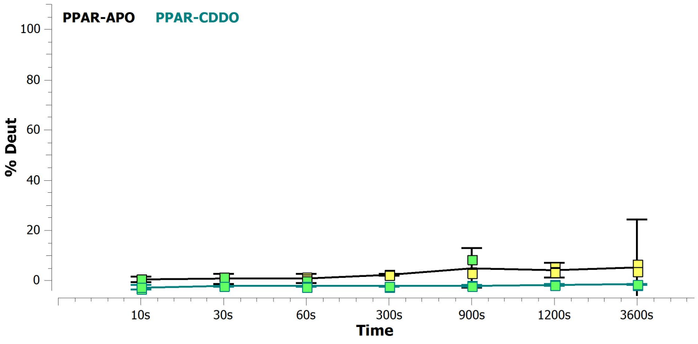

160-167: FFRRTIRL (#50)

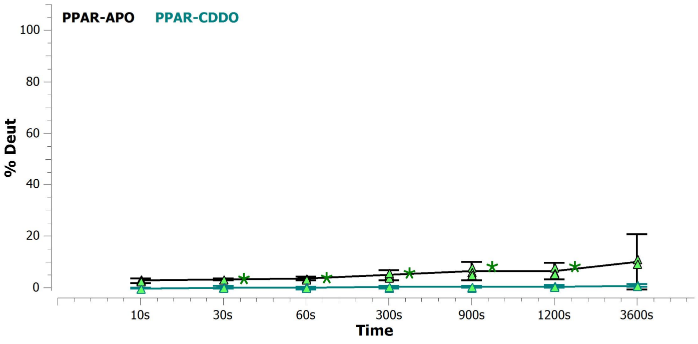

160-169: FFRRTIRLKL (#51)

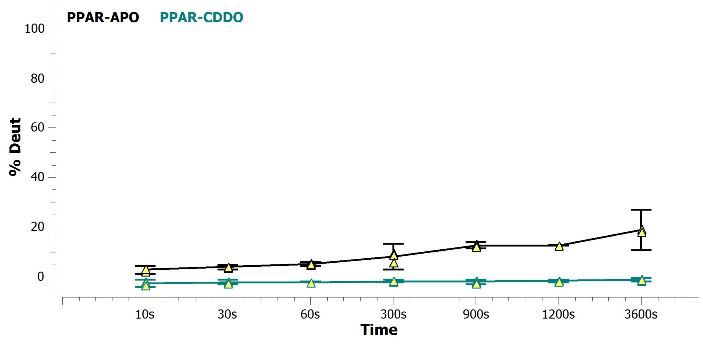

161-169: FRRTIRLKL (#52)

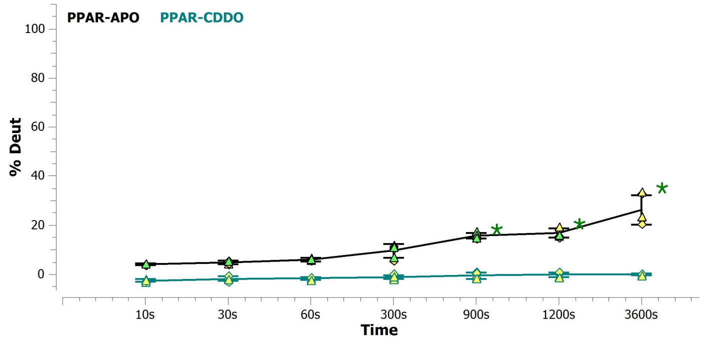

162-169: RRTIRLKL (#53)

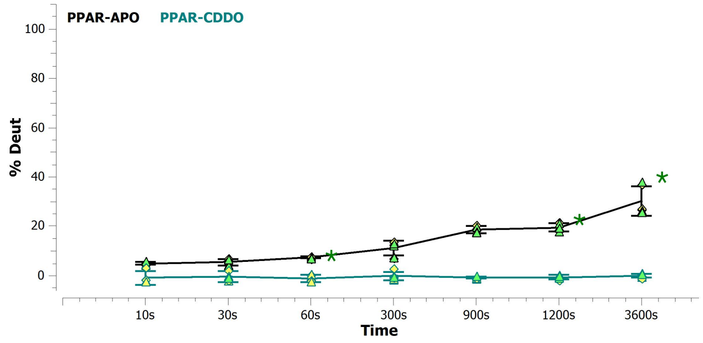

168-176: KLIYDRCDL (#54)

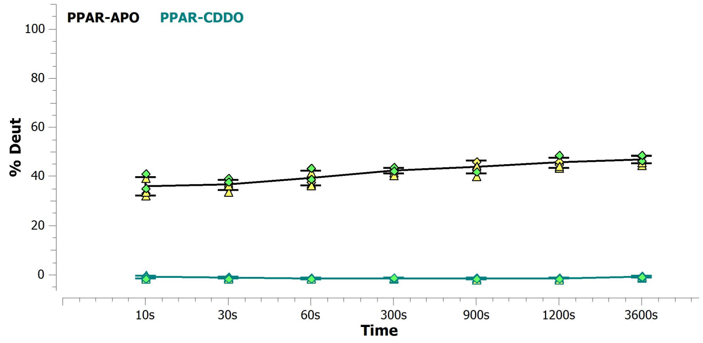

170-176: IYDRCDL (#55)

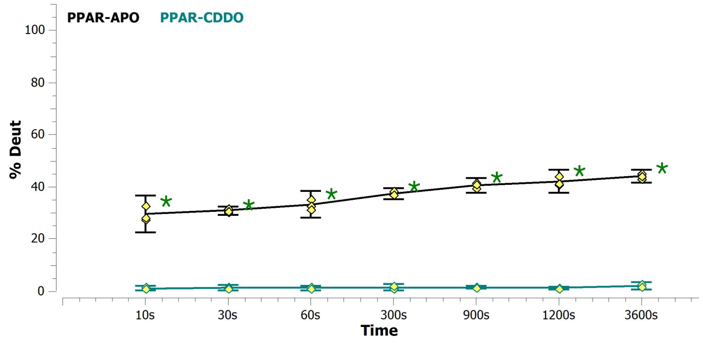

194-208: QKCLAVGMSHNAIRF (#56)

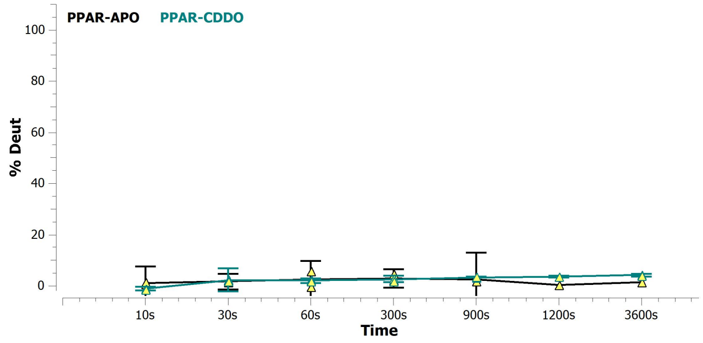

197-208: LAVGMSHNAIRF (#57)

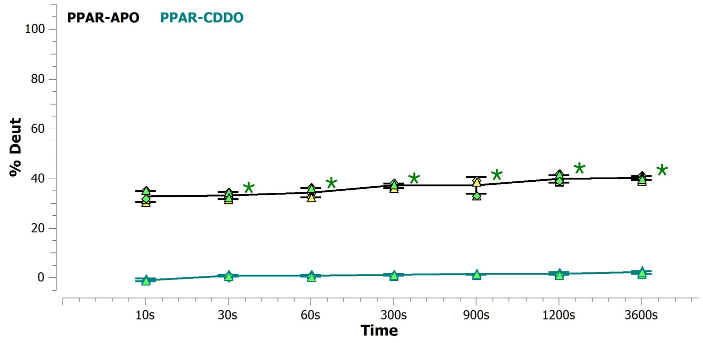

199-208: VGMSHNAIRF (#58)

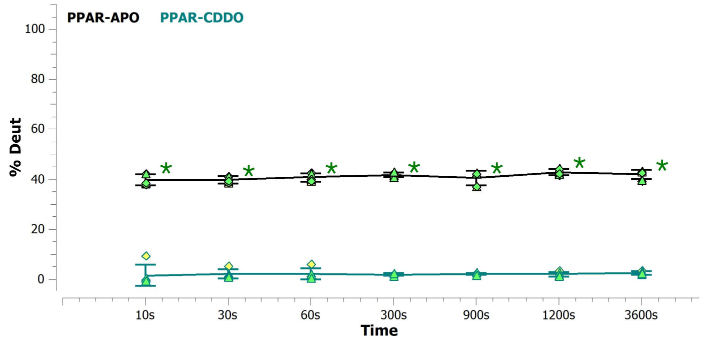

199-219: VGMSHNAIRFGRMPQAEKEKL (#59)

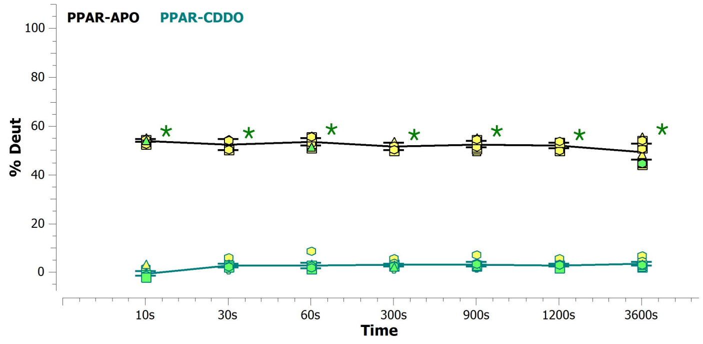

209-219: GRMPQAEKEKL (#60)

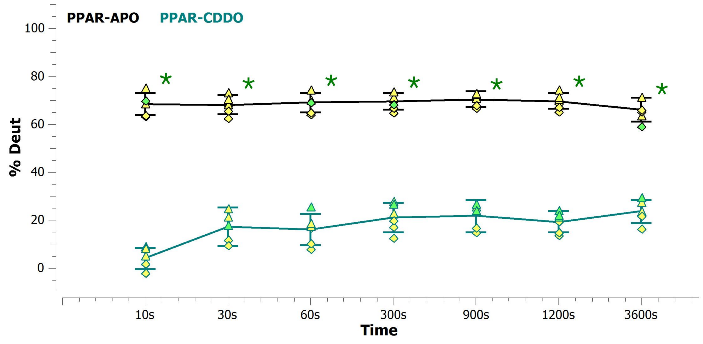

223-236: ISSDIDQLNPESAD (#61)

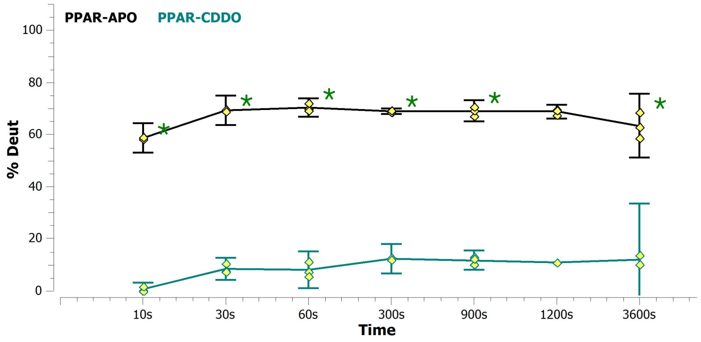

225-237: SDIDQLNPESADL (#62)

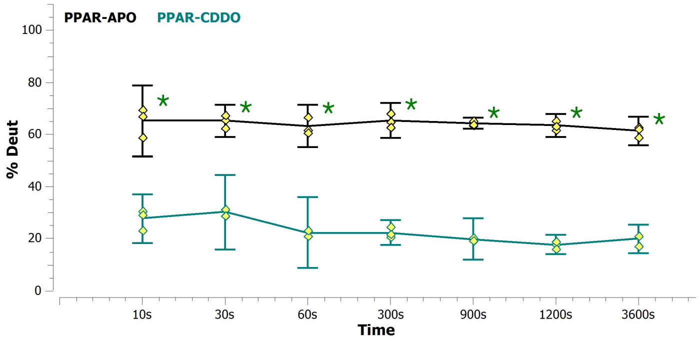

227-237: IDQLNPESADL (#63)

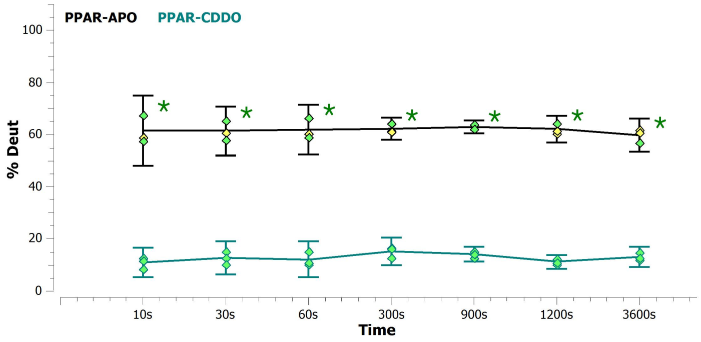

237-248: LRALAKHLYDSY (#64)

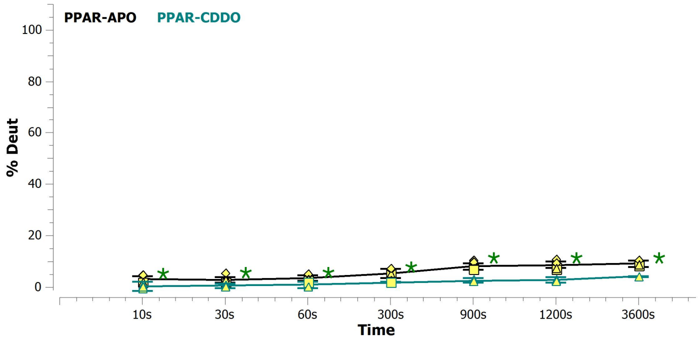

238-247: RALAKHLYDS (#65)

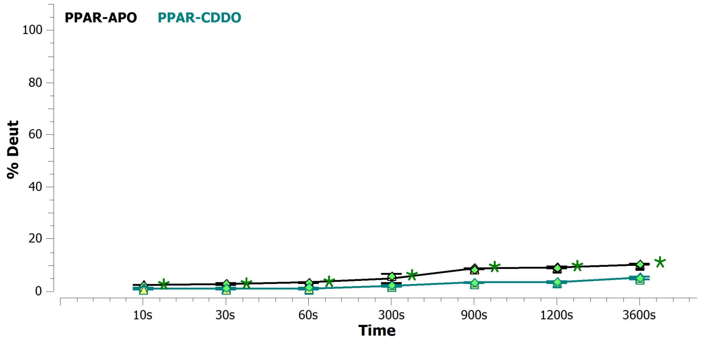

238-248: RALAKHLYDSY (#66)

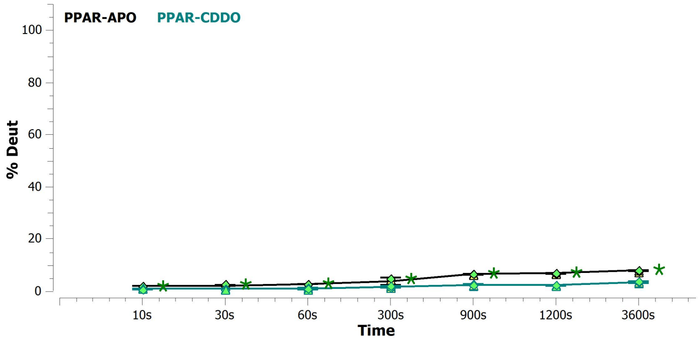

238-263: RALAKHLYDSYIKSFPLTKAKARAIL (#67)

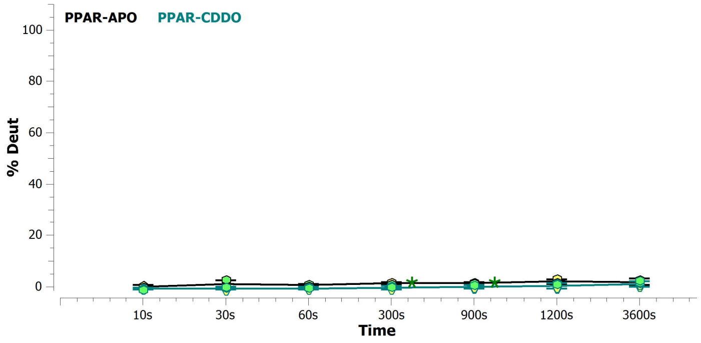

248-261: YIKSFPLTKAKARA (#68)

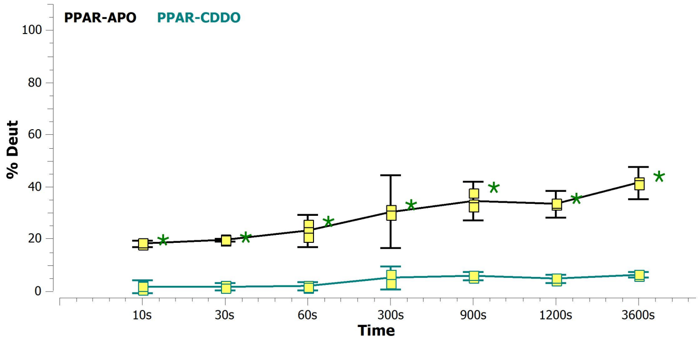

248-263: YIKSFPLTKAKARAIL (#69)

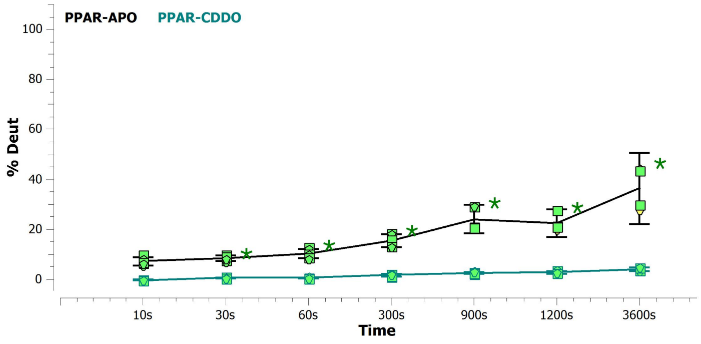

248-278: YIKSFPLTKAKARAILTGKTTDKSPFVIYDM (#70)

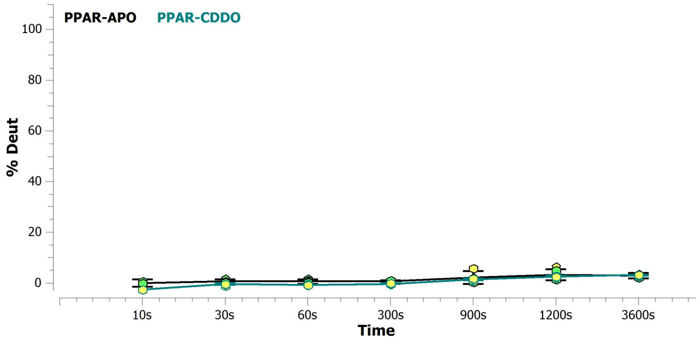

249-261: IKSFPLTKAKARA (#71)

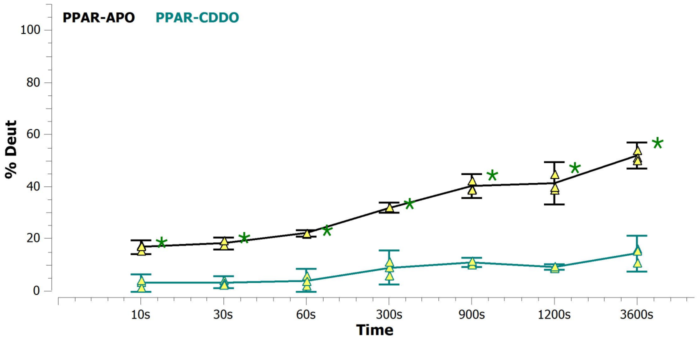

249-263: IKSFPLTKAKARAIL (#72)

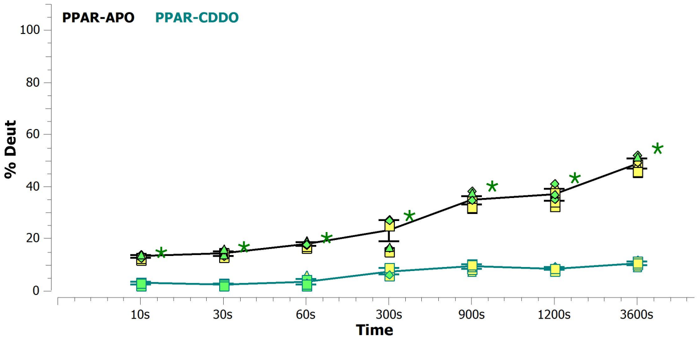

249-278: IKSFPLTKAKARAILTGKTTDKSPFVIYDM (#73)

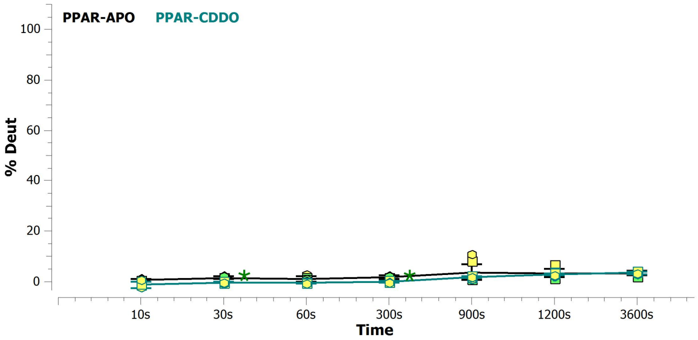

264-276: TGKTTDKSPFVIY (#74)

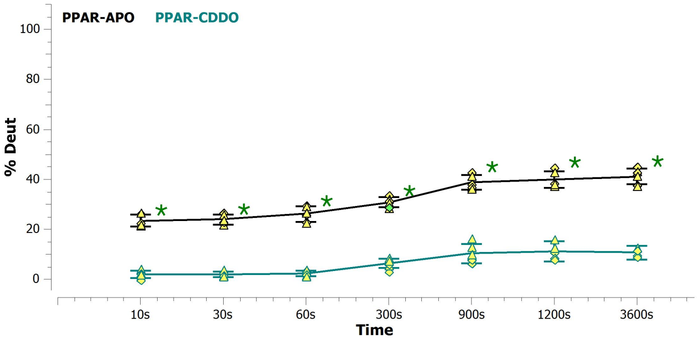

282-298: MMGEDKIKFKHITPLQE (#75)

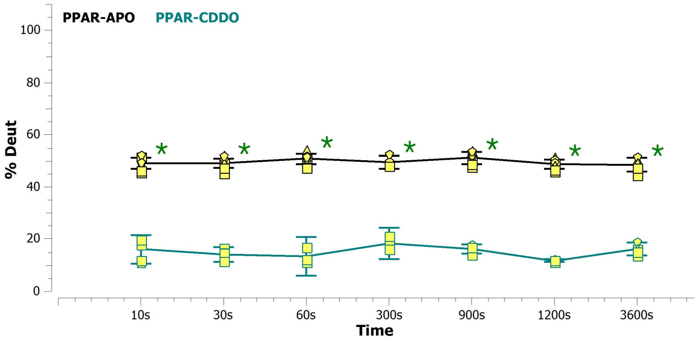

283-302: MGEDKIKFKHITPLQEQSKE (#76)

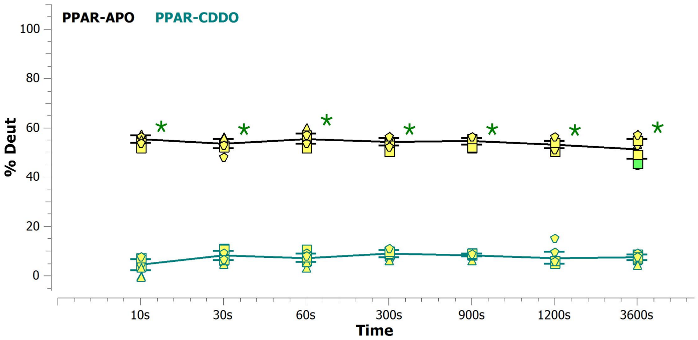

284-304: GEDKIKFKHITPLQEQSKEVA (#77)

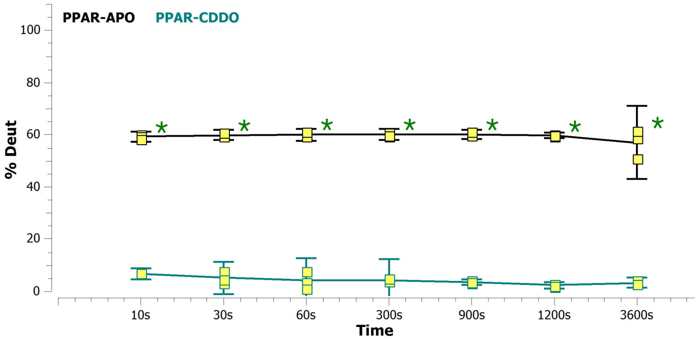

287-302: KIKFKHITPLQEQSKE (#78)

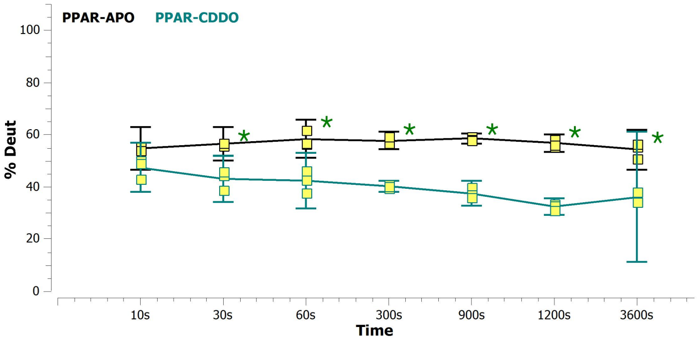

303-312: VAIRIFQGCQ (#79)

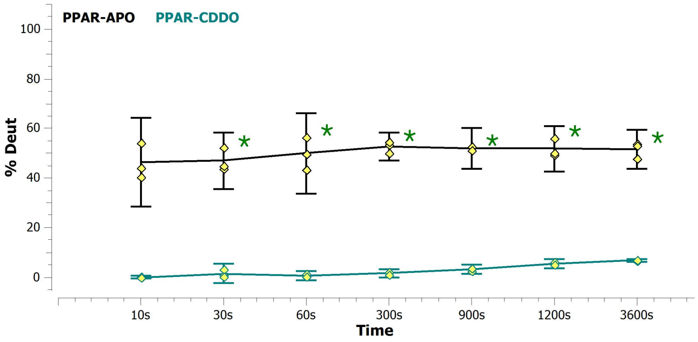

305-312: IRIFQGCQ (#80)

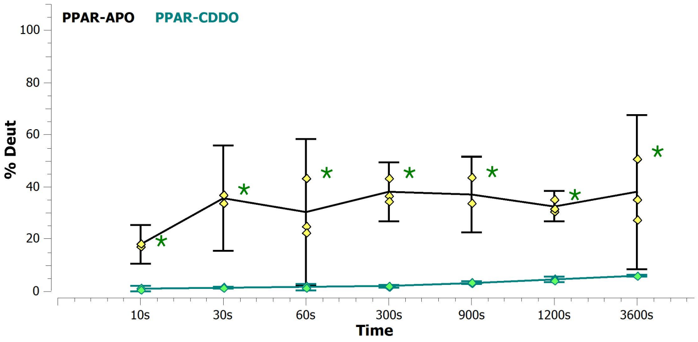

314-324: RSVEAVQEITE (#81)

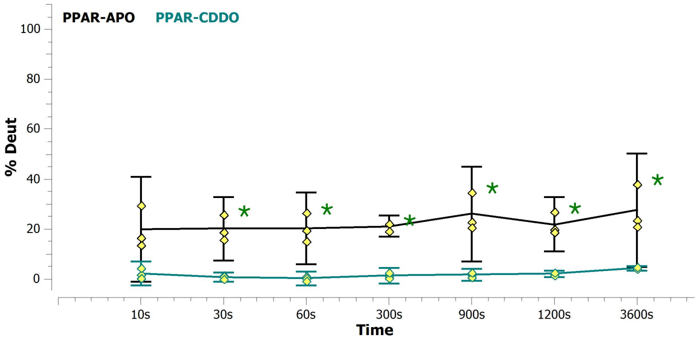

325-332: YAKNIPGF (#82)

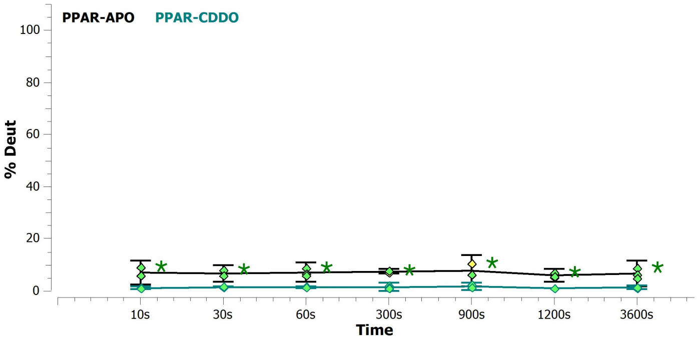

325-335: YAKNIPGFINL (#83)

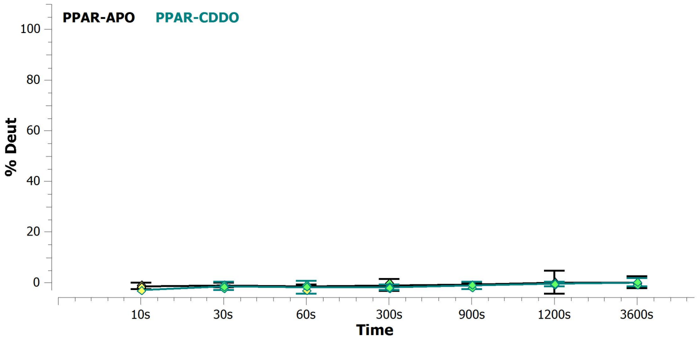

336-343: DLNDQVTL (#84)

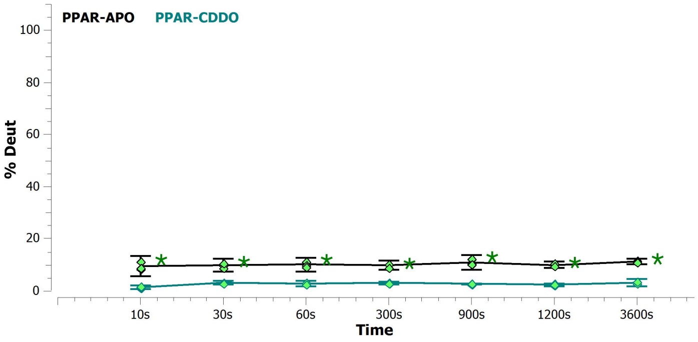

344-350: LKYGVHE (#85)

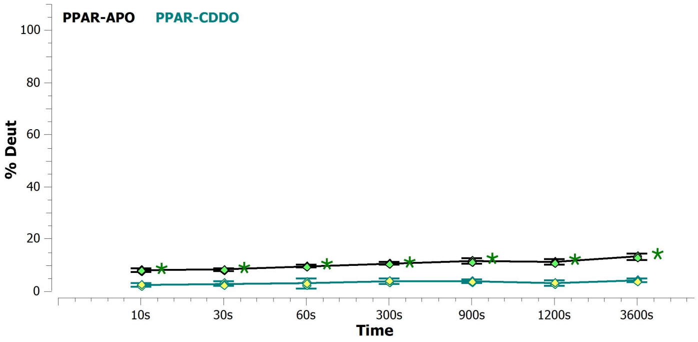

344-353: LKYGVHEIIY (#86)

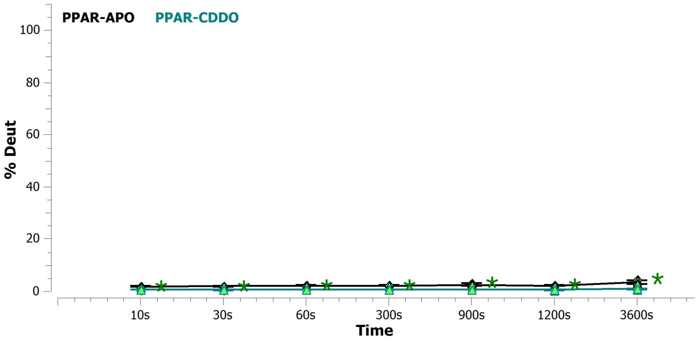

357-366: ASLMNKDGVL (#87)

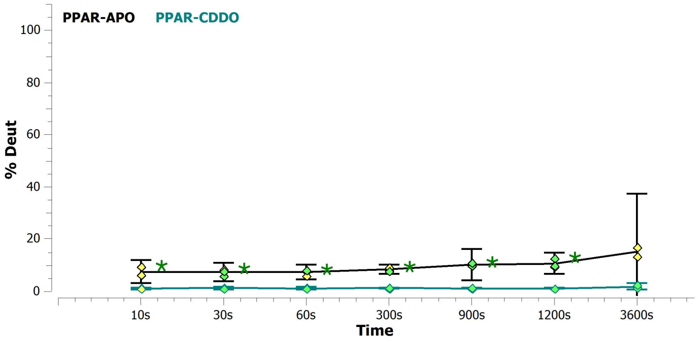

360-366: MNKDGVL (#88)

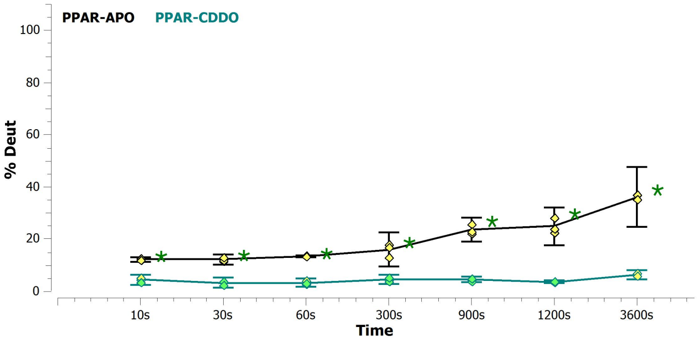

367-377: ISEGQGFMTRE (#89)

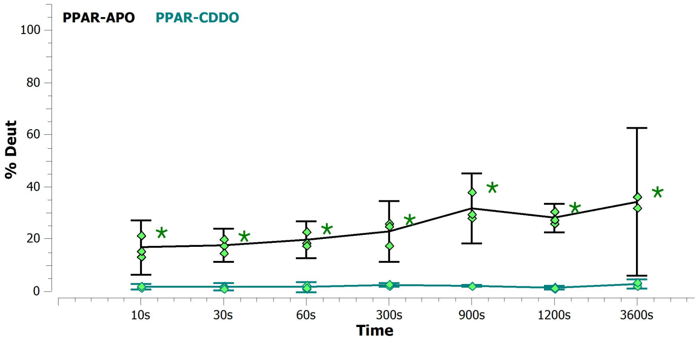

374-389: MTREFLKSLRKPFGDF (#90)

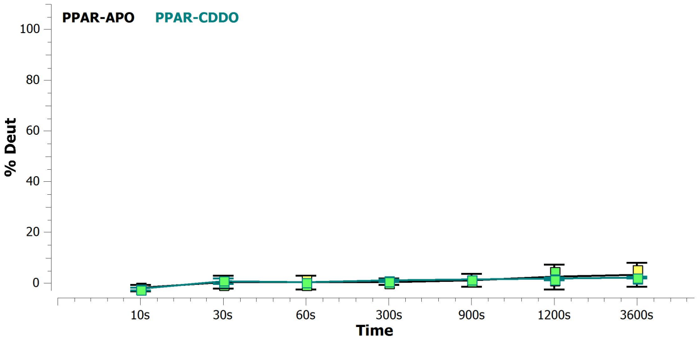

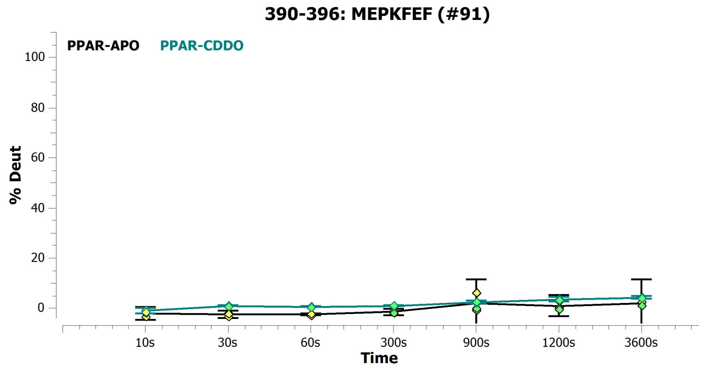
390-396: MEPKFEF (#91)
PPAR-APO
PPAR-CDDO
% Deut
0 20 40 60 80 100
10s 30s 60s 300s 900s 1200s 3600s
Time

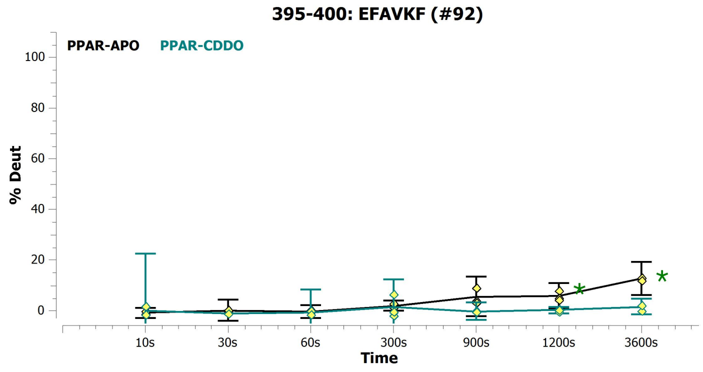
395-400: EFAVKF (#92)
PPAR-APO
PPAR-CDDO
% Deut
0 20 40 60 80 100
10s 30s 60s 300s 900s 1200s 3600s
Time

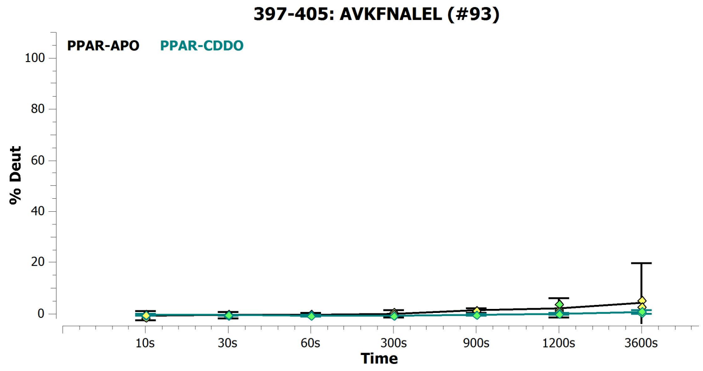
397-405: AVKFNALEL (#93)
PPAR-APO
PPAR-CDDO
% Deut
0 20 40 60 80 100
10s 30s 60s 300s 900s 1200s 3600s
Time

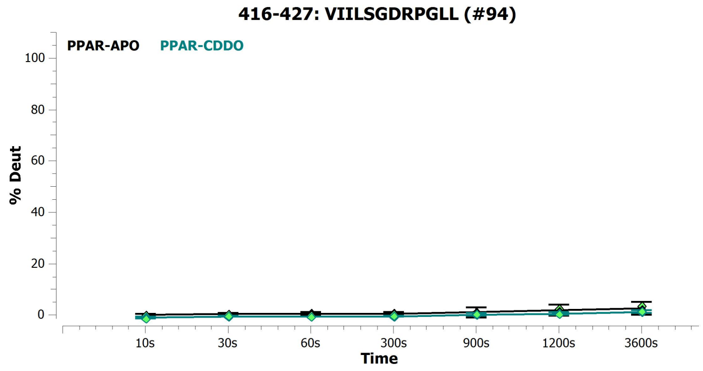
416-427: VIILSGDRPGLL (#94)
PPAR-APO
PPAR-CDDO
% Deut
0 20 40 60 80 100
10s 30s 60s 300s 900s 1200s 3600s
Time

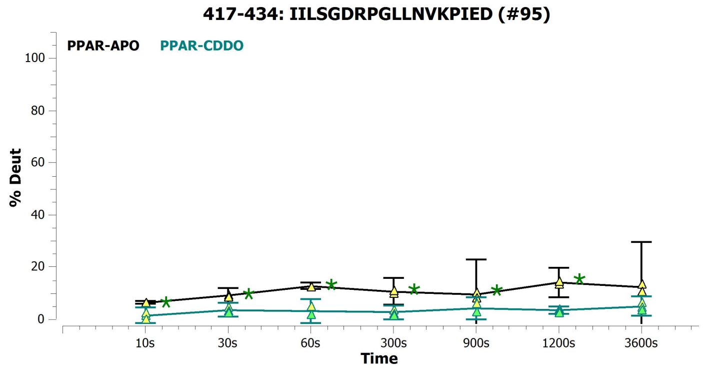
417-434: IILSGDRPGLLNVKPIED (#95)
PPAR-APO
PPAR-CDDO
% Deut
0 20 40 60 80 100
10s 30s 60s 300s 900s 1200s 3600s
Time

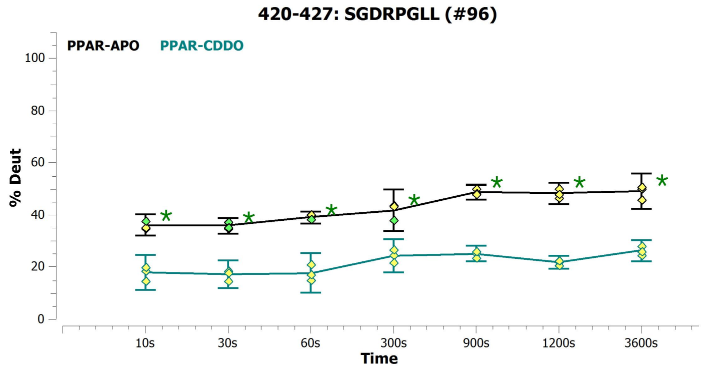
420-427: SGDRPGLL (#96)
PPAR-APO
PPAR-CDDO
% Deut
0 20 40 60 80 100
10s 30s 60s 300s 900s 1200s 3600s
Time

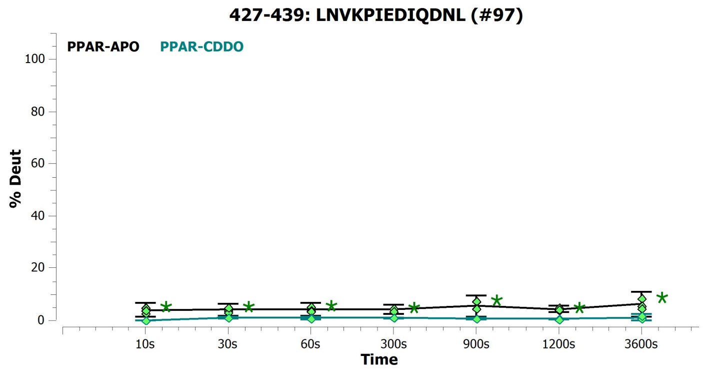
427-439: LNVKPIEDIQDNL (#97)
PPAR-APO
PPAR-CDDO
% Deut
0 20 40 60 80 100
10s 30s 60s 300s 900s 1200s 3600s
Time

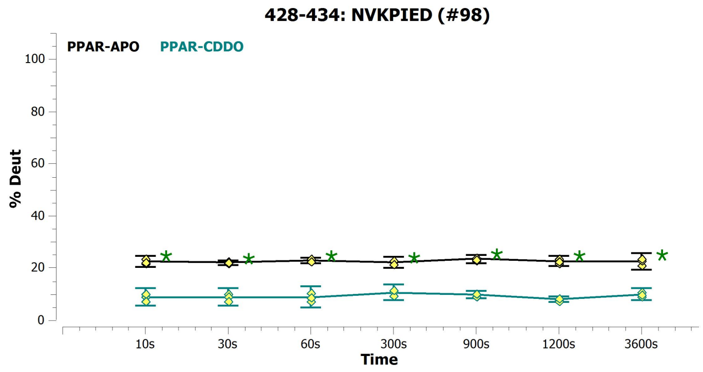
428-434: NVKPIED (#98)
PPAR-APO
PPAR-CDDO
% Deut
0 20 40 60 80 100
10s 30s 60s 300s 900s 1200s 3600s
Time

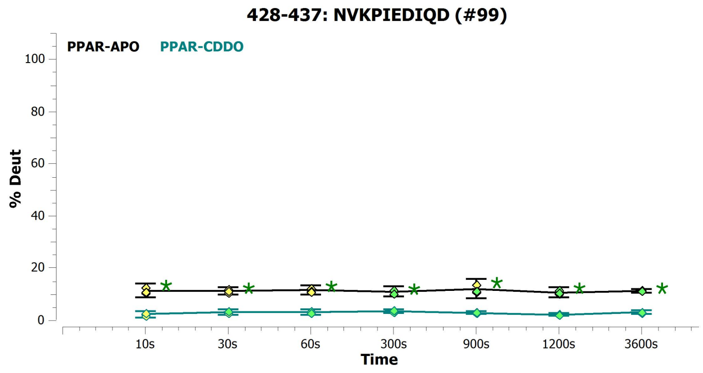
428-437: NVKPIEDIQD (#99)
PPAR-APO
PPAR-CDDO
% Deut
0 20 40 60 80 100
10s 30s 60s 300s 900s 1200s 3600s
Time

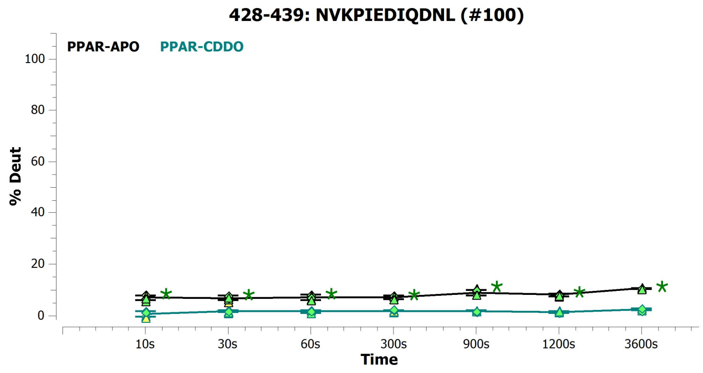
428-439: NVKPIEDIQDNL (#100)
PPAR-APO
PPAR-CDDO
% Deut
0 20 40 60 80 100
10s 30s 60s 300s 900s 1200s 3600s
Time

434-439: DIQDNL (#101)

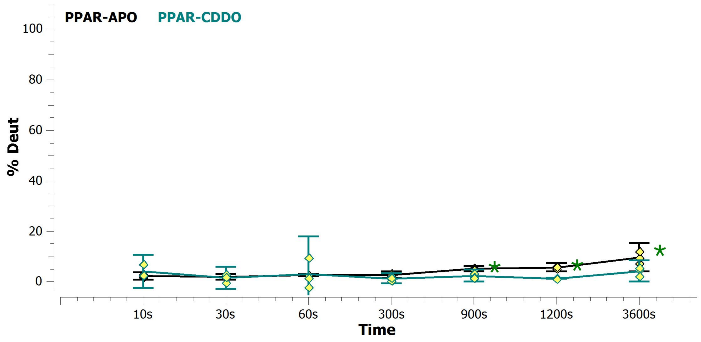

434-440: DIQDNLL (#102)

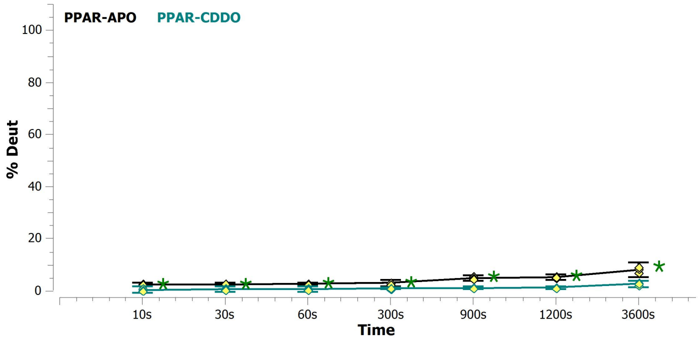

435-442: IQDNLLQA (#103)

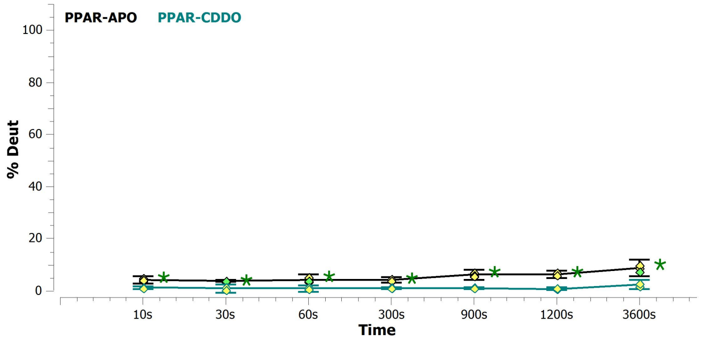

443-457: LELQLKLNHPESSQL (#104)

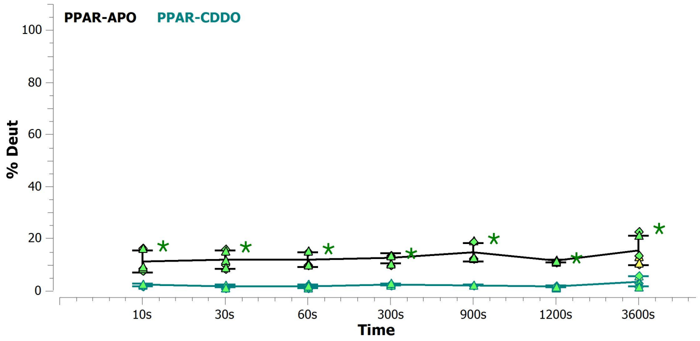

444-457: ELQLKLNHPESSQL (#105)

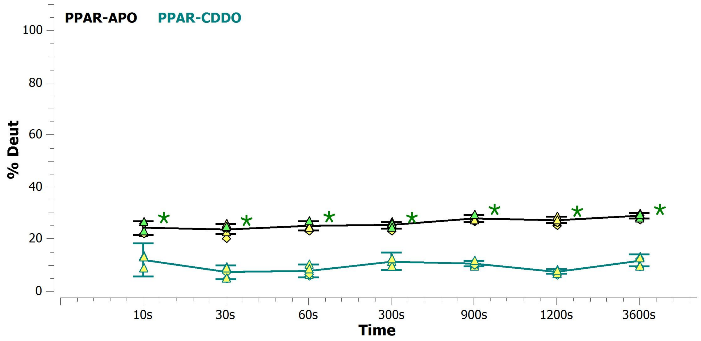

445-457: LQLKLNHPESSQL (#106)

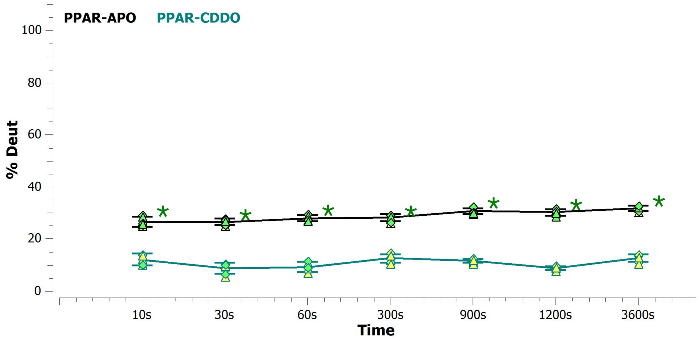

446-457: QLKLNHPESSQL (#107)

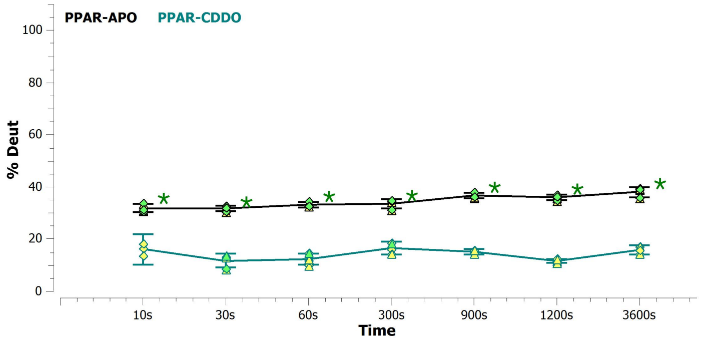

458-467: FAKVLQKMTD (#108)

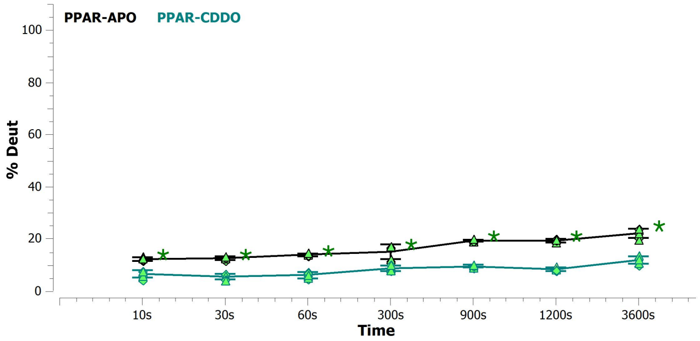

458-468: FAKVLQKMTDL (#109)

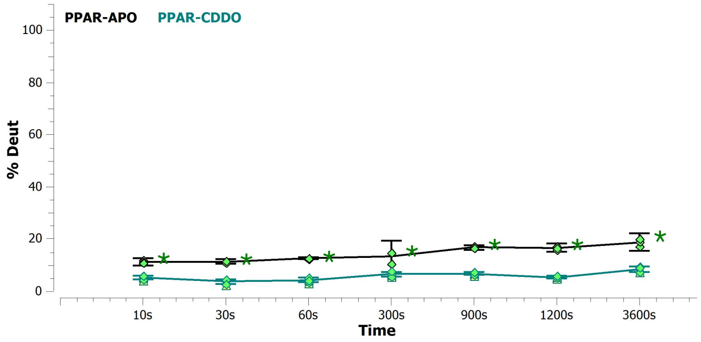

458-470: FAKVLQKMTDLRQ (#110)

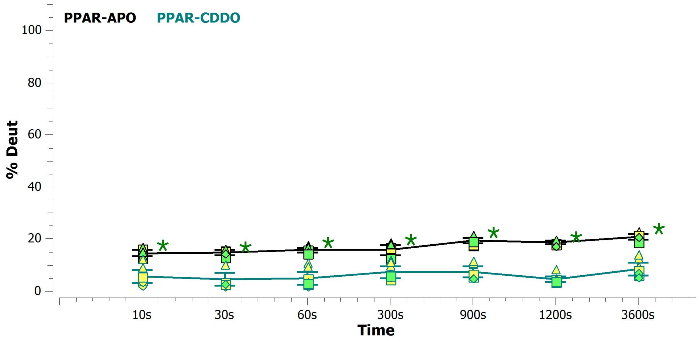

459-468: AKVLQKMTDL (#111)

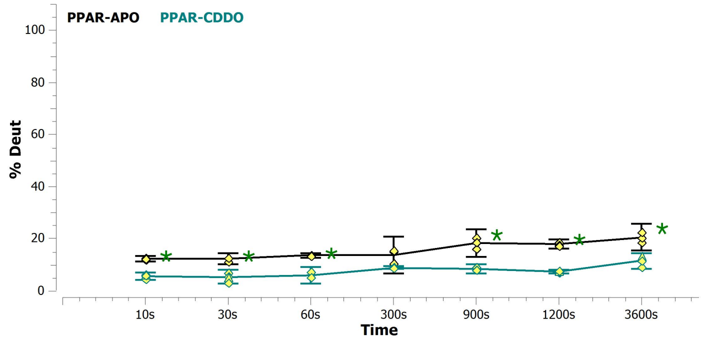

468-474: LRQIVTE (#112)

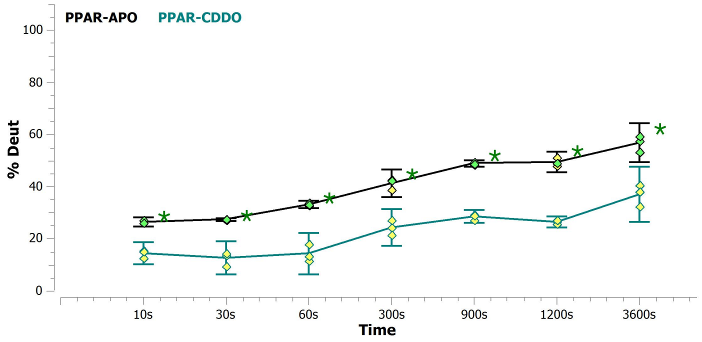

475-488: HVQLLHVIKKTETD (#113)

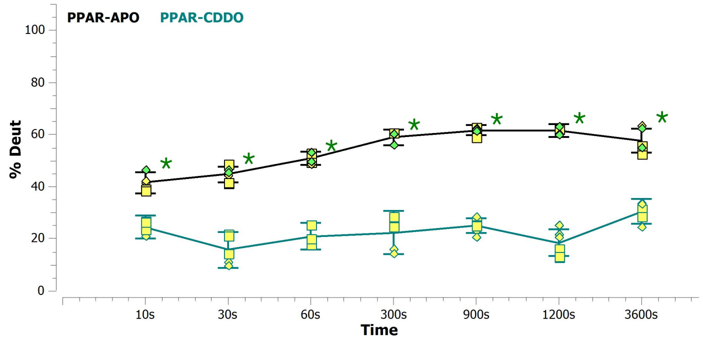

475-489: HVQLLHVIKKTETDM (#114)

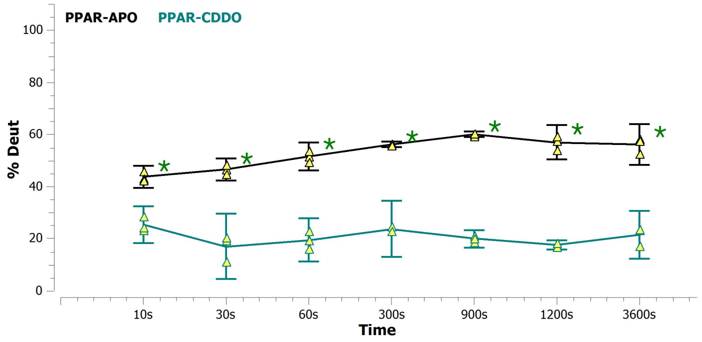

479-491: LHVIKKTETDMSL (#115)

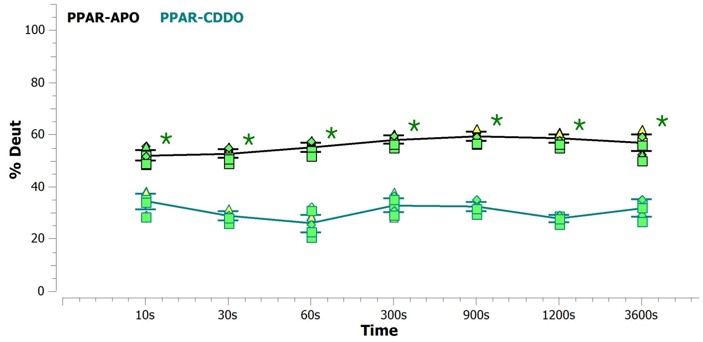

489-495: MSLHPLL (#116)

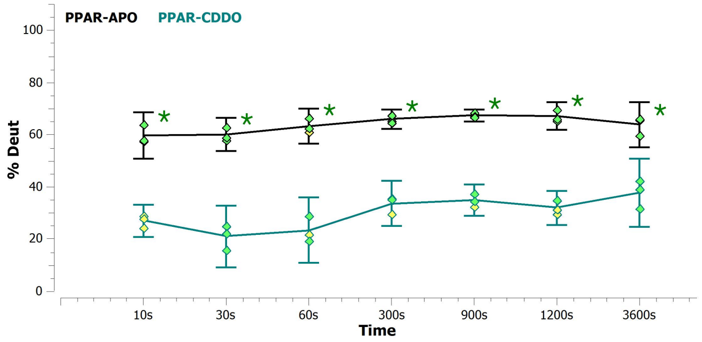

490-497: SLHPLLQE (#117)

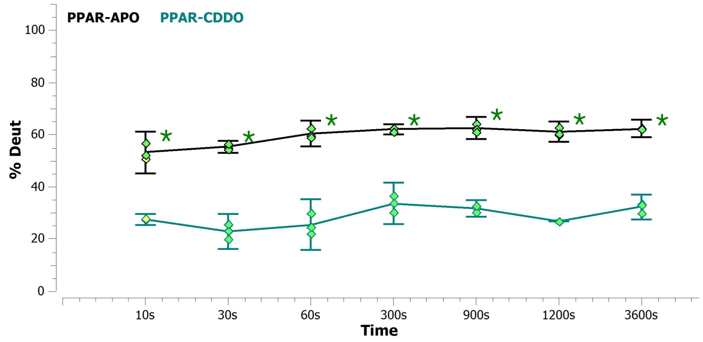

492-497: HPLLQE (#118)

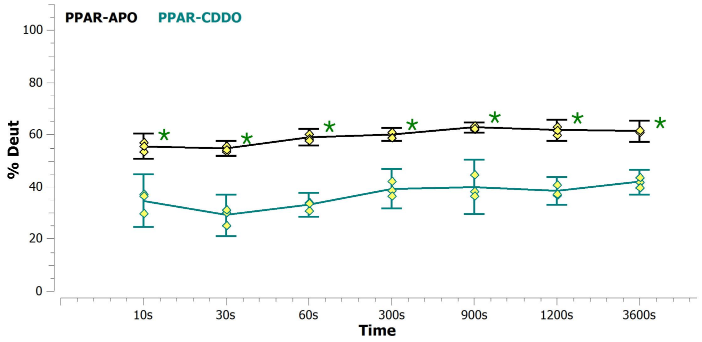

496-503: QEIYKDLY (#119)

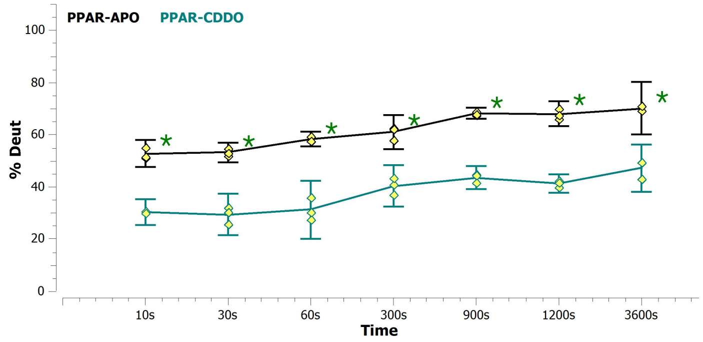

498-503: IYKDLY (#120)

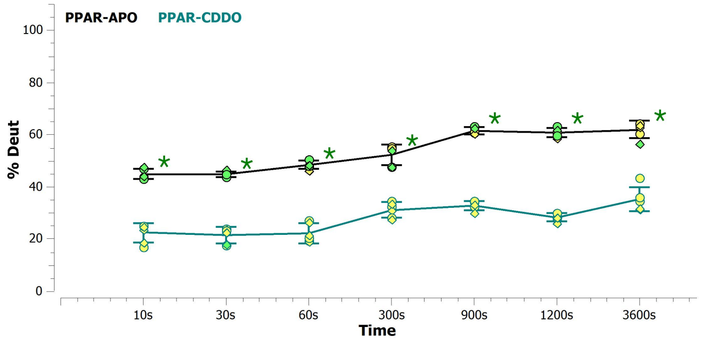

Supplement: Supplementary file 3 — Supplemental dataset 2 [file 41418_2022_1077_MOESM3_ESM.pdf]
