# Supplementary material for: Blockage of PPARγ T166 phosphorylation enhances the inducibility of beige adipocytes and improves metabolic dysfunctions
Source: Cell Death Differ. 2022 Nov 3;30(3):766–78. doi: 10.1038/s41418-022-01077-x (PMC9984430; doi:10.1038/s41418-022-01077-x)
Supplement: Supplementary file 7 — Uncropped Western blotting data [file 41418_2022_1077_MOESM7_ESM.pdf]

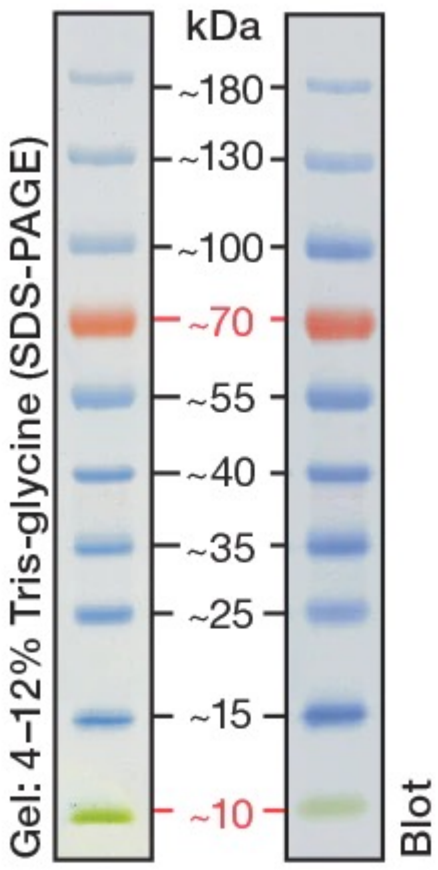

Thermo Fisher (26616)

WB marker used in this study

Figure 1d

Full unedited gel for Figure 1d

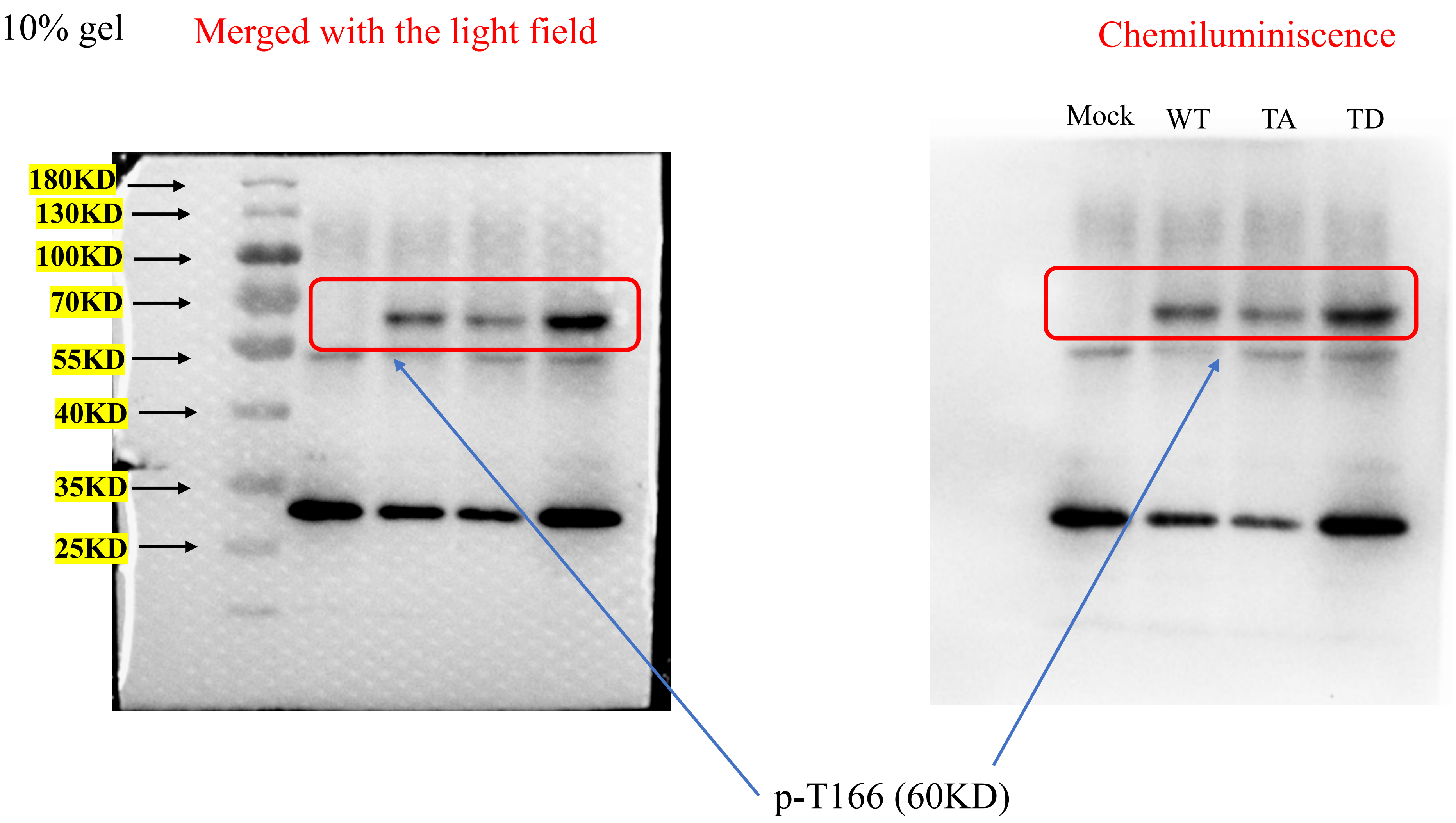

Antibody: T166 phosphorylation antibody generated by our lab

Full unedited gel for Figure 1d

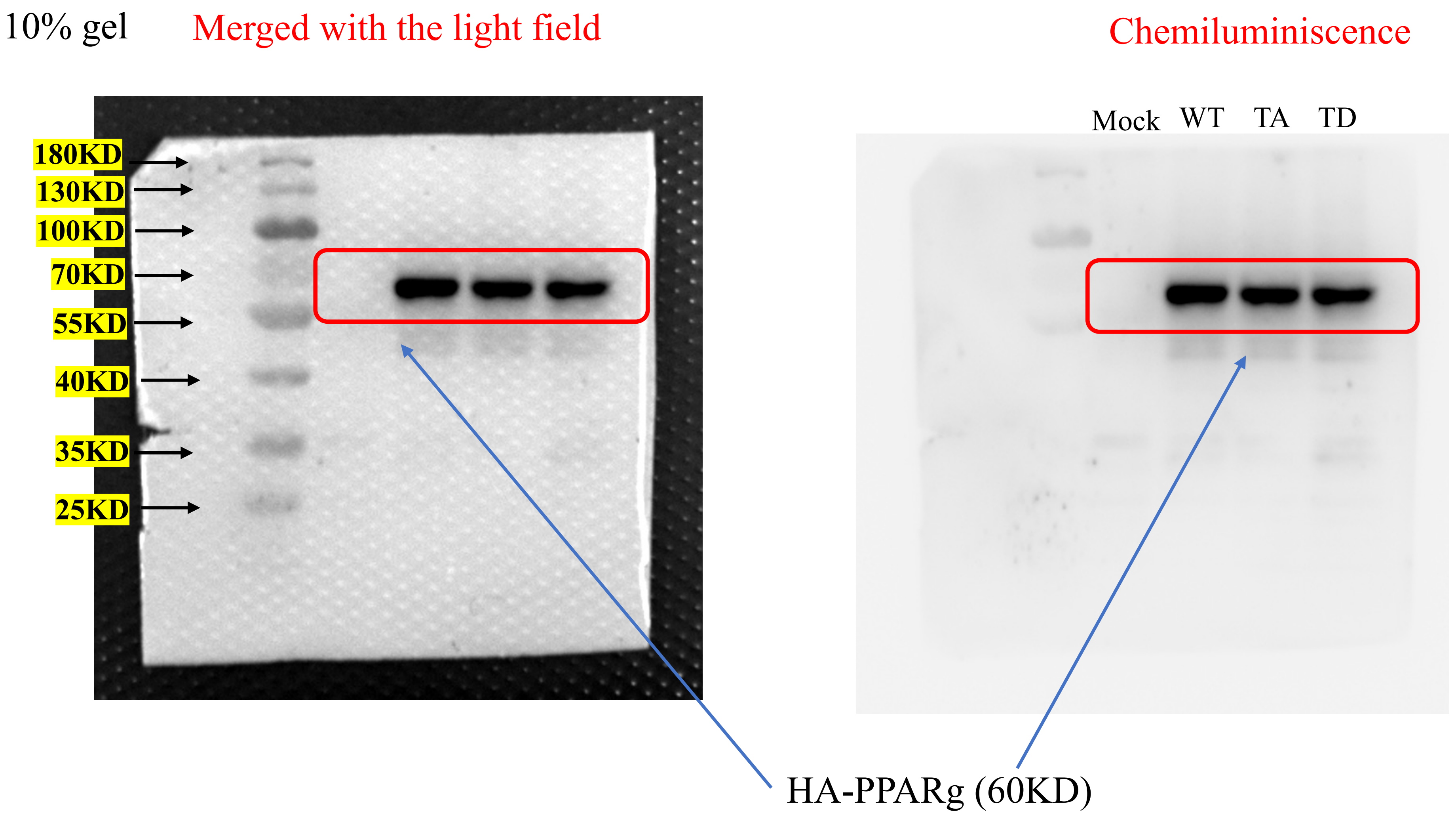

Antibody: Santa cruz PPARg antibody (sc-7273)

Full unedited gel for Figure 1d

10% gel

Merged with the light field

Chemiluminiscence

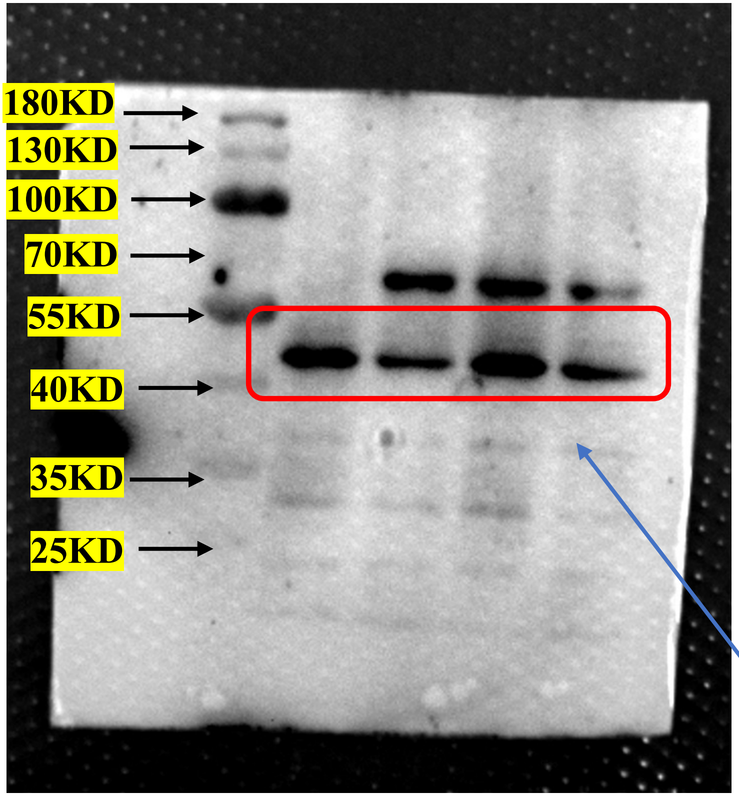

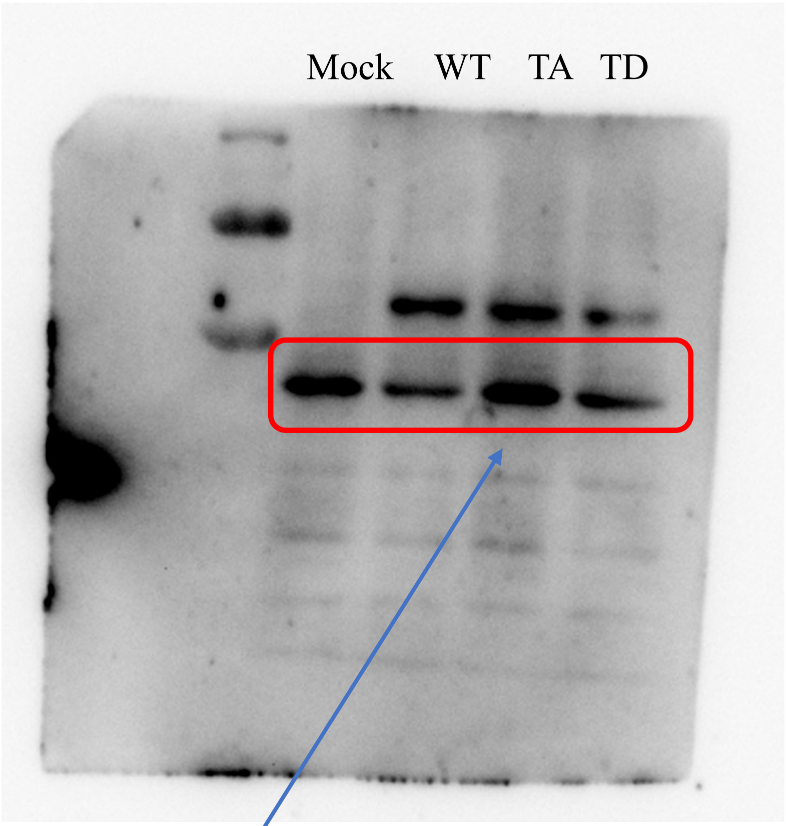

Antibody: b-Actin (Genscript A00702)

b-actin (43KD)

Figure 1f

Full unedited gel for Figure 1f

10% gel   Merged with the light field

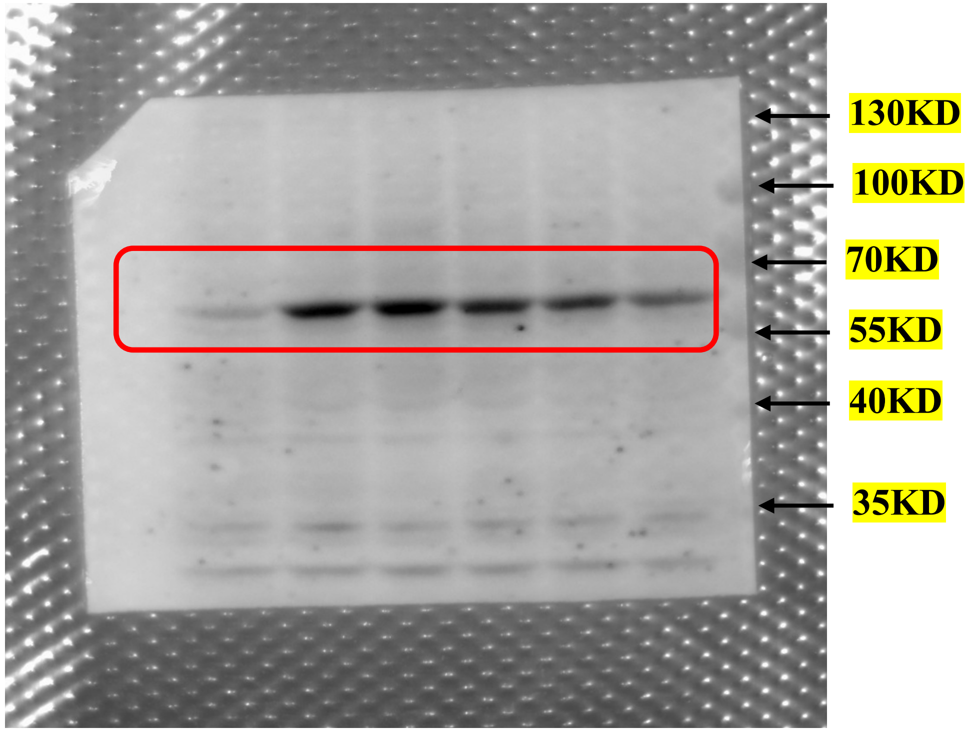

Chemiluminiscence

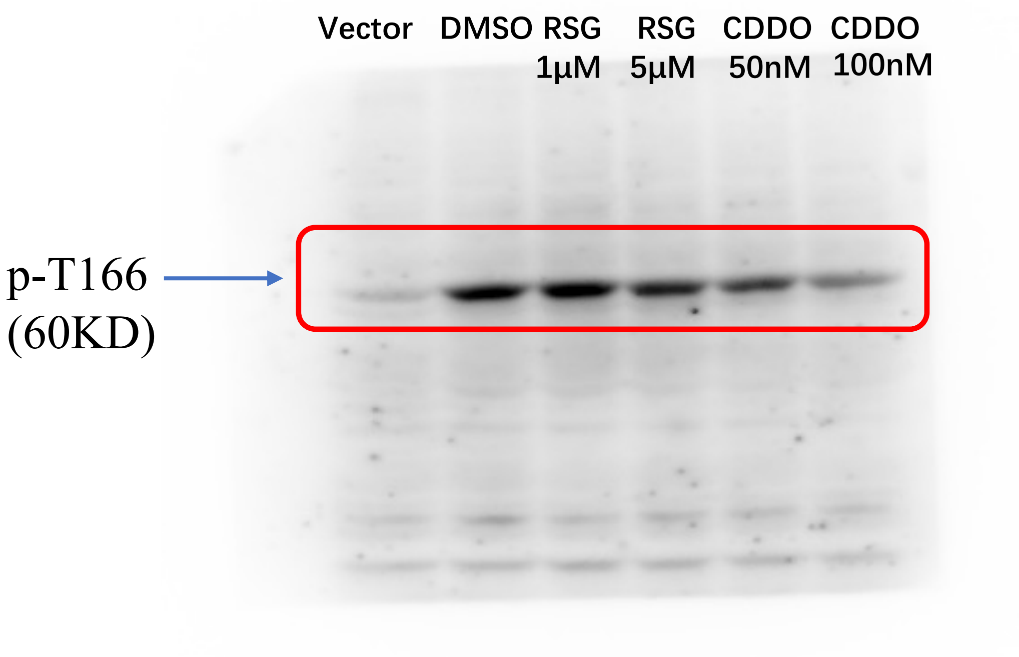

Antibody: T166 phosphorylation antibody generated by our lab

Full unedited gel for Figure 1f

10% gel

Merged with the light field

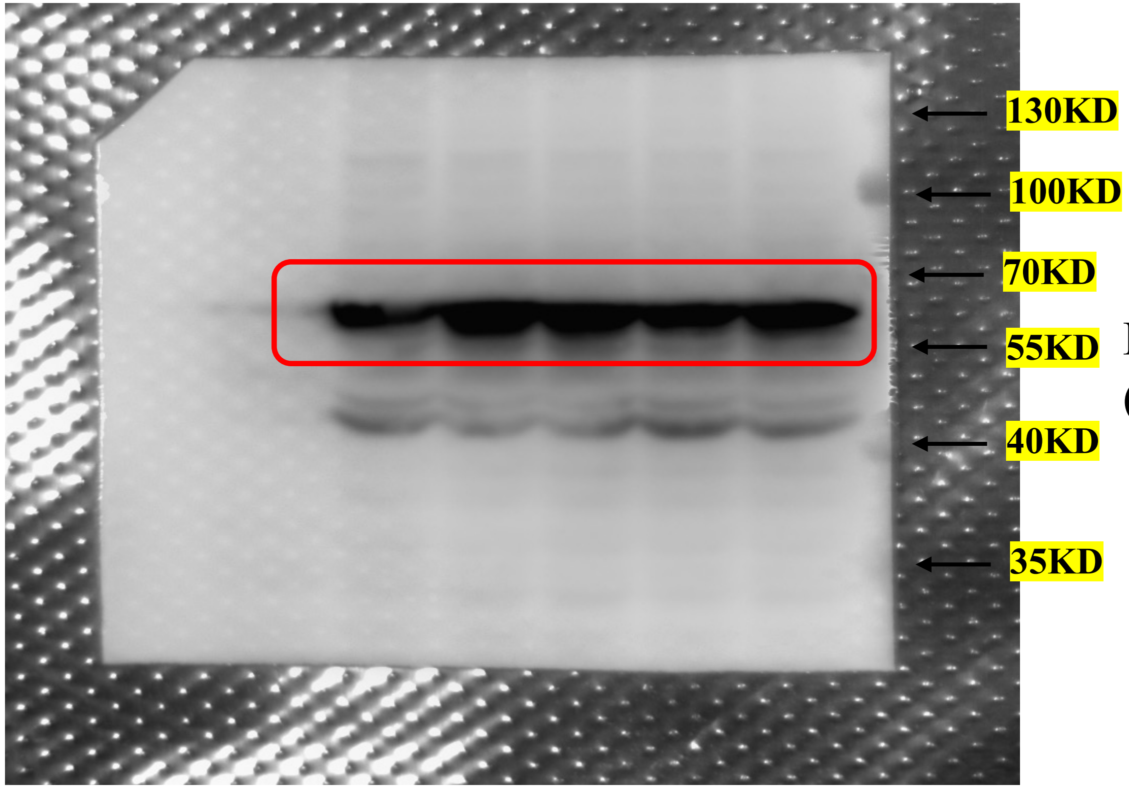

HA-PPARg2
(60KD)

Chemiluminiscence

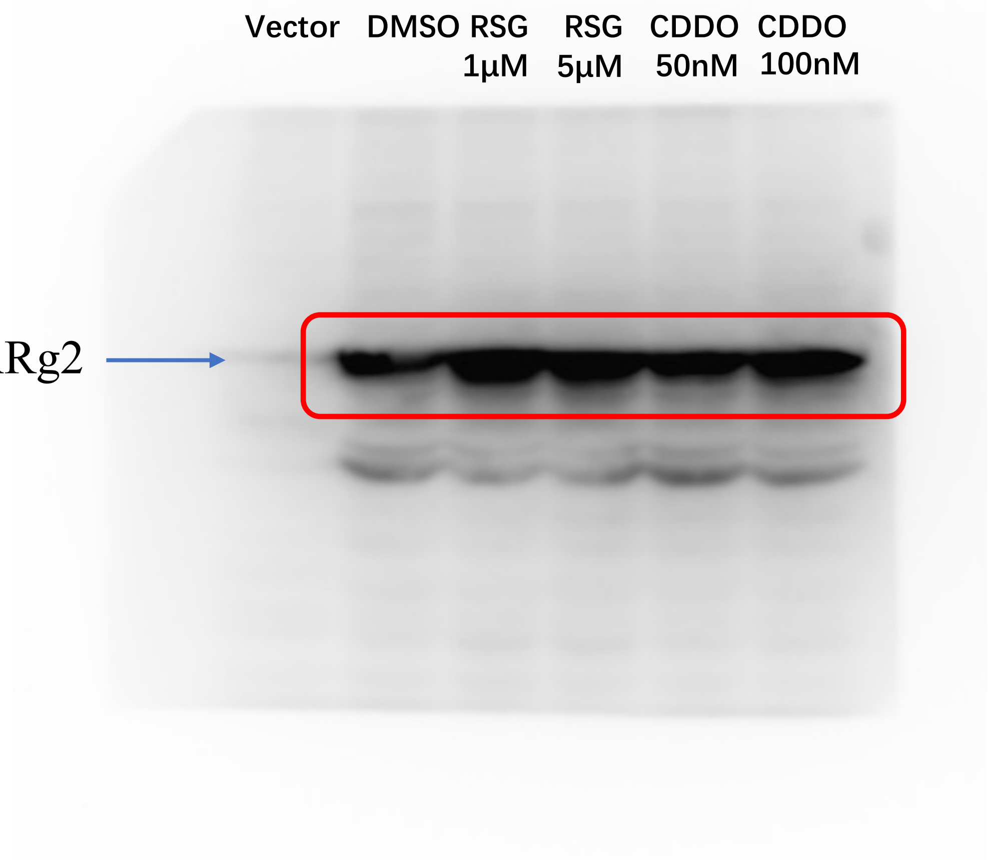

Antibody: PPARg antibody (Cell signaling technology #81b8)

Full unedited gel for Figure 1f

10% gel

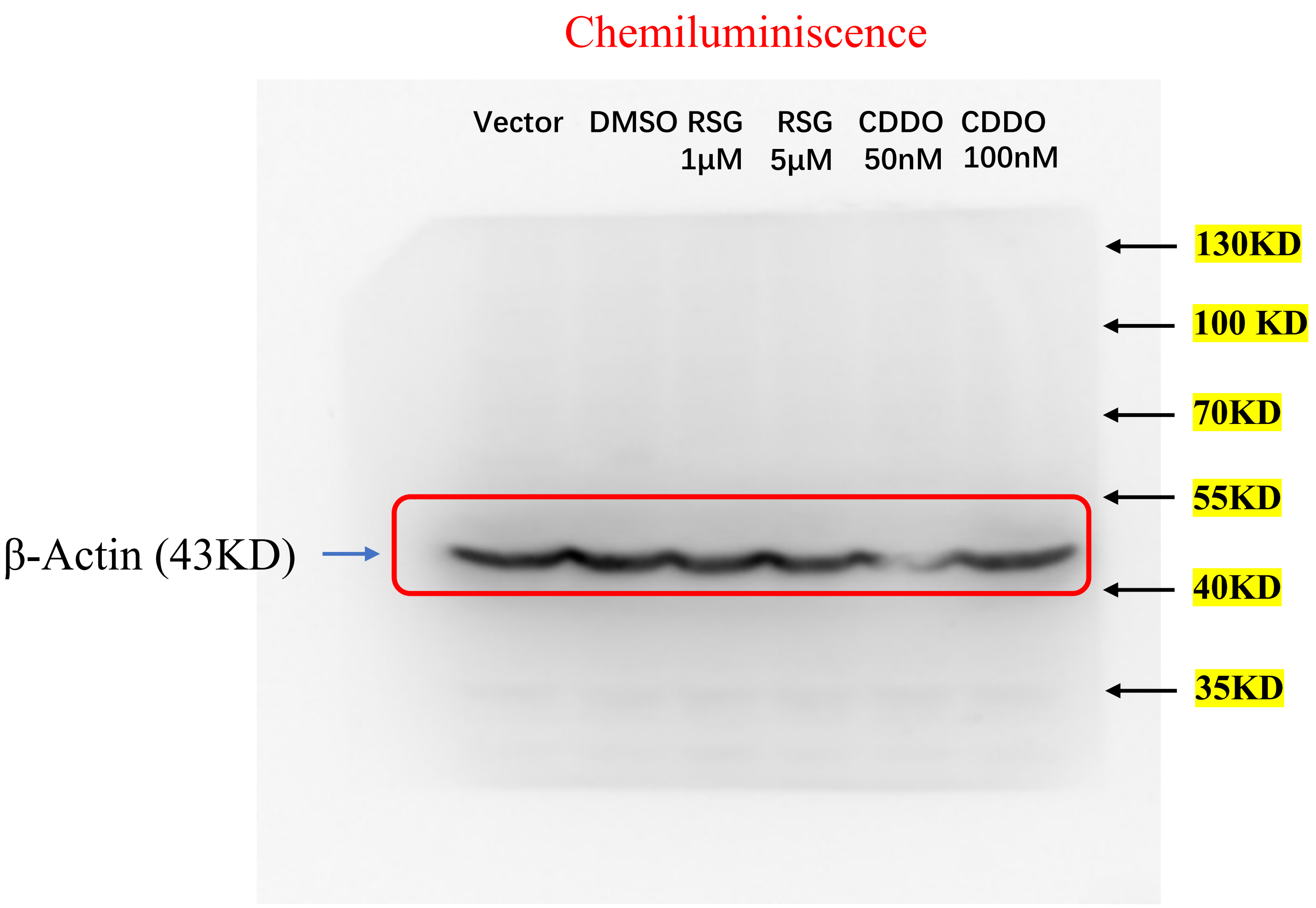

Antibody: b-Actin (Genscript A00702)

Figure 1g

Full unedited gel for Figure 1g

IP

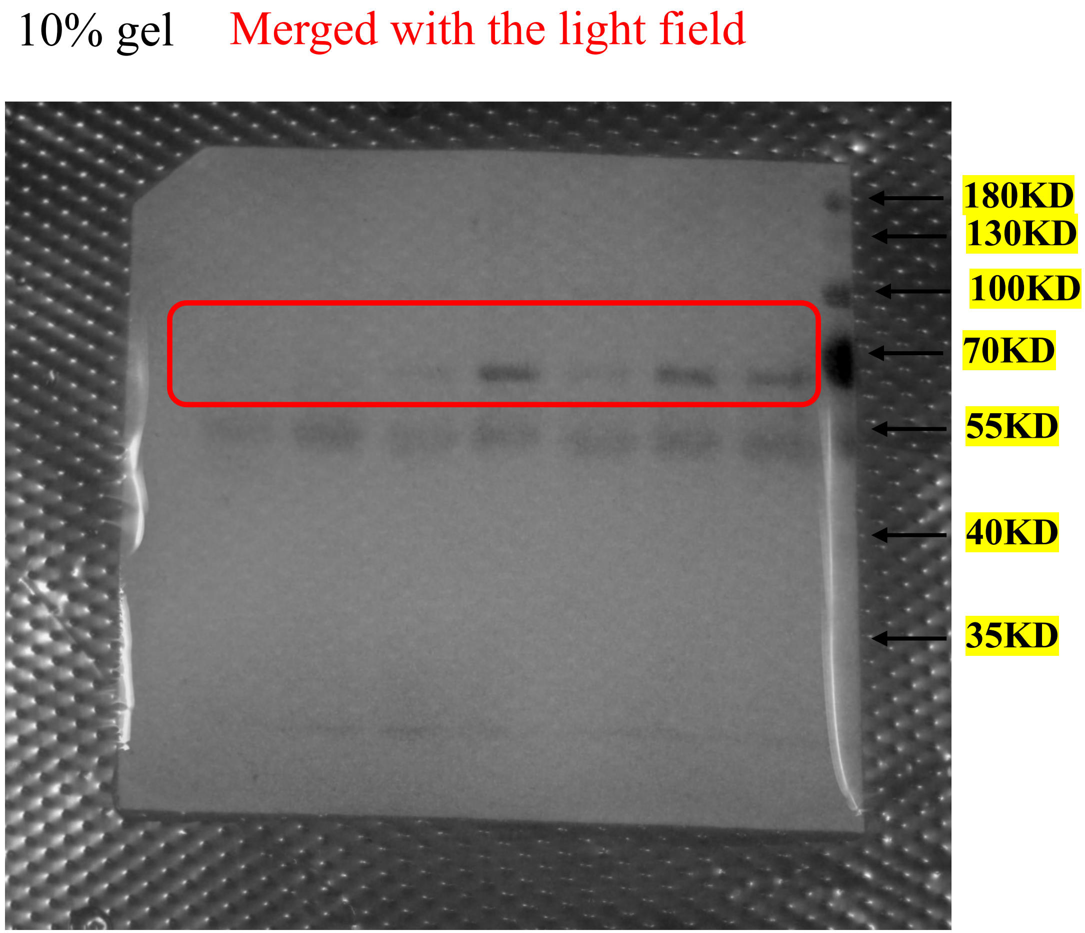

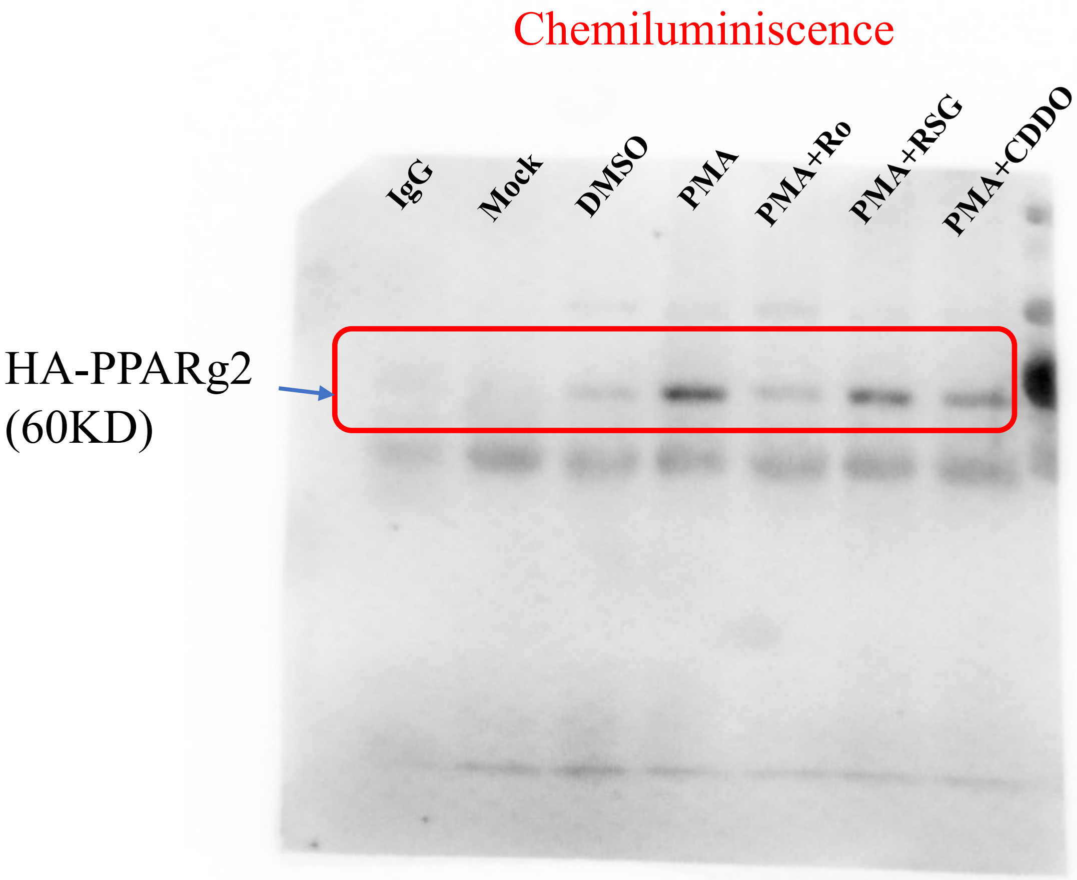

Antibody: PPARg antibody (Cell signaling technology #81b8)

Full unedited gel for Figure 1g

10% gel

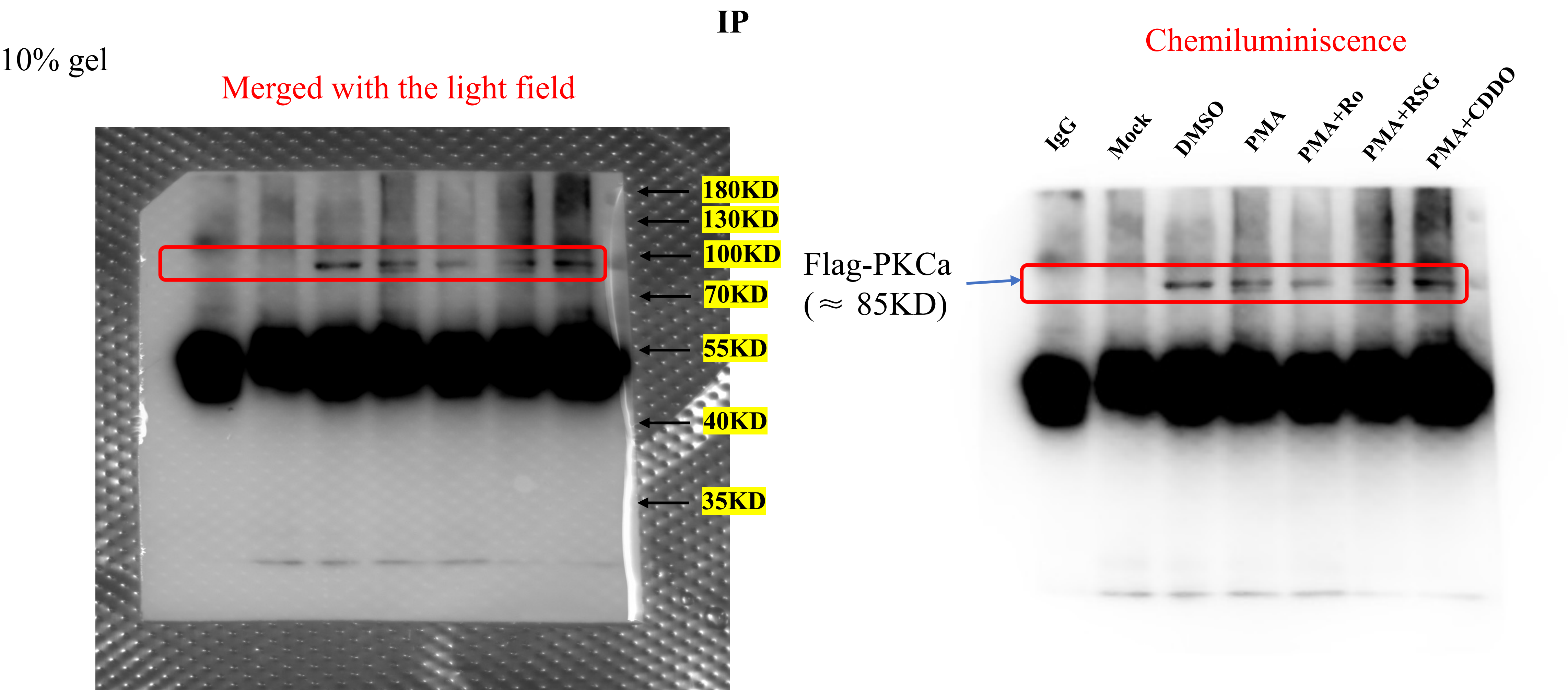

Antibody: PKCa antibody (Cell signaling technology #2056)

Full unedited gel for Figure 1g

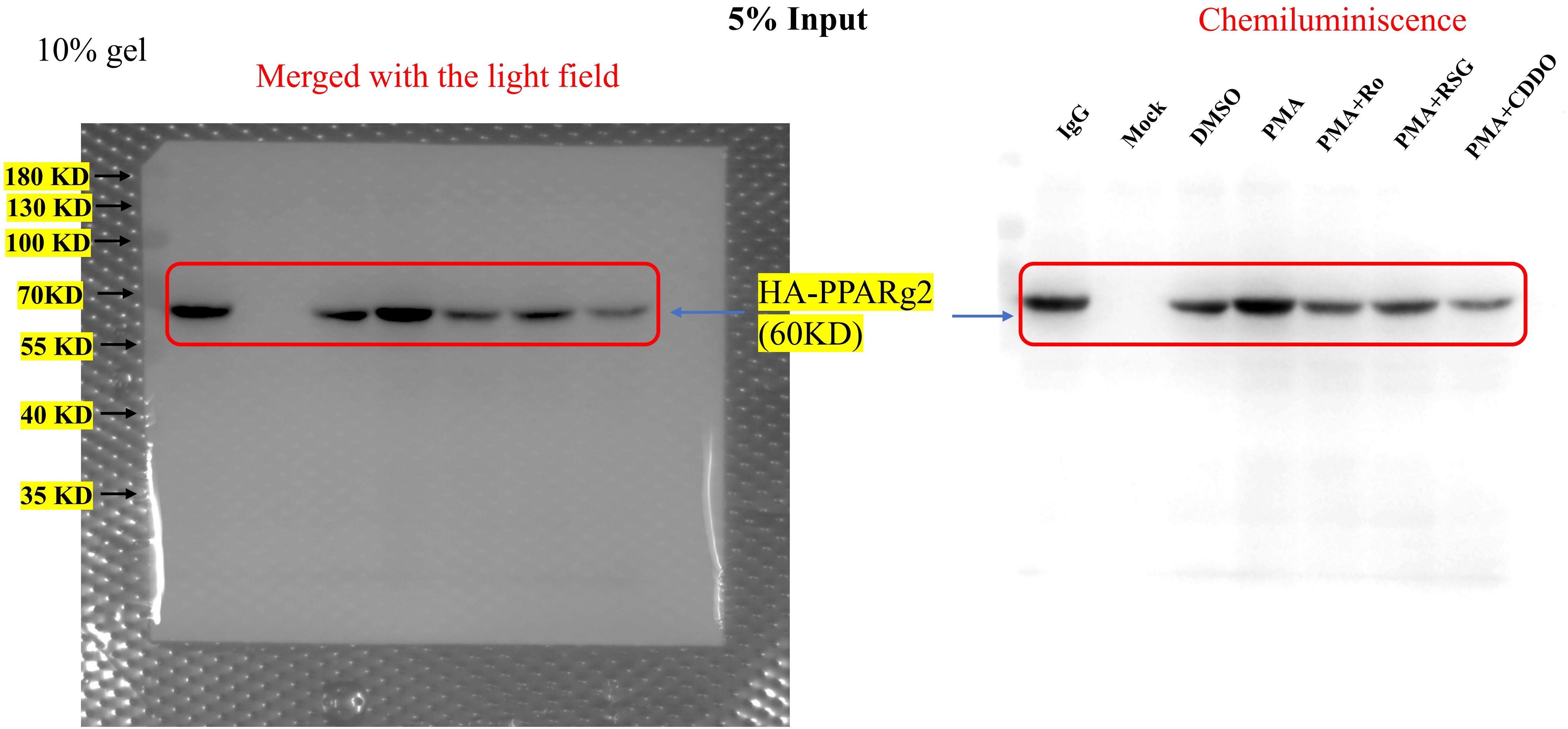

Antibody: Santa cruz PPARg antibody (sc-7273)

Full unedited gel for Figure 1g

10% gel

5% Input

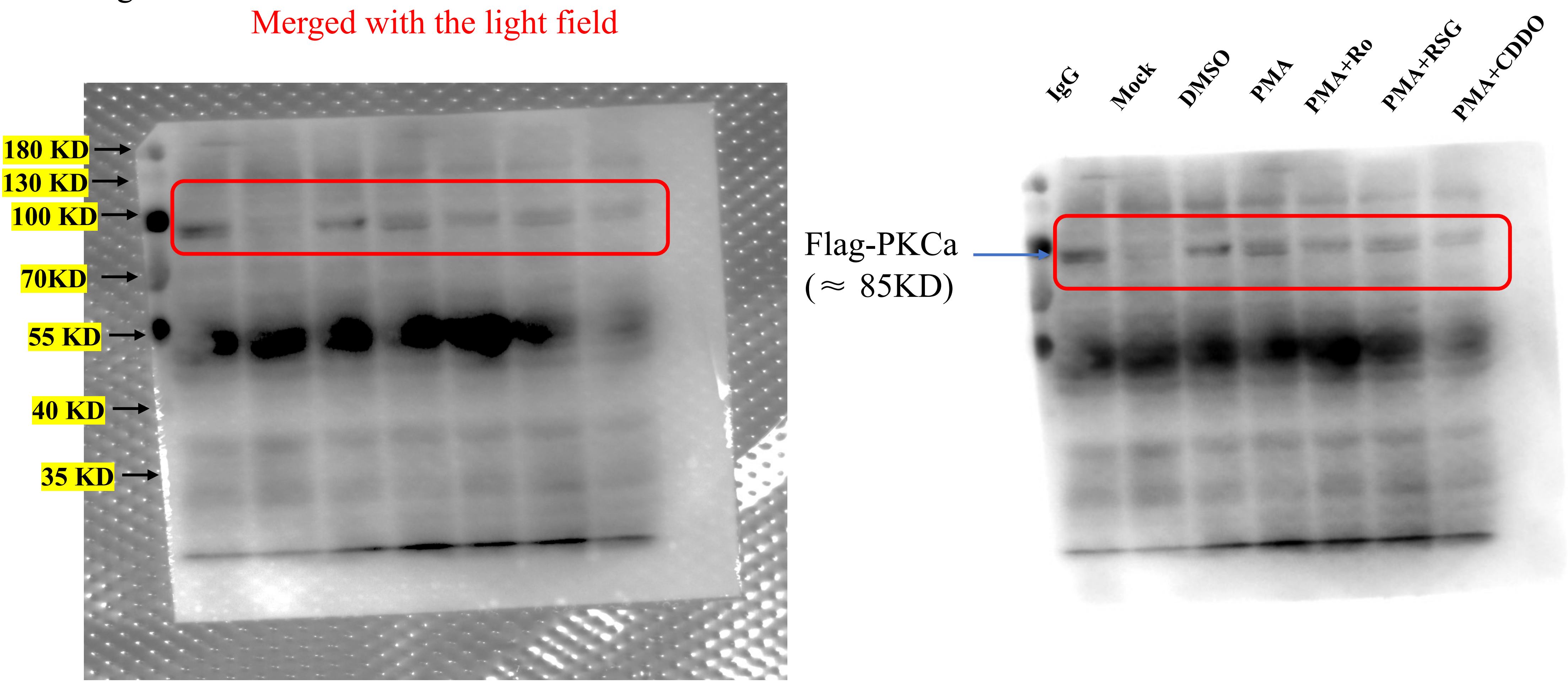

Antibody: PKCa antibody (Cell signaling technology #2056)

Figure 2a

Full unedited gel for Figure 2a

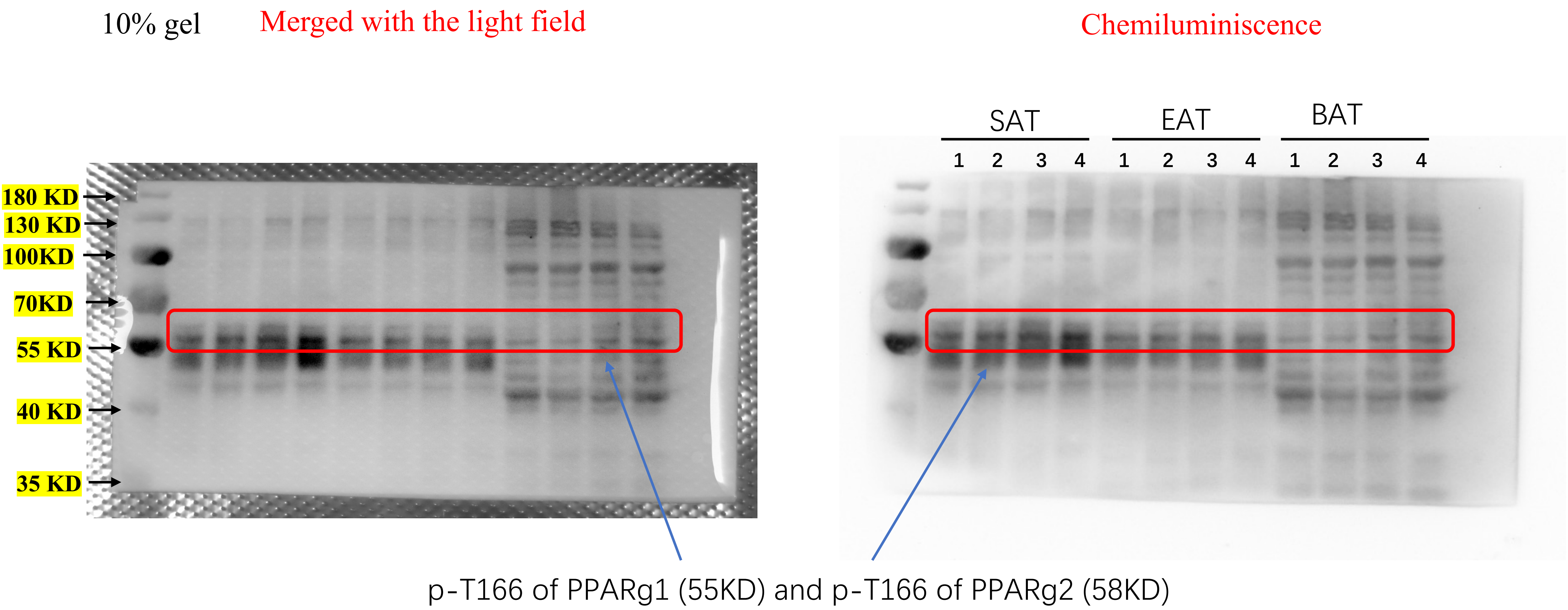

Antibody: T166 phosphorylation antibody generated by our lab

Full unedited gel for Figure 2a

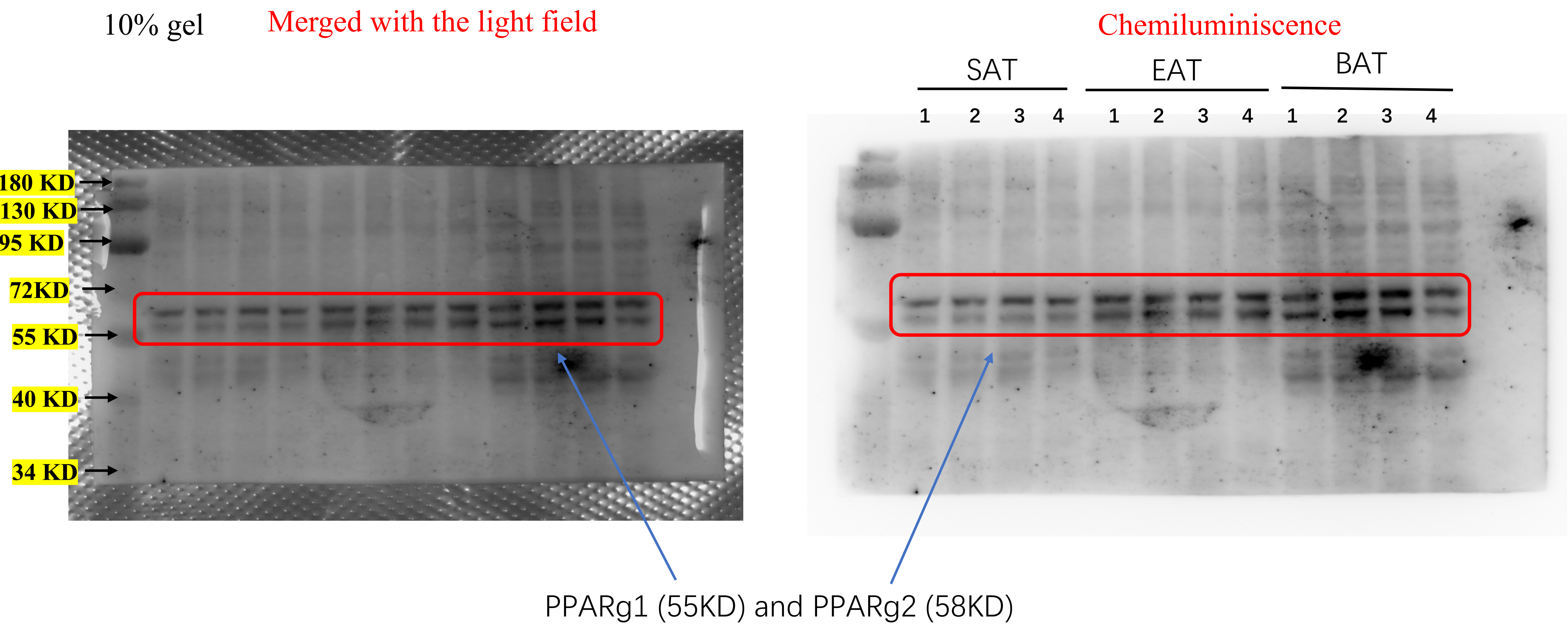

Antibody: PPARg antibody (Cell signaling technology #81b8)

Full unedited gel for Figure 2a

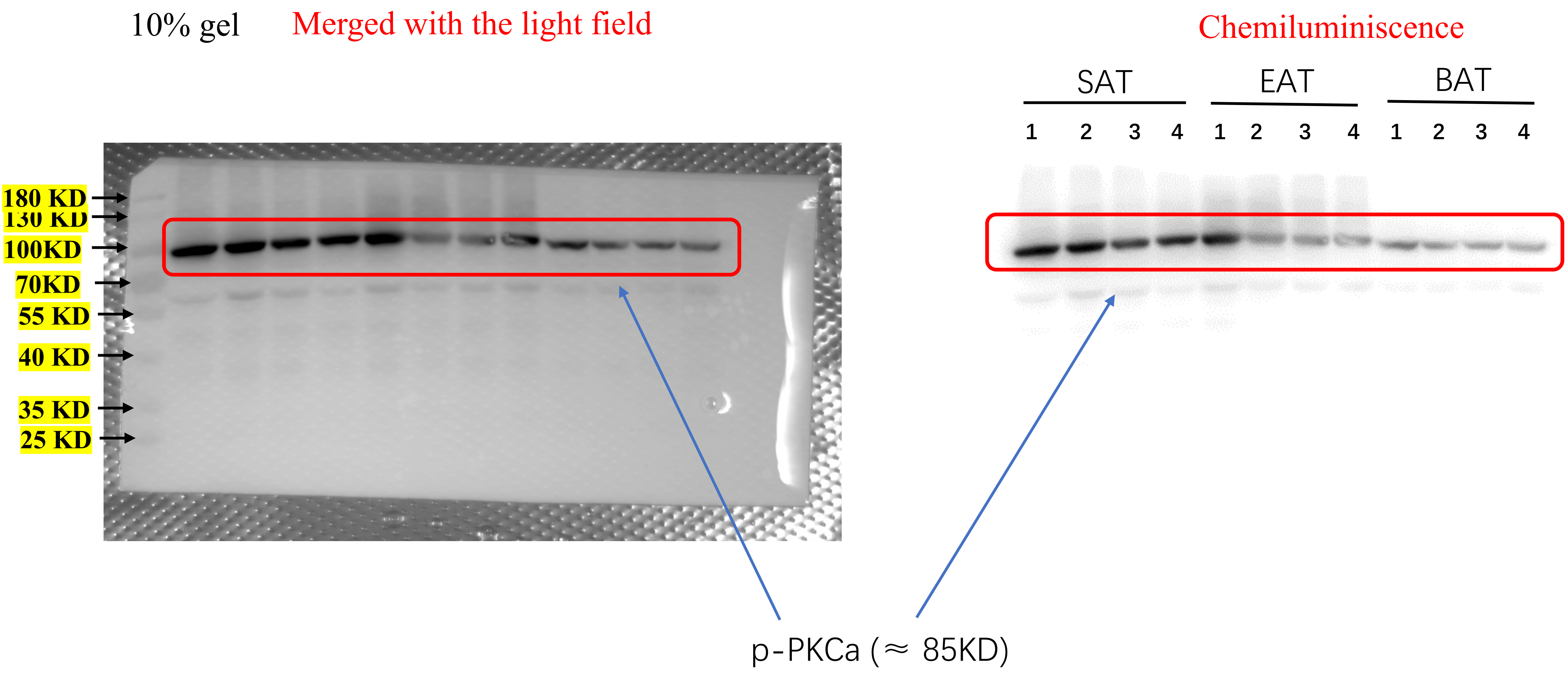

Antibody: p-PKCa antibody (Abcam ab32502)

Full unedited gel for Figure 2a

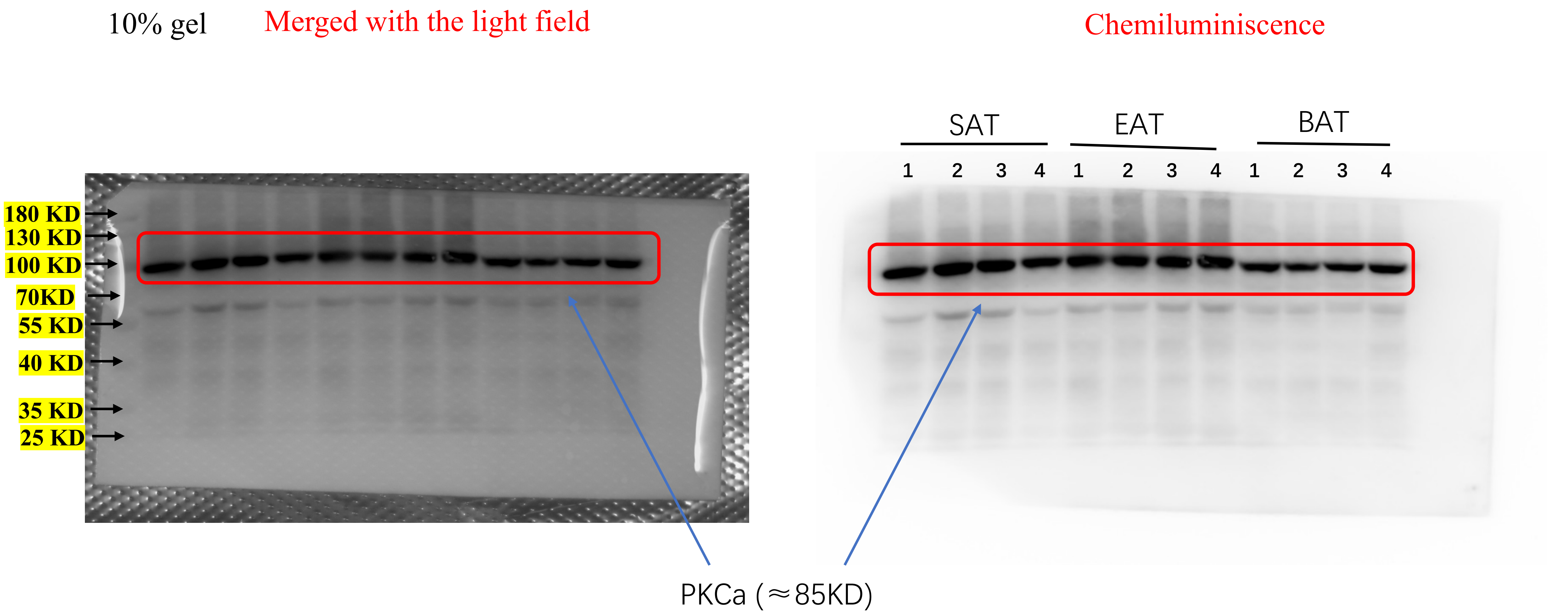

Antibody: PKCa antibody (Cell signaling technology #2056)

Full unedited gel for Figure 2a

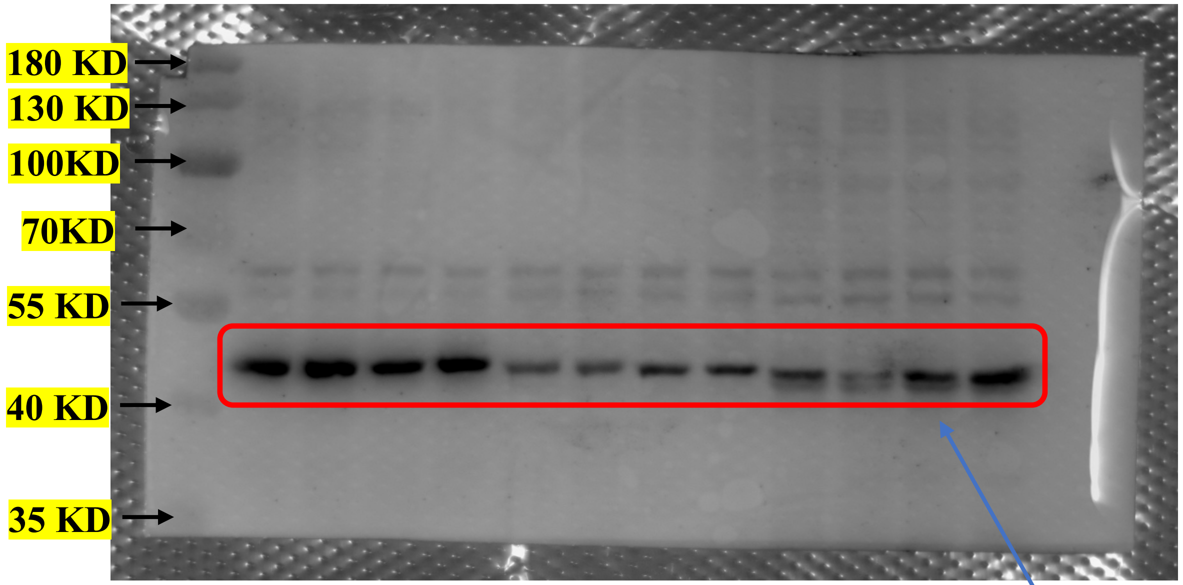

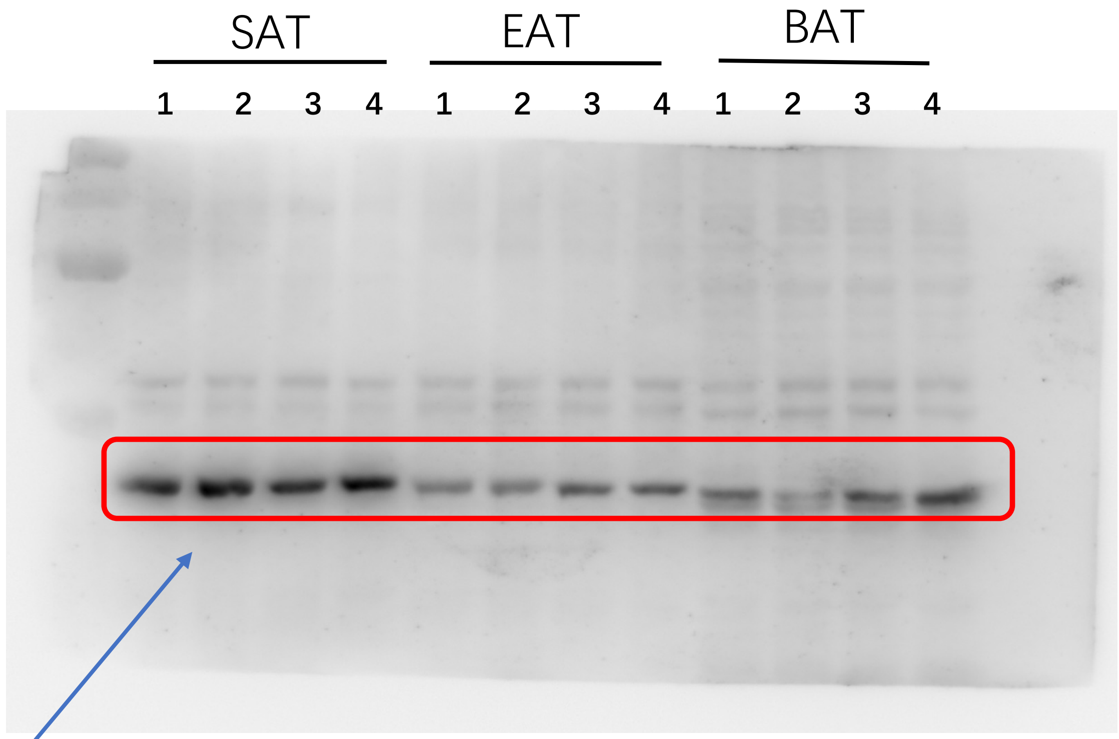

Antibody: b-Actin (Genscript A00702)

Figure 2d

Full unedited gel for Figure 2d

10% gel

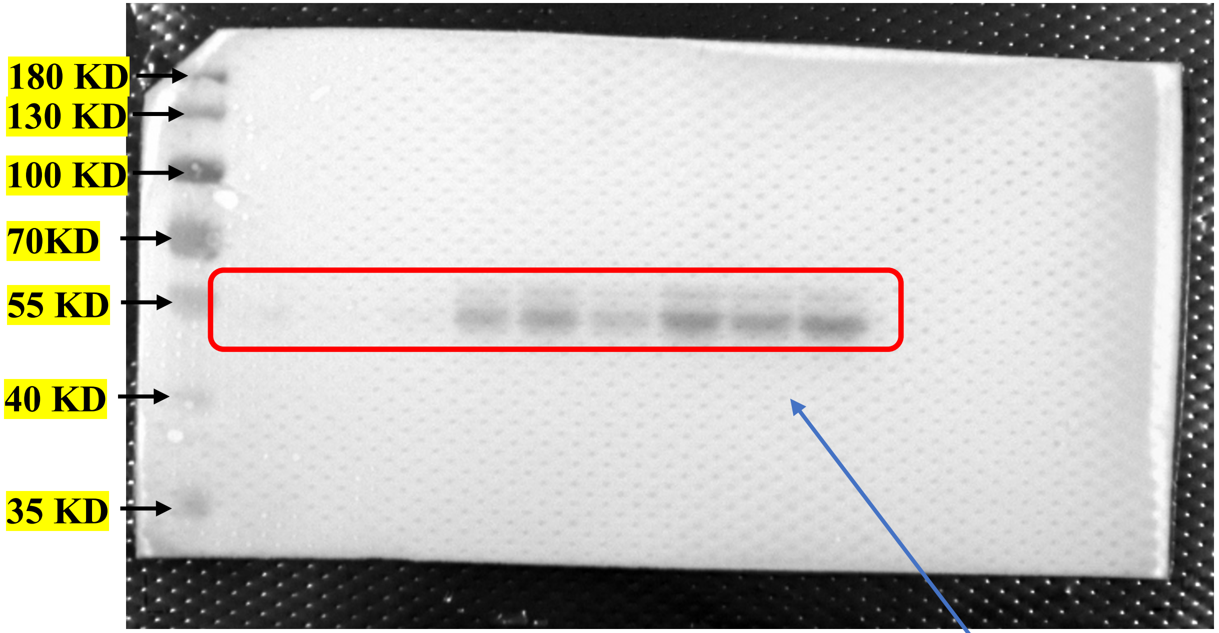

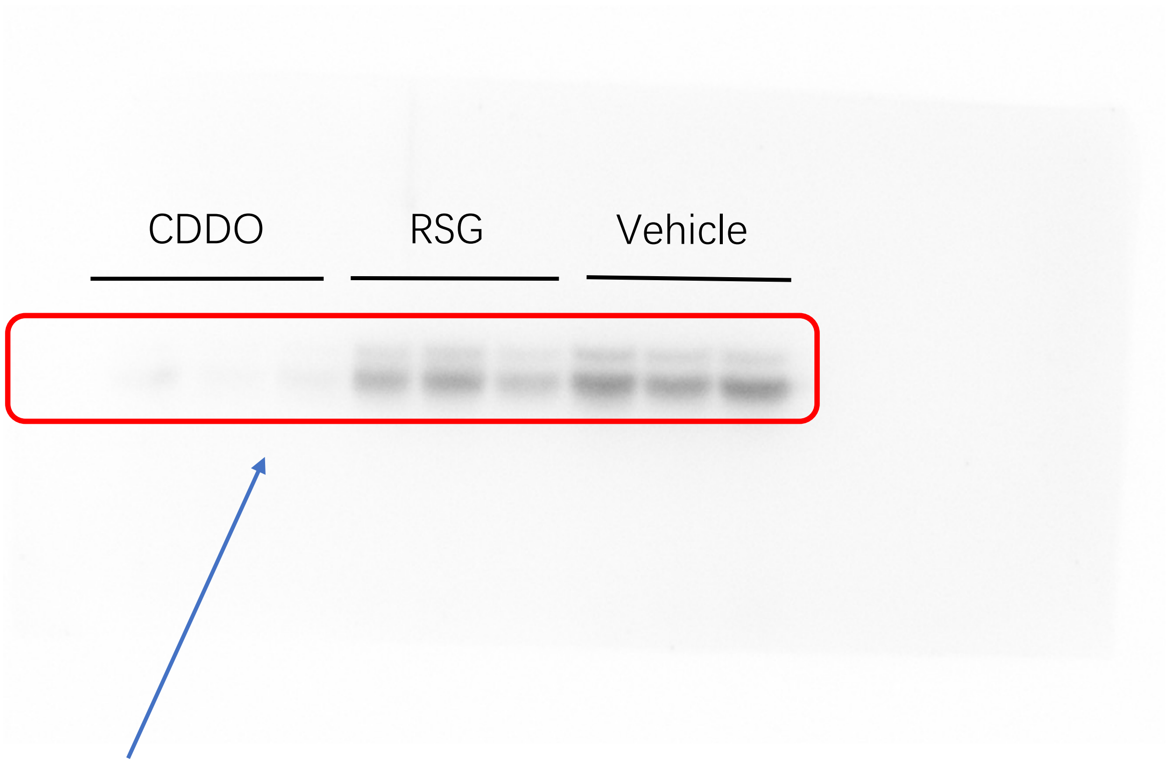

p-T166 of PPARg1 (55KD) and p-T166 of PPARg2 (58KD)

Antibody: T166 phosphorylation antibody generated by our lab

Full unedited gel for Figure 2d

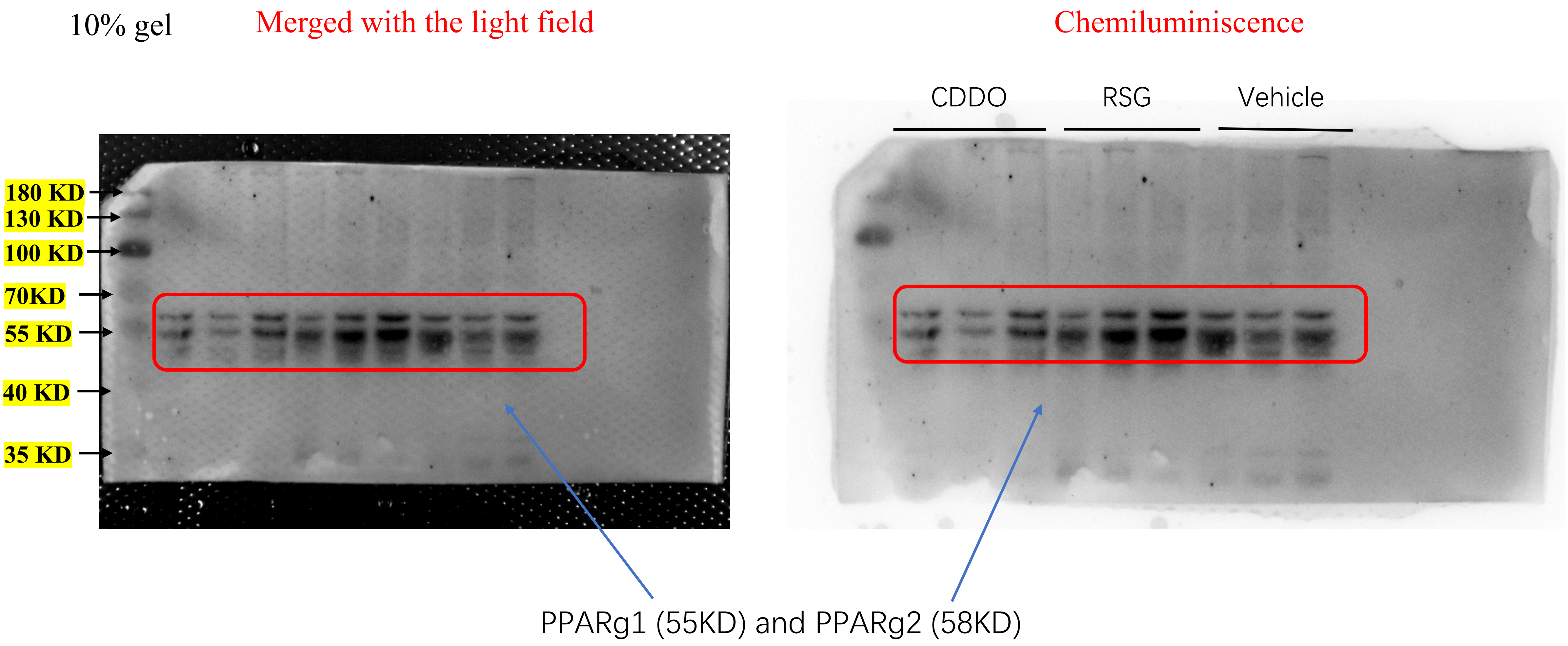

Antibody: PPARg antibody (Cell signaling technology #81b8)

Full unedited gel for Figure 2d

10% gel Merged with the light field Chemiluminiscence

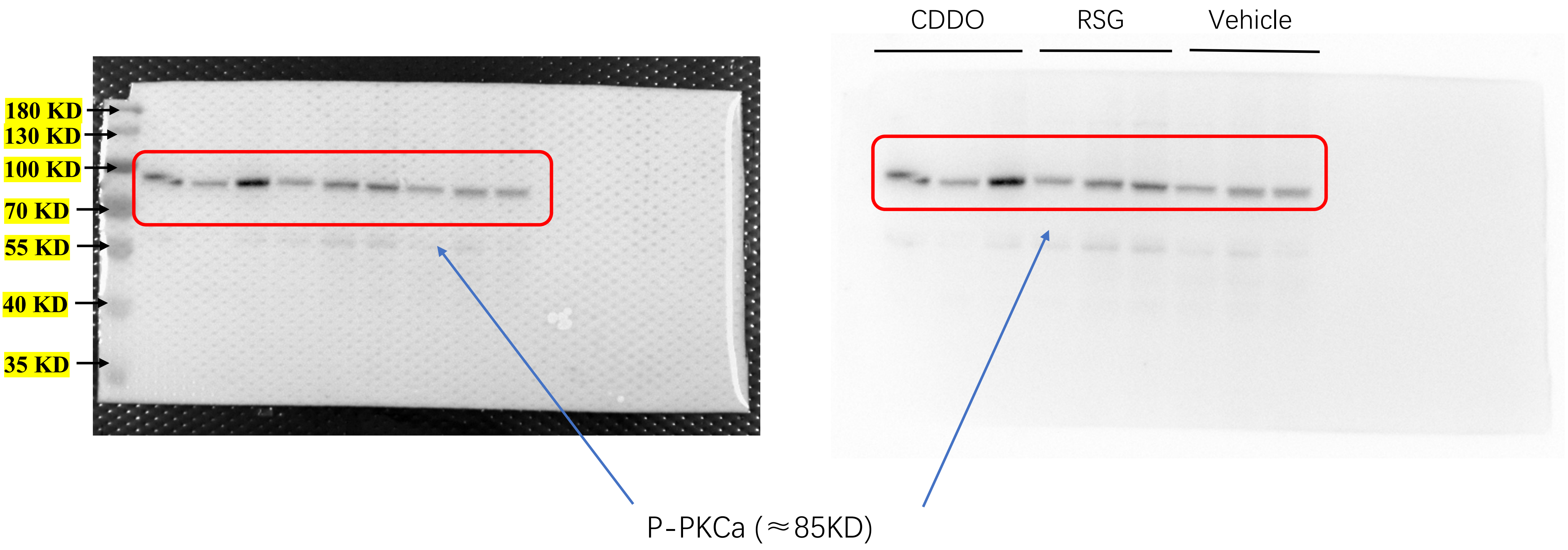

Antibody: p-PKCa antibody (Abcam ab32502)

Full unedited gel for Figure 2d

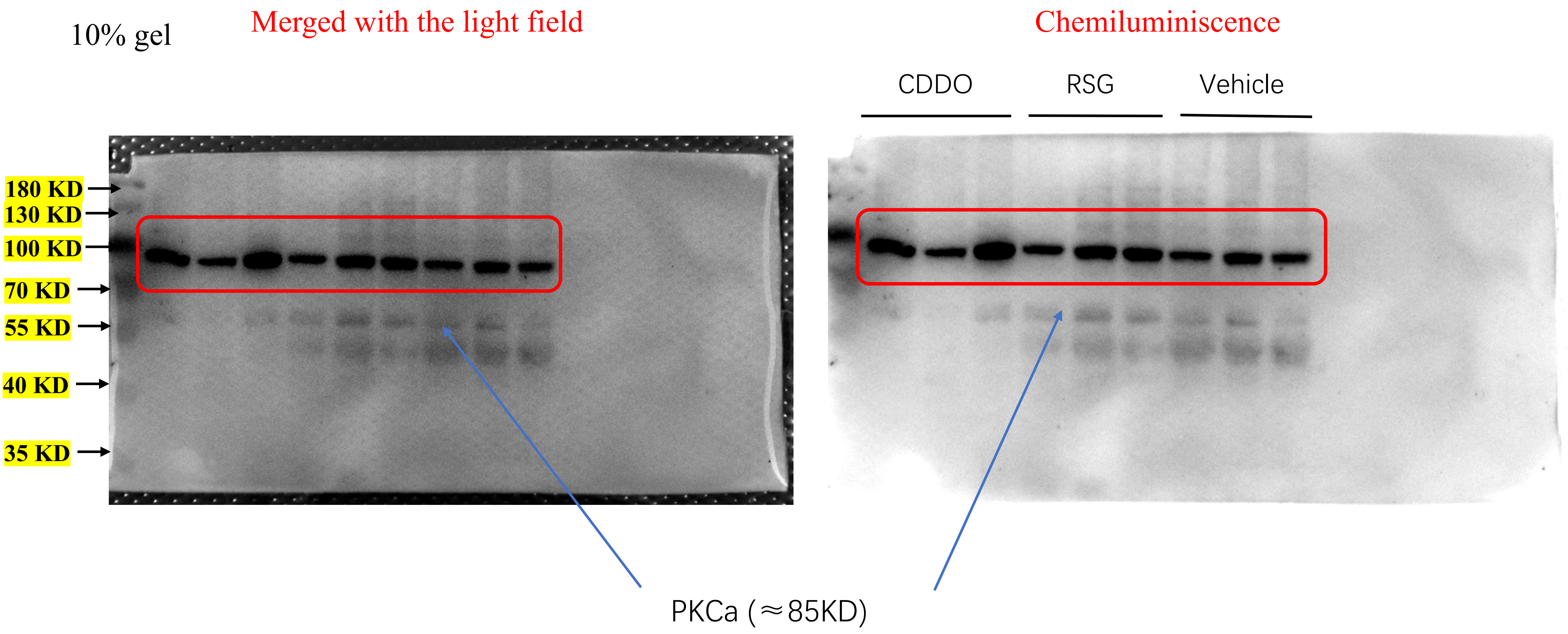

Antibody: PKCa antibody (Cell signaling technology #2056)

Full unedited gel for Figure 2d

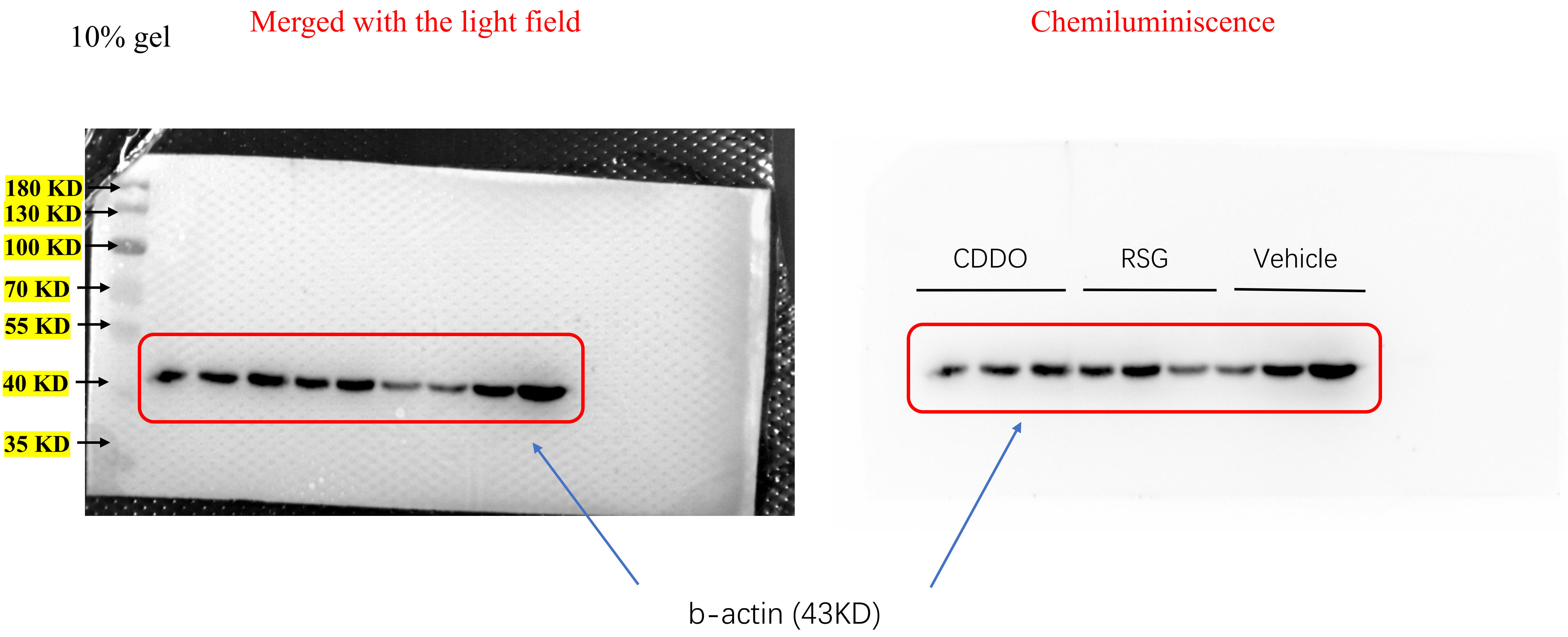

Antibody: b-Actin (Genscript A00702)

Figure 2g

Full unedited gel for Figure 2g

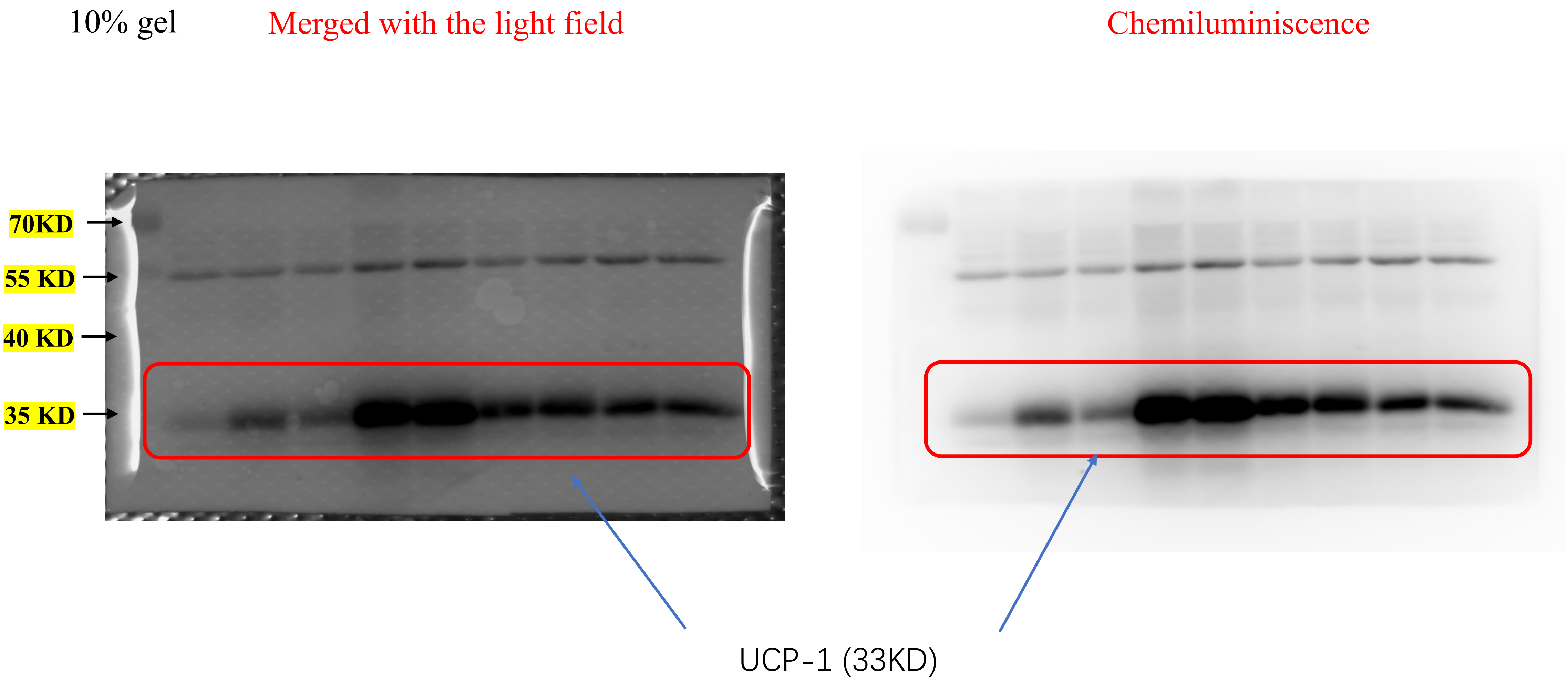

Antibody: UCP1 antibody (Abcam ab209483)

Full unedited gel for Figure 2g

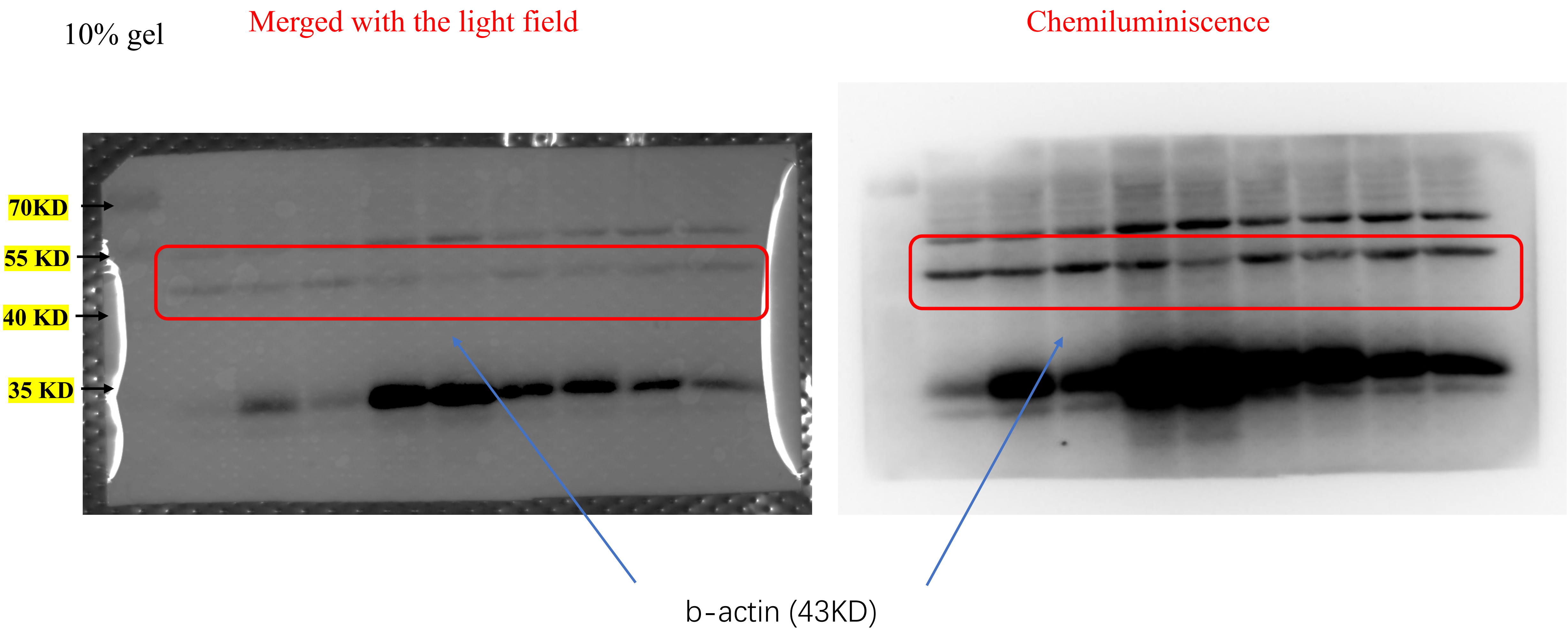

Antibody: b-Actin (Genscript A00702)

Figure 4f

Full unedited gel for Figure 4f

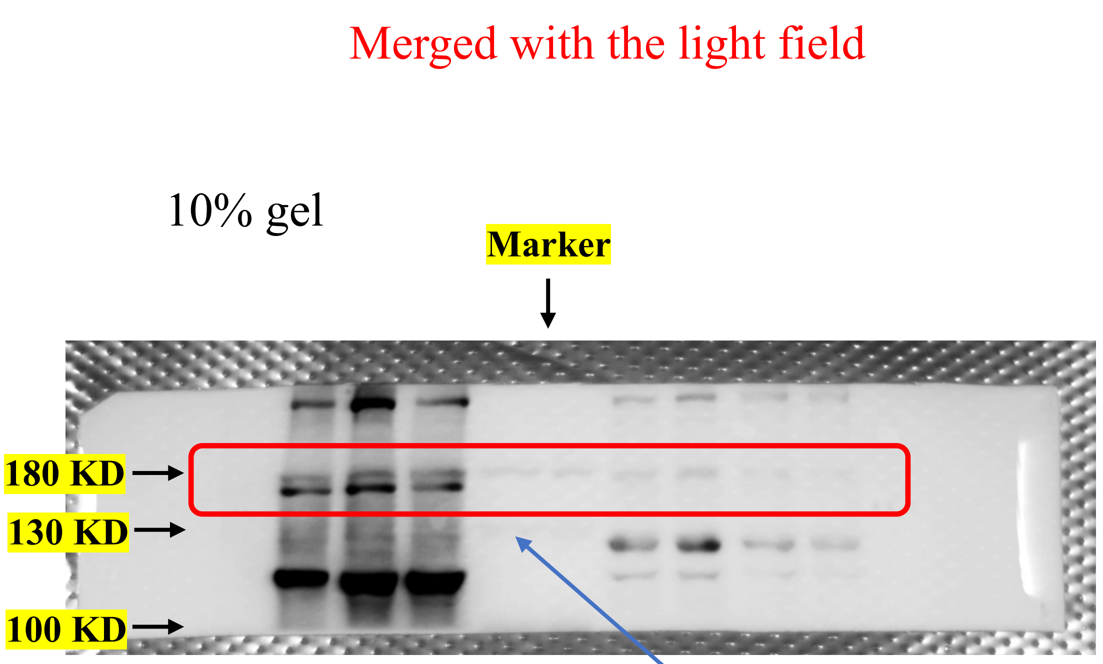

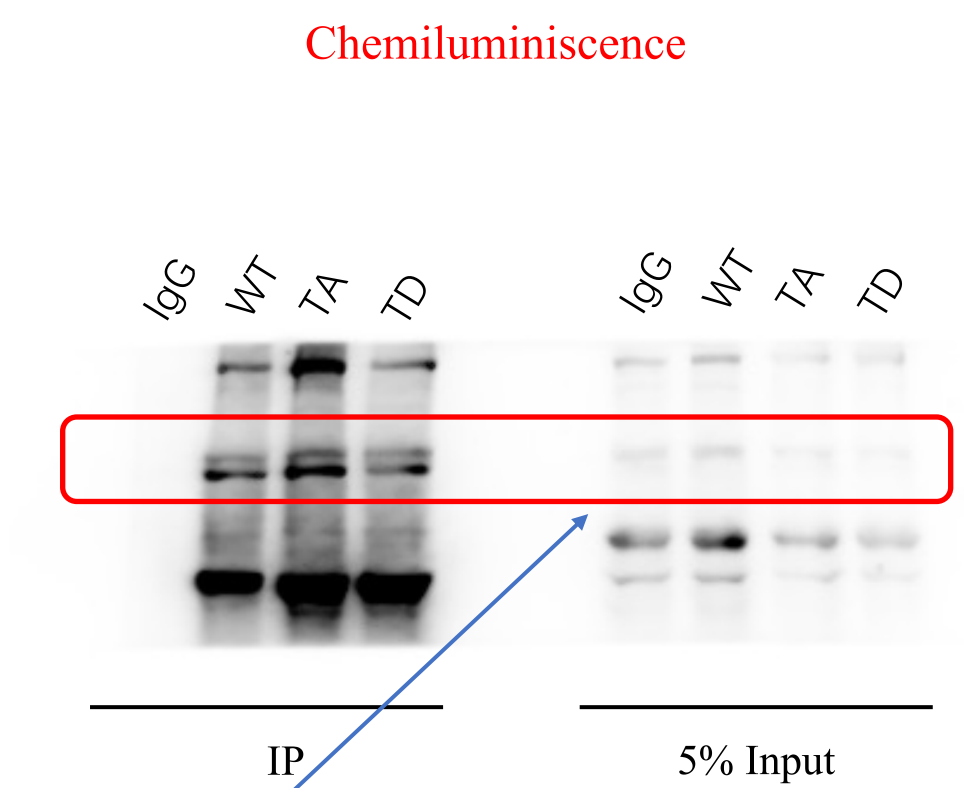

endogenous PRDM16 (≈150KD)

Antibody: PRDM16 antibody (Abcam ab106410)

Full unedited gel for Figure 4f

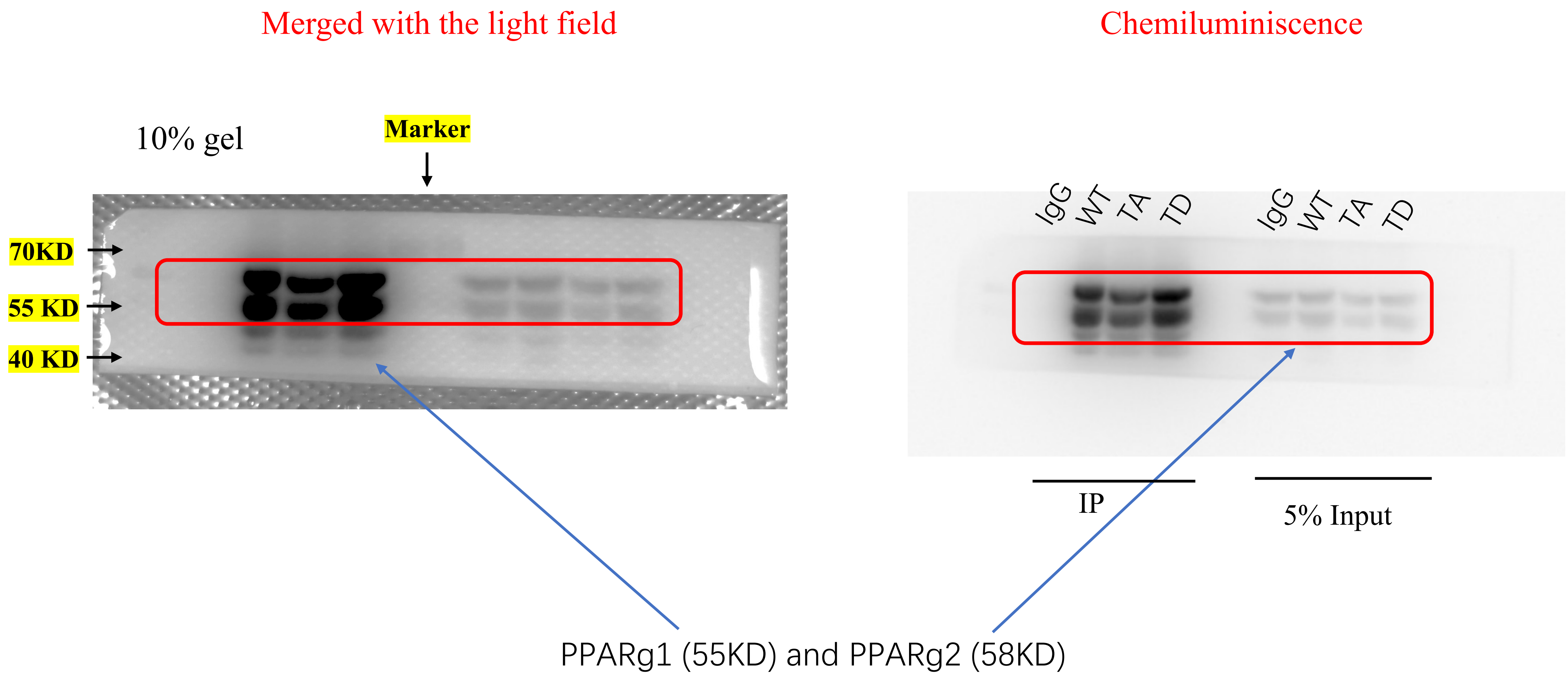

Antibody: PPARg antibody (Cell signaling technology #81b8)

Figure 4g

Full unedited gel for Figure 4g

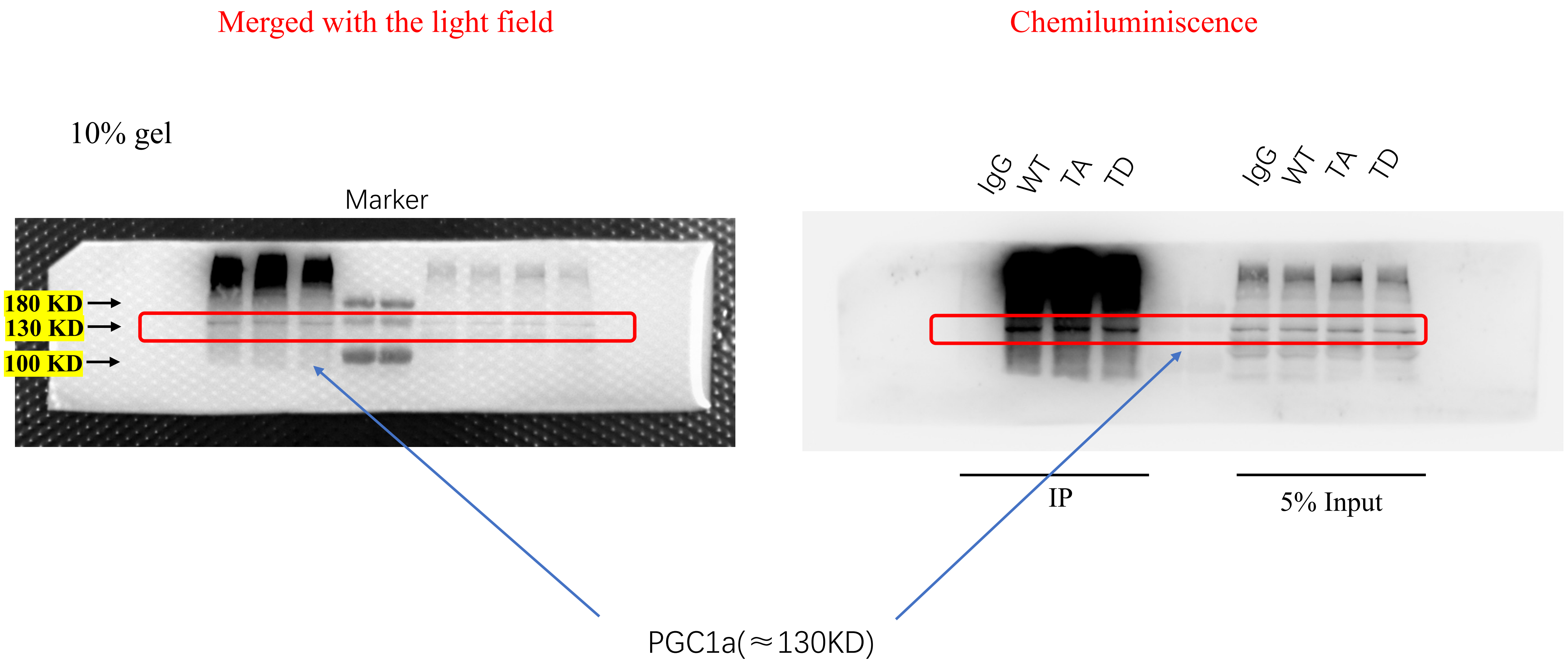

Antibody: PGC1a antibody (Cell signaling technology #2178)

Full unedited gel for Figure 4g

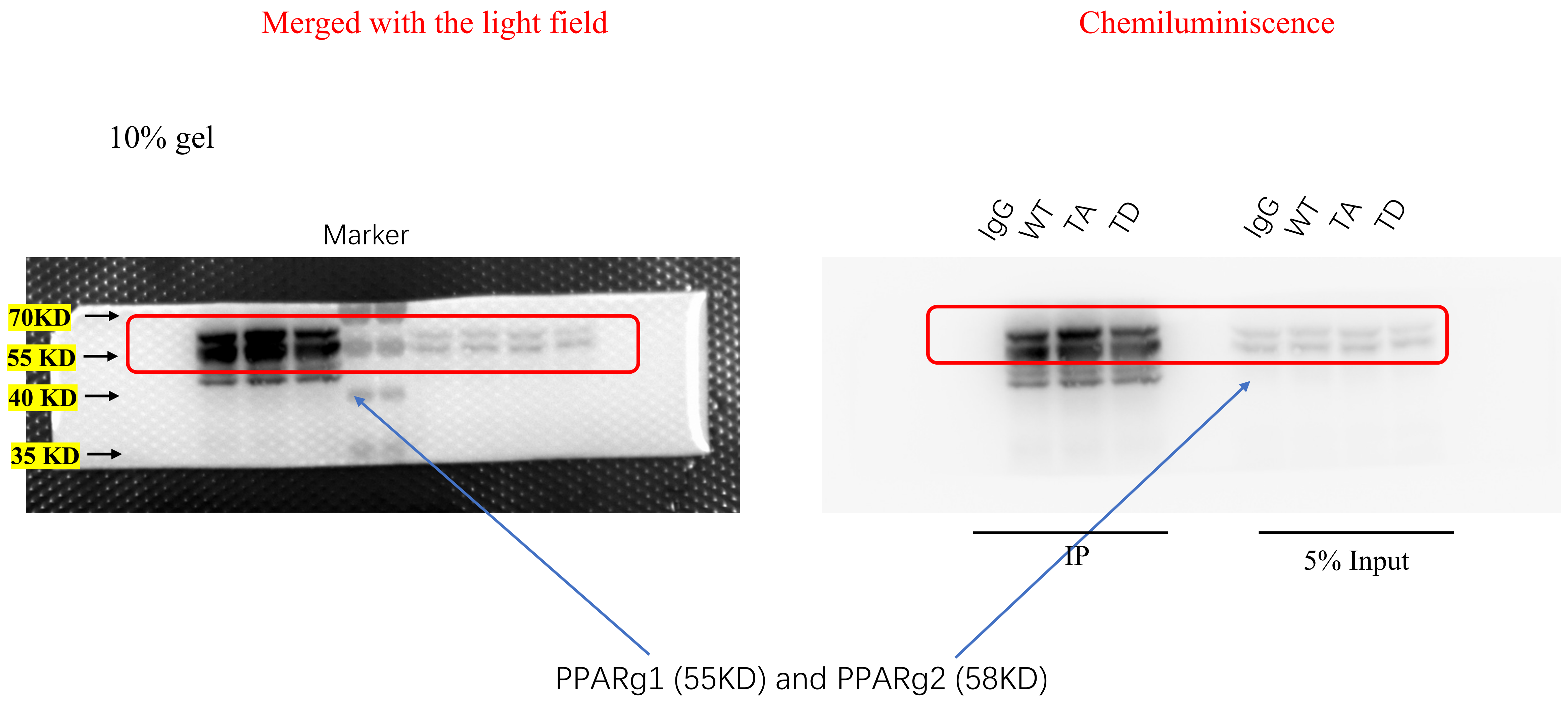

Antibody: PPARg antibody (Cell signaling technology #81b8)

Figure S1b

Full unedited gel for Figure S1b

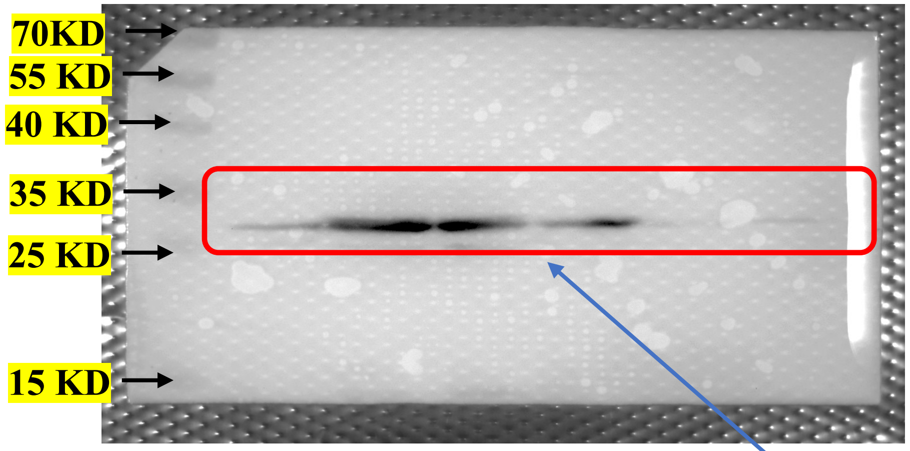

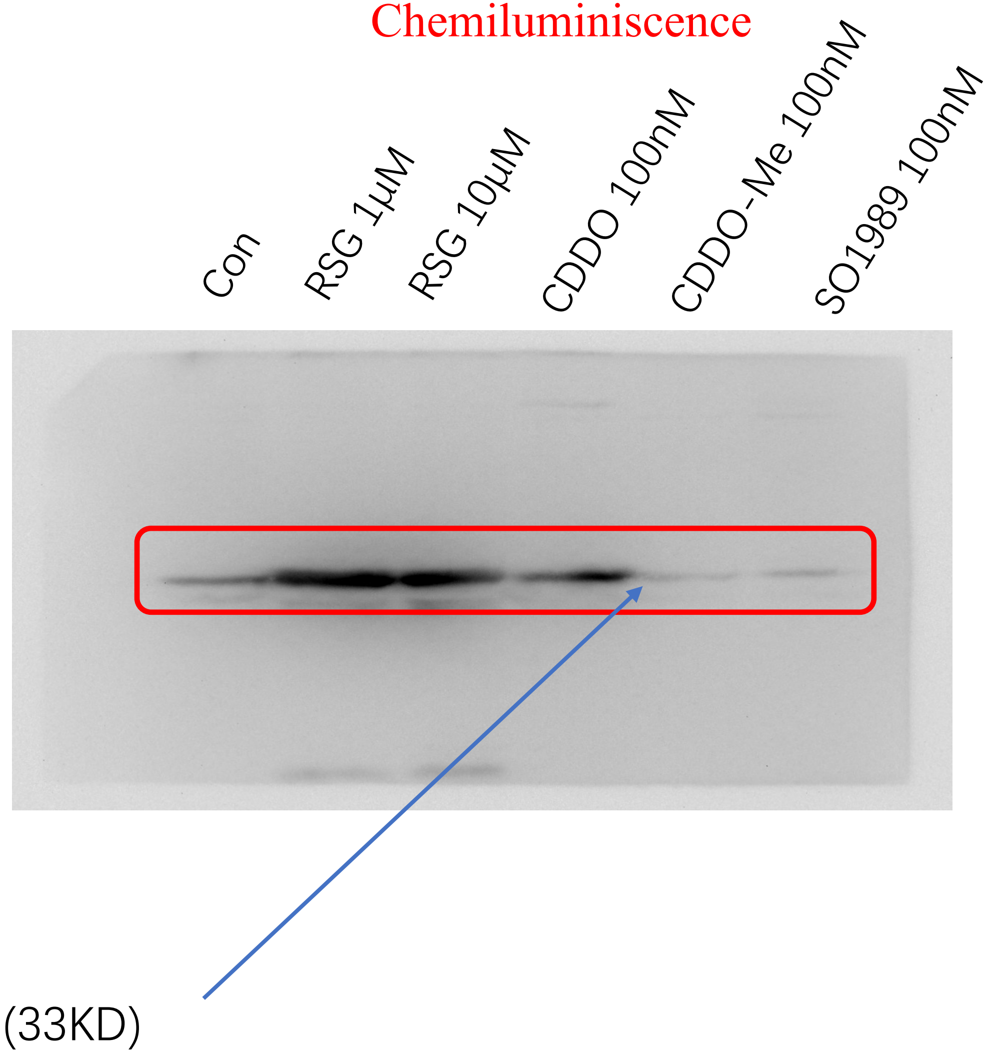

Antibody: UCP1 antibody (Abcam ab209483)

Full unedited gel for Figure S1b

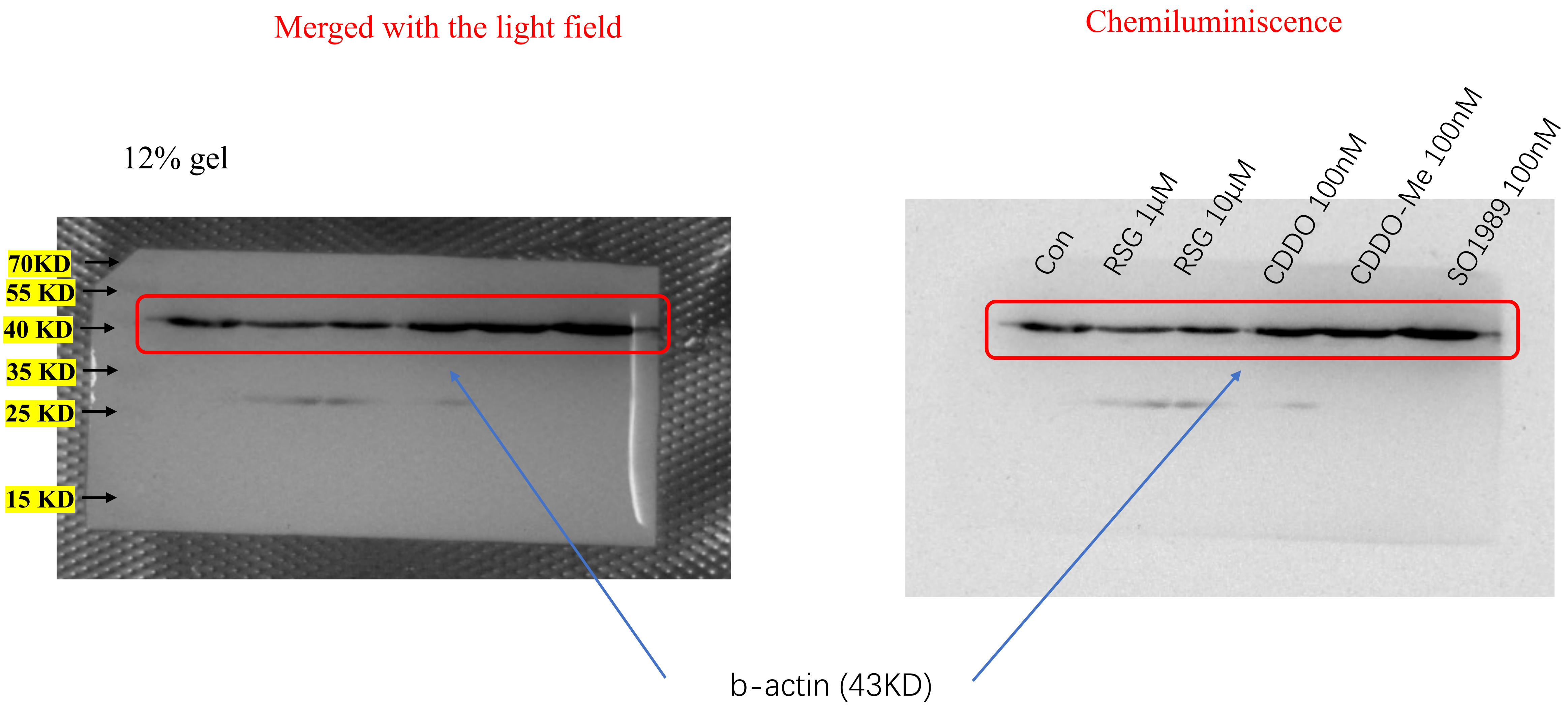

Antibody: b-Actin (Genscript A00702)

Figure S1d

Full unedited gel for Figure S1d

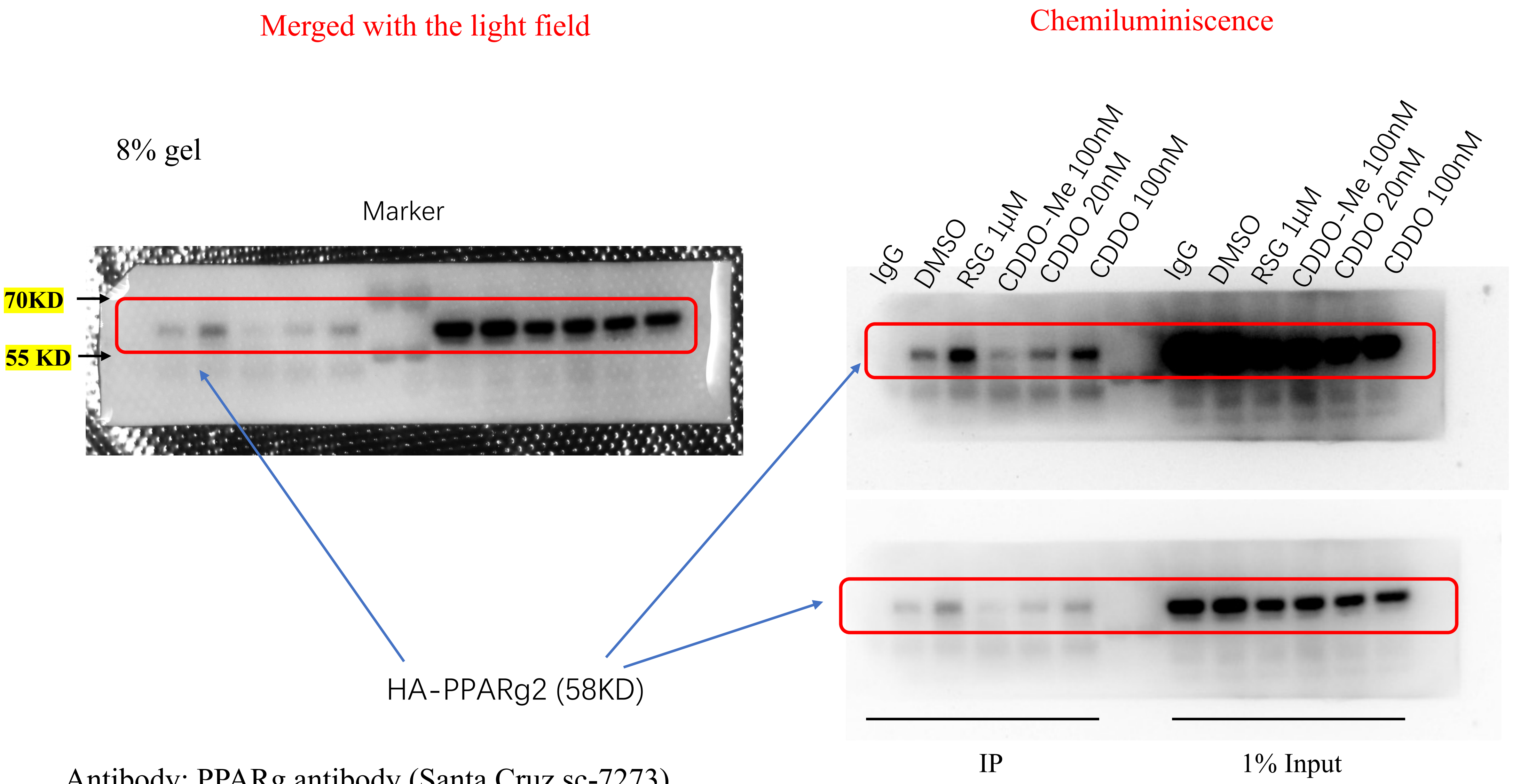

Antibody: PPARg antibody (Santa Cruz sc-7273)

Full unedited gel for Figure S1d

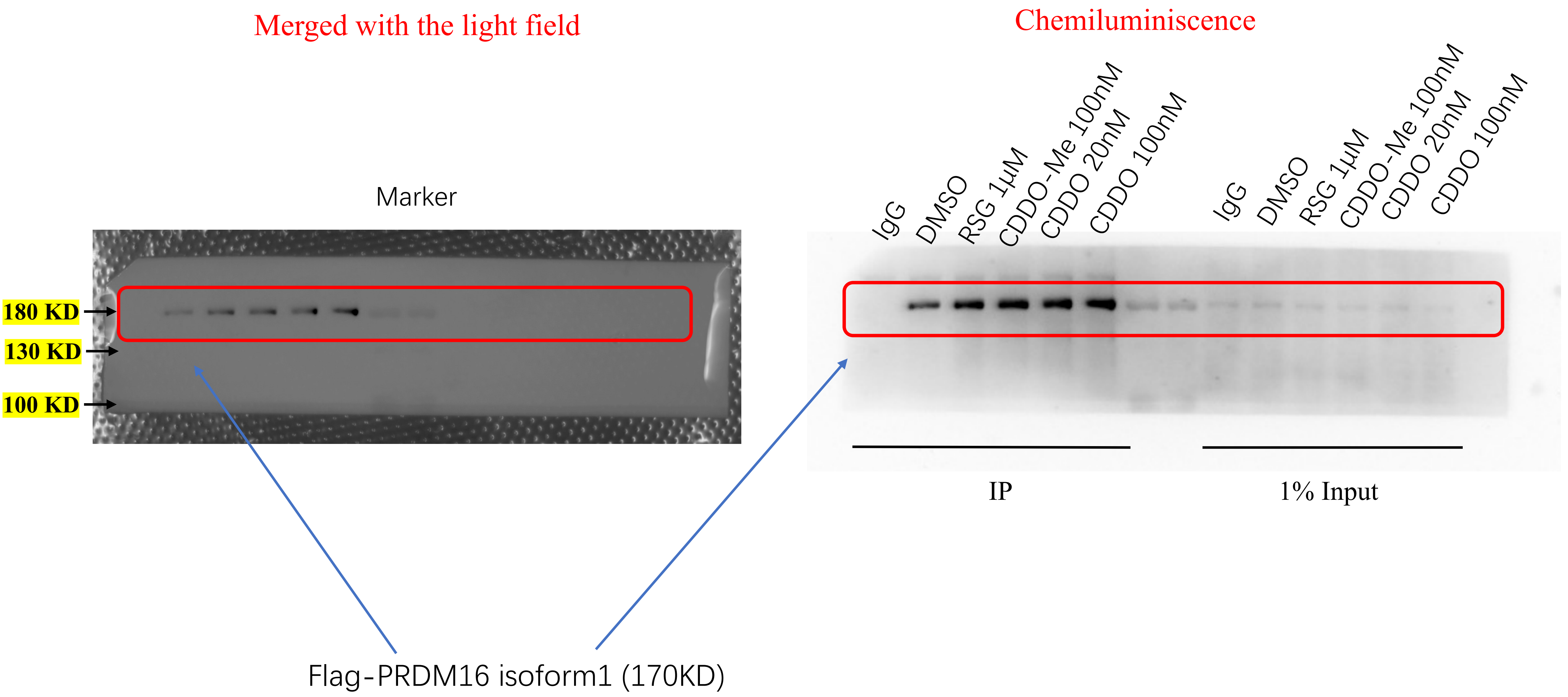

Note: PRDM16 Isoform 1 is the largest isoform of PRDM16

Antibody: Flag antibody (Proteintech Cat No. 20543-1-AP)

Figure S3a

Full unedited gel for Figure S3a

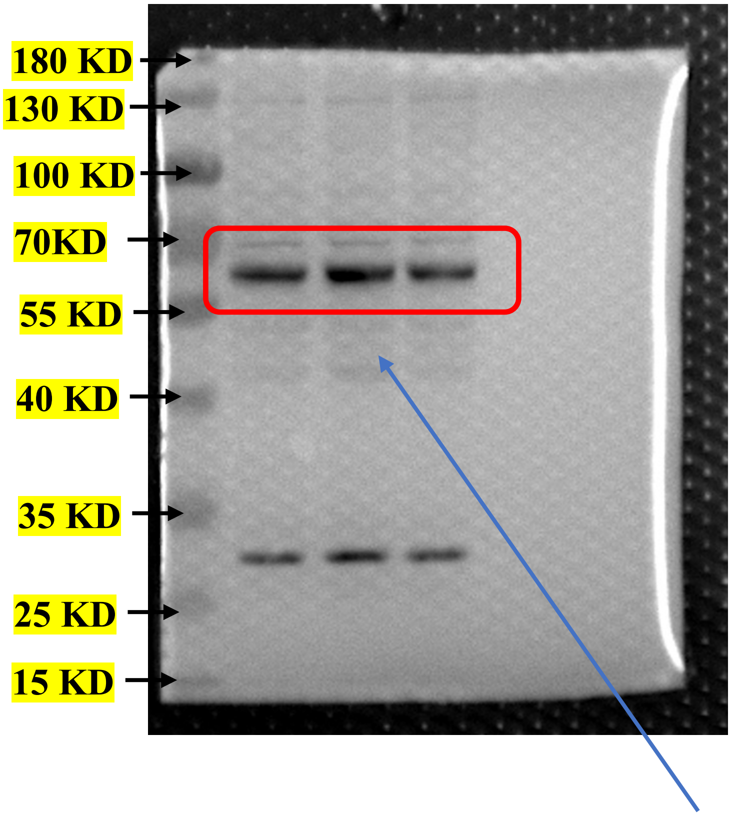

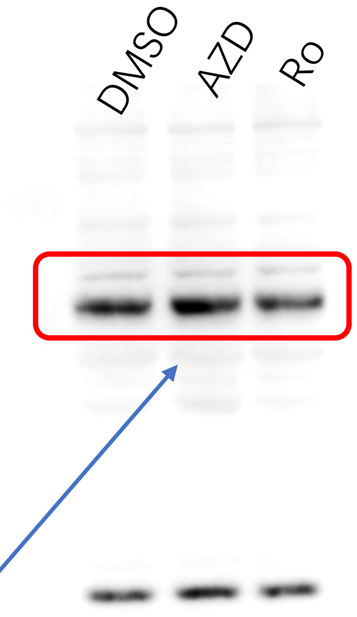

p-T166 (58KD)

Antibody: T166 phosphorylation antibody generated by our lab

Full unedited gel for Figure S3a

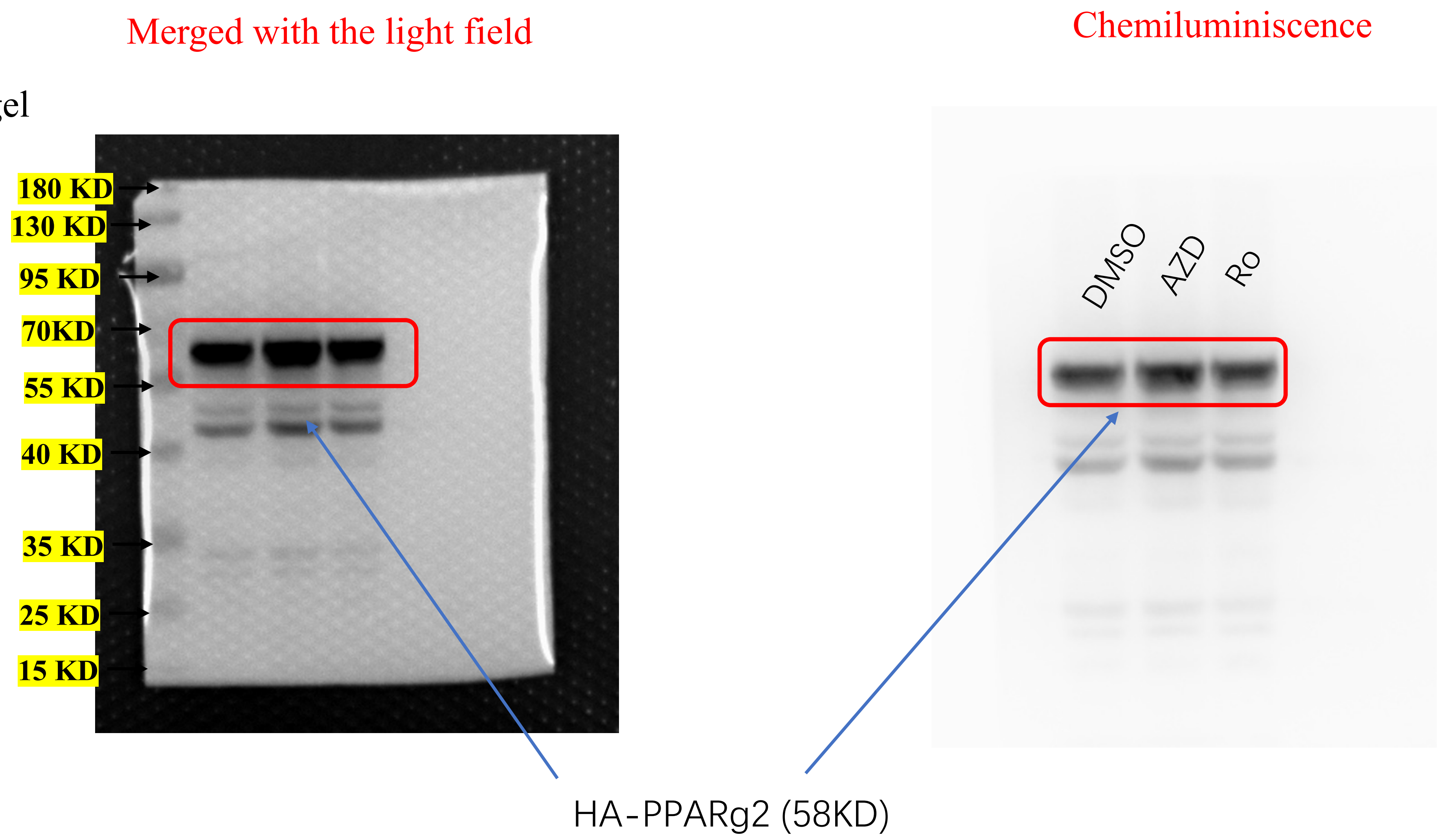

Antibody: PPARg antibody (Cell signaling technology #81b8)

Full unedited gel for Figure S3a

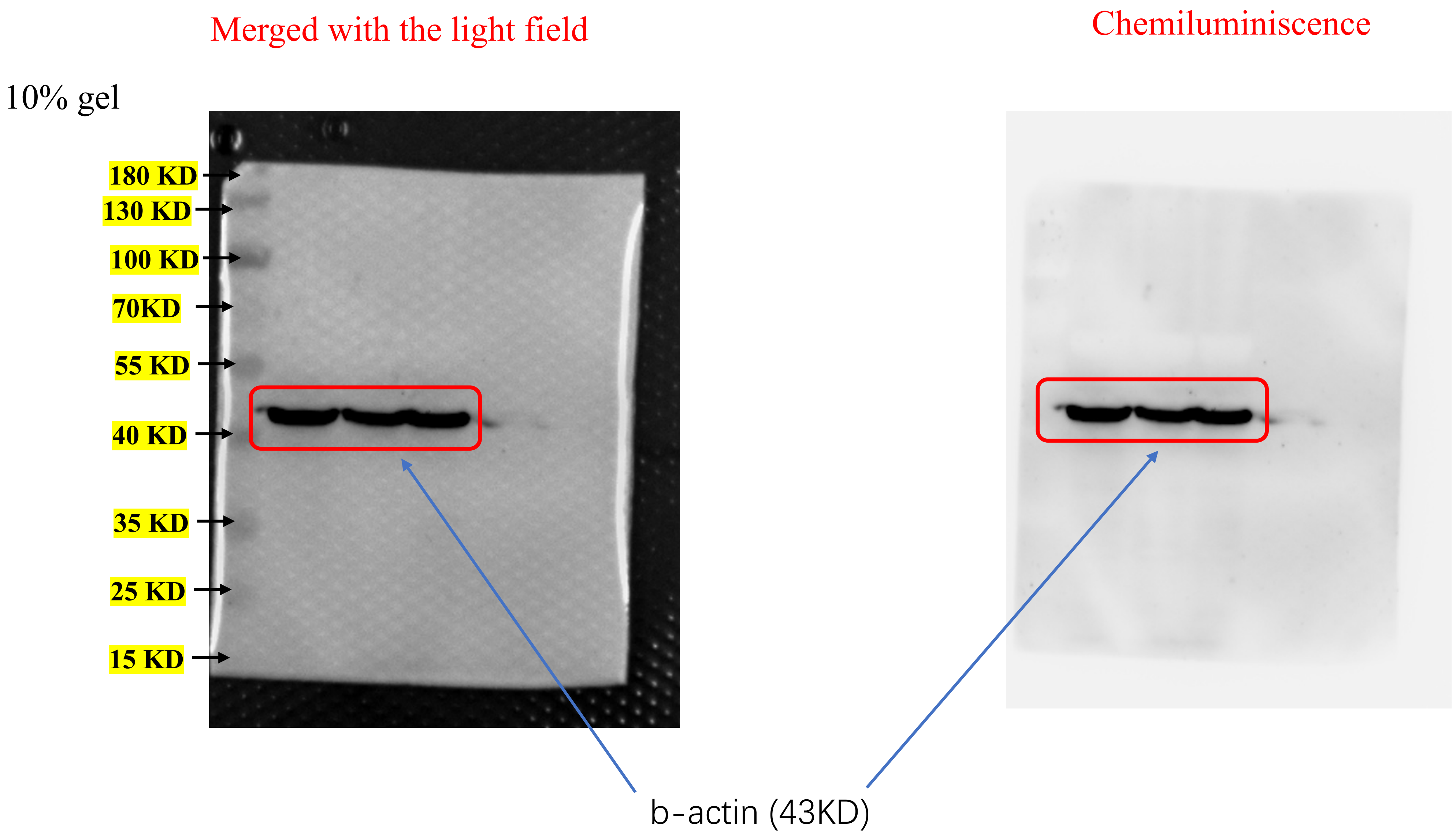

Antibody: b-Actin (Genscript A00702)

Figure S3c

Full unedited gel for Figure S3c

Chemiluminiscence

10% gel

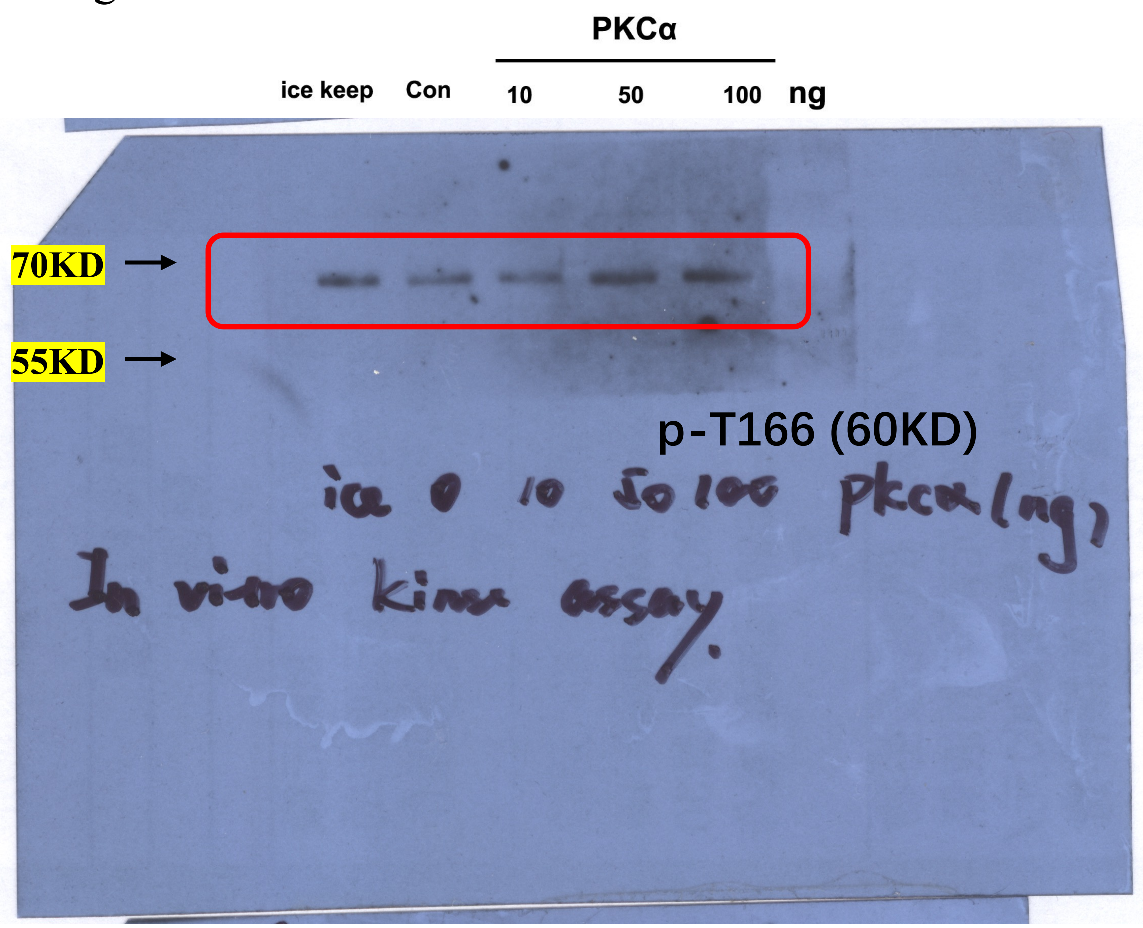

Antibody: T166 phosphorylation antibody generated by our lab

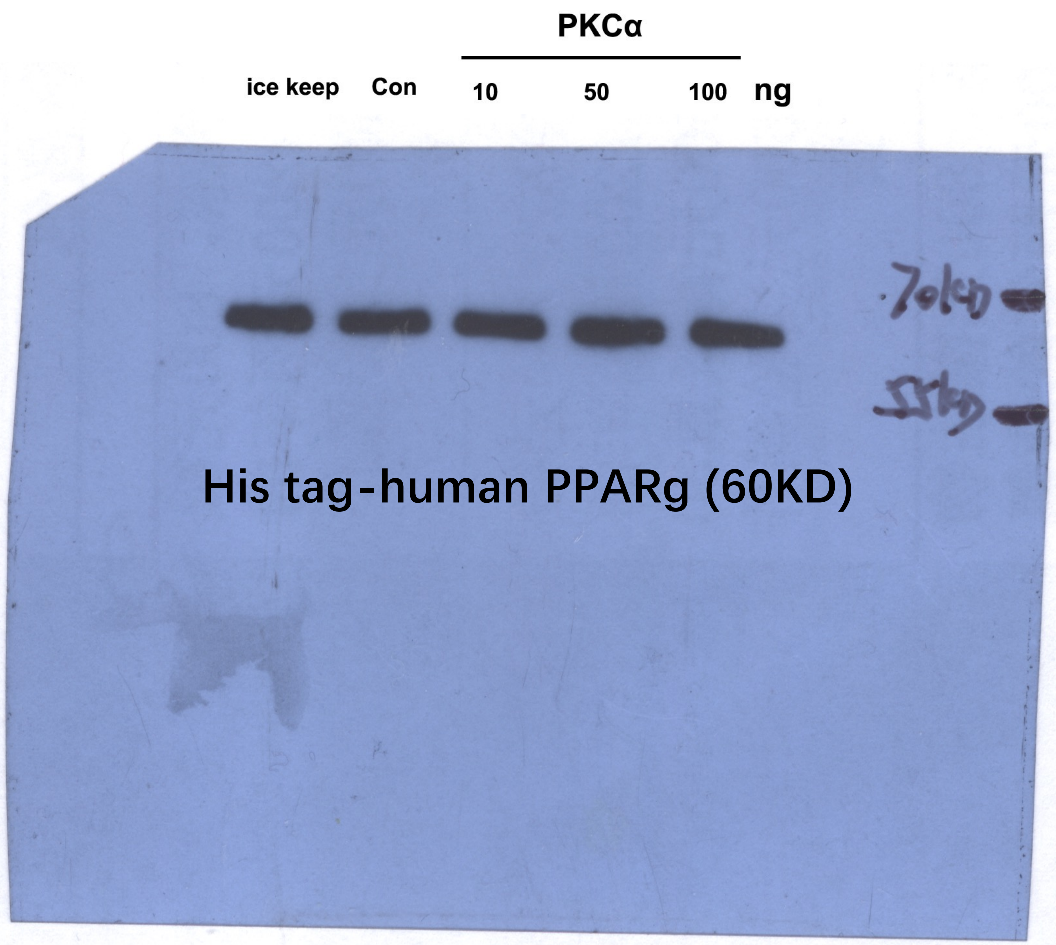

Antibody: PPARg antibody (Cell signaling technology #81b8)

Full unedited gel for Figure S3c

12% gel

Merged with the light field

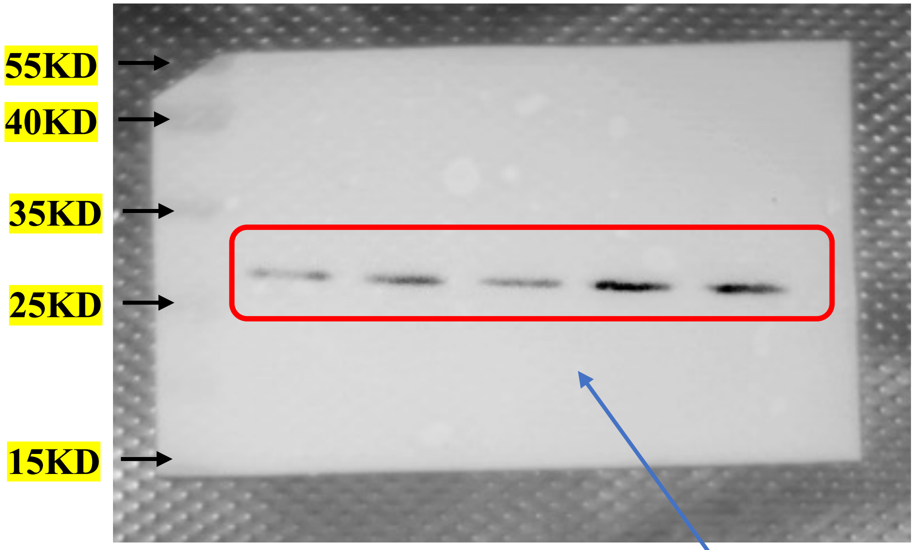

Chemiluminiscence

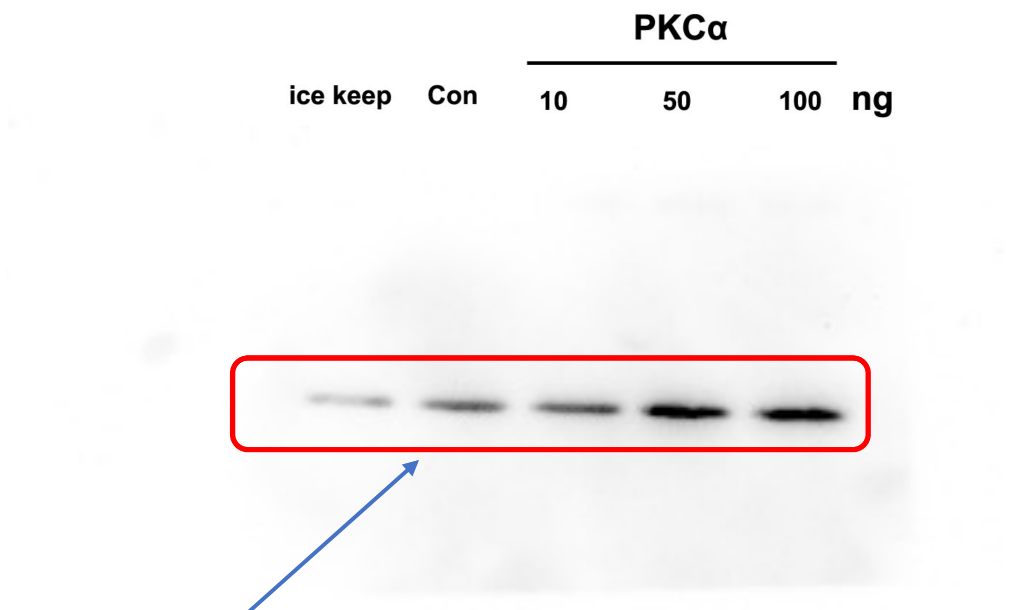

p-Histone H1 (32KD)

Antibody: p-histone H1 (05-1324, Millipore)

Figure S3e

Full unedited gel for Figure S3e

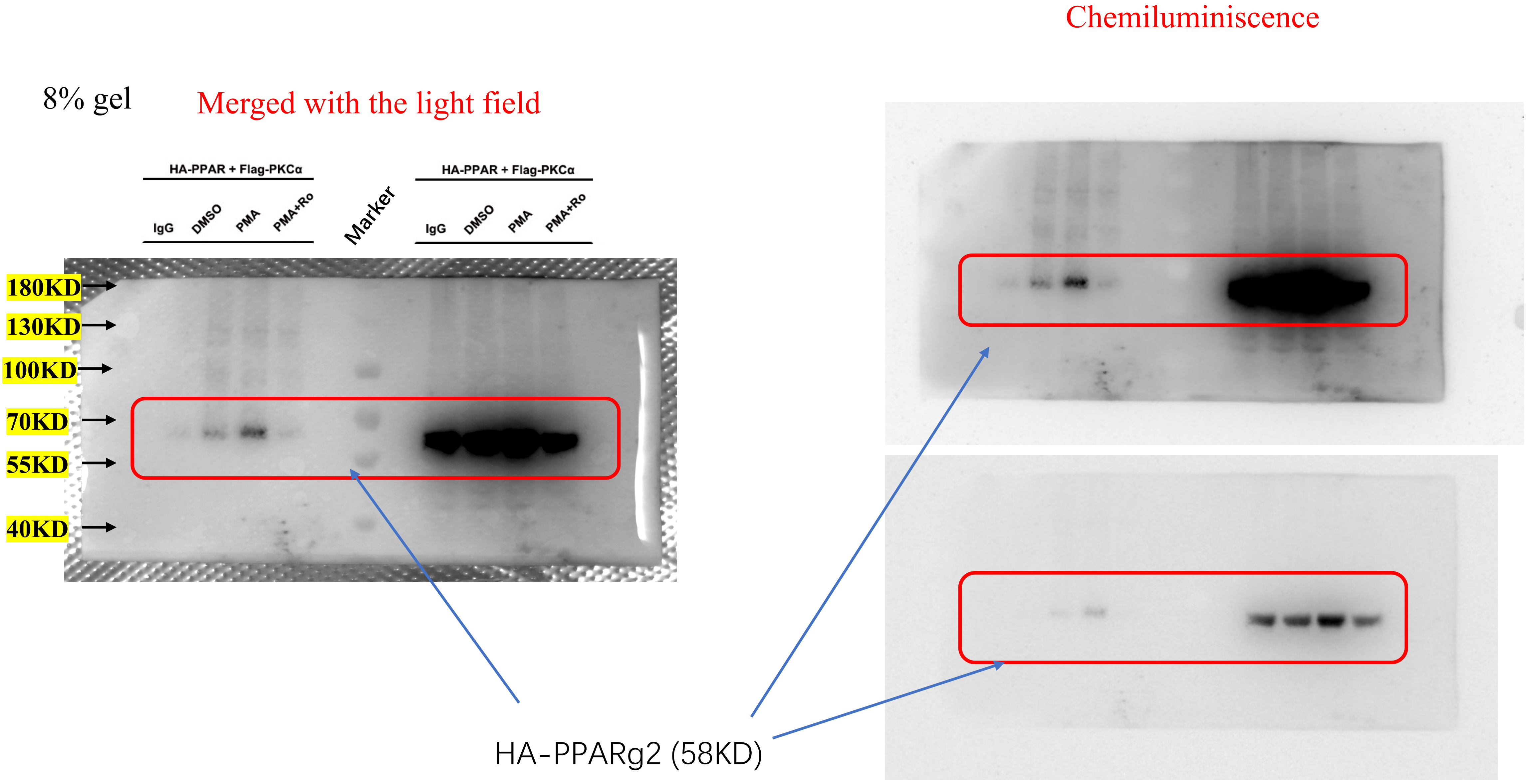

Antibody: PPARg antibody (Santa Cruz sc-7273)

Full unedited gel for Figure S3e

8% gel

Merged with the light field

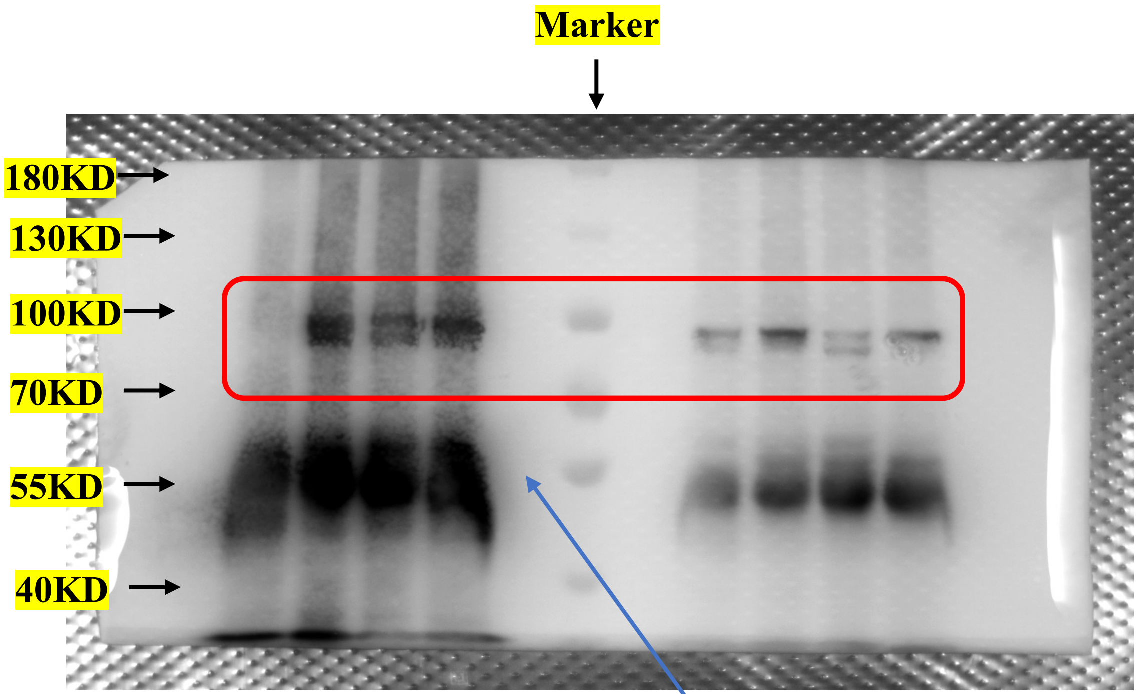

Chemiluminiscence

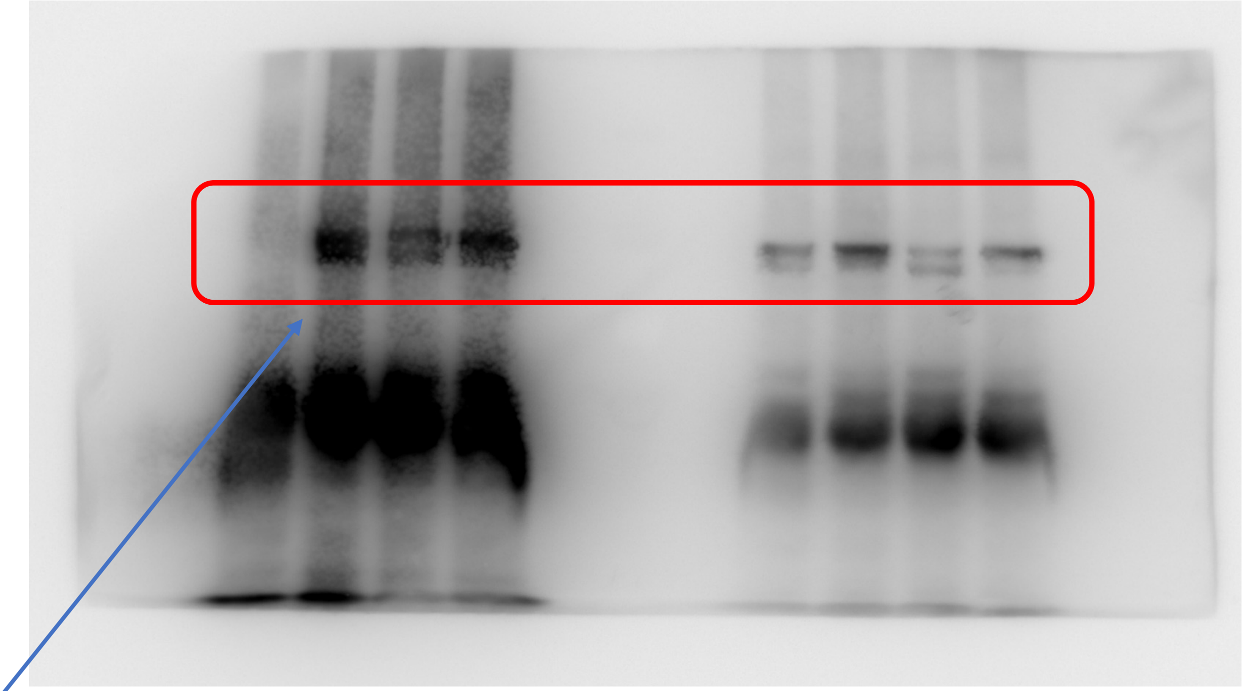

Flag-PKCa (≈ 85KD)

Antibody: Flag antibody (Proteintech Cat No. 20543-1-AP)

Figure S3f

Full unedited gel for Figure S3f

Chemiluminiscence

10% gel   Merged with the light field

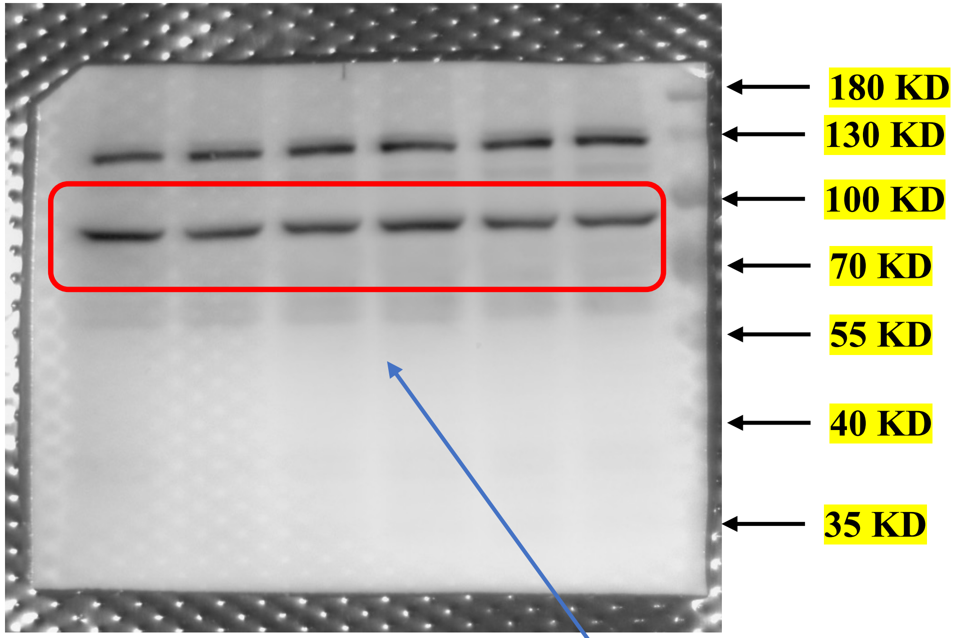

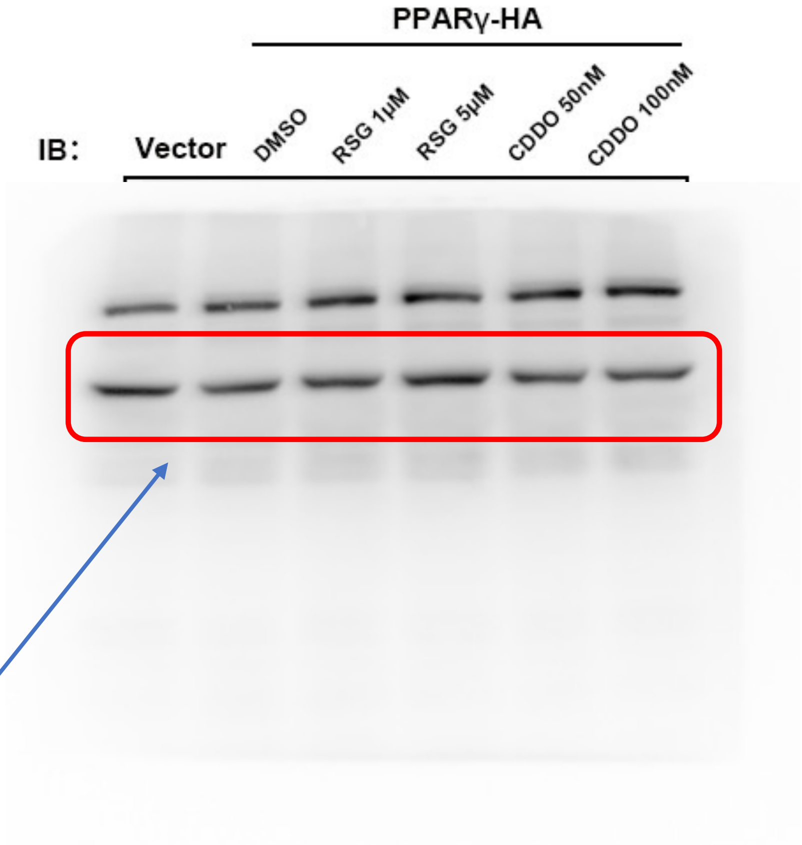

p-PKCa (≈ 85KD)

Antibody: p-PKCa antibody (Abcam ab32502)

Full unedited gel for Figure S3f

10% gel

Merged with the light field

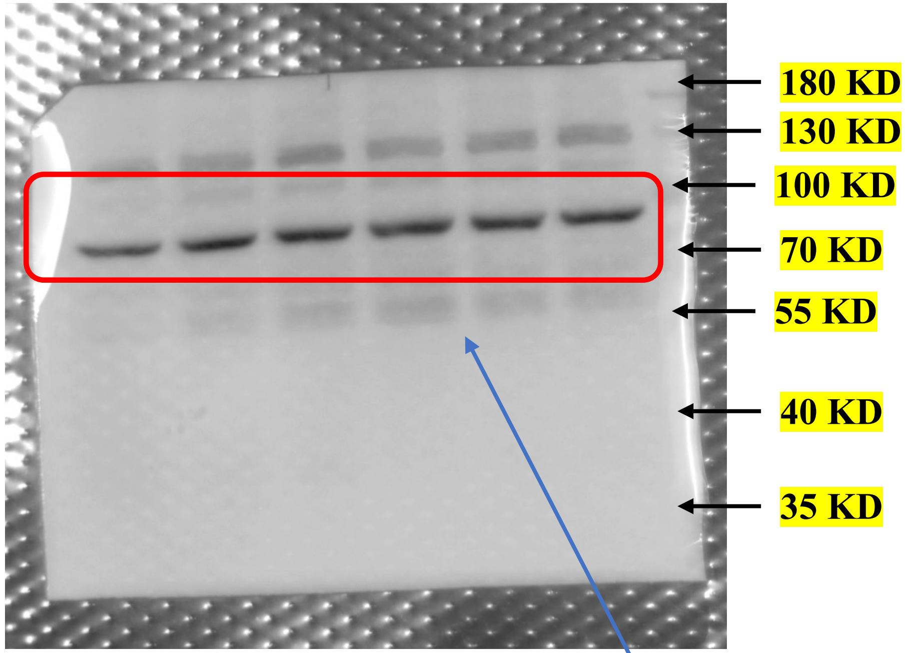

Chemiluminiscence

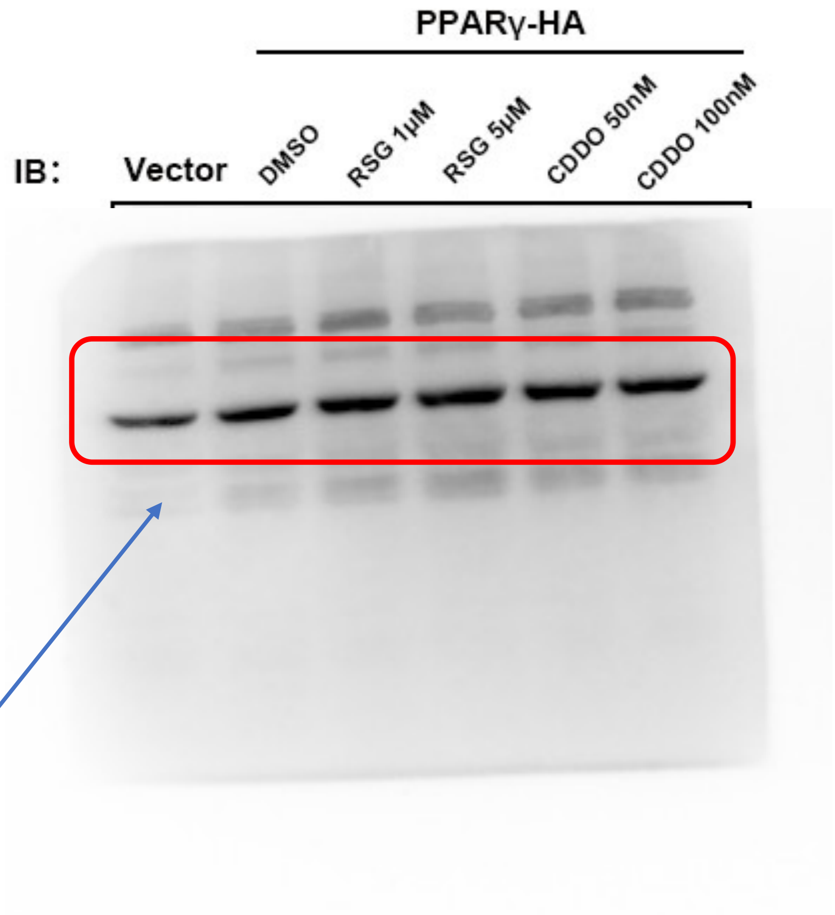

PKCa (≈ 85KD)

Antibody: PKCa antibody (Cell signaling technology #2056)

Full unedited gel for Figure S3f

Chemiluminiscence

10% gel Merged with the light field

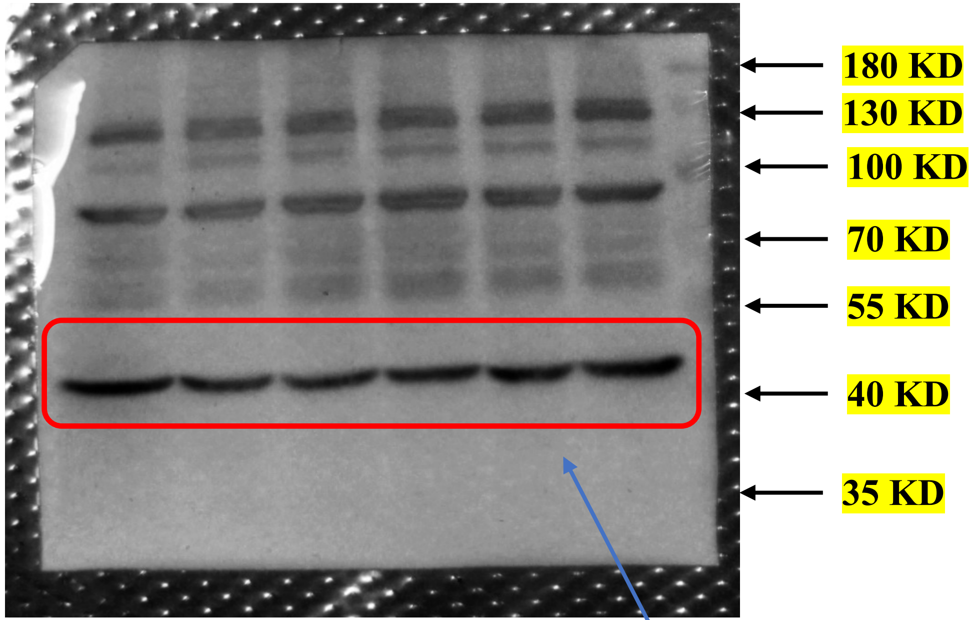

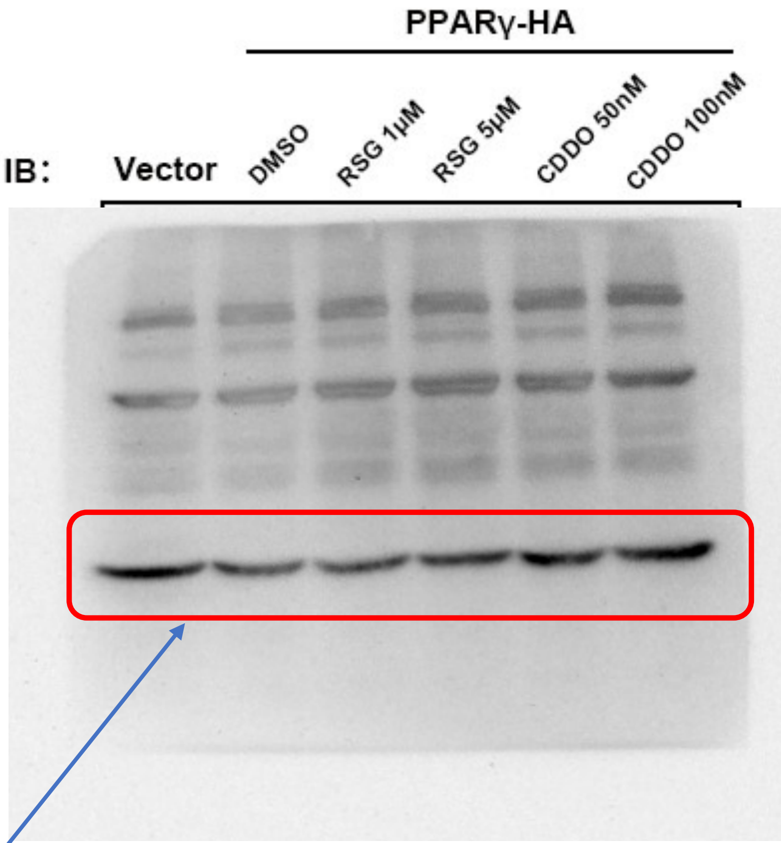

b-Actin (43KD)

Antibody: b-Actin (Genscript A00702)

Figure S3g

Full unedited gel for Figure S3g

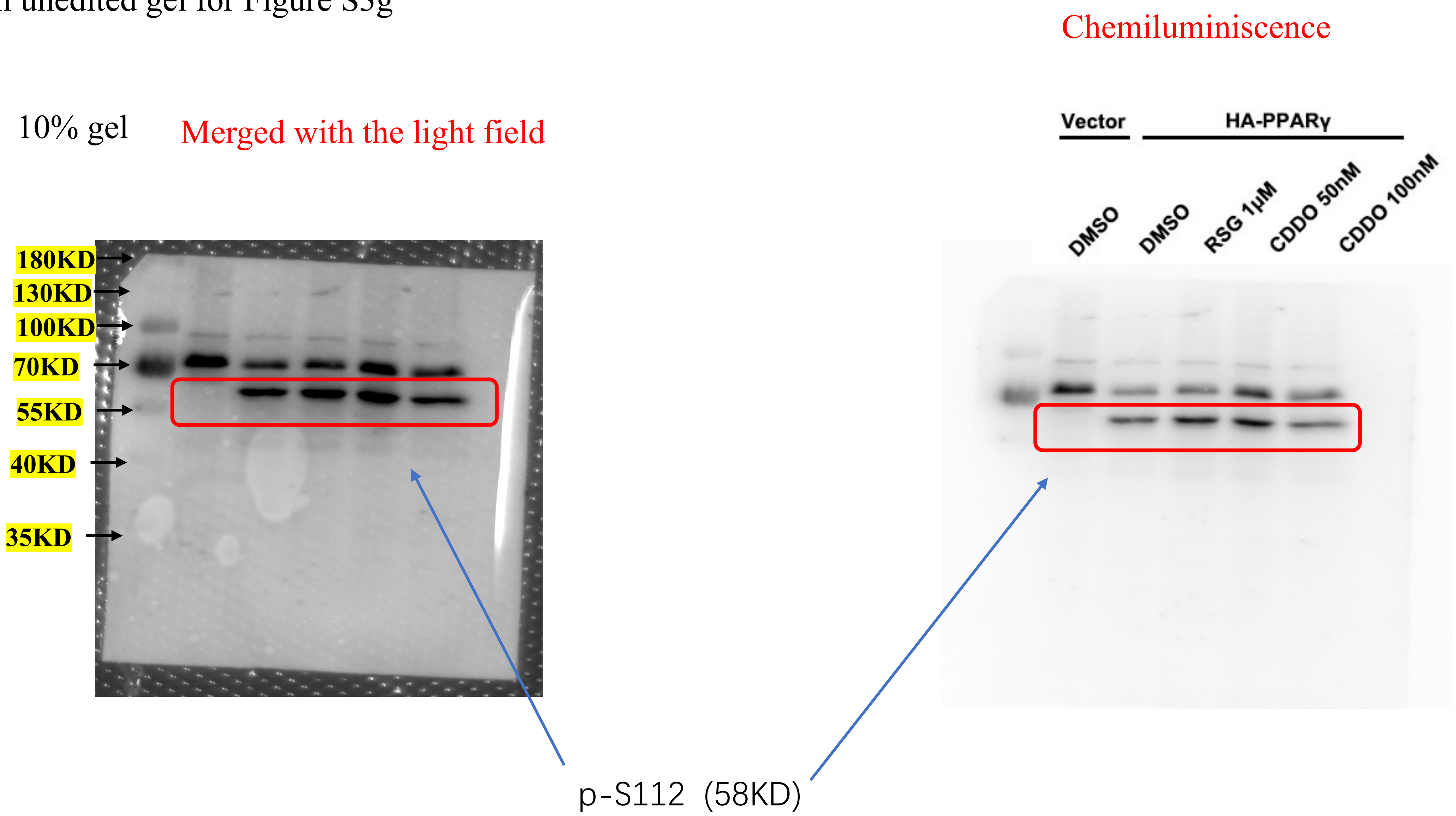

Antibody: anti-PPARg p-Ser112 (ab195925, Abcam)

Full unedited gel for Figure S3g

Chemiluminiscence

10% gel

Merged with the light field

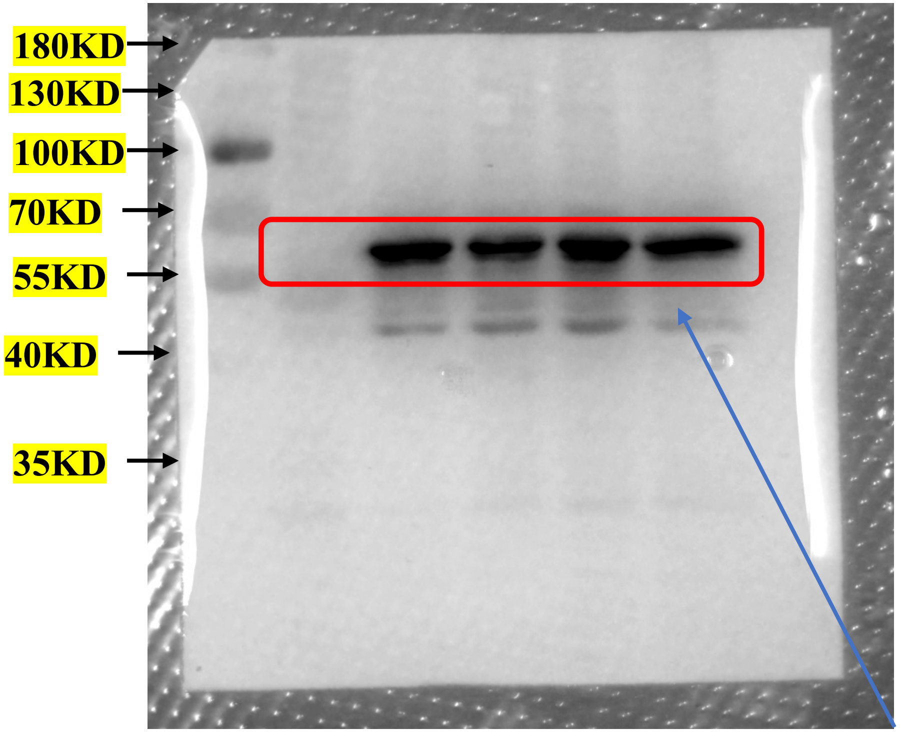

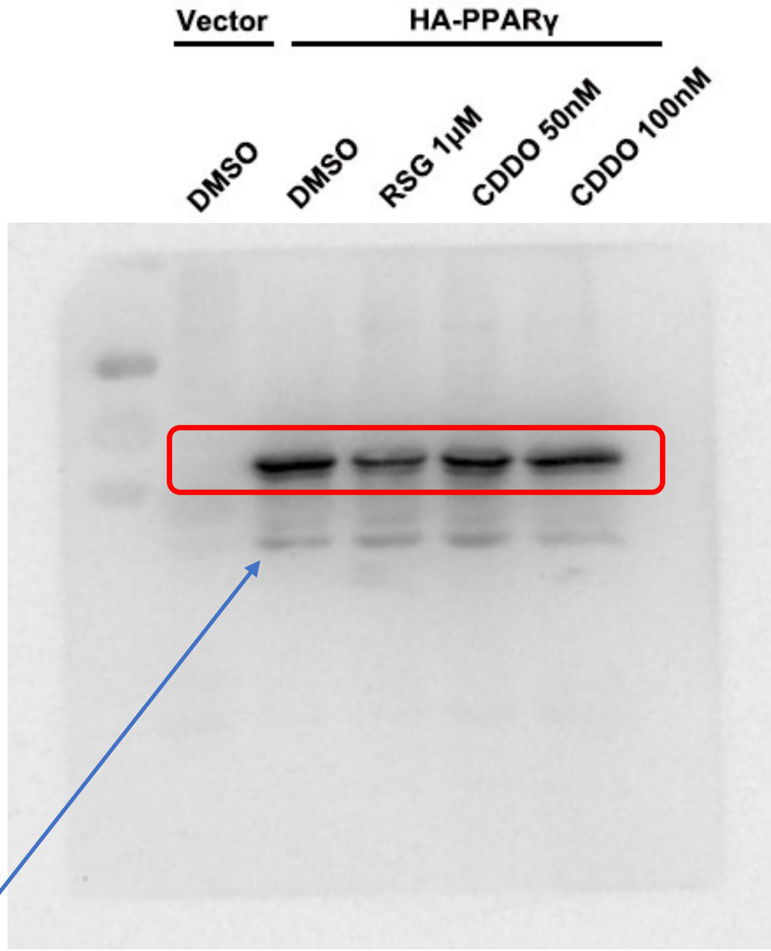

HA-PPARg (58KD)

Antibody: PPARg antibody (Santa Cruz sc-7273)

Full unedited gel for Figure S3g

10% gel Merged with the light field

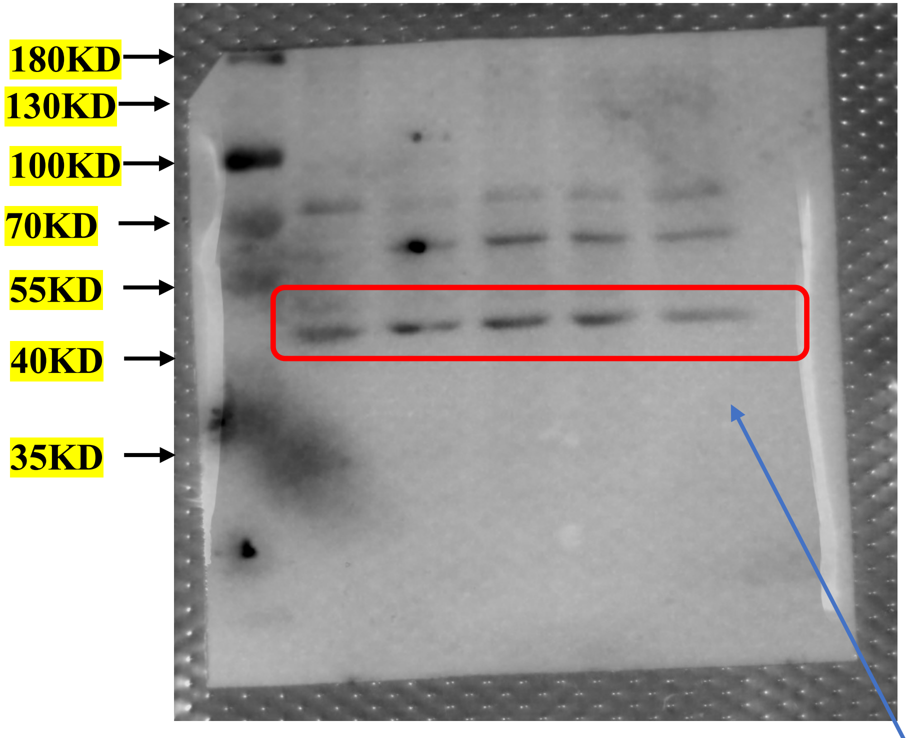

Chemiluminiscence

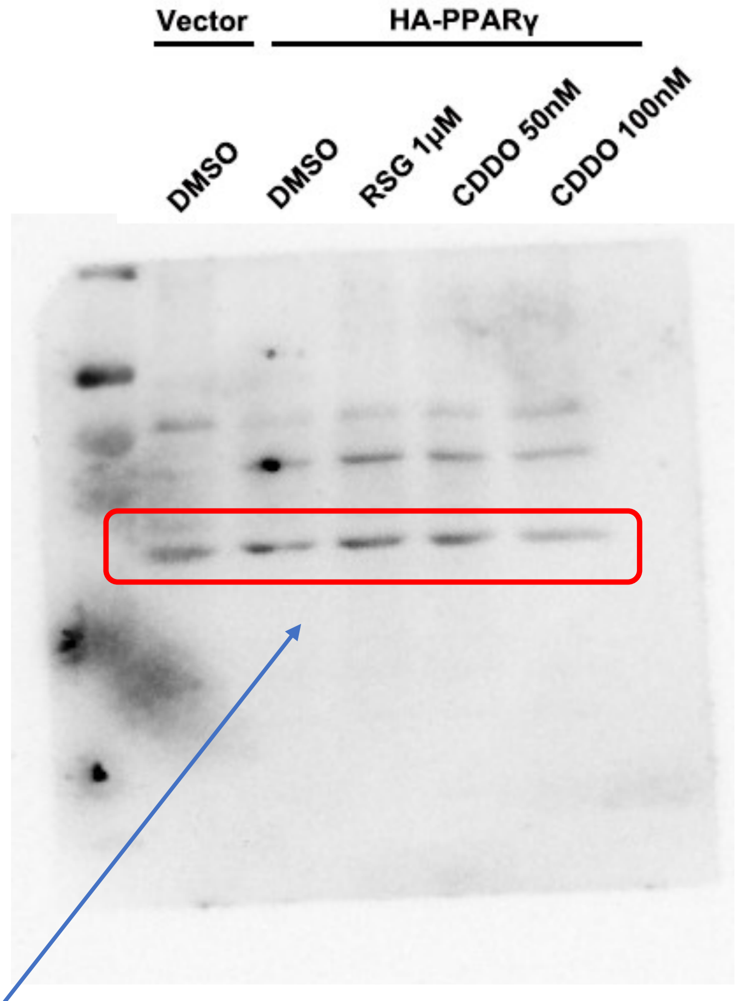

b-actin (43KD)

Antibody: b-Actin (Genscript A00702)

Figure S3h

Full unedited gel for Figure S3h

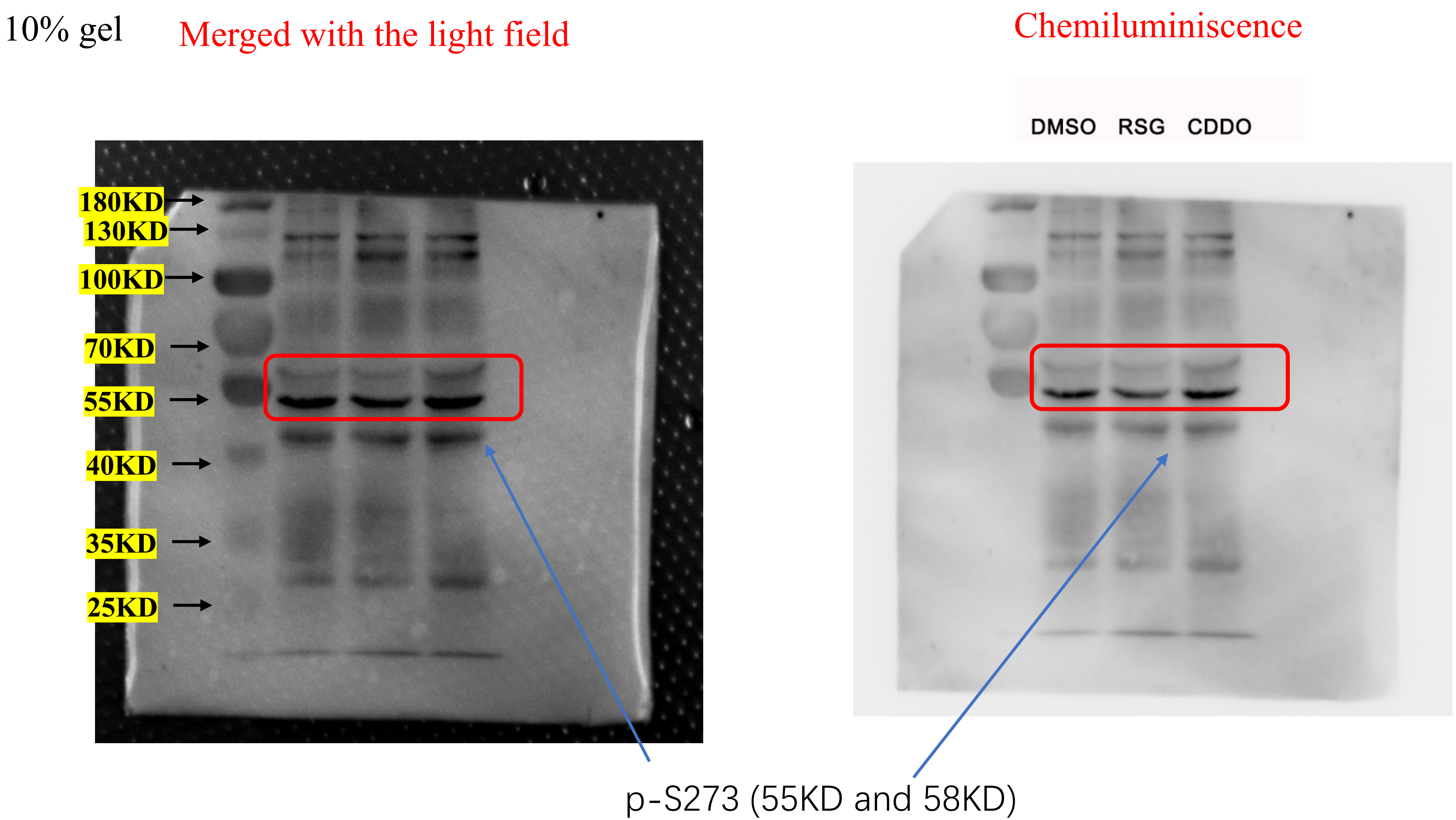

Antibody: Anti-PPARg p-Ser273 (bs-4888R, Bioss)

Full unedited gel for Figure S3h

10% gel

Merged with the light field

Chemiluminiscence

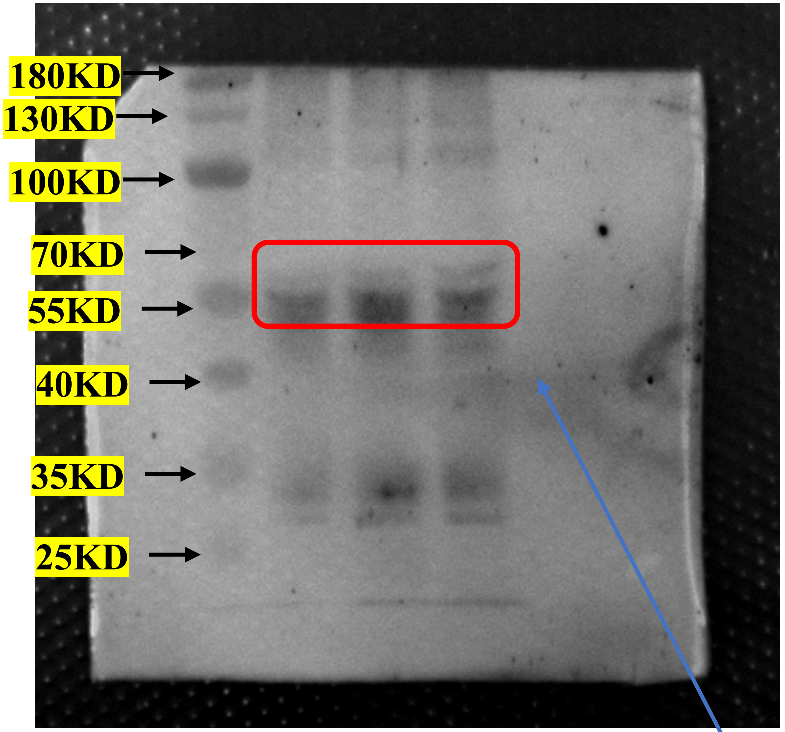

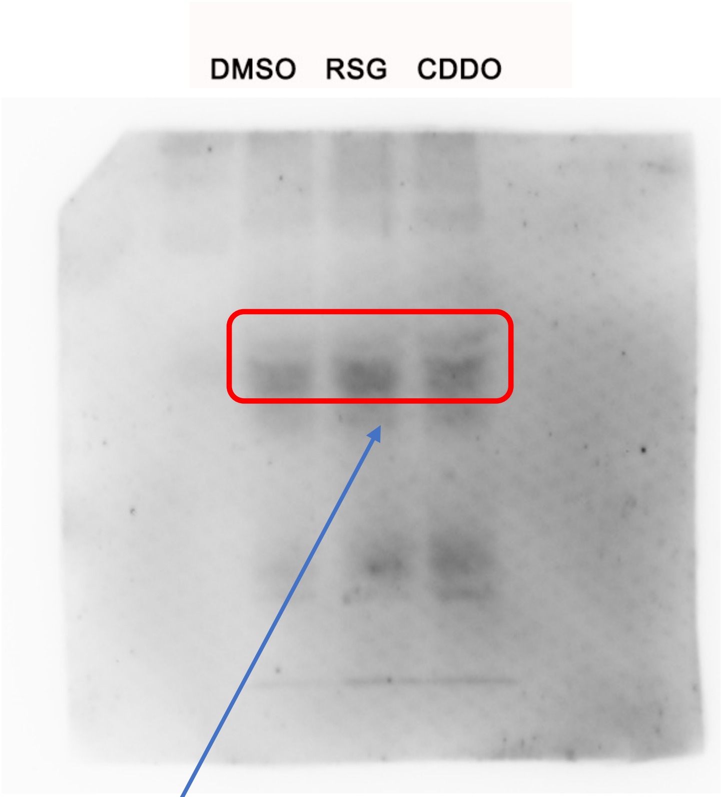

PPARg1 and PPARg2 (55KD and 58KD)

Antibody: PPARg antibody (Santa Cruz sc-7273)

Full unedited gel for Figure S3h

10% gel

Merged with the light field

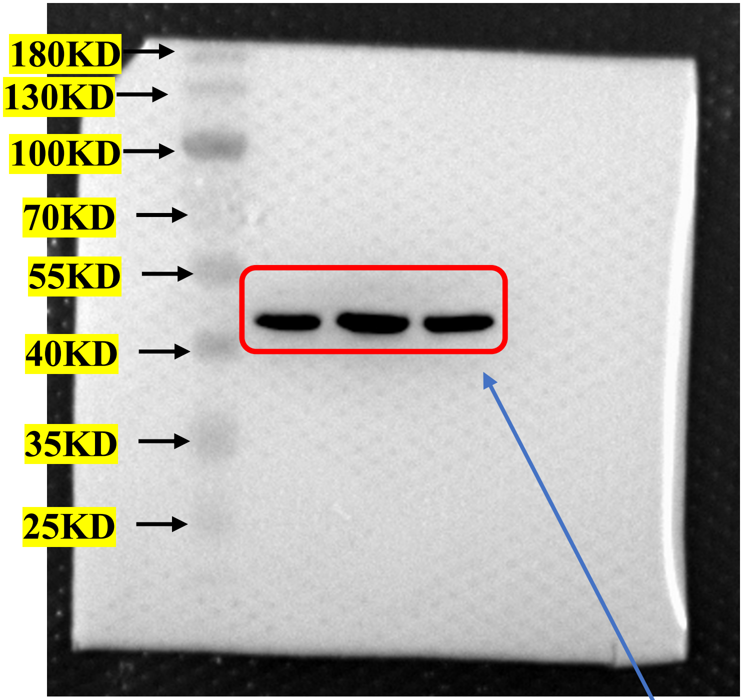

Chemiluminiscence

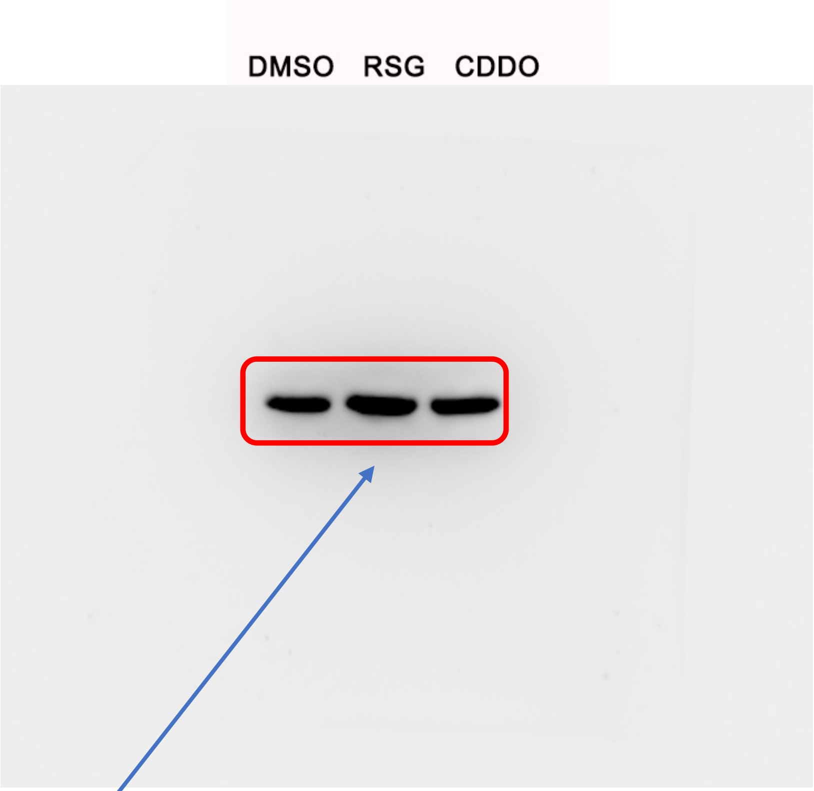

b-actin (43KD)

Antibody: b-Actin (Genscript A00702)

Figure S4b

Full unedited gel for Figure S4b

10% gel

Merged with the light field

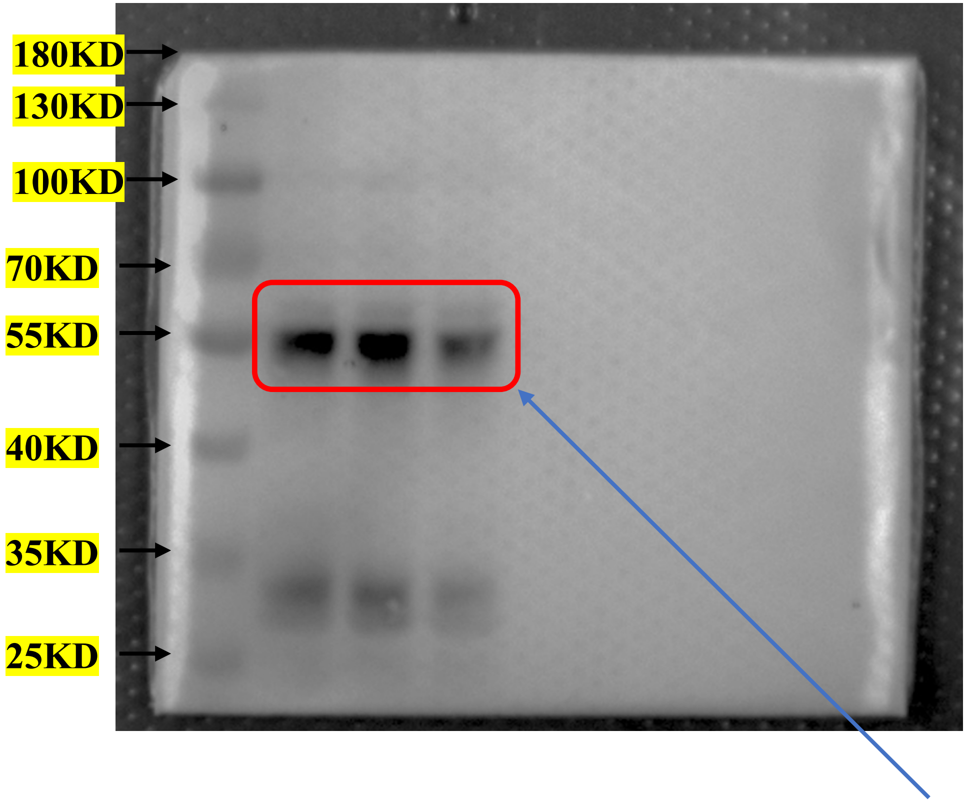

Chemiluminiscence

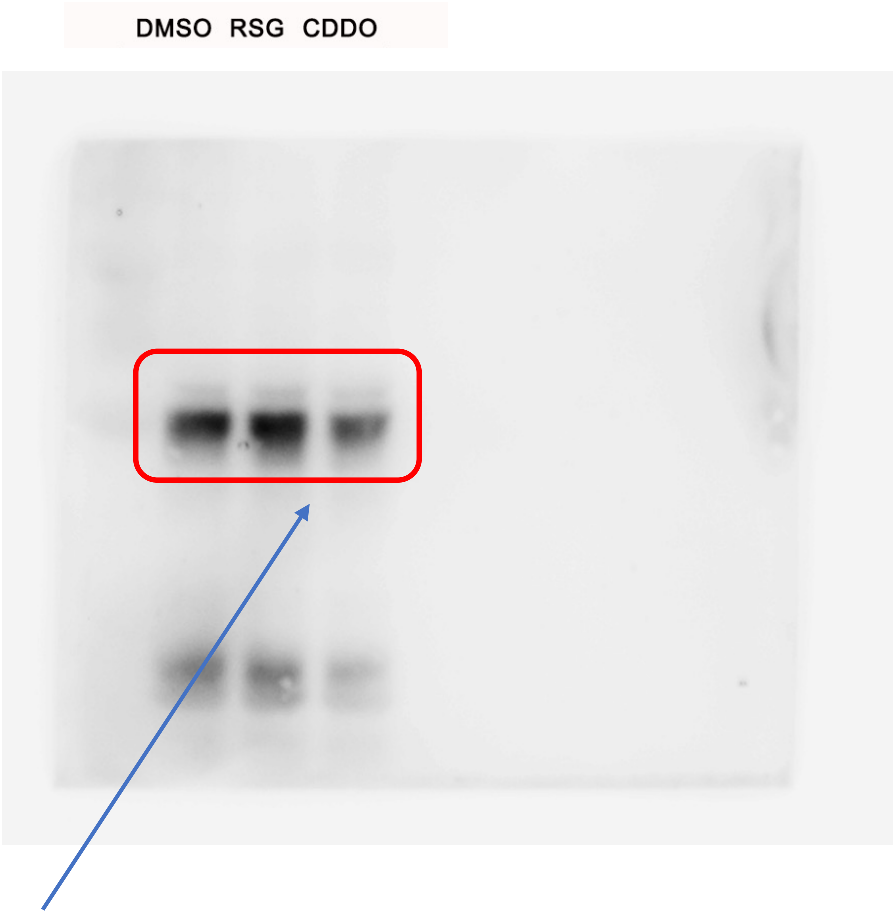

P-T166 (55KD and 58KD)

Antibody: T166 phosphorylation antibody generated by our lab

Full unedited gel for Figure S4b

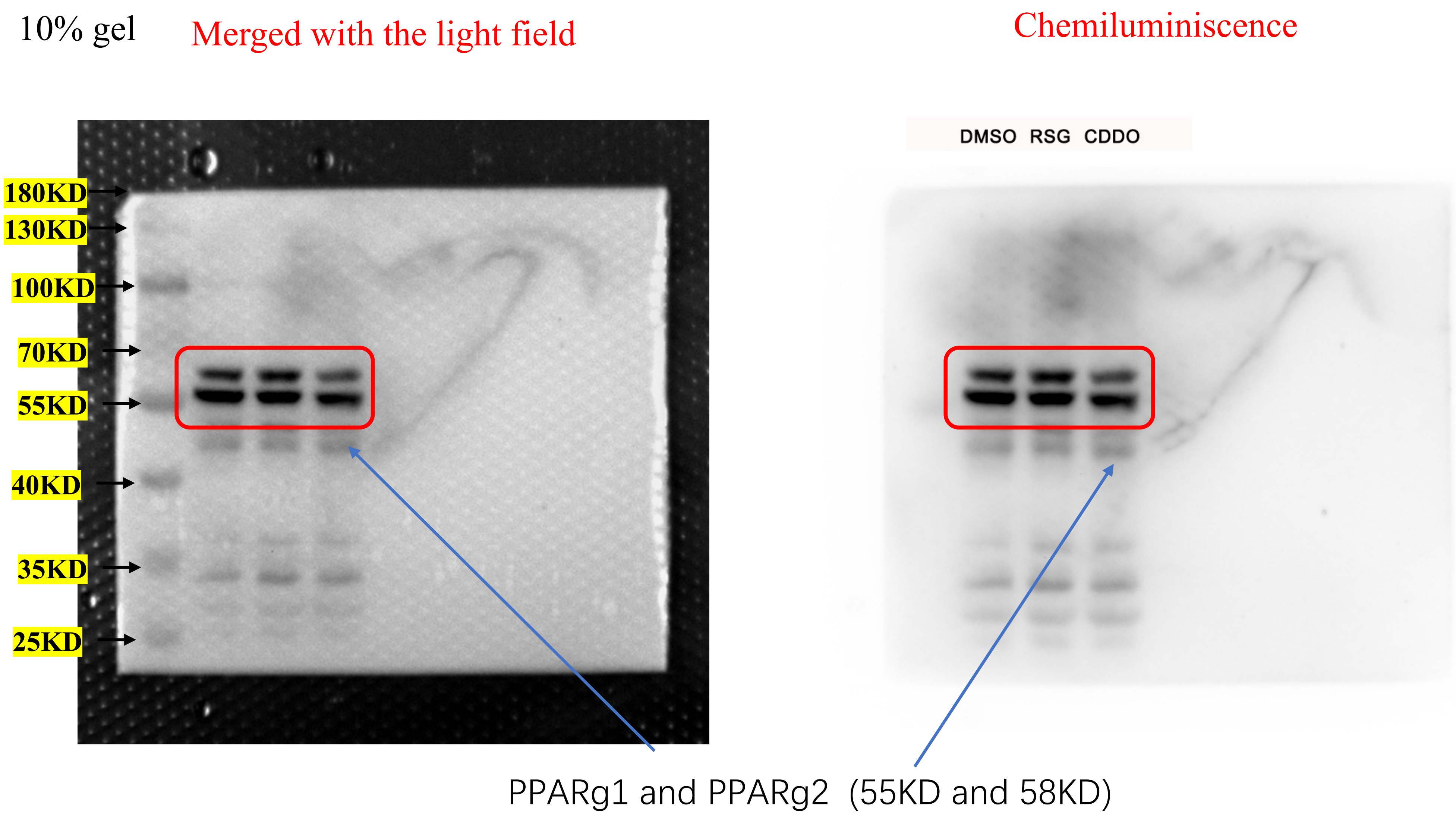

Antibody: PPARg antibody (Cell signaling technology #81b8)

Full unedited gel for Figure S4b

10% gel

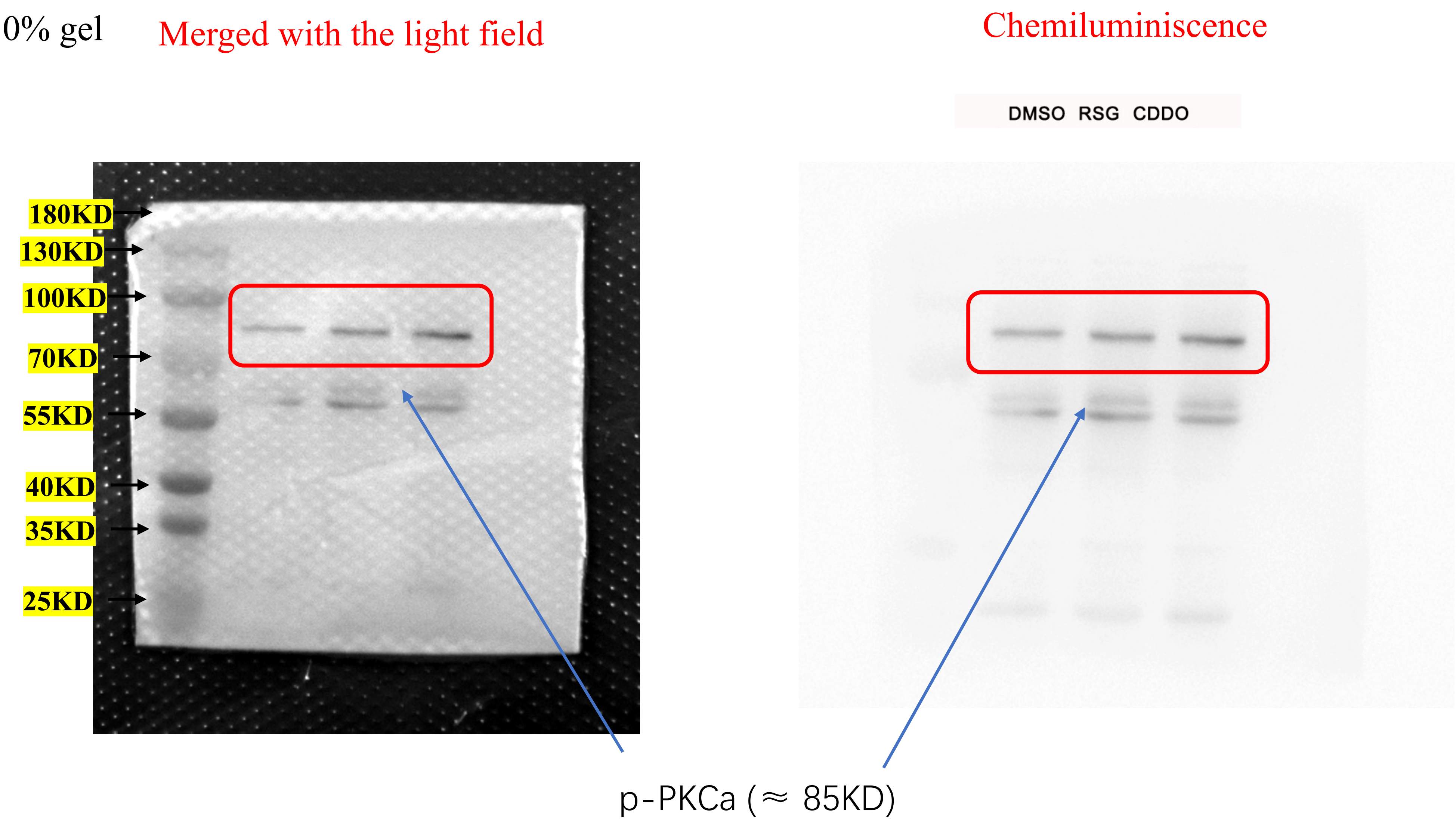

Antibody: p-PKCa antibody (Abcam ab32502)

Full unedited gel for Figure S4b

10% gel

Merged with the light field

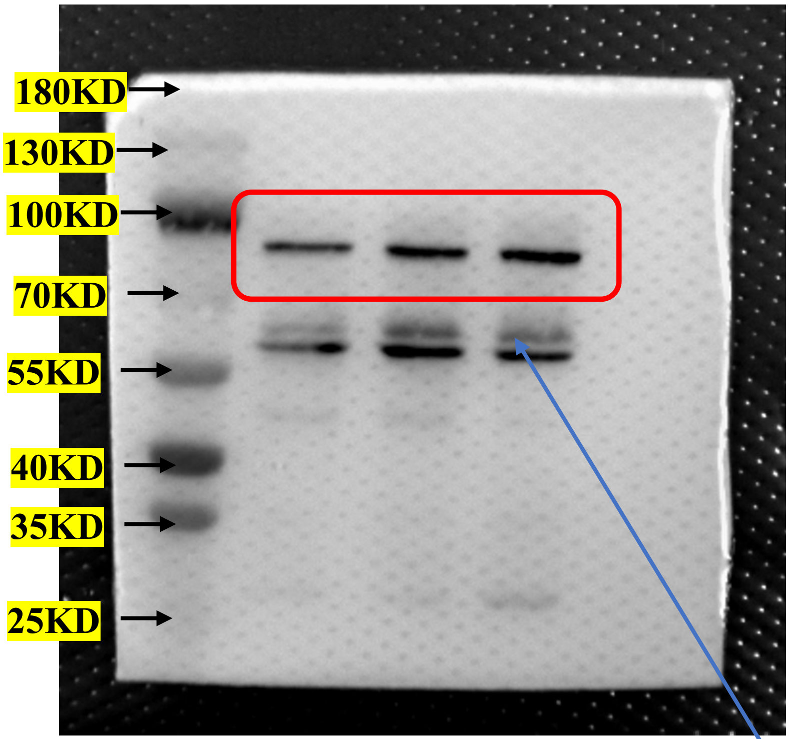

Chemiluminiscence

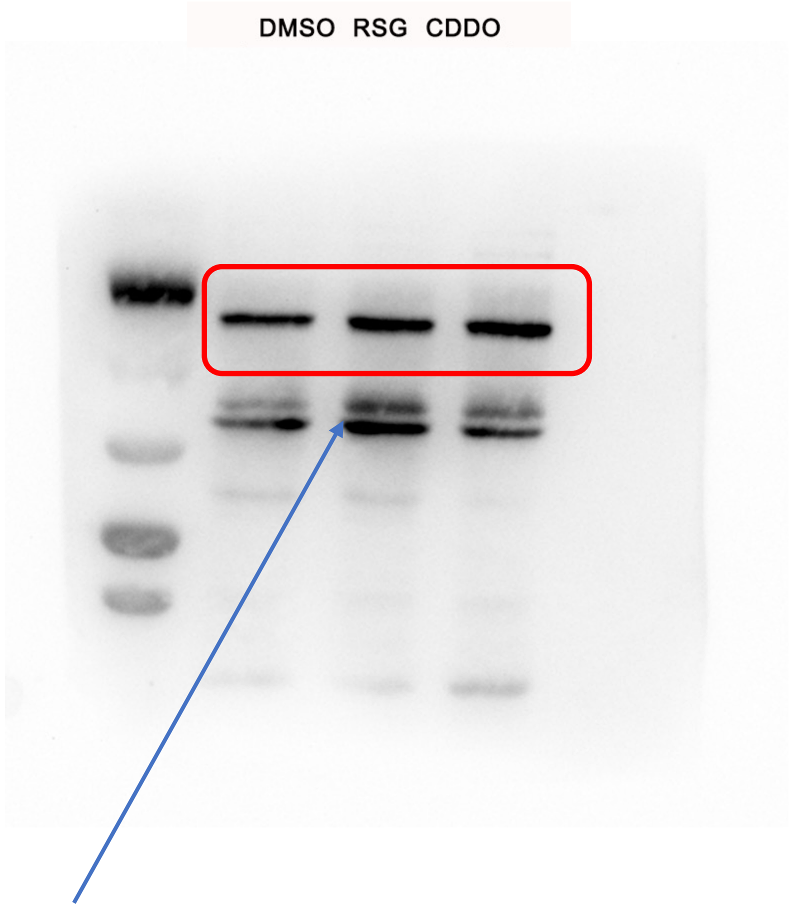

PKCa (≈ 85KD)

Antibody: PKCa antibody (Cell signaling technology #2056)

Full unedited gel for Figure S4b

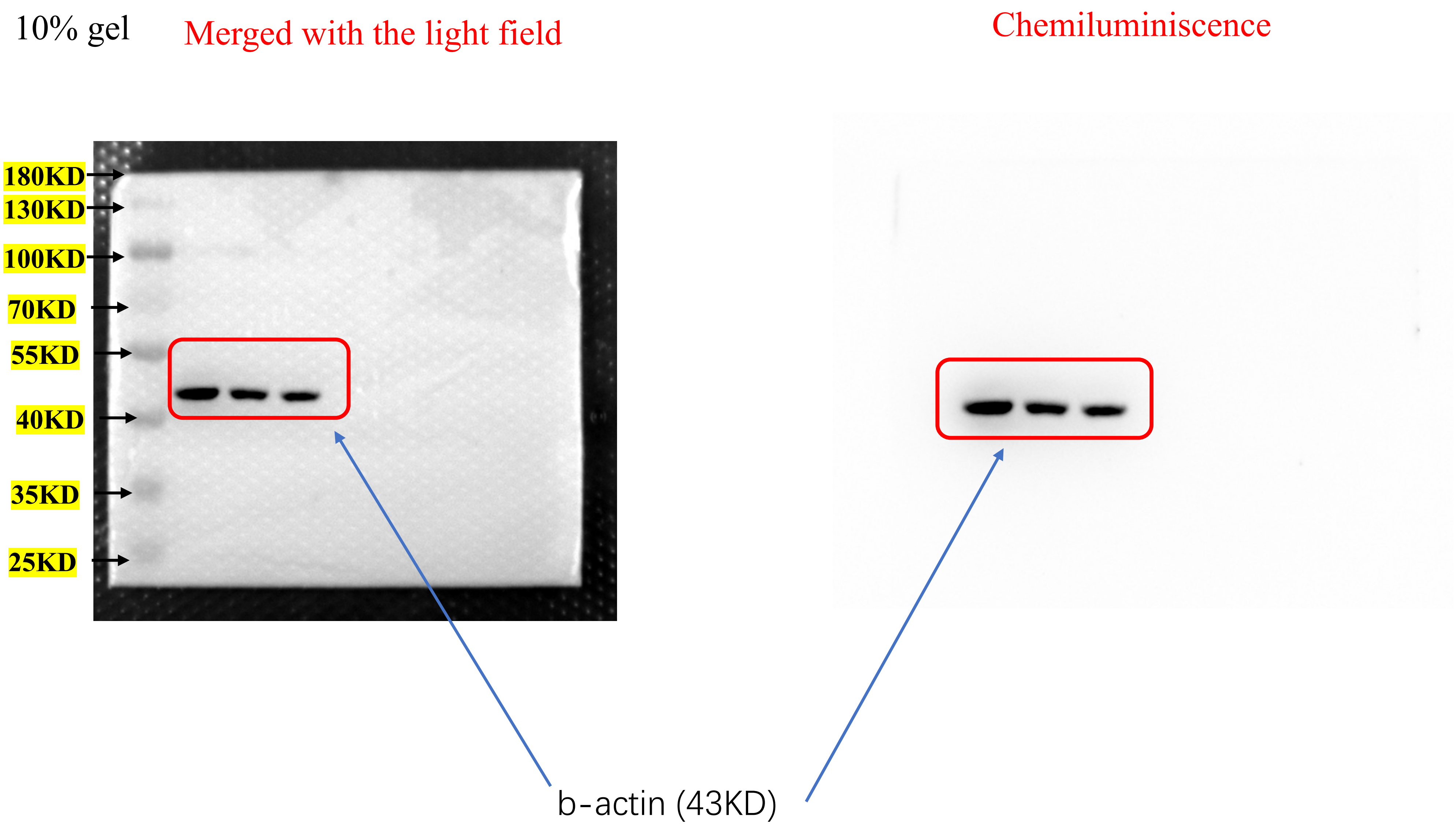

Antibody: b-Actin (Genscript A00702)

Figure S4d

Full unedited gel for Figure S4d

12% gel

Merged with the light field

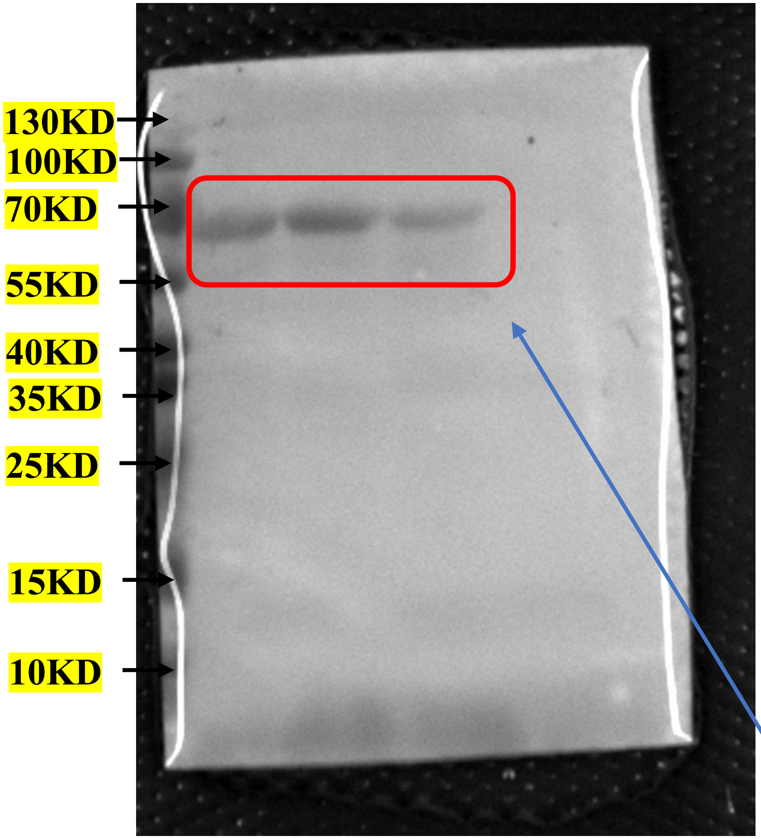

Chemiluminiscence

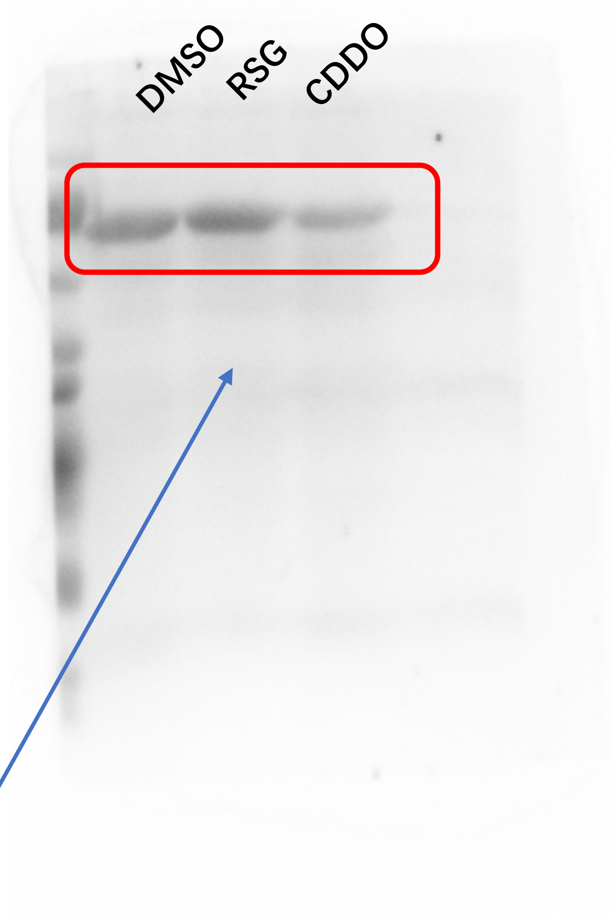

Human sample #1: P-T166 (≈60KD)

Antibody: T166 phosphorylation antibody generated by our lab

Full unedited gel for Figure S4d

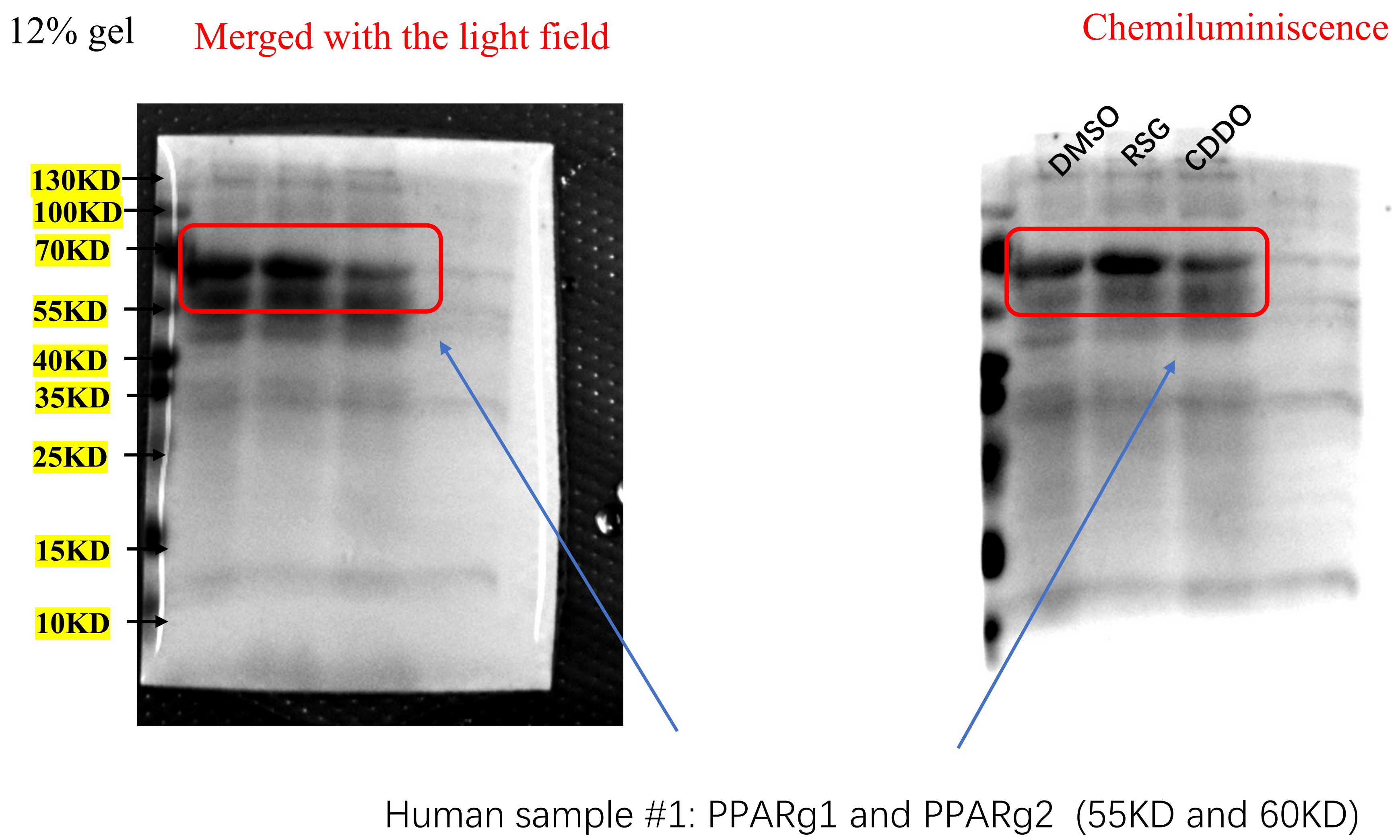

Antibody: PPARg antibody (Santa Cruz sc-7273)

Full unedited gel for Figure S4d

10% gel — Merged with the light field — Chemiluminiscence

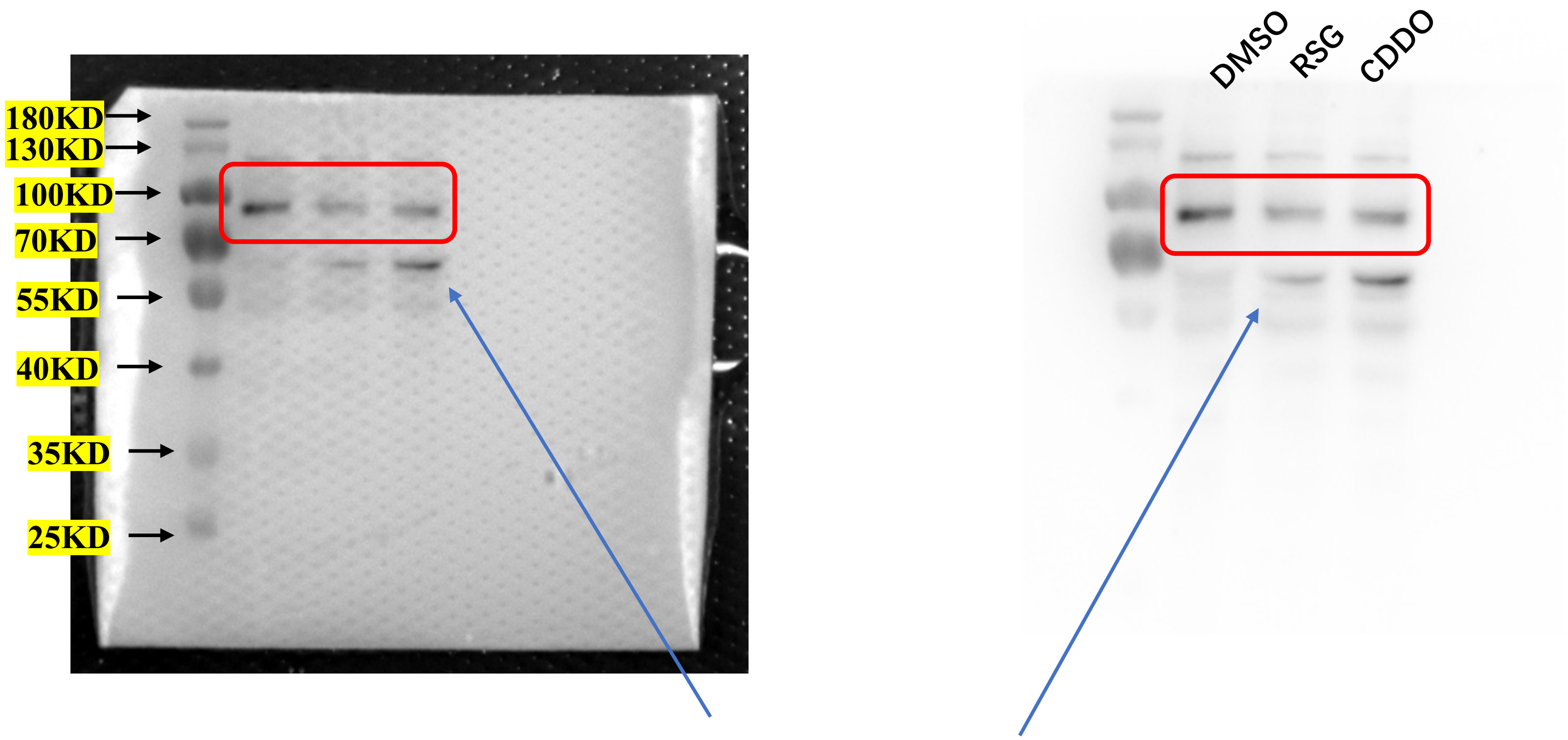

Human sample #1: p-PKCa (≈ 85KD)

Antibody: p-PKCa antibody (Abcam ab32502)

Full unedited gel for Figure S4d

10% gel

Merged with the light field

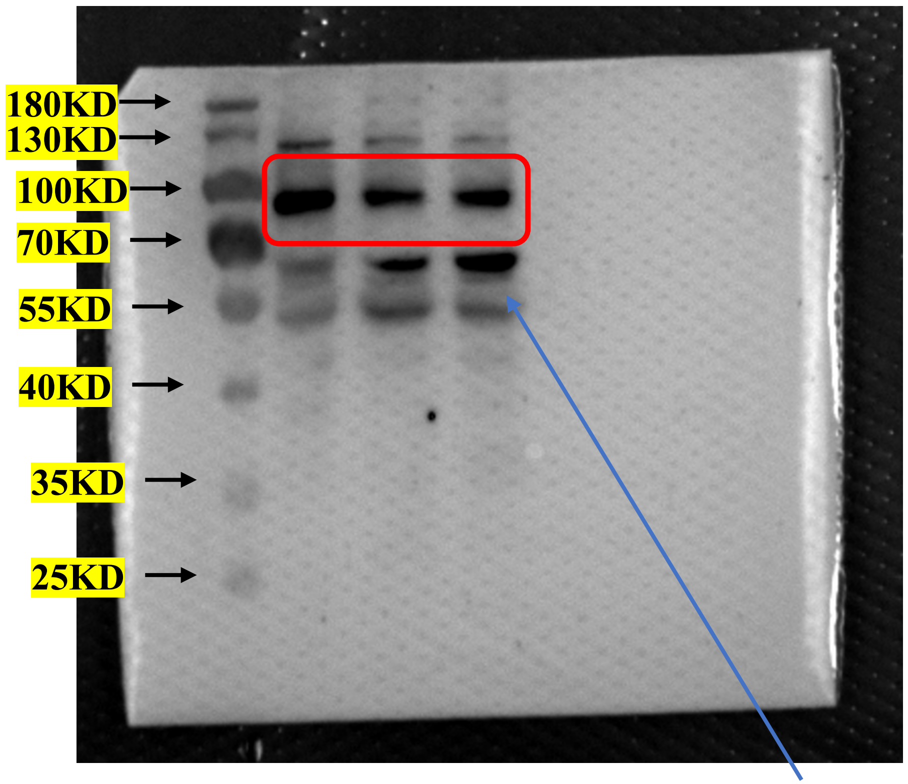

Chemiluminiscence

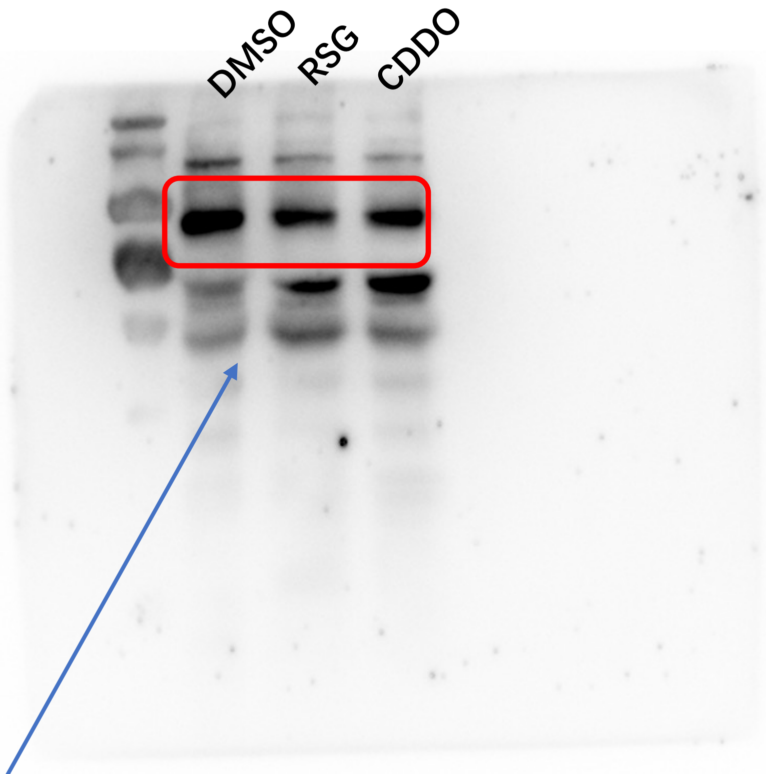

Human sample #1: PKCa (≈85KD)

Antibody: PKCa antibody (Cell signaling technology #2056)

Full unedited gel for Figure S4d

10% gel

Merged with the light field

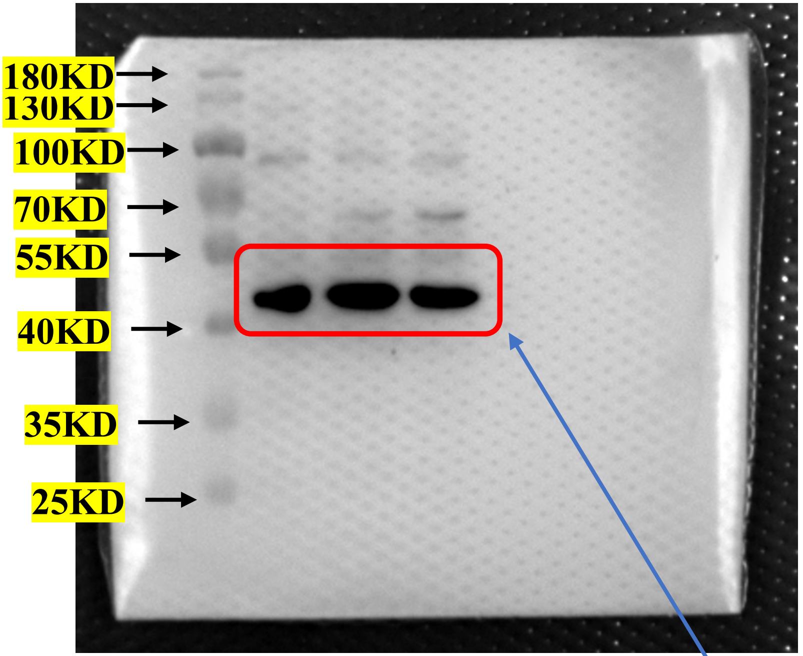

Chemiluminiscence

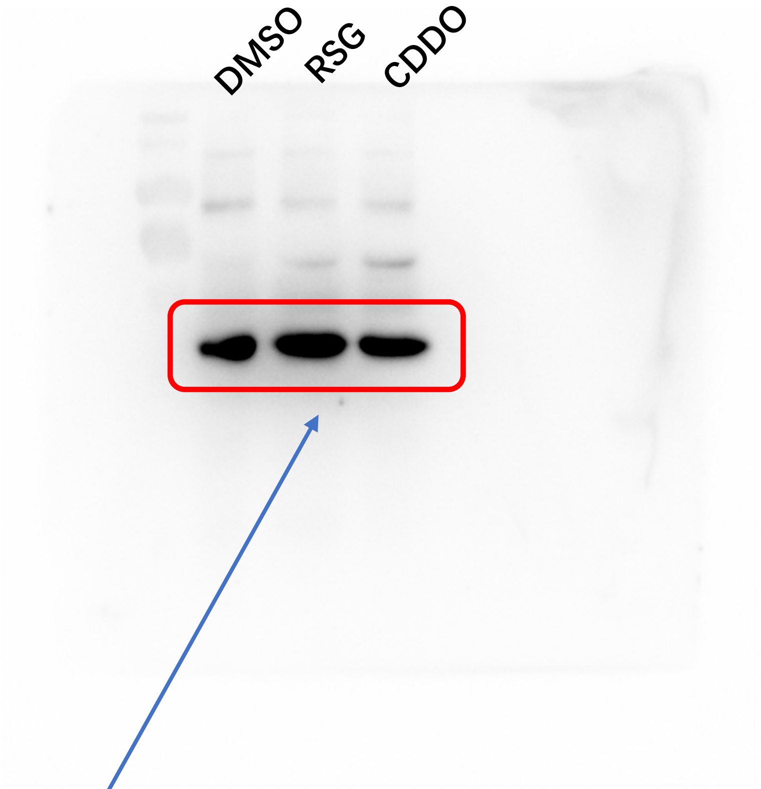

b-actin (43KD)

Antibody: b-Actin (Genscript A00702)

Figure S4e

Full unedited gel for Figure S4e

10% gel

Merged with the light field

Chemiluminiscence

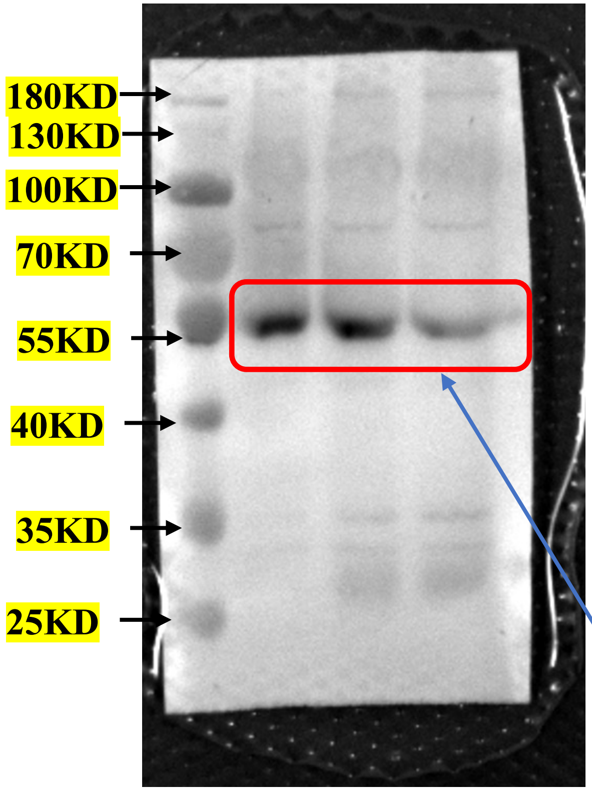

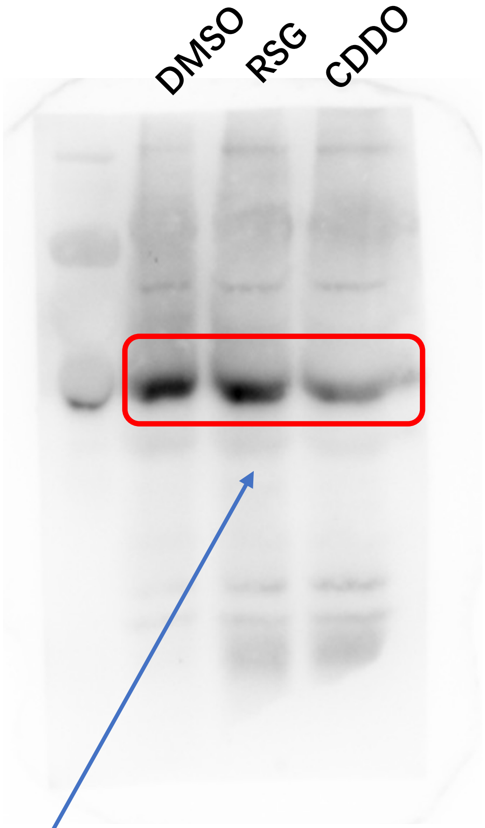

Human sample #2: P-T166 (55KD)

Antibody: T166 phosphorylation antibody generated by our lab

Full unedited gel for Figure S4e

10% gel Merged with the light field

Chemiluminiscence

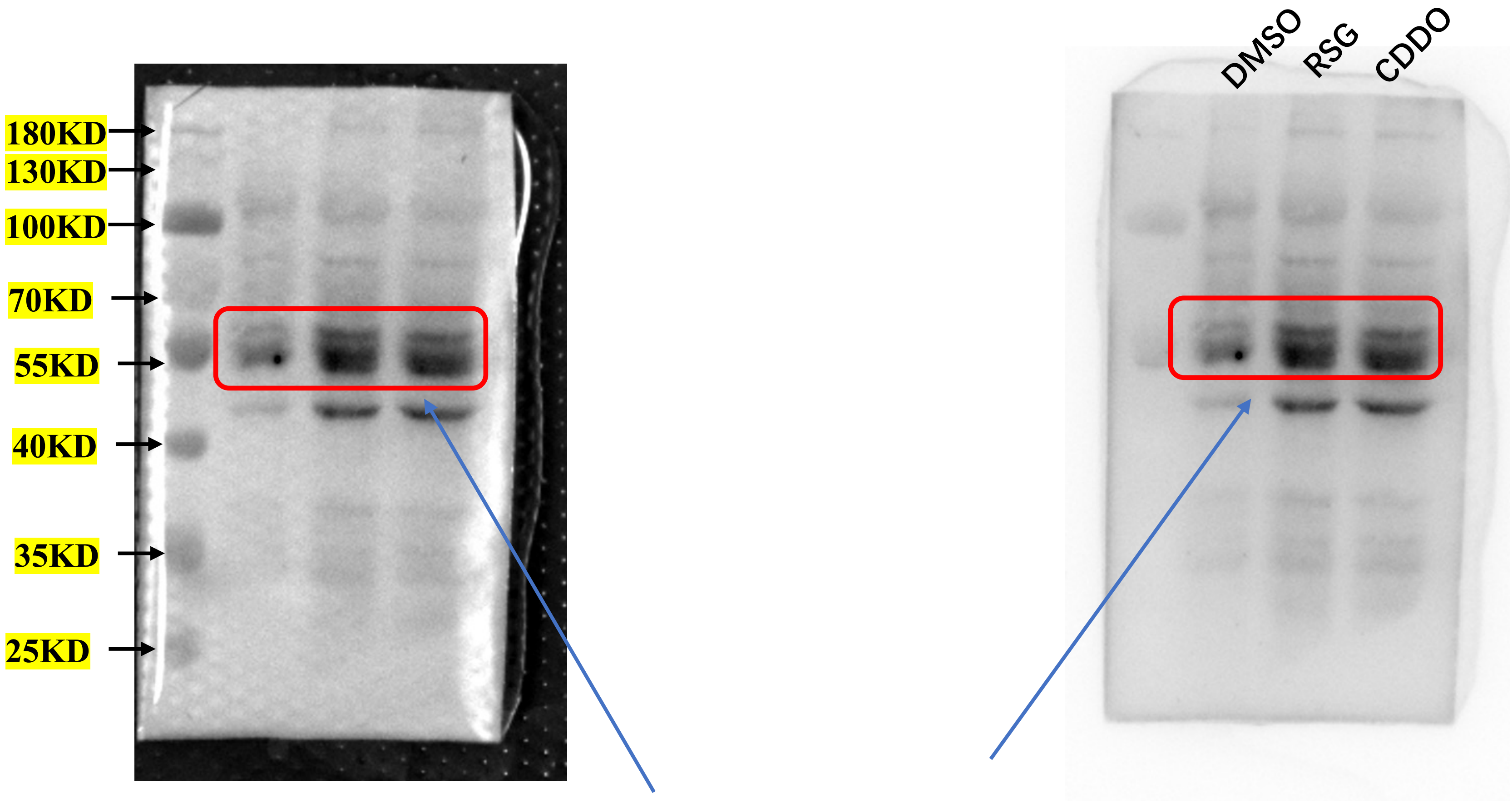

Human sample #2: PPARg1 and PPARg2 (55KD and 58KD)

Antibody: PPARg antibody (Santa Cruz sc-7273)

Full unedited gel for Figure S4e

10% gel

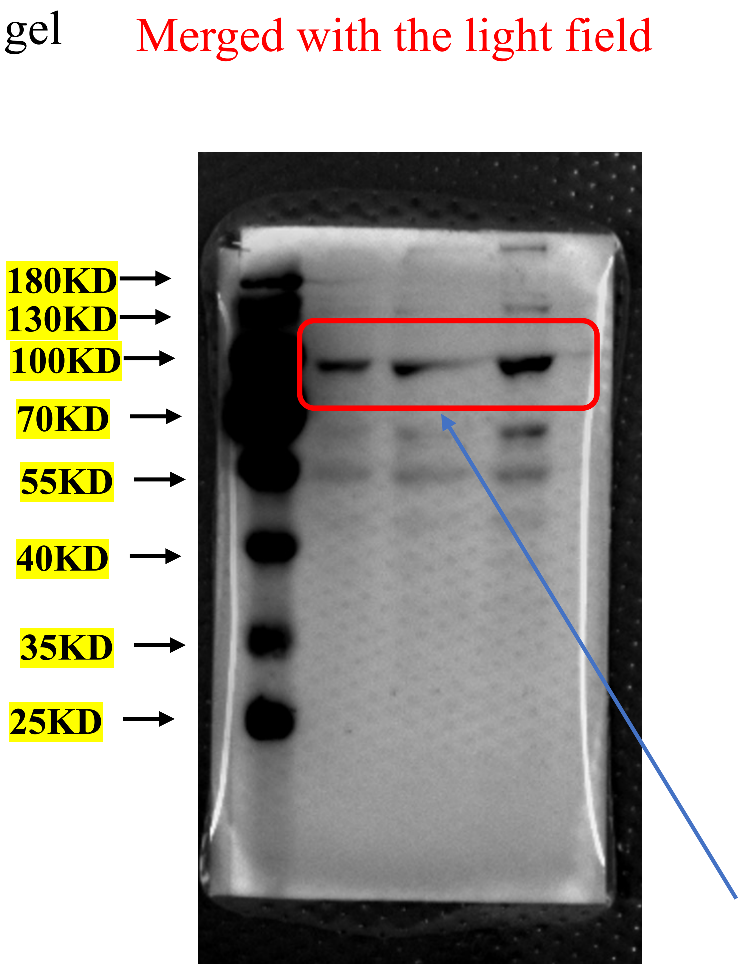

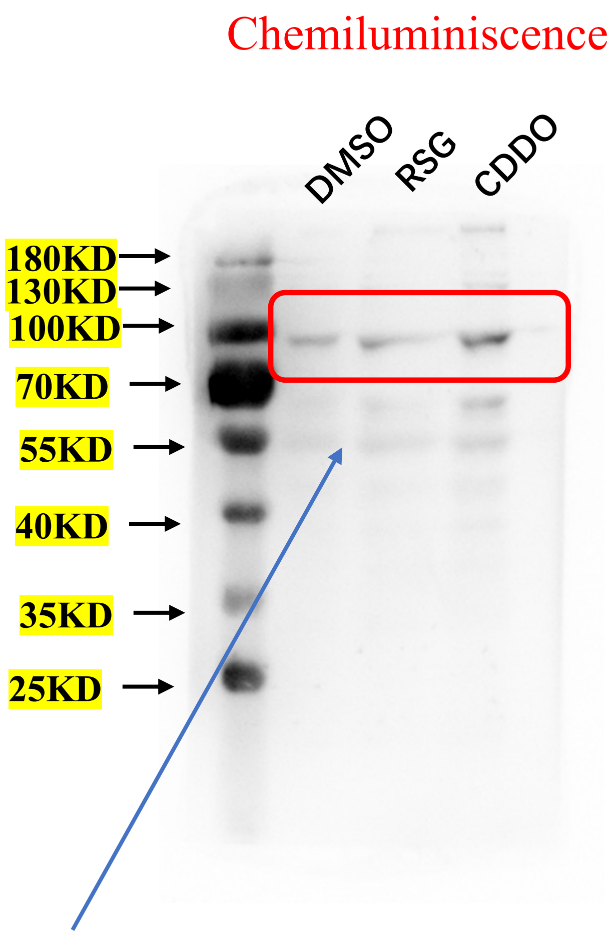

Human sample #2: p-PKCa (≈ 85KD)

Antibody: p-PKCa antibody (Abcam ab32502)

Full unedited gel for Figure S4e

10% gel

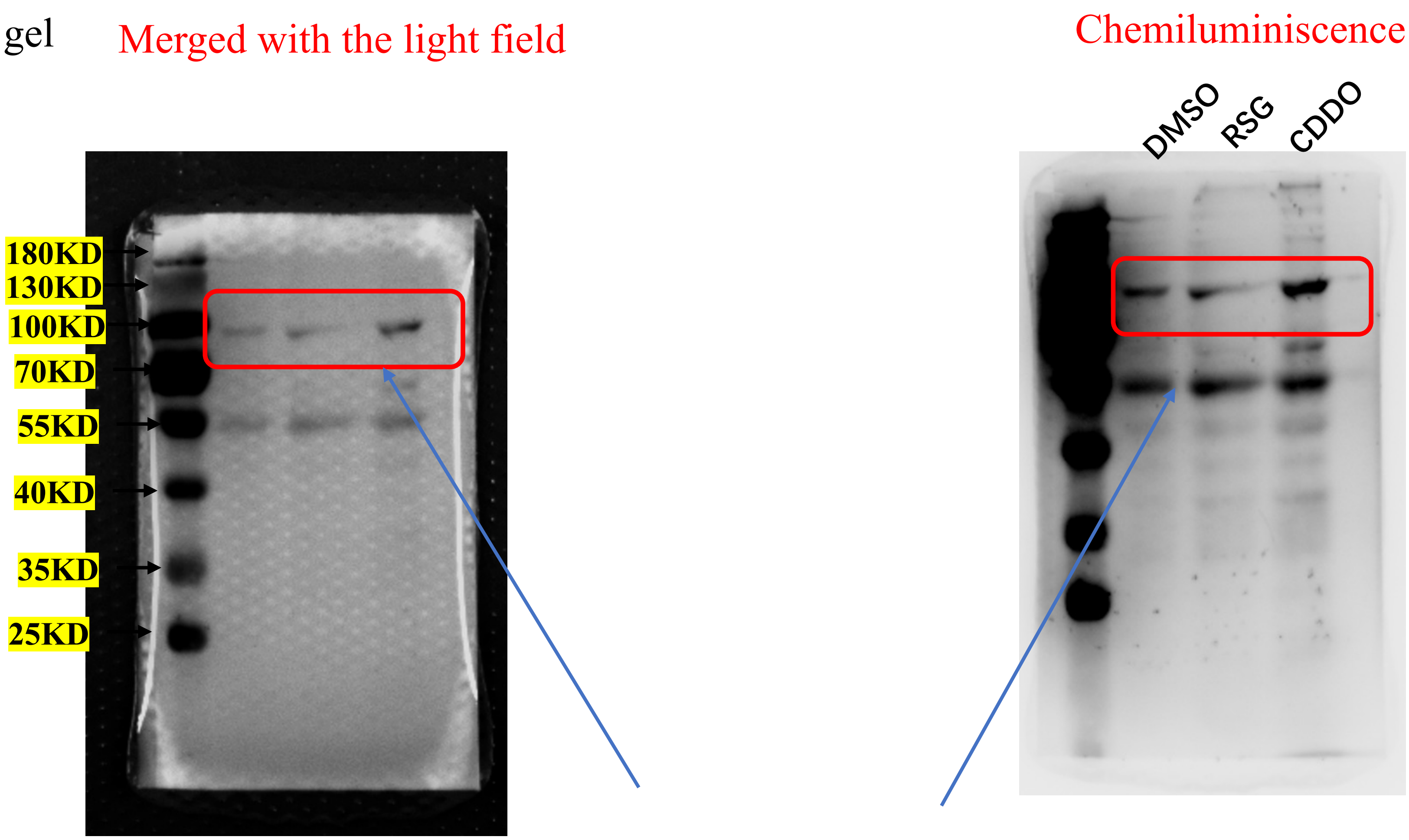

Human sample #2: PKCa (≈85KD)

Antibody: PKCa antibody (Cell signaling technology #2056)

Full unedited gel for Figure S4e

10% gel

Merged with the light field

Chemiluminiscence

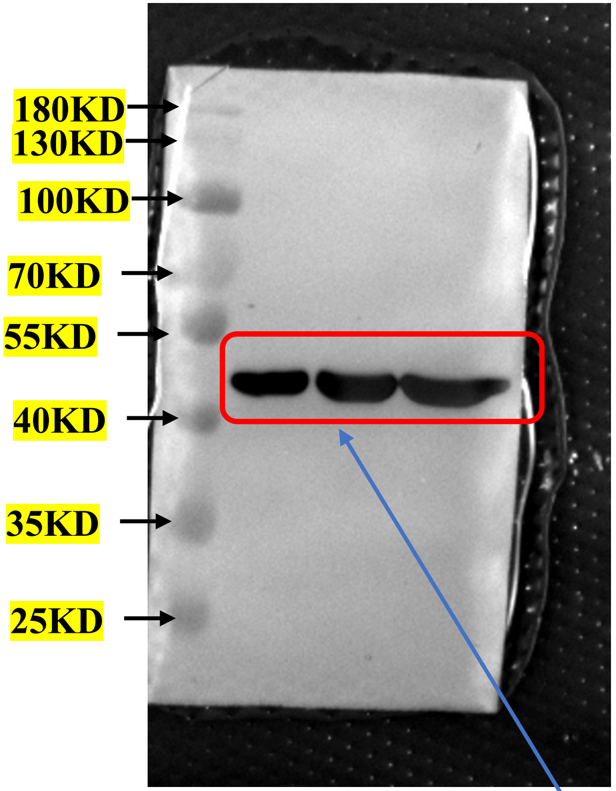

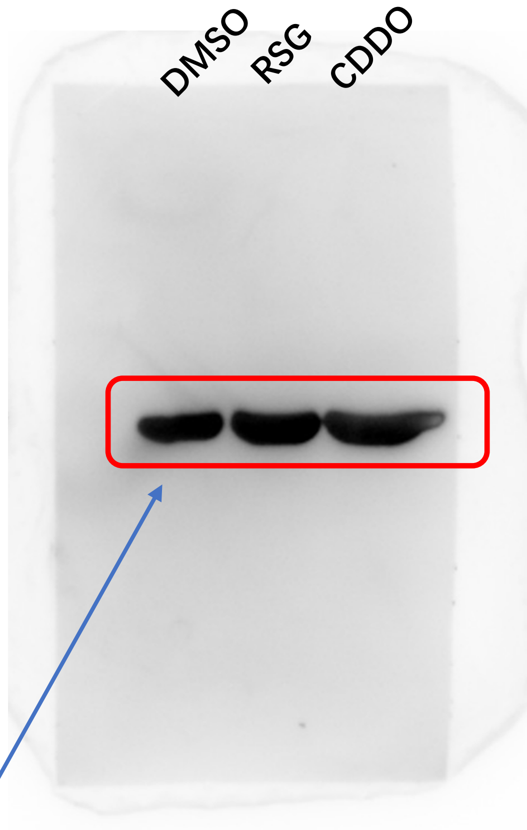

b-actin (43KD)

Antibody: b-Actin (Genscript A00702)

Figure S4g

Full unedited gel for Figure S4g

10% gel Merged with the light field

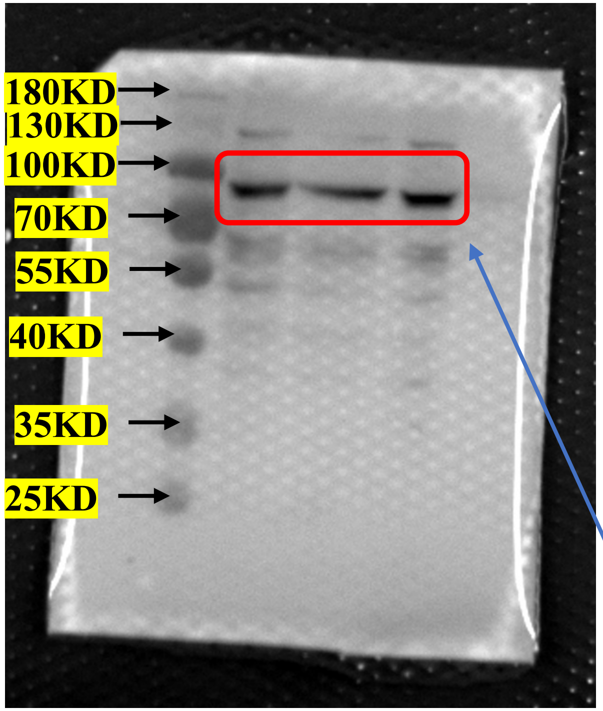

Chemiluminiscence

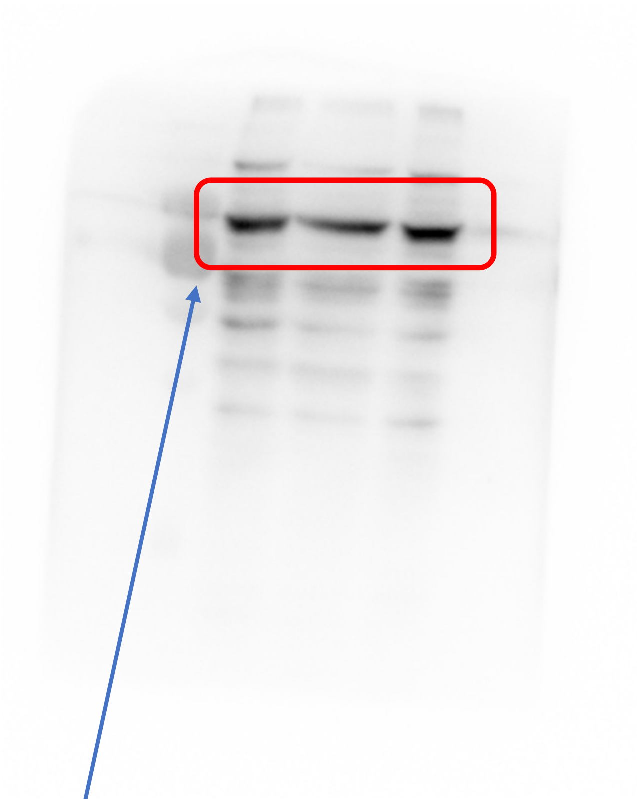

Human sample #3: p-PKCa (≈ 85KD)

Antibody: p-PKCa antibody (Abcam ab32502)

Full unedited gel for Figure S4g

10% gel Merged with the light field

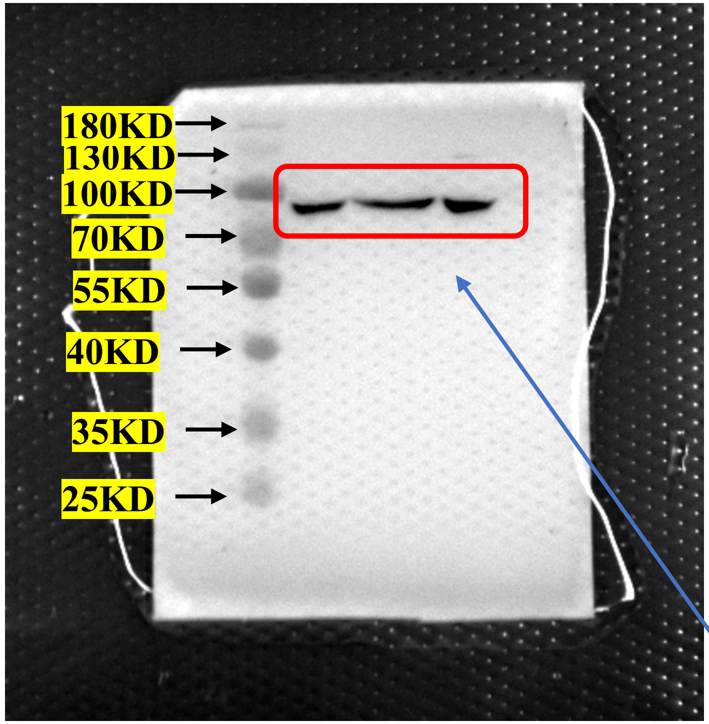

Chemiluminiscence

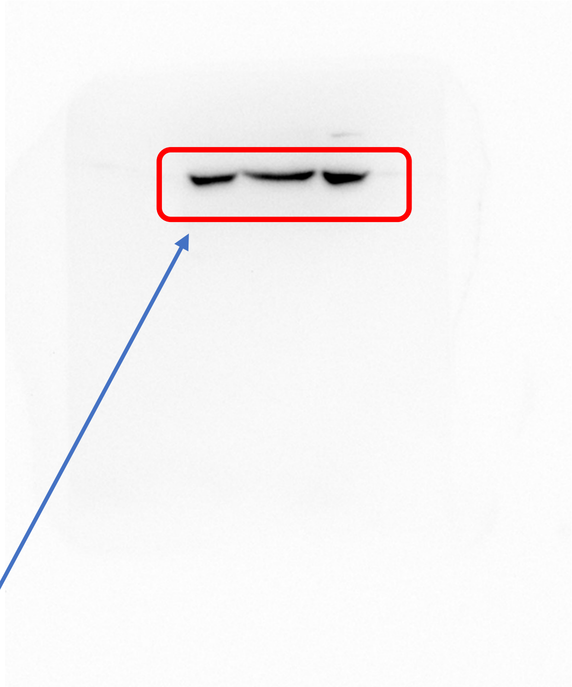

Human sample #3: PKCa (≈85KD)

Antibody: PKCa antibody (Cell signaling technology #2056)

Full unedited gel for Figure S4g

10% gel

Merged with the light field

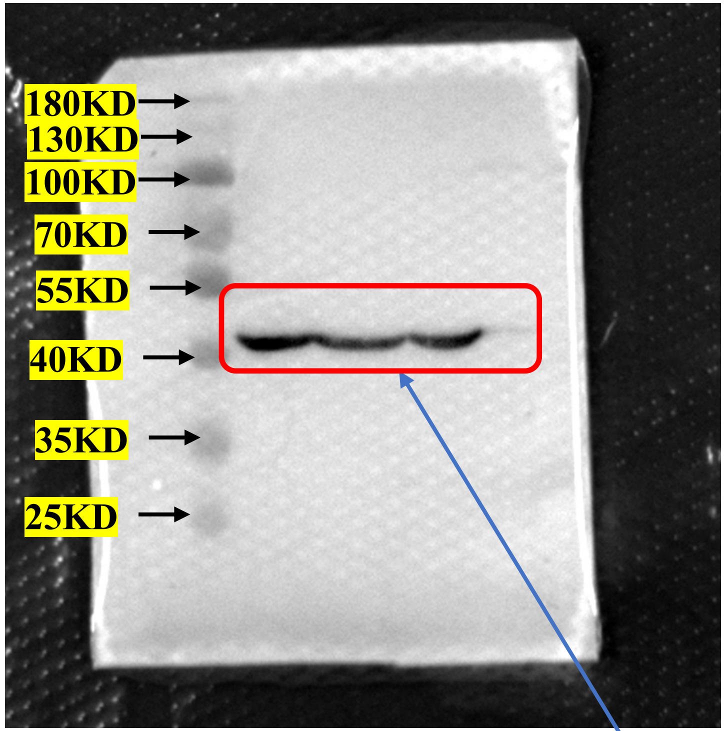

Chemiluminiscence

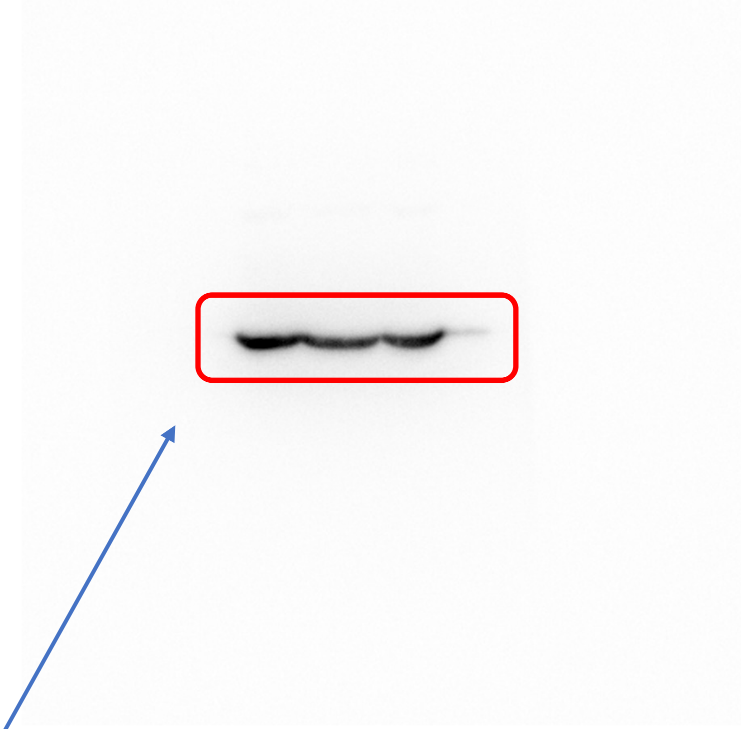

b-actin (43KD)

Antibody: b-Actin (Genscript A00702)

Full unedited gel for Figure S4g

10% gel

Merged with the light field

Chemiluminiscence

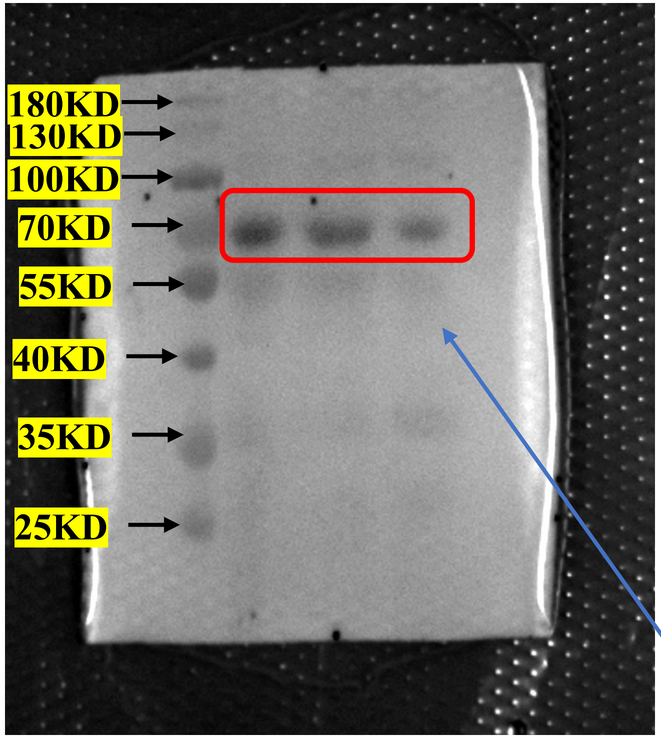

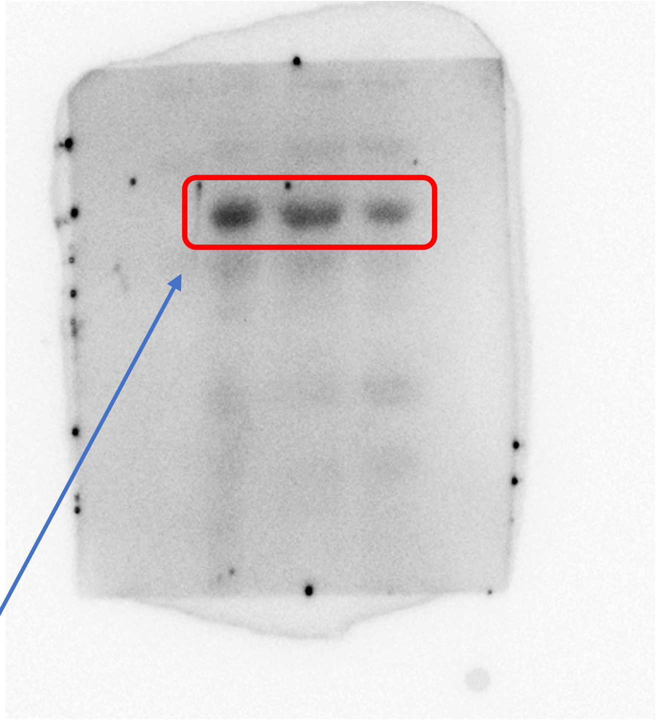

Human sample #3: P-T166 (≈60KD)

Antibody: T166 phosphorylation antibody generated by our lab

Full unedited gel for Figure S4g

10% gel

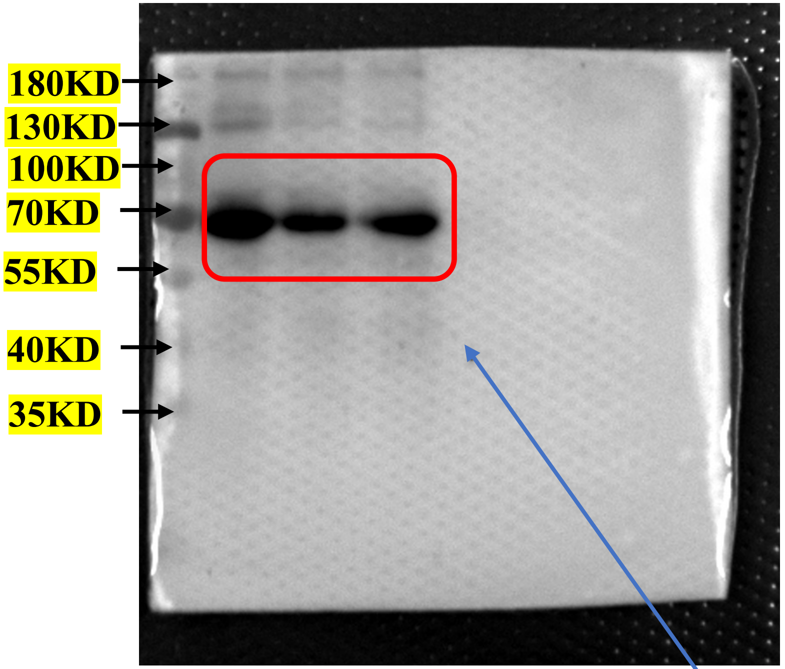

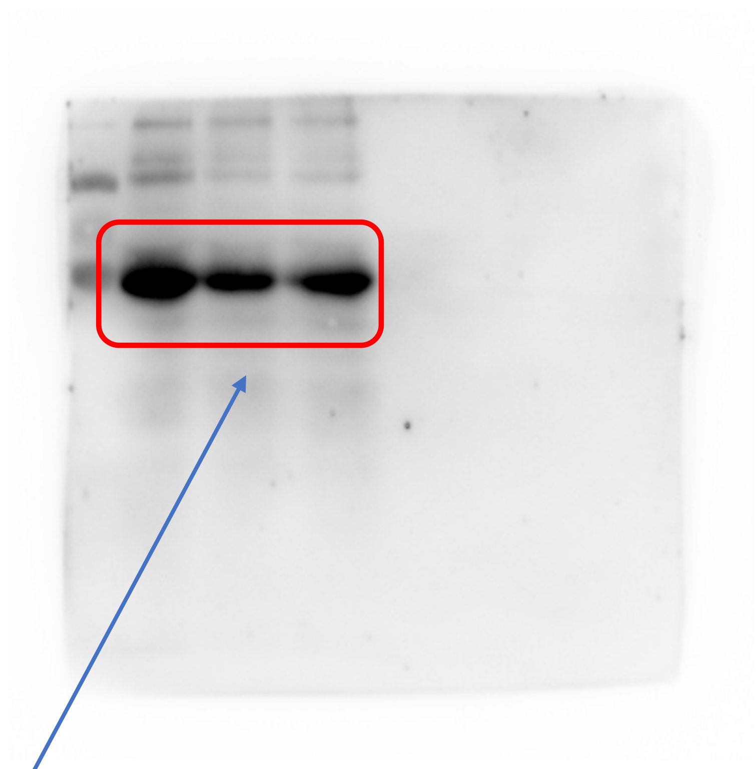

Human sample #3: PPARg (≈60KD)

Antibody: PPARg antibody (Cell signaling technology #81B8)

Figure S5c

Full unedited gel for Figure S5c

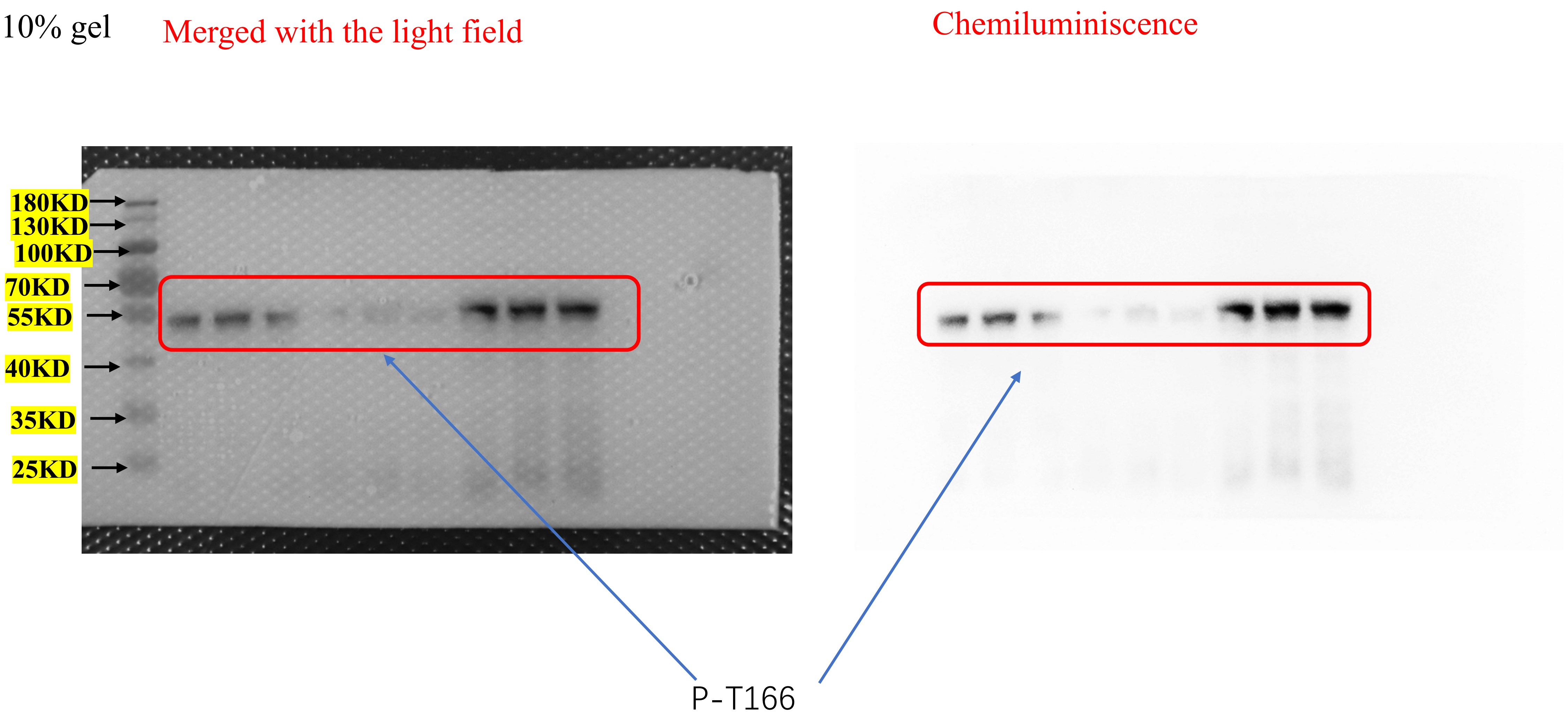

Antibody: T166 phosphorylation antibody generated by our lab

Full unedited gel for Figure S5c

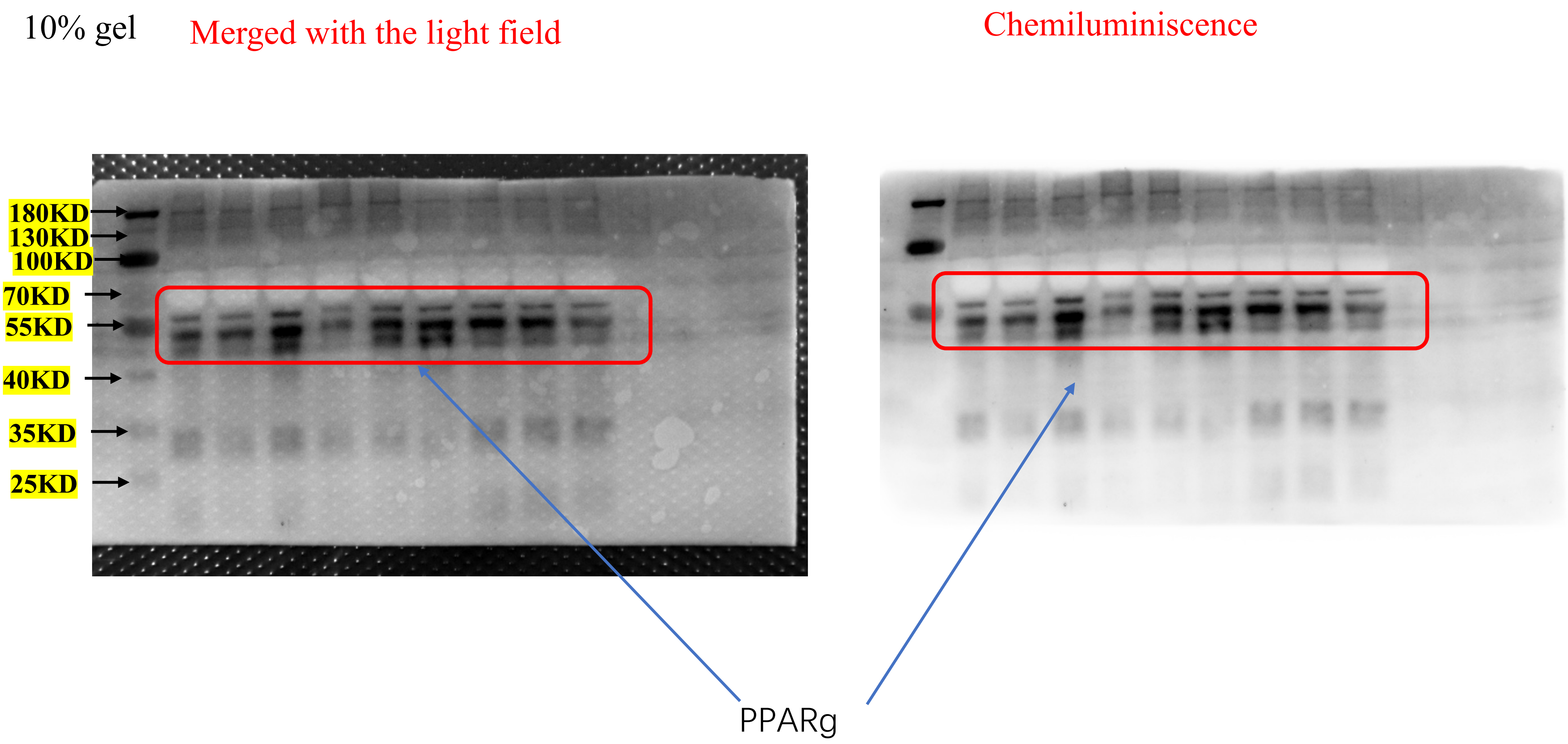

Antibody: PPARg antibody (Cell signaling technology, #81B8)

Full unedited gel for Figure S5c

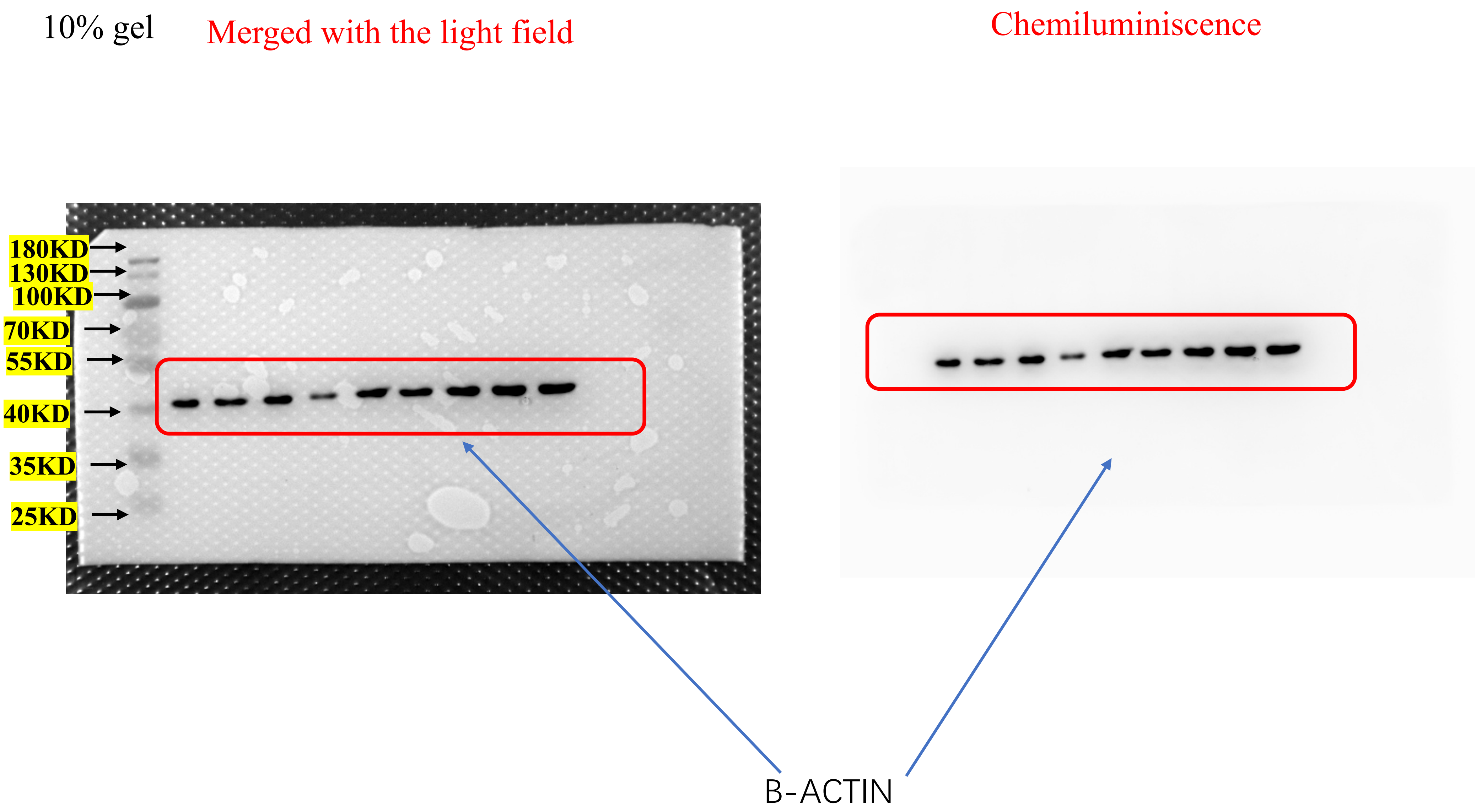

Antibody: b-Actin (Genscript A00702)

Figure S5d

Full unedited gel for Figure S5d

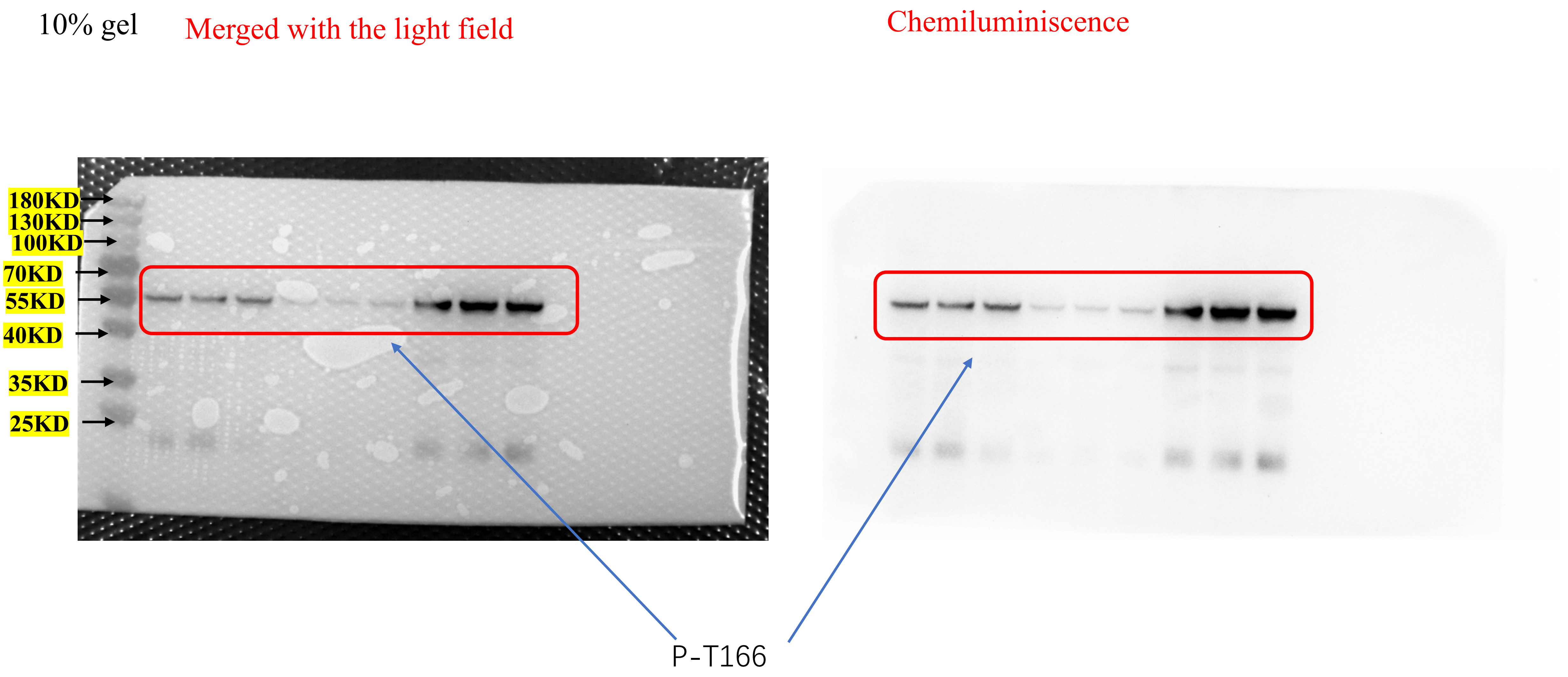

Antibody: T166 phosphorylation antibody generated by our lab

Full unedited gel for Figure S5c

10% gel Merged with the light field Chemiluminiscence

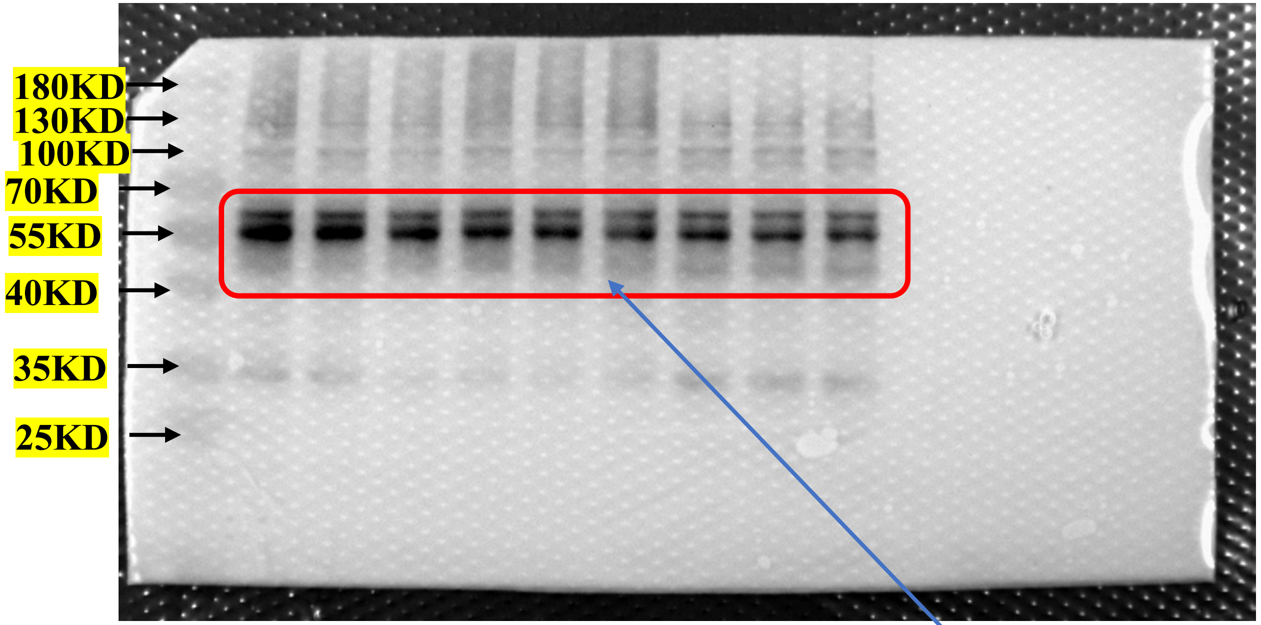

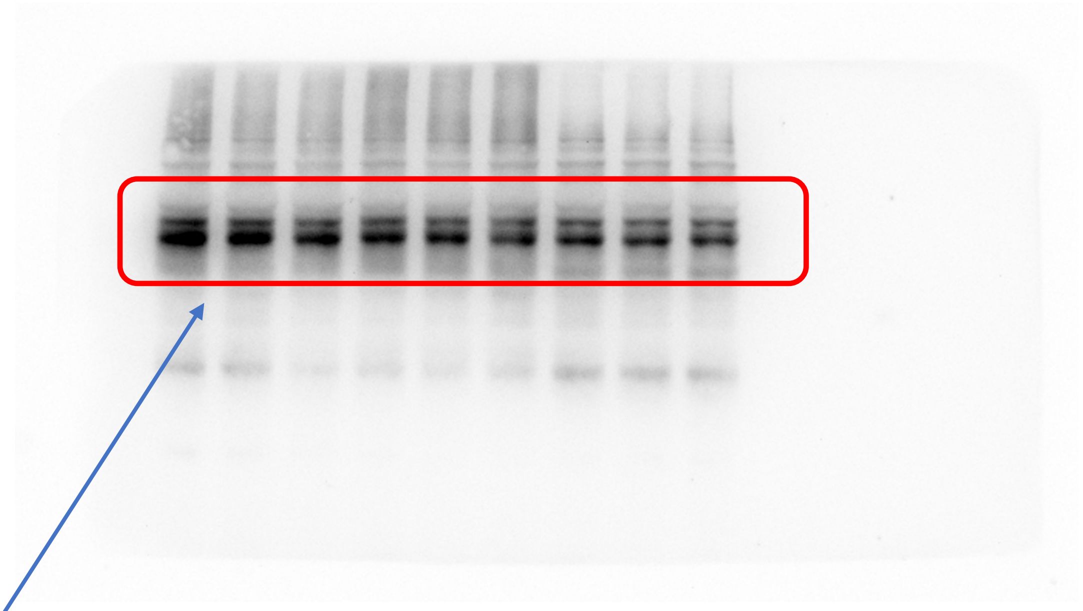

PPARg

Antibody: PPARg antibody (Cell signaling technology, #81B8)

Full unedited gel for Figure S5c

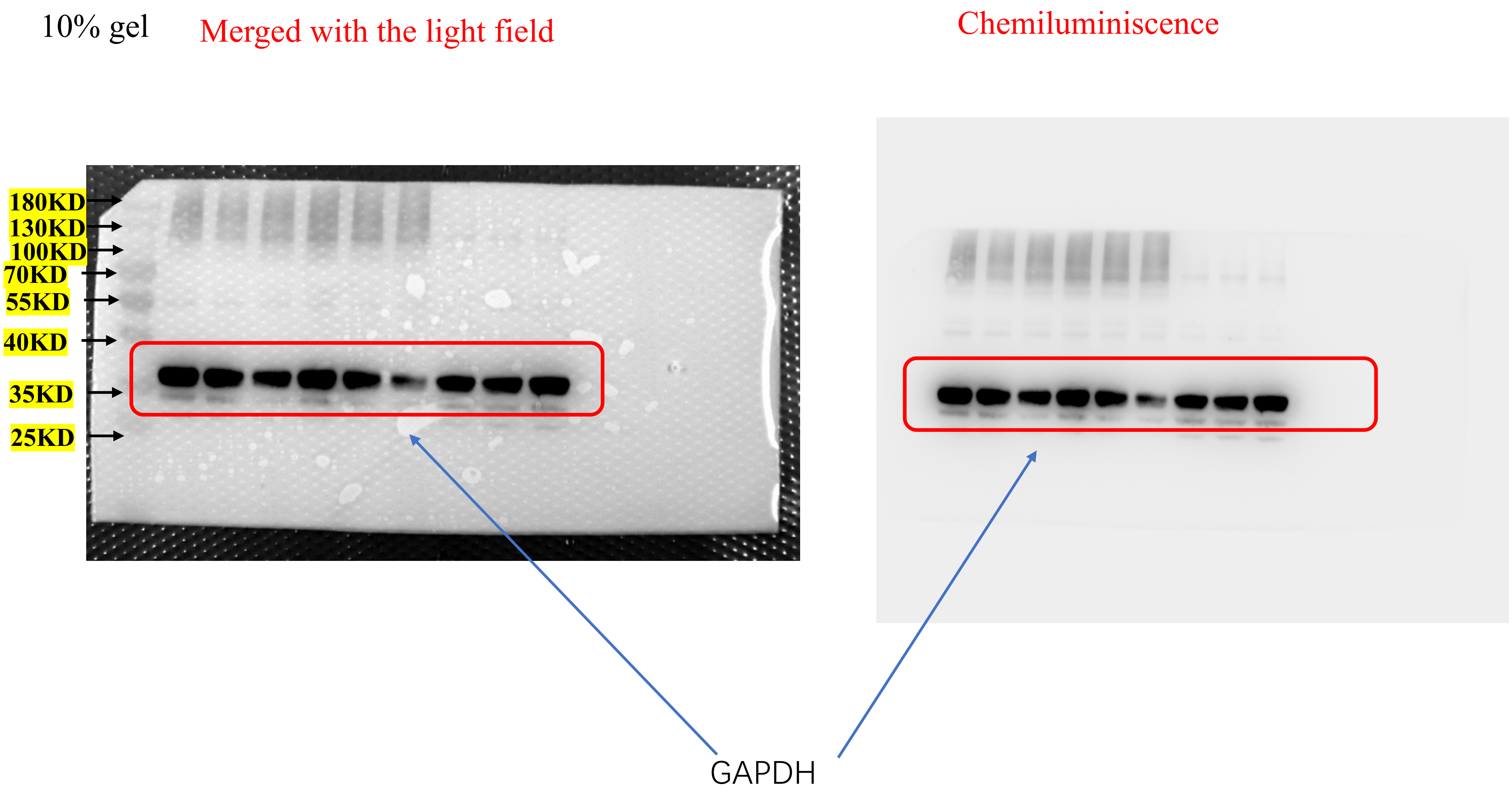

Figure S7a

Full unedited gel for Figure S7a

10% gel Merged with the light field Chemiluminiscence

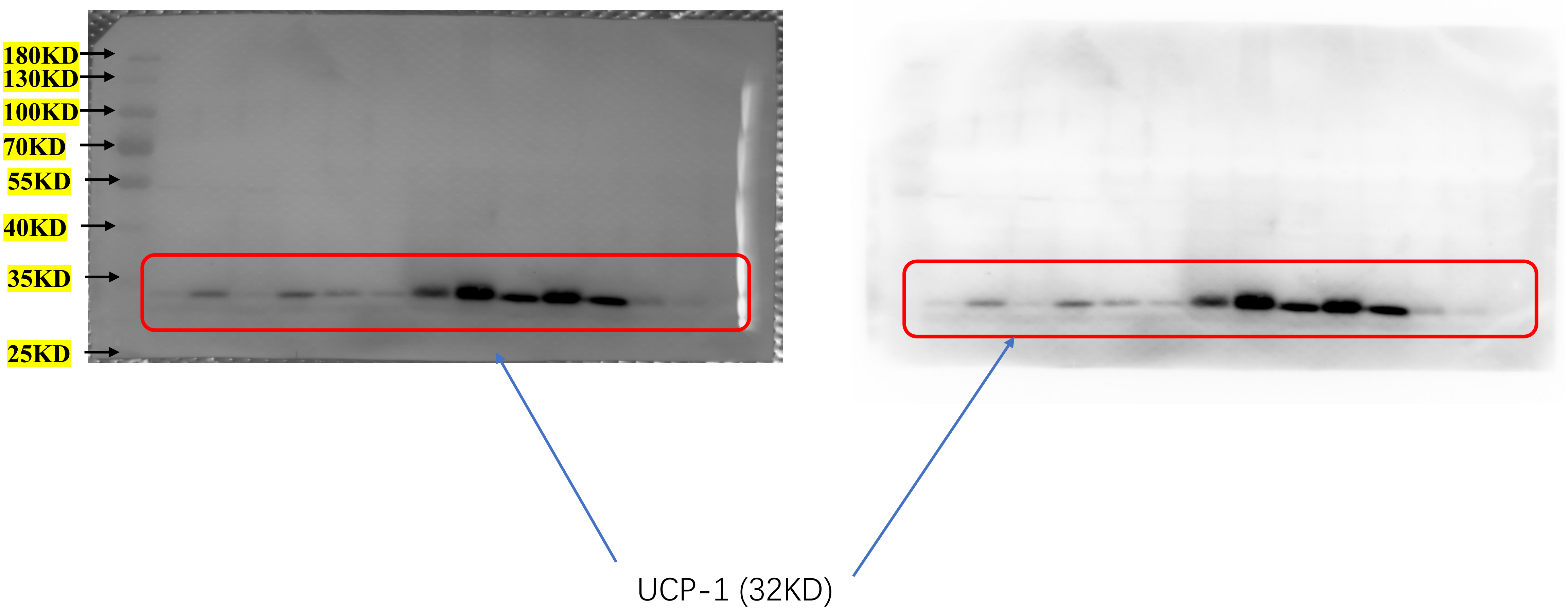

Antibody: UCP1 antibody (Abcam ab209483)

Full unedited gel for Figure S7a

10% gel

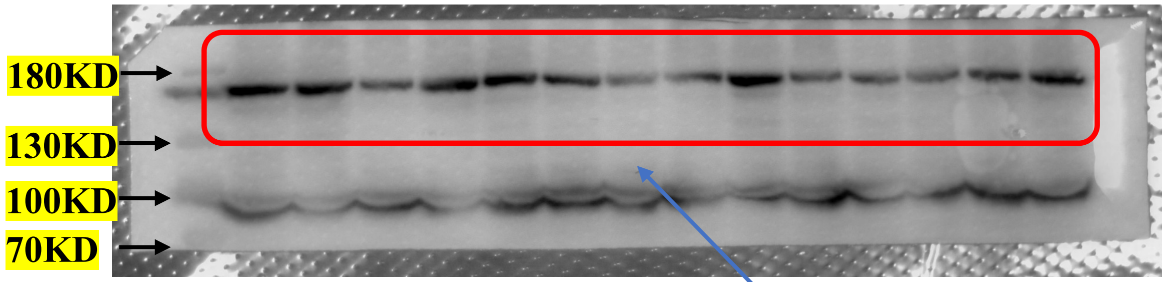

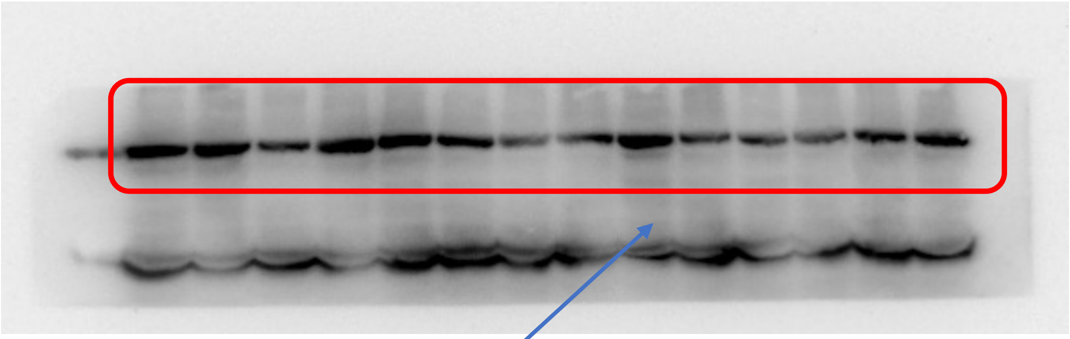

endogenous PRDM16 (≈150KD)

Antibody: PRDM16 antibody (Abcam ab106410)

Full unedited gel for Figure S7a

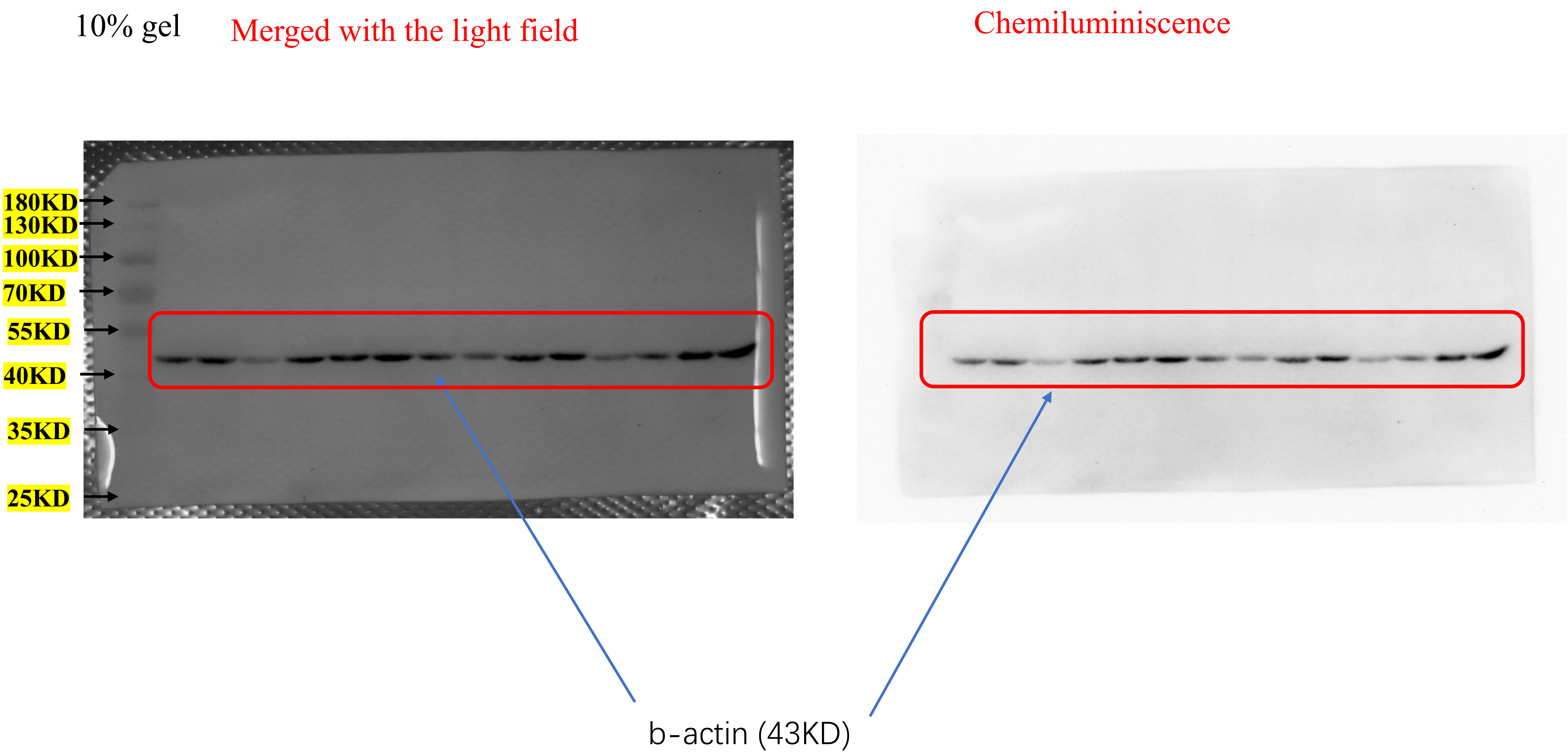

Antibody: b-Actin (Genscript A00702)

Figure S11d

Full unedited gel for Figure S11d

10% gel

Merged with the light field

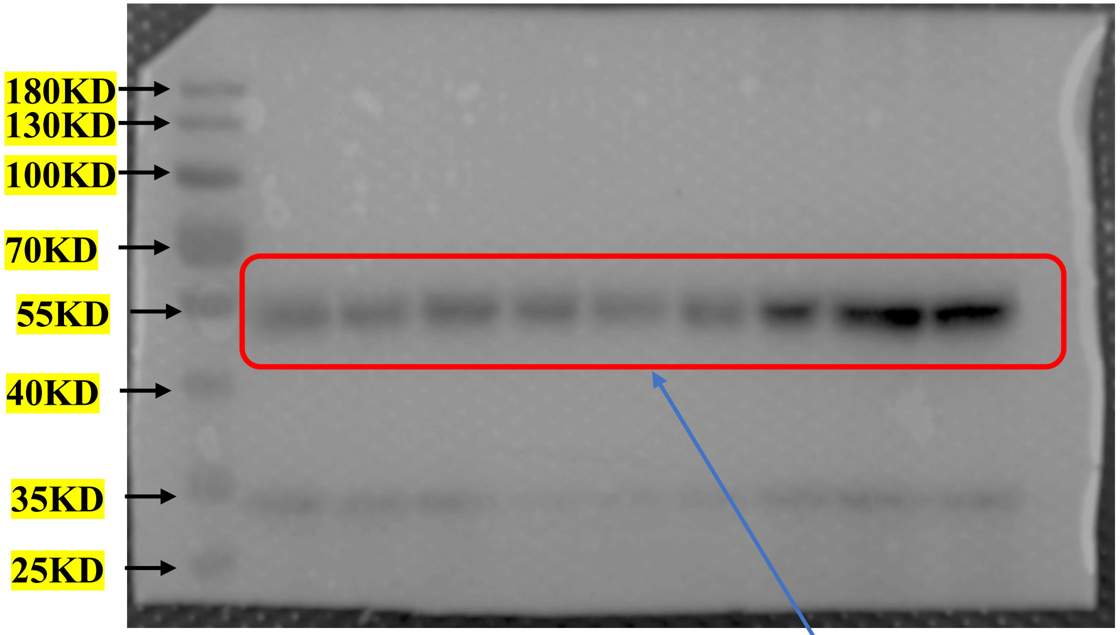

Chemiluminiscence

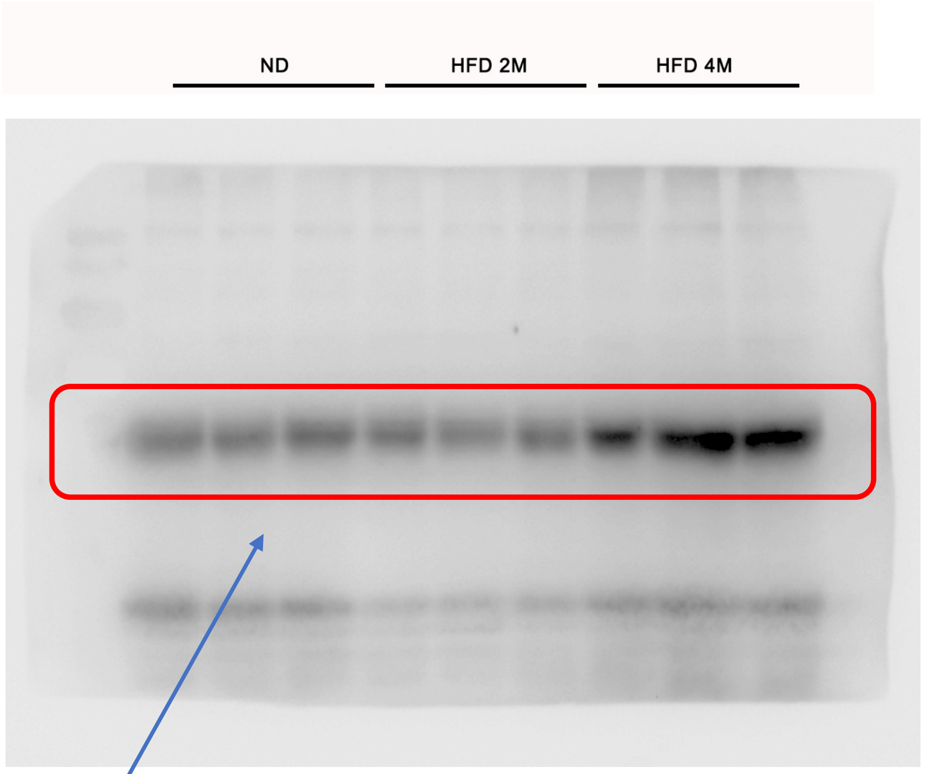

p-T166 (55KD)

Antibody: T166 phosphorylation antibody generated by our lab

Full unedited gel for Figure S11d

10% gel

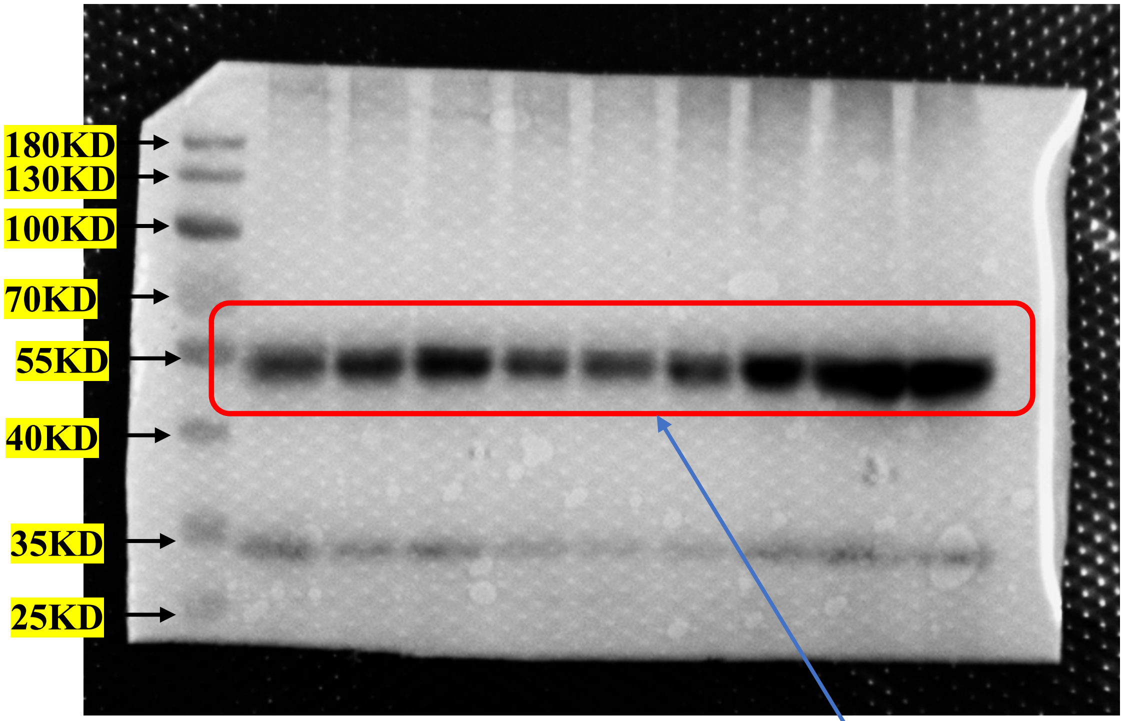

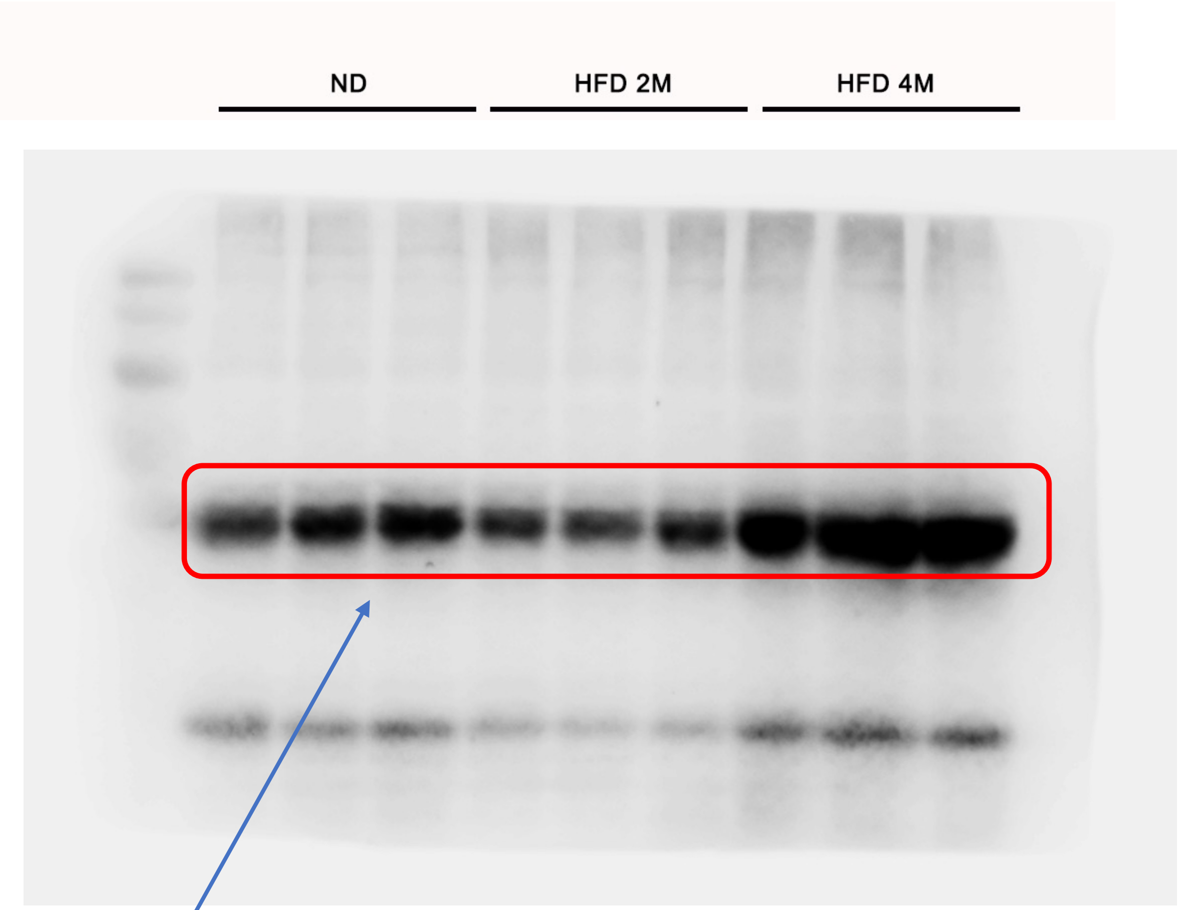

PPARg (55KD)

Antibody: PPARg antibody (Santa Cruz sc-7273)

Full unedited gel for Figure S11d

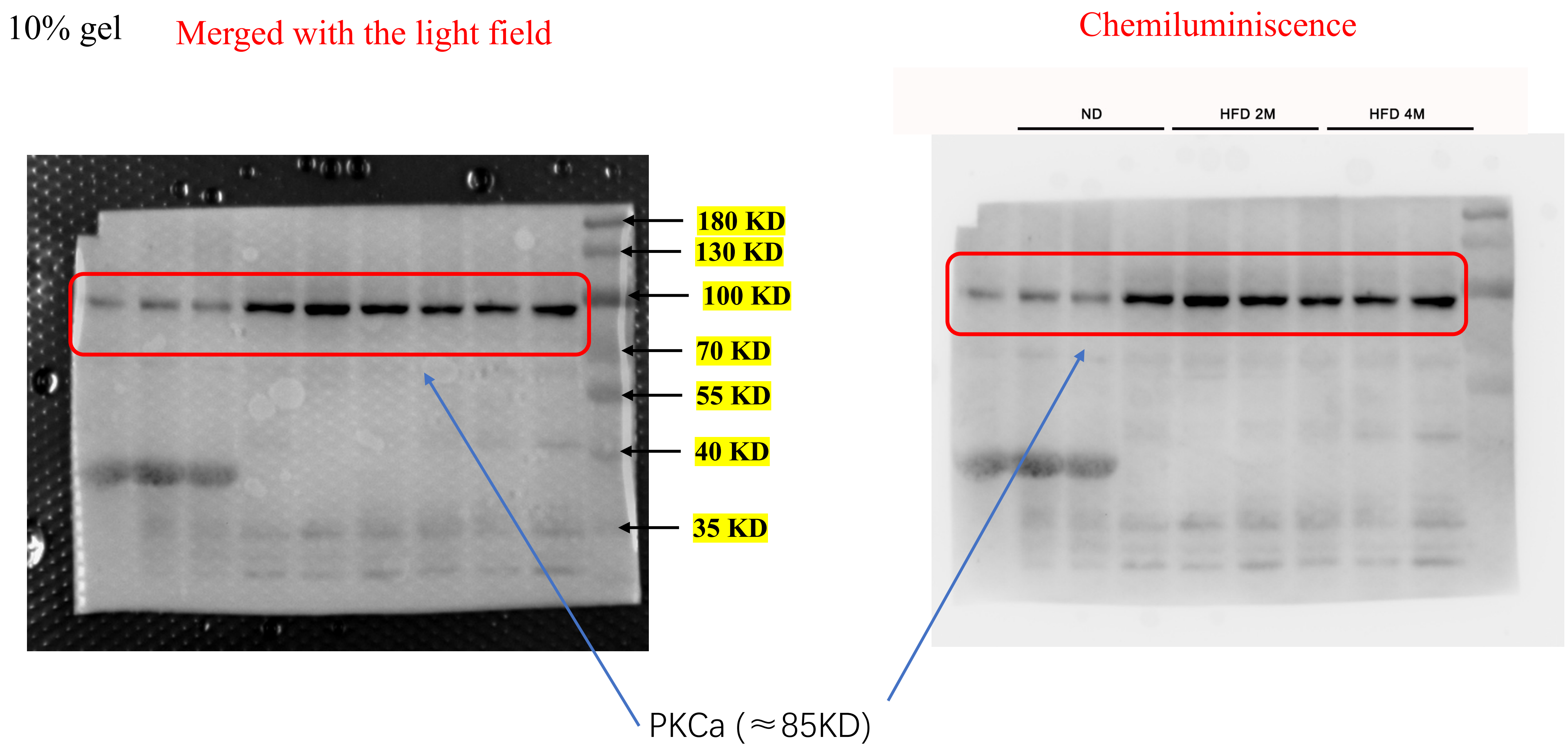

Antibody: PKCa antibody (Cell signaling technology #2056)

Full unedited gel for Figure S11d

10% gel Merged with the light field

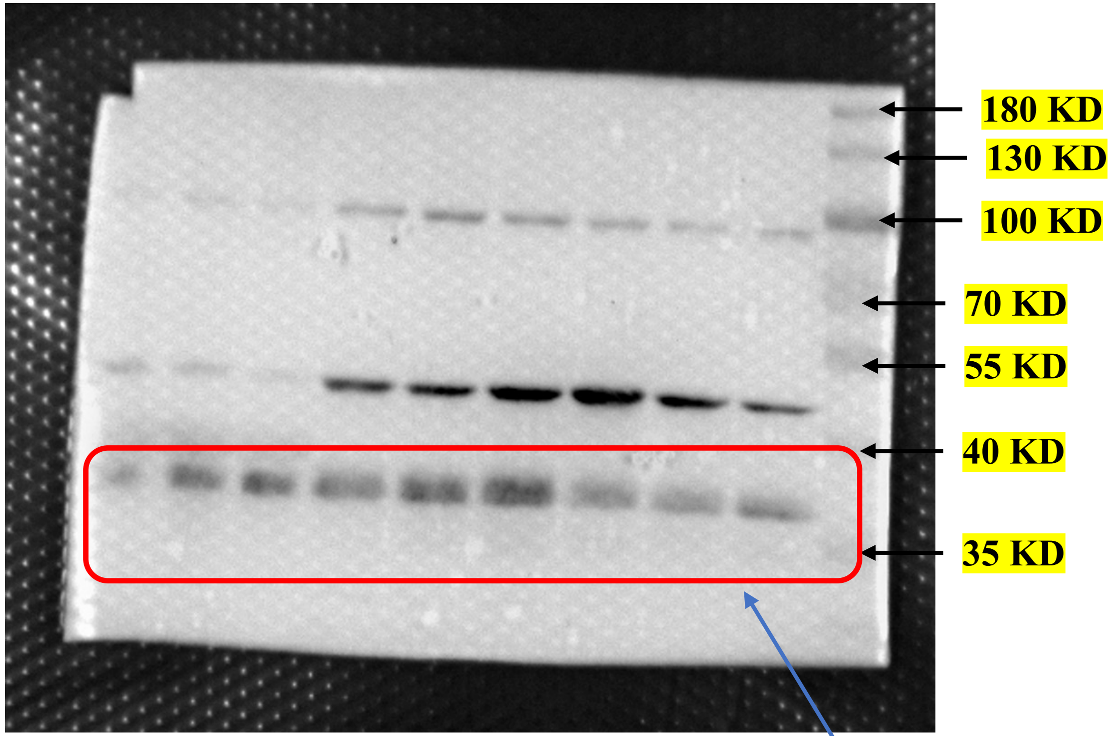

Chemiluminiscence

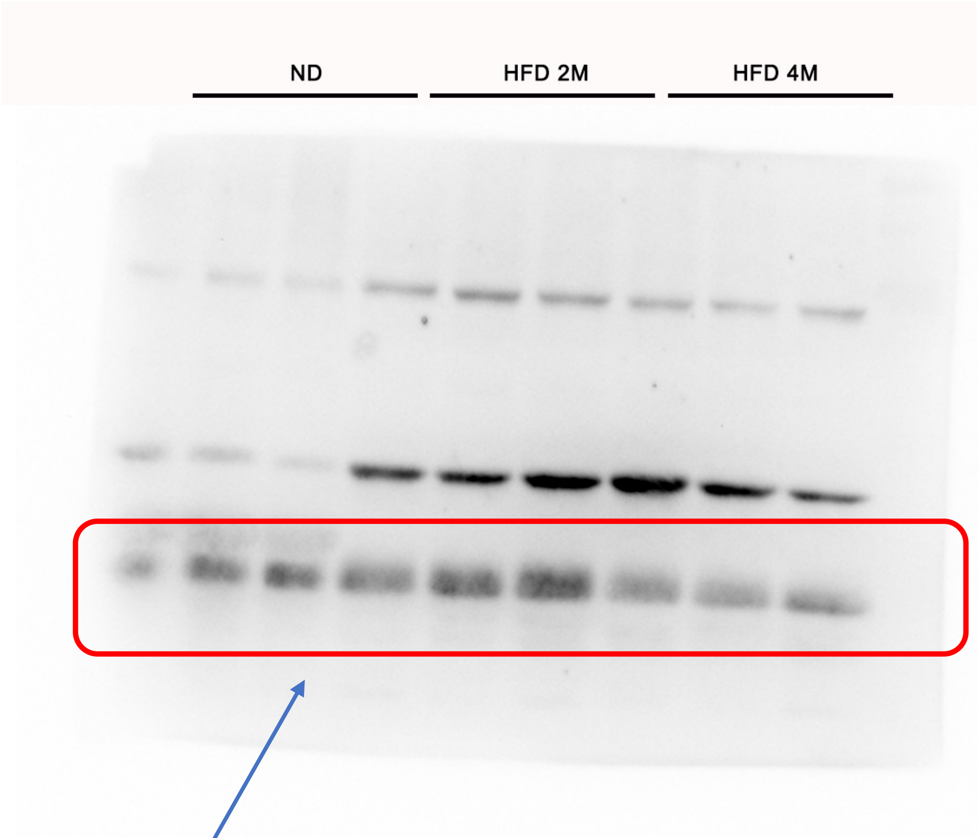

GAPDH (37KD)

Figure S11f

Full unedited gel for Figure S11f

8% gel

Merged with the light field

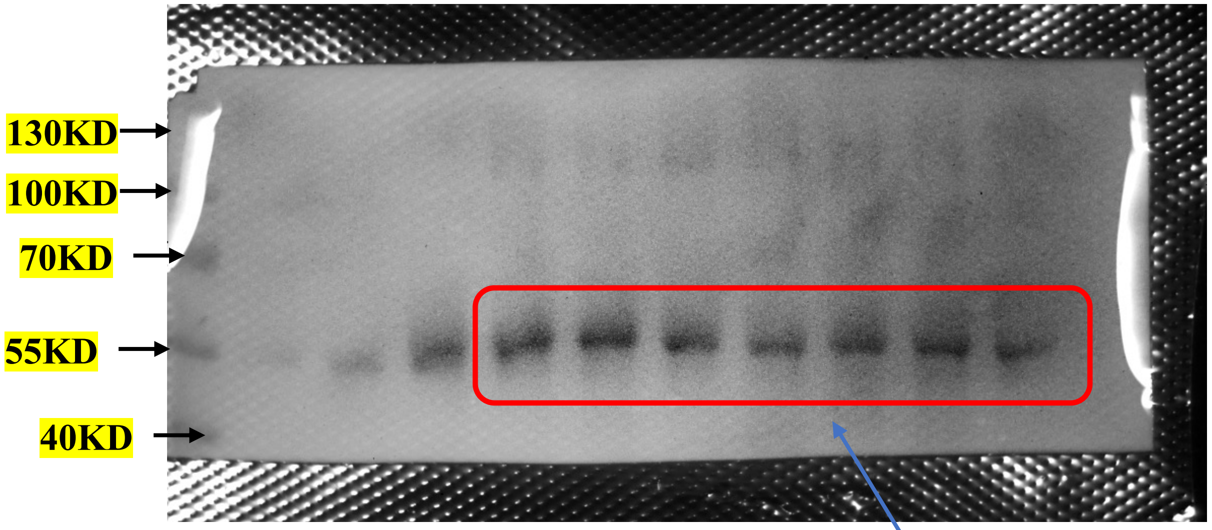

Chemiluminiscence

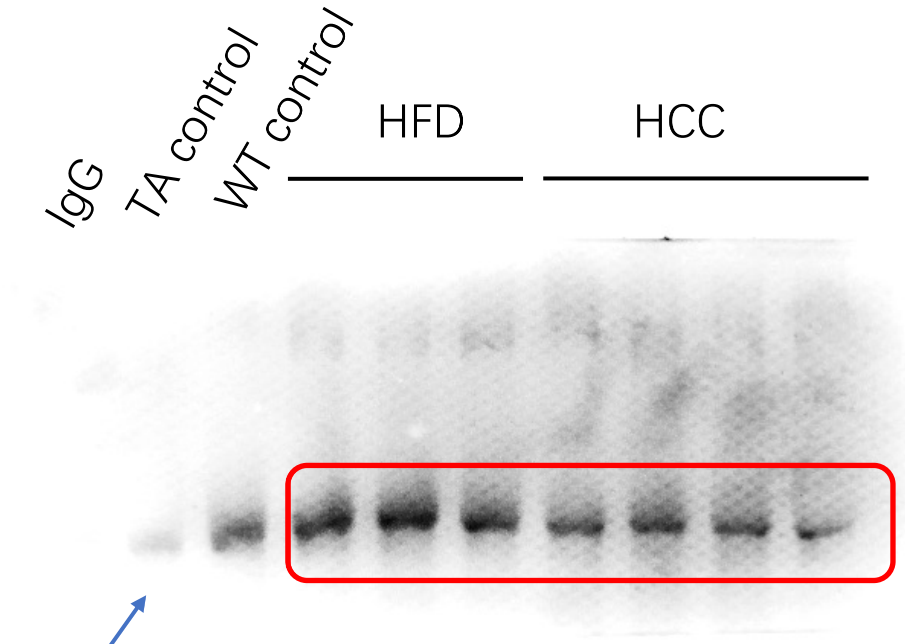

p-T166 (55KD)

Antibody: T166 phosphorylation antibody generated by our lab

TA control: protein lysis from normal diet TA mice SAT

WT control: protein lysis from normal diet WT mice SAT

Full unedited gel for Figure S11f

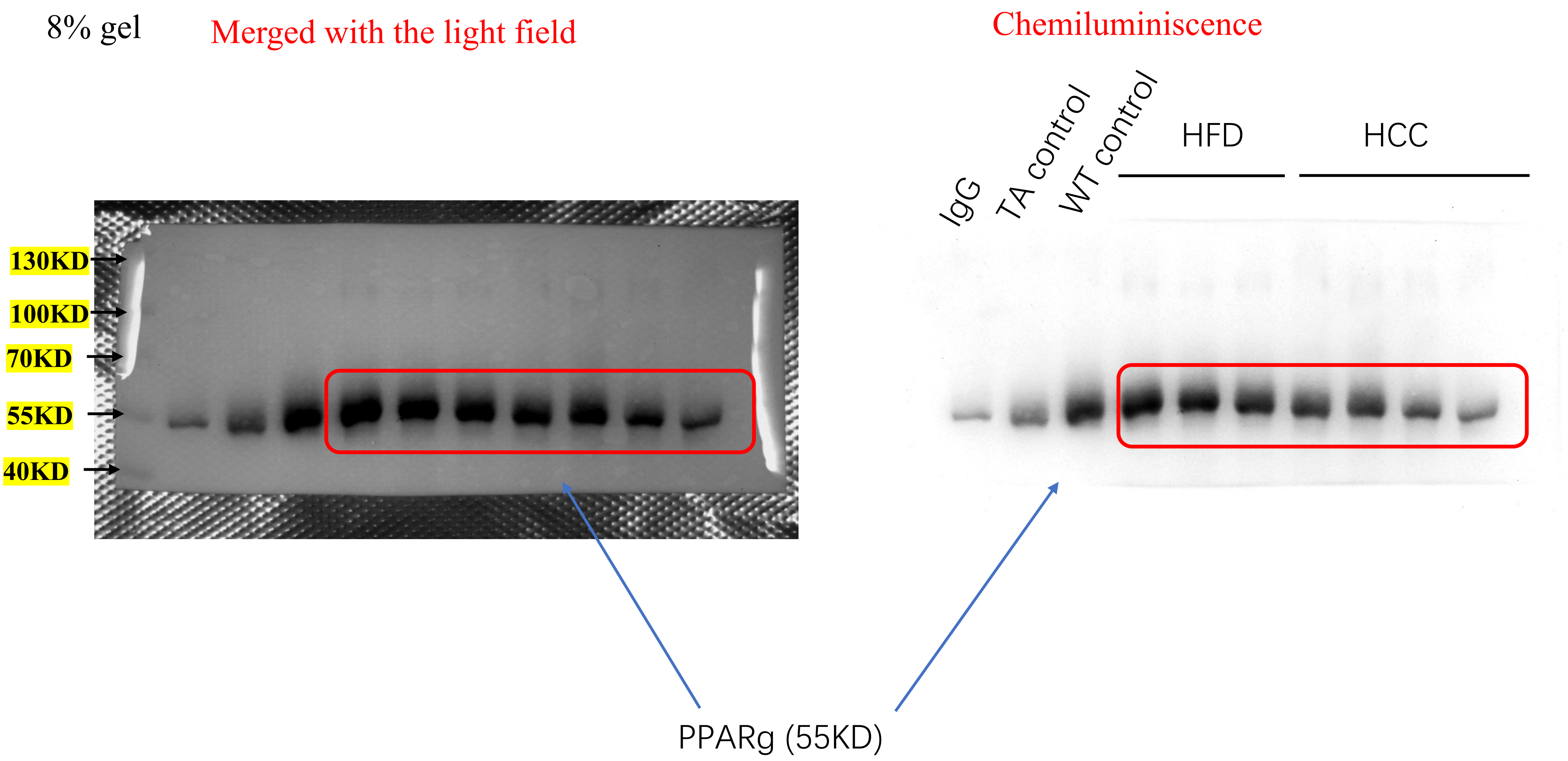

Antibody: PPARg antibody (Cell signaling technology #81b8)

Full unedited gel for Figure S11f

8% gel

Merged with the light field

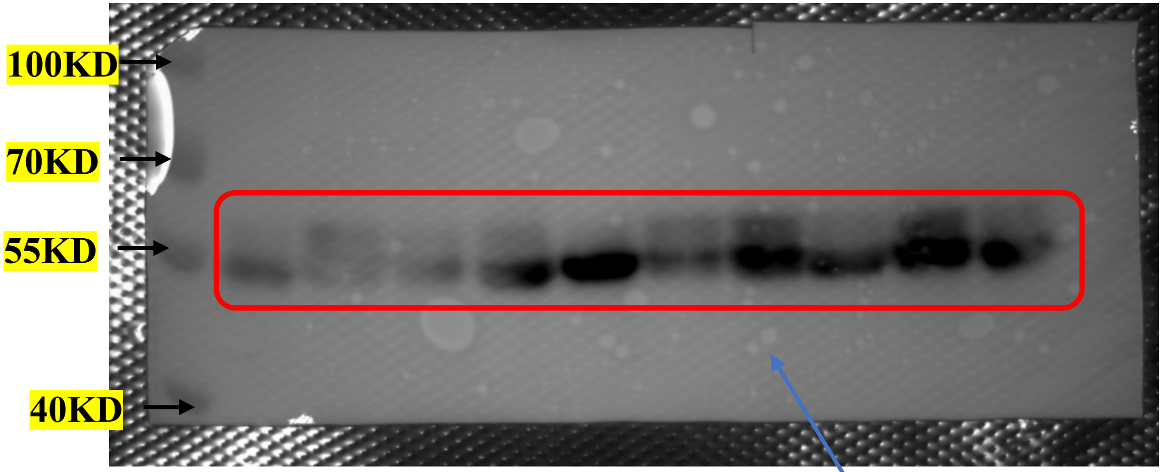

Chemiluminiscence

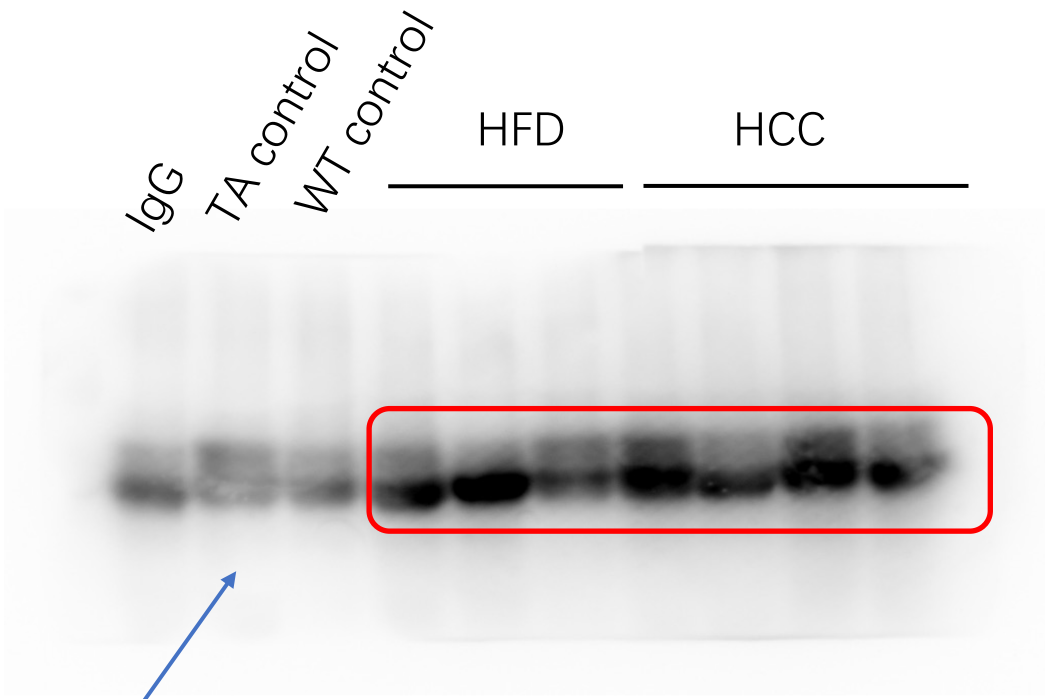

PPARg (55KD)

Full unedited gel for Figure S11f

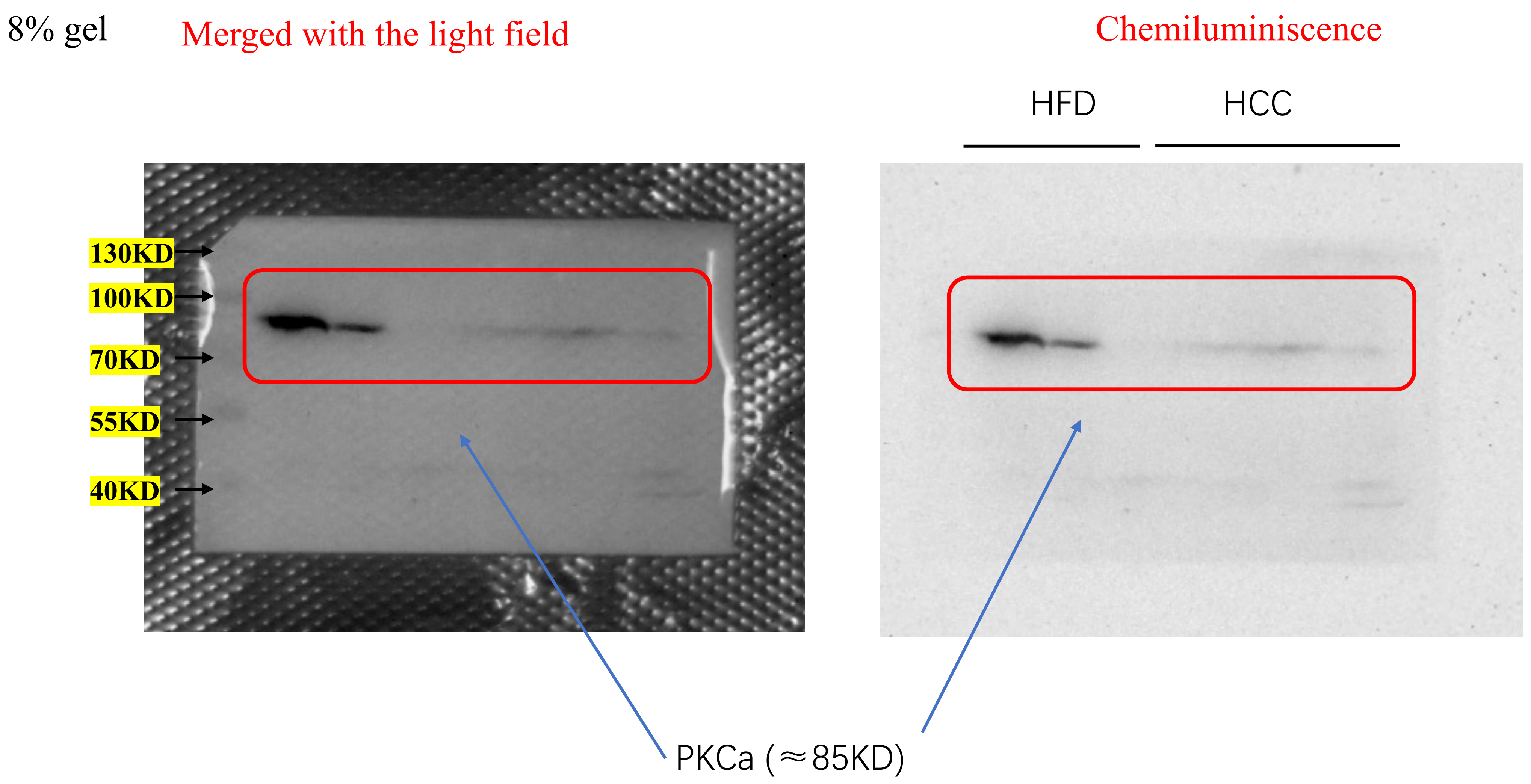

Antibody: PKCa antibody (Cell signaling technology #2056)

Figure S11i

Full unedited gel for Figure S11i

10% gel Merged with the light field

Chemiluminiscence

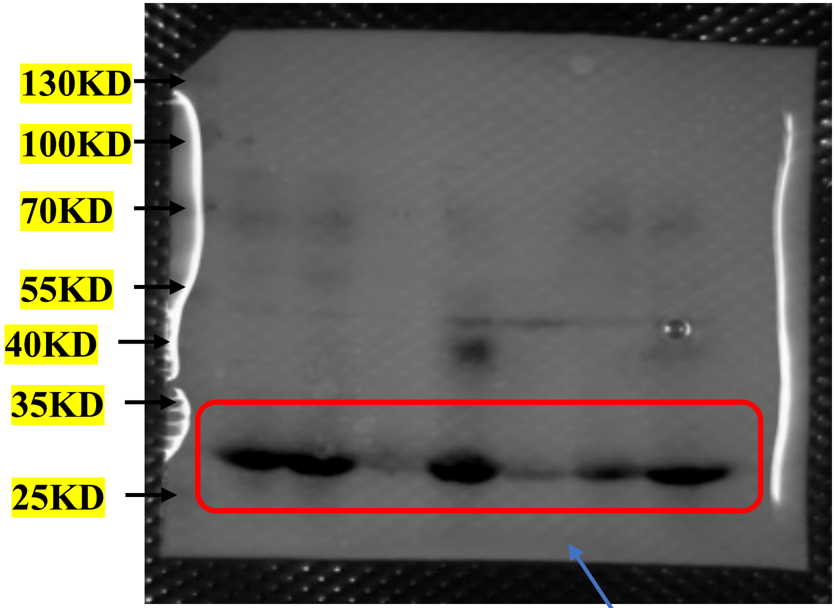

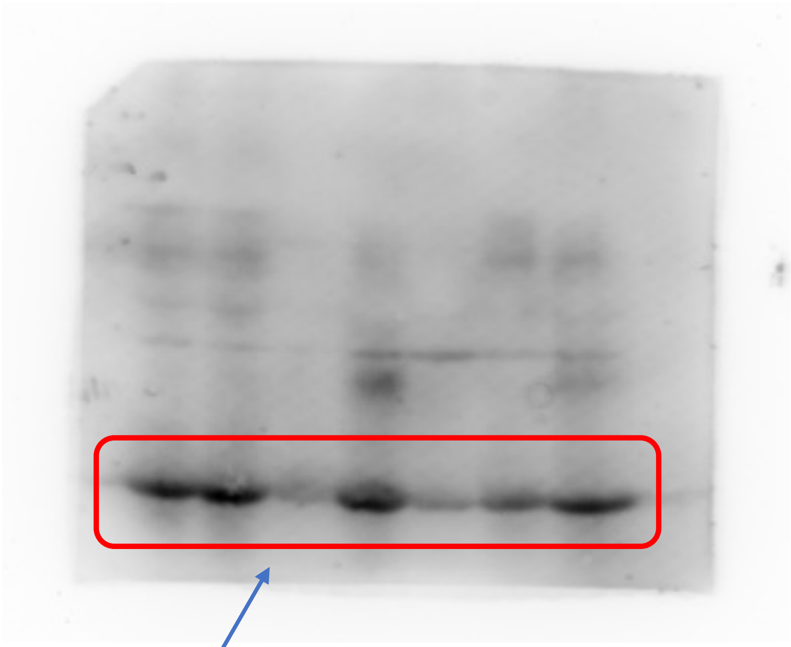

Antibody: p-CDK5 antibody
(Bioss bs-6411R)

p-CDK5 (30KD)

Full unedited gel for Figure S11i

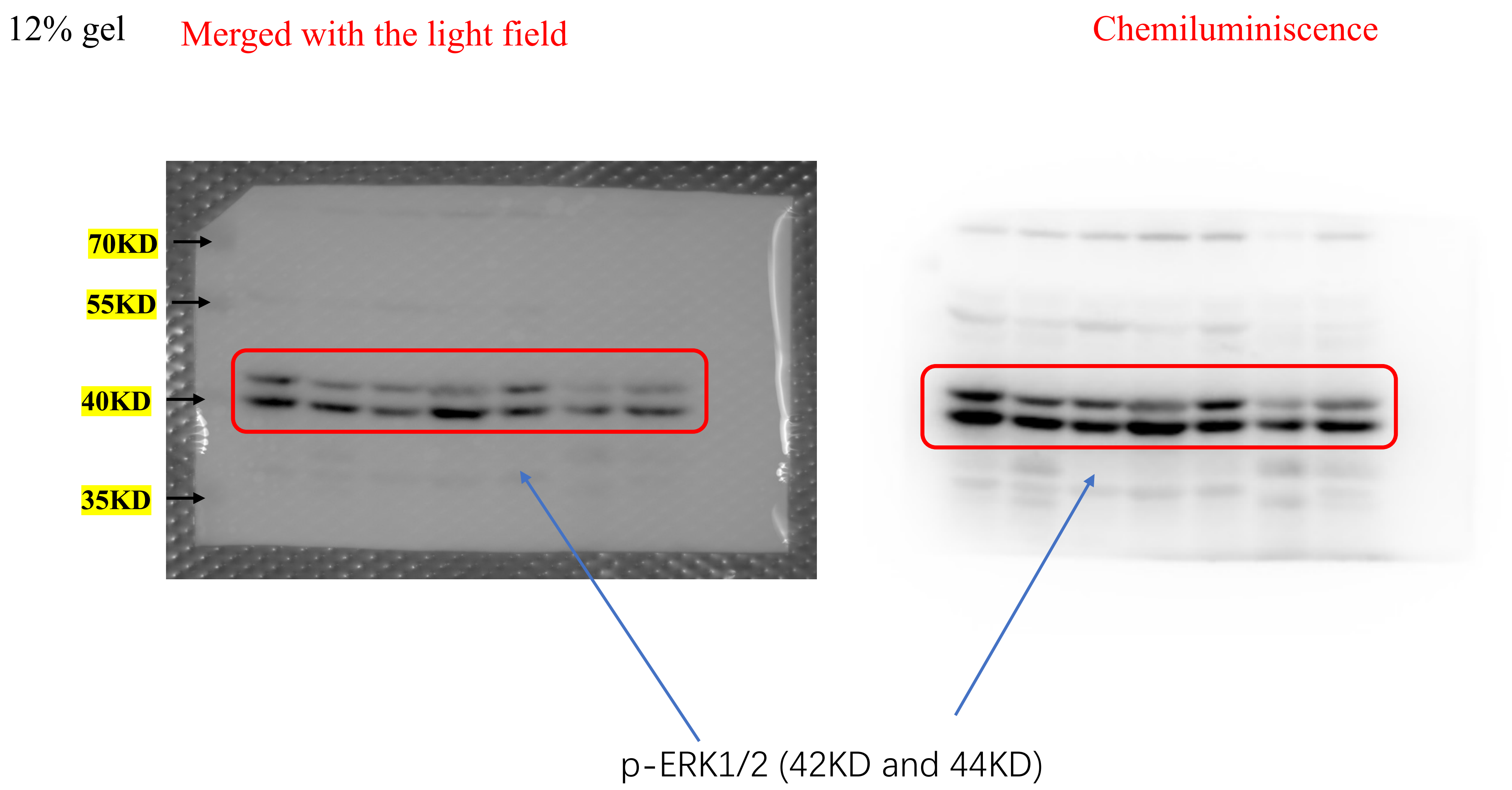

Antibody: p-ERK1/2 antibody
(ab223500, Abcam)

Full unedited gel for Figure S11i

12% gel Merged with the light field Chemiluminiscence

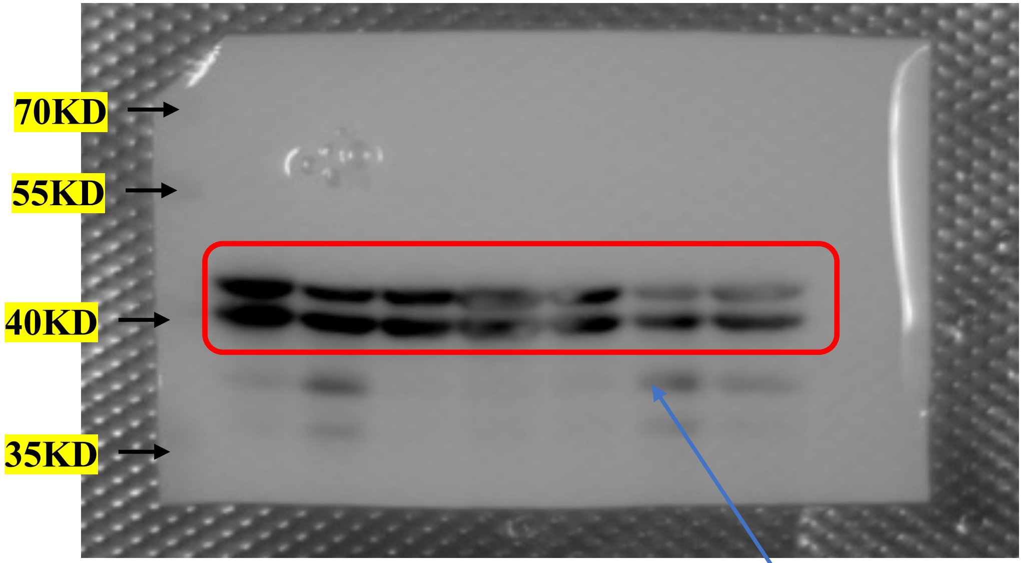

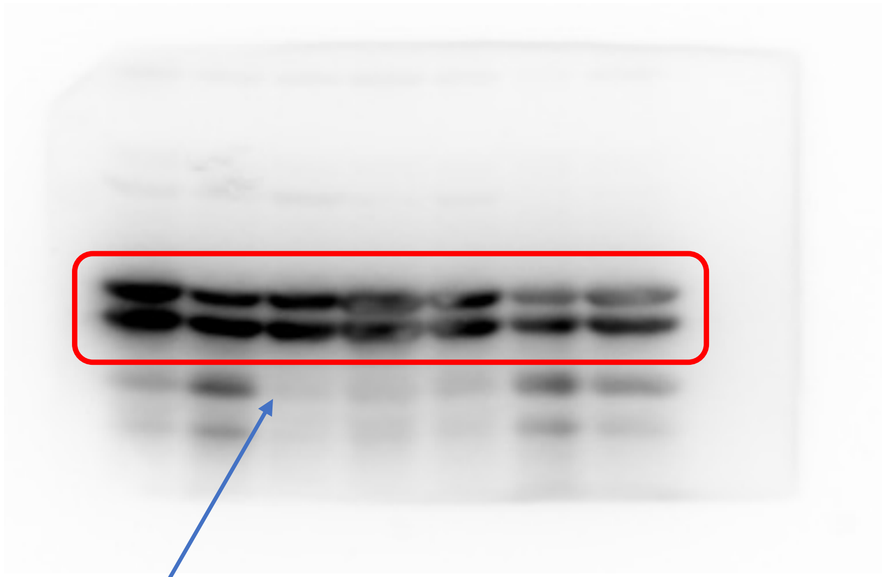

ERK1/2 (42KD and 44KD)

Antibody: p-ERK1/2 antibody
(ab17942, Abcam)

Full unedited gel for Figure S11i

12% gel

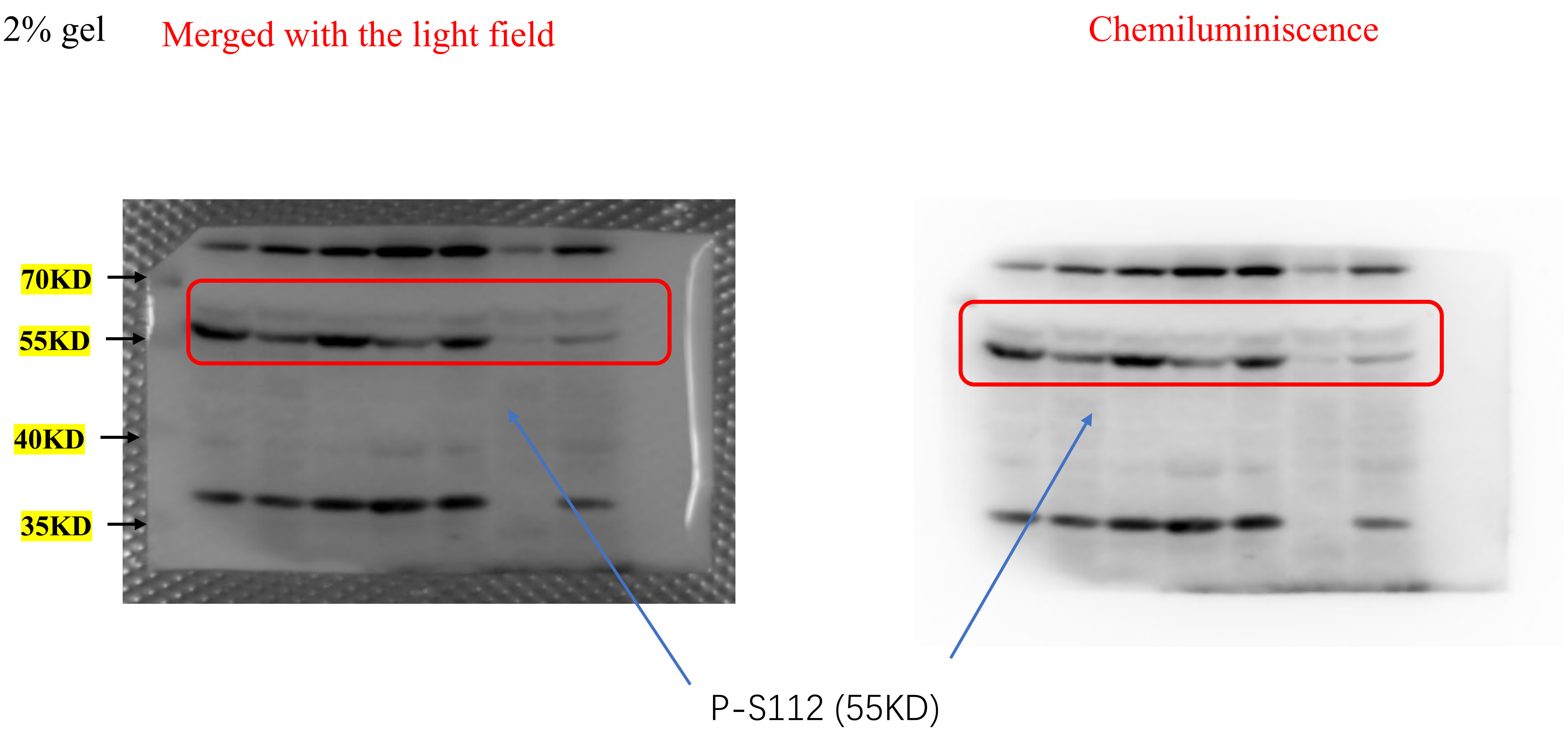

Antibody: p-ERK1/2 antibody
(ab195925, Abcam)

Full unedited gel for Figure S11i

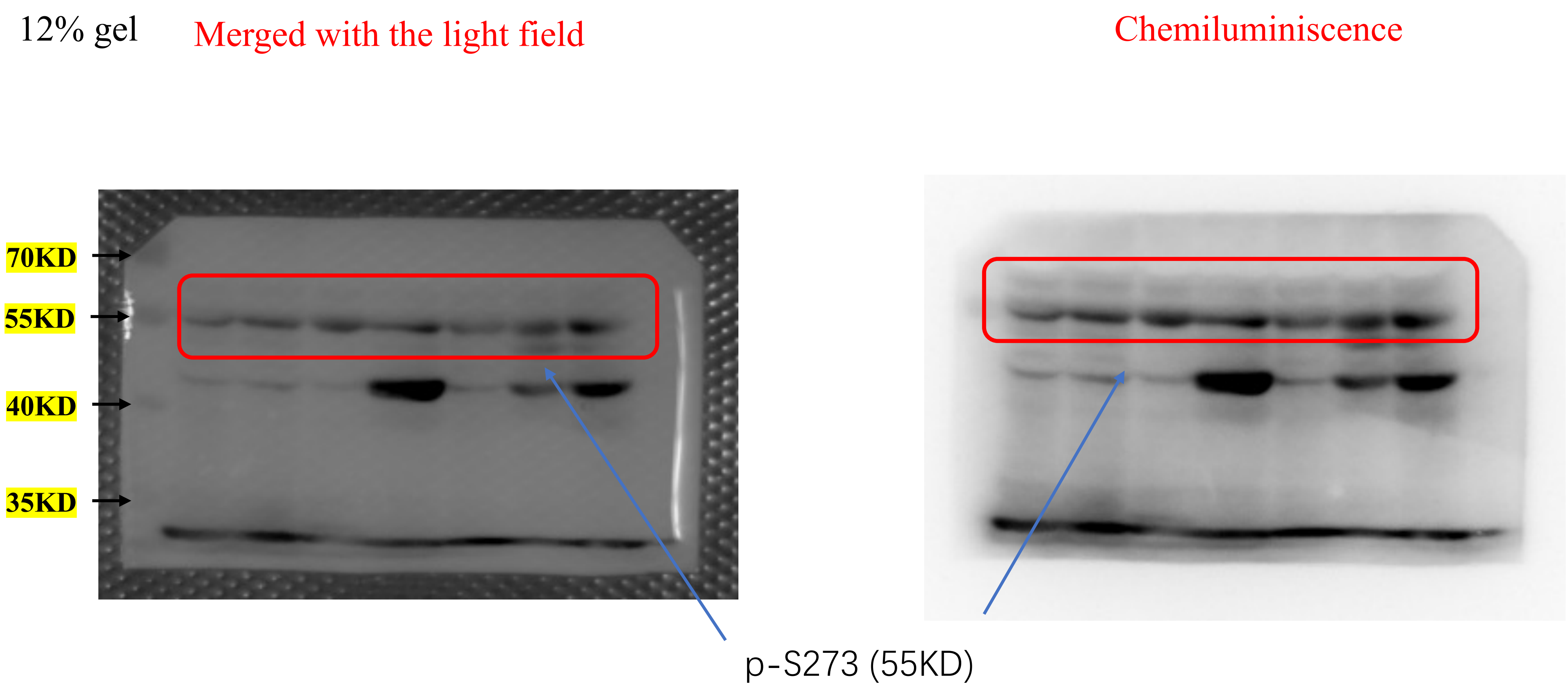

Antibody: p-ERK1/2 antibody
(bs-4888R, Bioss)

Full unedited gel for Figure S11i

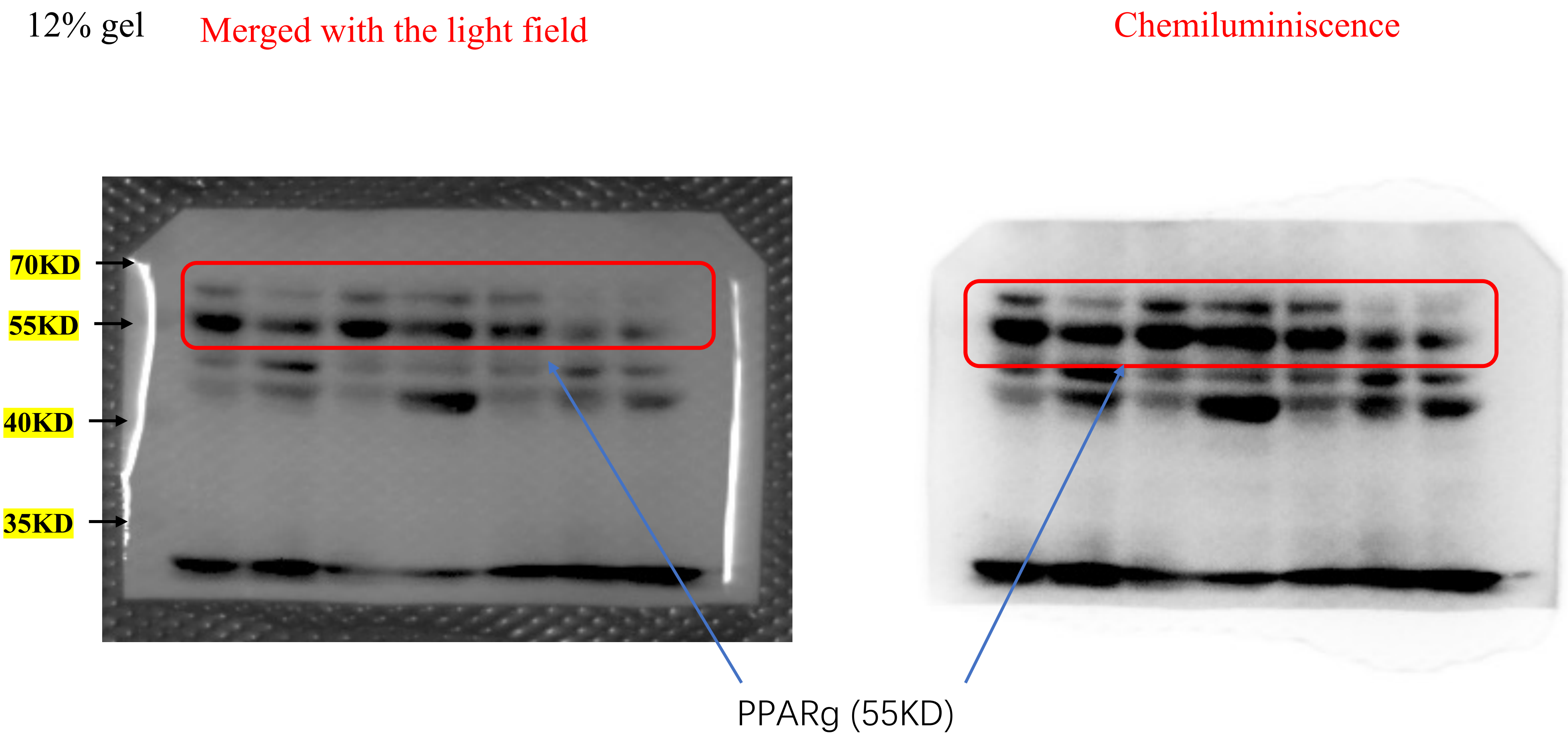

Antibody: PPARg antibody (#81B8, Cell signaling technology)

Full unedited gel for Figure S11i

10% gel

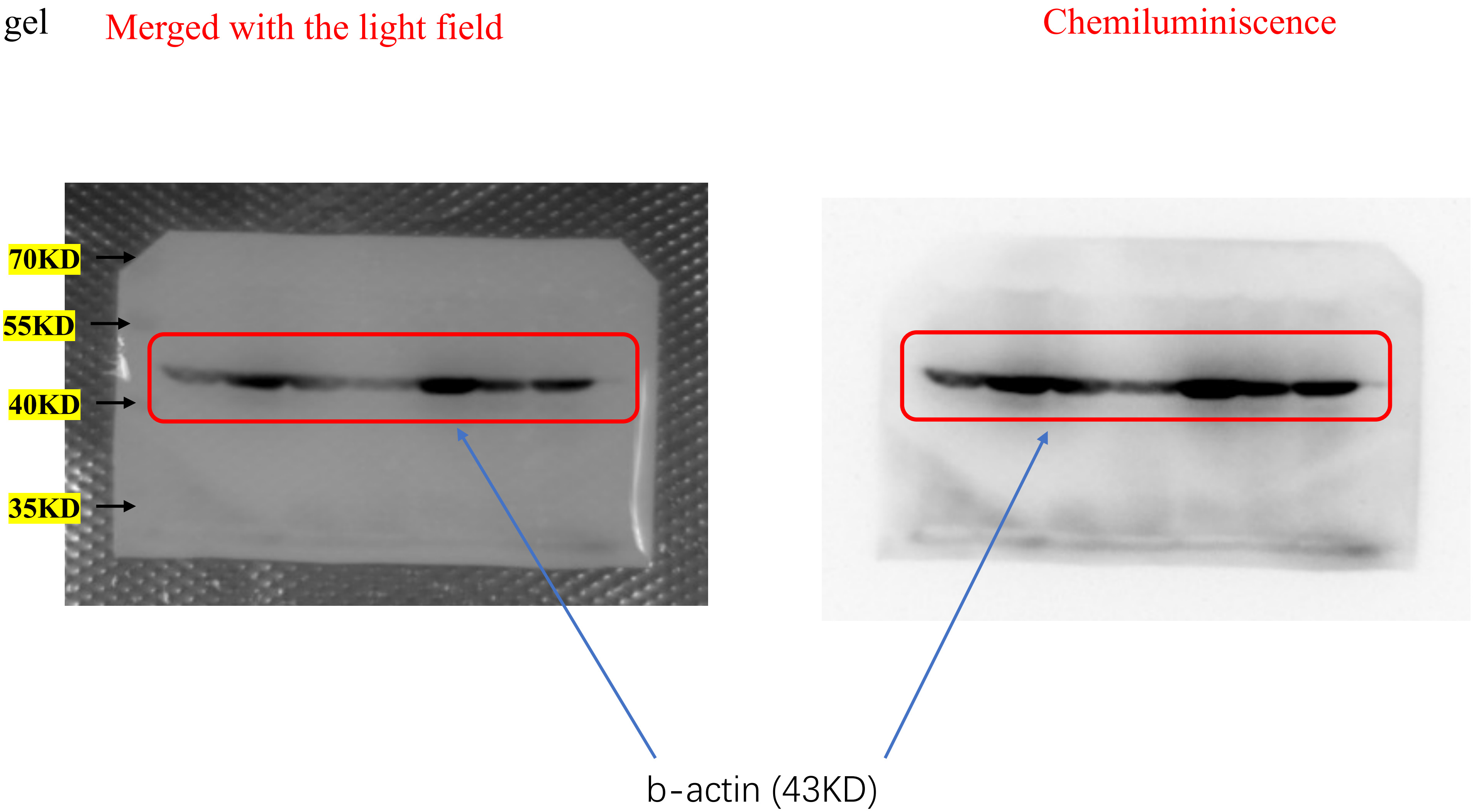

Antibody: b-Actin (Genscript A00702)
